# Bioinspired microcapsule reactor with engineered probiotics for IBD therapy

**DOI:** 10.1038/s41467-026-72027-1

**Published:** 2026-07-13

**Authors:** Minhui Xu, Yuanchun Du, Guangfu Feng

**Affiliations:** 1 College of Bioscience and Biotechnology, Hunan Agricultural University, Changsha, Hunan China; 2 College of Chemistry and Materials Science, Hunan Agricultural University, Changsha, Hunan China

**Keywords:** Ulcerative colitis, Synthetic biology, Drug delivery

## Abstract

Although probiotic-based bionic strategies show therapeutic promise for inflammatory bowel disease, their clinical translation is limited by poor gastric acid survival, inefficient intestinal colonization and inadequate targeting. Inspired by the multi-level cooperative mechanism of defense protection–danger sensing–tissue repair observed in coral communities, we developed a core–shell bionic microcapsule reactor (MY-E@SS). Here we show that the multifunctional bionic shell enables safe delivery of engineered bacteria through the gastrointestinal tract. Upon reaching inflamed intestinal sites, these bacteria sense the pathological microenvironment and responsively release an anti-inflammatory peptide. In a male murine model of inflammatory bowel disease, this system exhibited excellent biocompatibility and pronounced therapeutic efficacy, restoring intestinal barrier integrity, attenuating systemic inflammation and oxidative stress, modulating respiratory metabolism, and reestablishing microbial homeostasis. Mechanistically, therapeutic effects were attributed to inhibition of TNF-α/NF-κB signaling pathway. This work provides an intelligent platform to modulate inflammatory microenvironments and advance therapies for complex diseases.

## Abstract (web summary)

The authors developed a bionic microcapsule containing engineered bacteria that sense gut inflammation and release targeted therapy. Microcapsule treatment in mouse model of inflammatory bowel disease restored intestinal barrier function.

## Introduction

By emulating structures, functions, and mechanisms from biological systems, bionics draws inspiration from nature to address complex challenges at the interface of engineering and medicine^1–3^. Within biomedical engineering, bionic strategy are employed in areas such as tissue engineering and drug delivery to create therapeutic platforms with improved biocompatibility and superior performance^4,5^. The core superiority of bionic strategy lies in its capacity to leverage solutions refined by millions of years of natural evolution, enabling the design of precise, efficient, and low-toxicity therapies for complex diseases^6–8^.

Inflammatory bowel disease (IBD) is a heterogeneous group of chronic, inflammatory intestinal disorders, primarily represented by ulcerative colitis (UC) and Crohn’s disease (CD)^9^. The pathogenesis of IBD is multifactorial, with core pathological mechanisms involving intestinal barrier dysfunction, immune dysregulation, microbial dysbiosis, and oxidative stress. This pathogenic progression develops into more serious complications over time, including intestinal ischemia, hemorrhage, and dysplasia^10–12^. Notably, IBD is not confined to the gastrointestinal tract, up to 40% of patients develop extraintestinal subclinical pathologies^13^. Gut-derived pathogenic factors, including inflammatory mediators and microbial metabolites, can gain access to the peripheral circulation^14^. Intestinal dysbiosis can exacerbate pulmonary inflammation by altering adaptive immune responses, thereby promoting respiratory metabolic dysregulation or acute lung injury (ALI)^15^. The current clinical management of IBD centers on pharmacologic agents, including 5-aminosalicylic acid (5-ASA), antibiotics, corticosteroids, and immunosuppressant^16^. Although these therapies can attenuate inflammation, they largely fail to address key pathological features such as impaired mucosal repair, barrier dysfunction, and microbial dysbiosis. Meanwhile, they often lack the capacity to rapidly reinstate essential physiological functions compromised during active disease, notably respiratory metabolism. Long-term use is also associated with adverse effects including acneiform rash, peripheral edema, nausea, as well as potential hepatorenal toxicity, elevated infection risks, and drug dependence^17,18^.

Against this backdrop, probiotic-based therapeutics have garnered significant interest as a potentially safer and more holistic alternative, capitalizing on their inherent biocompatibility and capacity to remodel the gut microbiota. Specifically, genetically engineered probiotics offer the capability for the precise, targeted expression of therapeutic proteins, holding considerable promise for biomimetic therapy^19,20^. However, current engineered bacterial strategies for the treatment of IBD remain constrained by several critical delivery limitations. Key challenges range from inadequate spatiotemporal control of therapeutic release and low targeting efficiency to susceptibility to degradation in the harsh gastrointestinal environment, poor colonization capability, and interference from reactive oxygen species (ROS)^21,22^. To address these challenges, we propose a biomimetic strategy that emulates the intelligent repair mechanisms of living organisms in response to pathological microenvironments. The aim is to improve both the survival and colonization efficiency of engineered bacteria in the digestive tract while equipping them with the ability to sense inflammatory signals and release therapeutic agents in a responsive manner. Notably, the design of this therapeutic strategy directly echoes the multi-layered survival mechanisms observed in natural coral colonies. Corals can sense and respond to environmental stressors by activating a series of defense and repair mechanisms. Their calcareous exoskeletons provide physical protection, while secreted mucus serves as a biochemical barrier against adverse conditions^23^. This mucus facilitates stable adhesion to substrates and contains immunologically active compounds, including antimicrobial peptides, effectively counteracting infections. Additionally, symbiotic microorganisms associated with corals enhance the immune response of their host by scavenging ROS and reducing oxidative stress^23,24^. This multi-layered and synergistic defense system, refined over millions of years of evolution, exhibits exceptional environmental adaptability and provides a valuable paradigm for addressing complex biomedical problems. In bionic design, we selected coral as the model because its exoskeleton–mucus–symbiont hierarchical cooperative architecture provides a highly compatible blueprint for addressing the synergistic challenges of probiotic survival, colonization, and inflammatory response in oral delivery. Compared to other stress-responsive systems (e.g., rapid closure in plants or the immune cascade in animals), this model more intuitively integrates the dual functions of long-term protection and intelligent response, directly inspiring the core defense protection–danger sensing–tissue repair structure of this work.

**Fig. 1:**
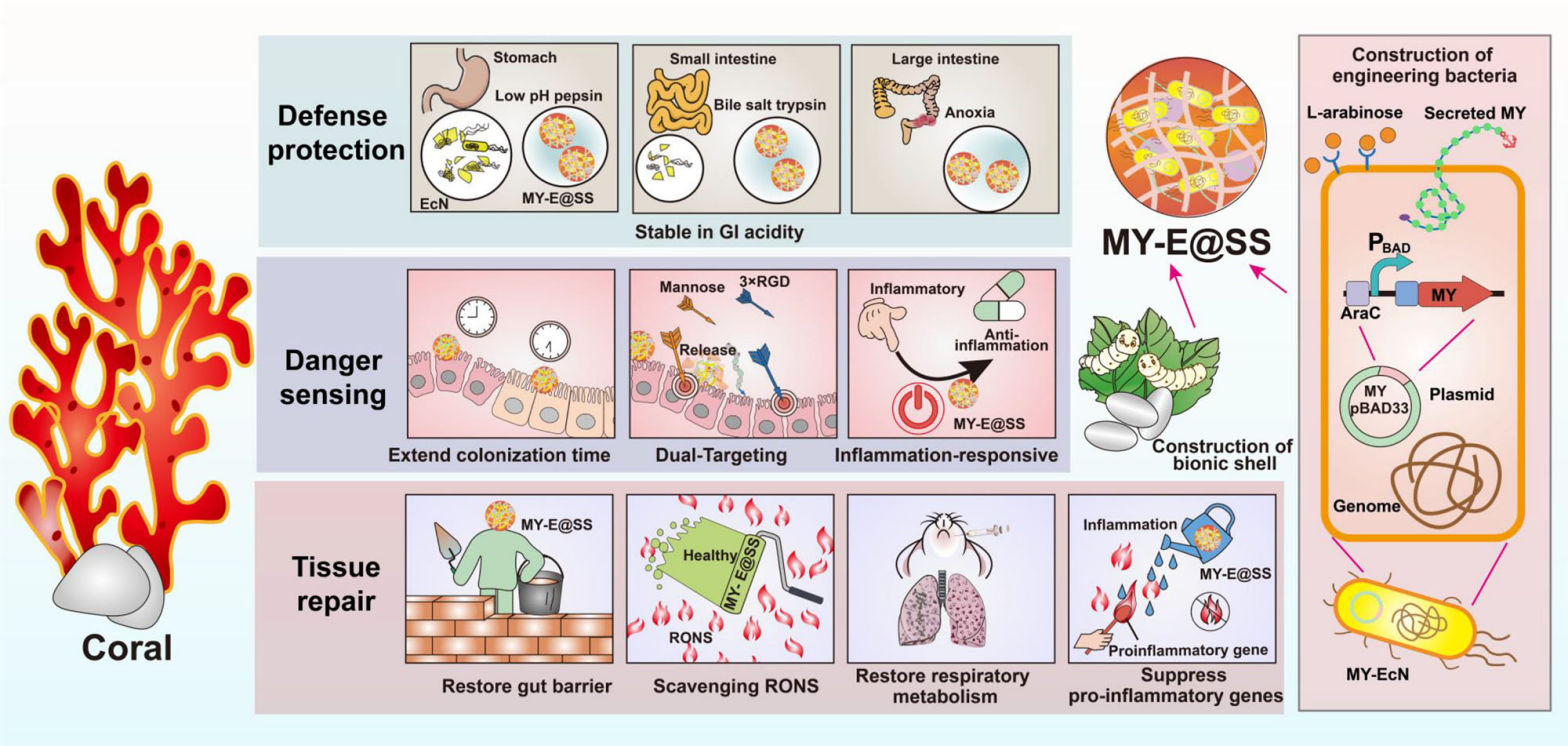
An IBD therapy was designed using a bioinspired coral microcapsule reactor with engineered probiotics. Inspired by the cascade strategy of defense protection - danger sensing - tissue repair employed by corals in response to adverse environments, this work aims to develop a bionics-based approach for the management of IBD. RGD Arg-Gly-Asp; GI Gastrointestinal; RONS Reactive oxygen and nitrogen species.

Inspired by the adaptive mechanisms of corals, we have engineered a bionic microcapsule reactor (MY-E@SS) through the integration of synthetic biology and biomimetic principles. It operates by implementing a biomimetic defense protection–danger sensing–tissue repair cascade, mimics the coral survival strategy, for the treatment of IBD (Fig. 1). MY-E@SS was fabricated via droplet microfluidics, with its core–shell structure comprising a biomimetic shell (SS) and engineered bacteria (MY-EcN) encapsulated within it. The MY protein expressed in MY-EcN was designed with a tandem peptide structure. This construct contains an Arg-Gly-Asp (RGD) sequence, which enhances cell adhesion by binding to integrin receptors on intestinal epithelial cells^25,26^. Leveraging this property, the MY protein can be directed to target intestinal epithelial cells. Alongside the RGD motif, the protein was designed to include the inflammation-sensitive Ala-Ala-Pro-Val (AAPV) motif and the anti-inflammatory peptide Lys-Pro-Val (KPV). In the inflammatory microenvironment, the AAPV motif is specifically cleaved by the abundant neutrophil elastase (NE)^25–27^. Thus, within the inflammatory colon, the AAPV-KPV tandem linkage is cleaved, thereby triggering the controlled release of KPV-a process that emulates the integrated danger-sensing and defense response of corals. To mimic the structure of the coral exoskeleton and mucus layer, the SS shell was synthesized via a one-pot chemical method based on silk fibroin (SF), dopamine, and mannose. Dopamine provides additional adhesive properties, while mannose confers targeting functionality; together, they enable the SS shell to scavenge reactive oxygen and nitrogen species (RONS). Furthermore, the SS shell enhances the aggregation density of engineered bacteria and works synergistically with MY-EcN to promote tissue repair, thereby simulating the coral-derived defense–tissue repair process. This design constitutes a synergistic therapy: the shell provides protection and targeted delivery, while the engineered bacteria enable sensing-triggered KPV release. Their combination ensures effective bacterial delivery and yields therapeutic outcomes that surpass the efficacy of either component alone.

Through mathematical modeling (TOPSIS-entropy weight method), the optimized formulation of MY-E@SS demonstrated excellent RONS scavenging ability and biocompatibility. The bionic system demonstrates robust resistance to upper gastrointestinal conditions, precise targeting of inflammatory microenvironments, and controlled release of KPV. Consequently, it significantly down-regulated pro-inflammatory factors (TNF-α, IL-6, IL-1β), up-regulated anti-inflammatory factors (IL-10), enhanced barrier integrity, and restored microbial homeostasis. In murine models of IBD, MY-E@SS not only alleviated intestinal inflammation but also facilitated the recovery of extraintestinal organs, most notably respiratory metabolism. Omics analyses revealed that the mechanism involves inhibiting the TNF-α/NF-κB signaling pathway, suppressing pro-inflammatory genes, and modulating the arachidonic acid metabolism pathway. Mimicking the multi-tiered defense and repair strategies of corals, this biomimetic system enables targeted, self-regulated therapy and represents a translatable strategy for innovative and sustainable IBD management.

## Results

### Design and construction of engineered bacteria MY-EcN

**Fig. 2:**
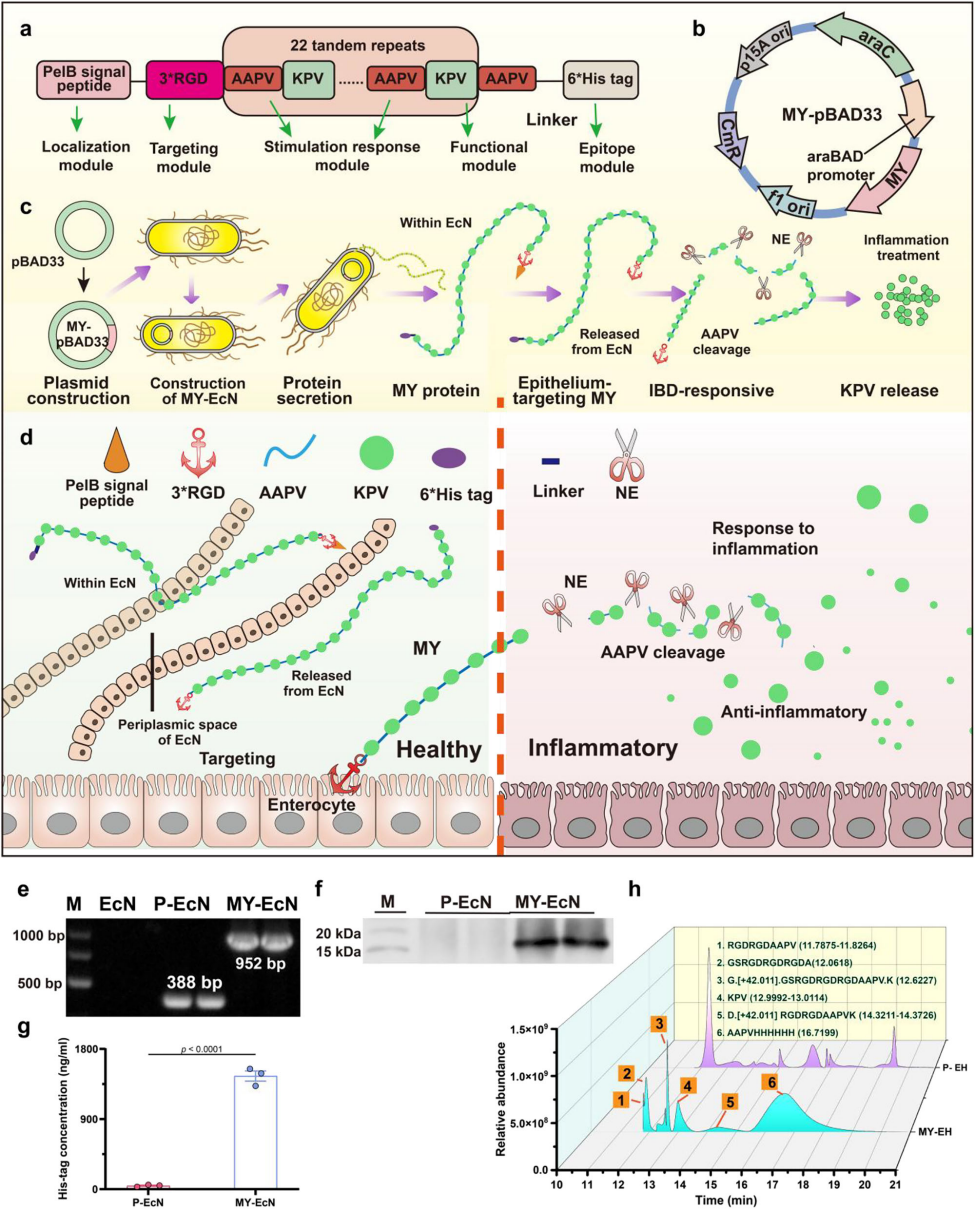
Design and construction of the engineered bacterial strain MY-EcN. **a** Modular design strategy of the MY protein expression system, illustrating its mechanism in the inflammation response and treatment. **b** Plasmid map of the recombinant vector MY-pBAD33. **c** Schematic of MY protein secretion by MY-EcN. **d** Mechanism of action of the MY protein against IBD. **e** Colony PCR verification of MY-EcN. The target amplicon size is 952 bp. M: DNA marker. **f** Western blot analysis of MY protein expression in LB medium. Representative from *n* = 3 biologically independent samples. **g** Concentration of His-tagged protein in the supernatant of P-EcN and MY-EcN after 8 h of culture. **h** LC-MS/MS analysis of MY protein responsiveness under inflammatory conditions. Representative from *n* = 3 biologically independent samples. Note: MY-EH / P-EH are the secreted proteins of MY-EcN or P-EcN, respectively, after NE digestion. Data are presented as the mean ± SEM (*n* = 3 biologically independent experiments). Statistical analysis was performed using one-way ANOVA with Tukey’s test (**g**). Source data are provided as a Source Data file. NE Neutrophil elastase; RGD Arg-Gly-Asp; KPV Lys-Pro-Val; AAPV Ala-Ala-Pro-Val; EcN *Escherichia coli* Nissle 1917.

Central to the bionic microcapsule reactor is constructing an engineered bacterial system that mimics the danger sensing-tissue repair cascade response process. The key step represented by the modification of *Escherichia coli* Nissle 1917 (EcN) is to design gene modules to precisely regulate gene expression and generate the required therapeutic products^19^. Accordingly, this work employed a modular strategy that combines tandem expression technology with synthetic biology methods to establish an expression system for the target protein MY. The expression system consists of 5 functional modules: localization (pelB), targeting (RGD), stimulus-responsive (AAPV), functional (KPV), and epitope tag (His-tag) (Fig. 2a). Following the construction of the recombinant plasmid MY-pBAD33 (Fig. 2b) and its transformation into EcN, the resulting engineering strain MY-EcN was capable of the secretory expression of MY (Fig. 2c). Within this expression system, the pelB signal peptide directs the localization protein to the periplasmic space, simplifying its acquisition. Featuring 22 AAPV-KPV repeat units, the expression system is capable of simultaneously releasing a high dose of the anti-inflammatory peptide KPV in the inflammatory microenvironment (Fig. 2d). Besides, the His-tag module enables efficient protein detection and purification. Critically, these modules function not in isolation, but as a synergistically integrated system.

With the recombinant vector confirmed (Fig. S1), the empty vector strain P-EcN was used as a negative control, and its supernatant proteins were collected and concentrated (named P). Following transformation, screening, and colony PCR verification, the successful expression of MY in the MY-EcN strain was confirmed by enzyme-linked immunosorbent assay (ELISA) and Western blot (Fig. 2e–g and S2). Liquid chromatography–tandem mass spectrometry (LC-MS/MS) analysis of NE-digested proteins P and MY revealed a substantial, specific release of KPV peptides only from MY (Table S1). In contrast, minimal KPV was detected in undigested MY or in protein P before and after digestion (Fig. 2h, S3). To further confirm the in vivo release and localization of KPV, we monitored its concentration in colonic contents over time. The levels peaked at 4 h post-administration (Fig. S4a), and specific accumulation in the colon was verified (Fig. S4b). Moreover, elevated MY protein levels in colonic contents of MY-E@SS treated mice (Fig. S5) corroborated effective in vivo expression and processing. The above results demonstrated that the engineered strain MY-EcN successfully expresses the MY protein, which is selectively cleaved in the intestinal inflammatory microenvironment to yield the anti-inflammatory peptide KPV.

### Preparation of MY-E@SS

**Fig. 3:**
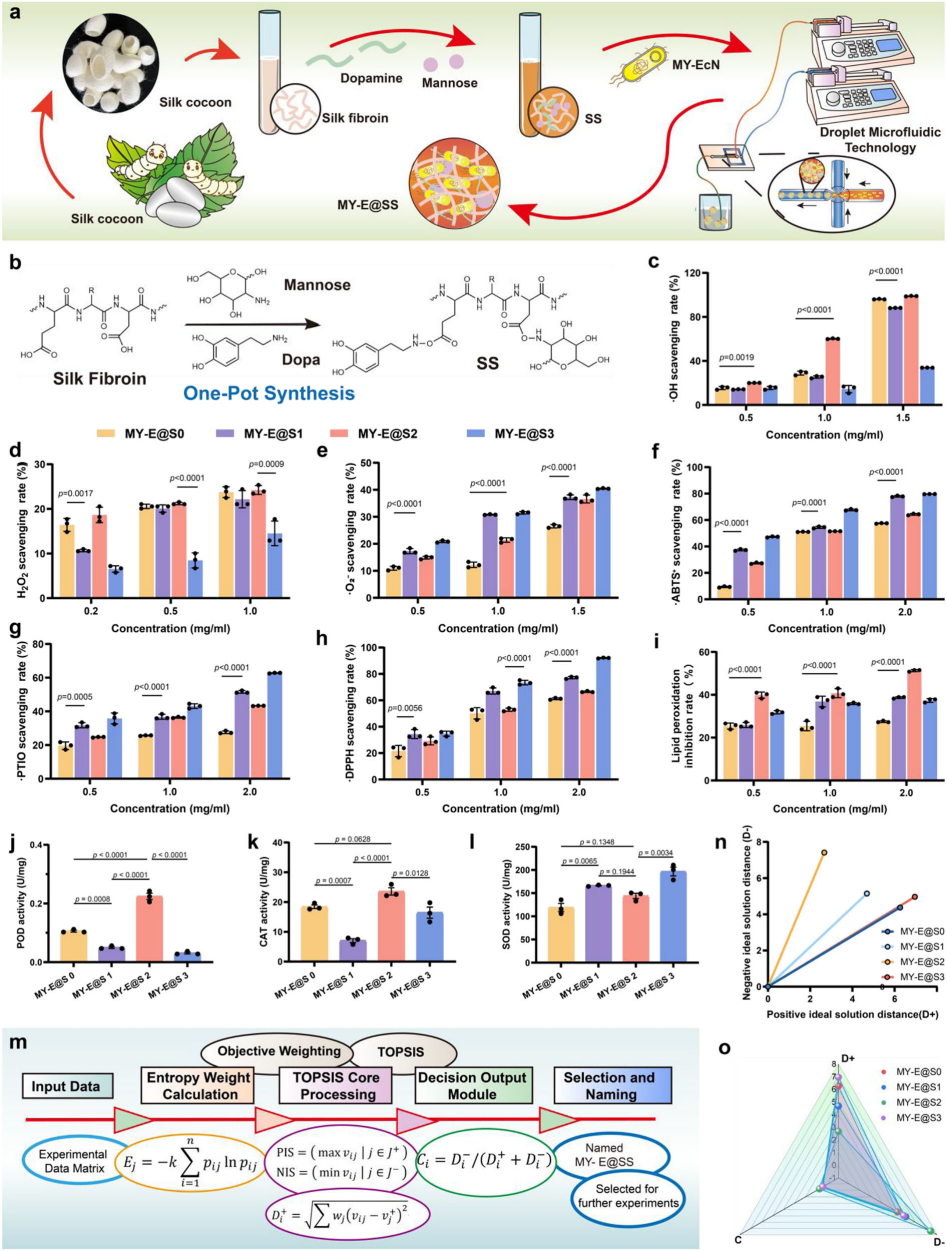
Preparation of MY-E@SS and evaluation of its antioxidant capacity. **a** Schematic of the MY-E@SS preparation process. **b** Chemical synthesis pathway of SS. **c**–**l** Scavenging capacity of four engineered bacterial microcapsules against RONS, including H₂O₂, ·OH, ·O₂⁻, ·ABTS⁺, ·PTIO, organic free radical (·DPPH) elimination, lipid peroxidation inhibition, and enzyme-like activities (POD, CAT, SOD). **m** Schematic workflow of the entropy weight-TOPSIS algorithm. **n** Calculated distances to positive and negative ideal solutions (D⁺ and D⁻) across different formulation ratios. **o** Comprehensive visualization of evaluation results via radar chart. Data are presented as the mean ± SEM (*n* = 3 biologically independent material batches). Statistical analysis was performed using one-way ANOVA with Tukey’s test (**c**–**l**). Source data are provided as a Source Data file. ABTS, 2,2’-Azinobis-(3-ethylbenzothiazoline-6-sulfonic acid); PTIO, 2-Phenyl-4,4,5,5-tetramethylimidazoline-1-oxyl-3-oxide; DPPH, 1,1-Diphenyl-2-picrylhydrazyl; POD Peroxidase; SOD Superoxide Dismutase; CAT Catalase; TOPSIS Technique for order preference by similarity to ideal solution.

To endow the engineered bacteria with external defensive capability and implement the complete “defense protection-danger sensing-tissue repair” cascade within a biomimetic microcapsule reactor, we designed and synthesized a shell based on the bionic strategy. This shell is intended to protect the reactor under harsh gastrointestinal conditions and synergistically promote colonic repair along with the engineered bacteria. Specifically, SF was extracted from natural silkworm cocoons (Fig. S6). Following carboxyl activation, it was conjugated with dopamine and mannose via Schiff base/Michael addition to form the biomimetic microcapsule shell (Fig. 3a and b). To explore the optimal shell for anti-inflammatory efficacy, three dopamine-to-mannose ratios (1:1, 1:2, and 2:1) were tested, yielding corresponding shells designated S1, S2, and S3. Using mixtures of the respective shell materials with the engineered bacteria MY-EcN as the stationary phase, we successfully fabricated uniform engineered bacterial microcapsules MY-E@S1, MY-E@S2, and MY-E@S3 via droplet microfluidic technology (Movie S1). Microcapsules encapsulated with pure SF (MY-E@S0) served as the control. Subsequently, the antioxidant properties of the four microcapsule types were evaluated through multiple assays (Fig. 3c–l and S7). MY-E@S2 demonstrated superior performance to both MY-E@S1 and MY-E@S3 in scavenging H₂O₂ and ·OH, inhibiting lipid peroxidation, and exhibiting POD-like and CAT-like activities. On the other hand, MY-E@S3 exhibited stronger scavenging ability toward ·PTIO and ·DPPH radicals, along with higher SOD-like activity. Remarkably, all three modified shells exhibited improved RONS scavenging and enzyme-mimetic activities compared to the pure SF shell.

After evaluating the anti-inflammatory potential of each microcapsule reactor, the entropy weight-TOPSIS method was applied to comprehensively assess the candidate systems and identify the optimal formulation (Fig. 3m). This approach minimizes the influence of subjective factors on the evaluation results by objectively determining weights based on the dispersion degree of each indicator^20,28^. Multiple performance indicators were used as inputs to the entropy weight method for calculating the weight of each index (Table S2). The positive (A+ ) and negative ideal solutions (A-) were then determined (Fig. S8a and Table S2), and the relative closeness (C) of each formulation to these ideal solutions was computed by incorporating the weights (Fig. 3n and S8b). Among them, MY-E@S2 demonstrated the closest proximity to the positive ideal solution (Fig. 3o and Table S4), and was consequently selected and renamed MY-E@SS for further experiments.

### Characterization and Evaluation of MY-E@SS

**Fig. 4:**
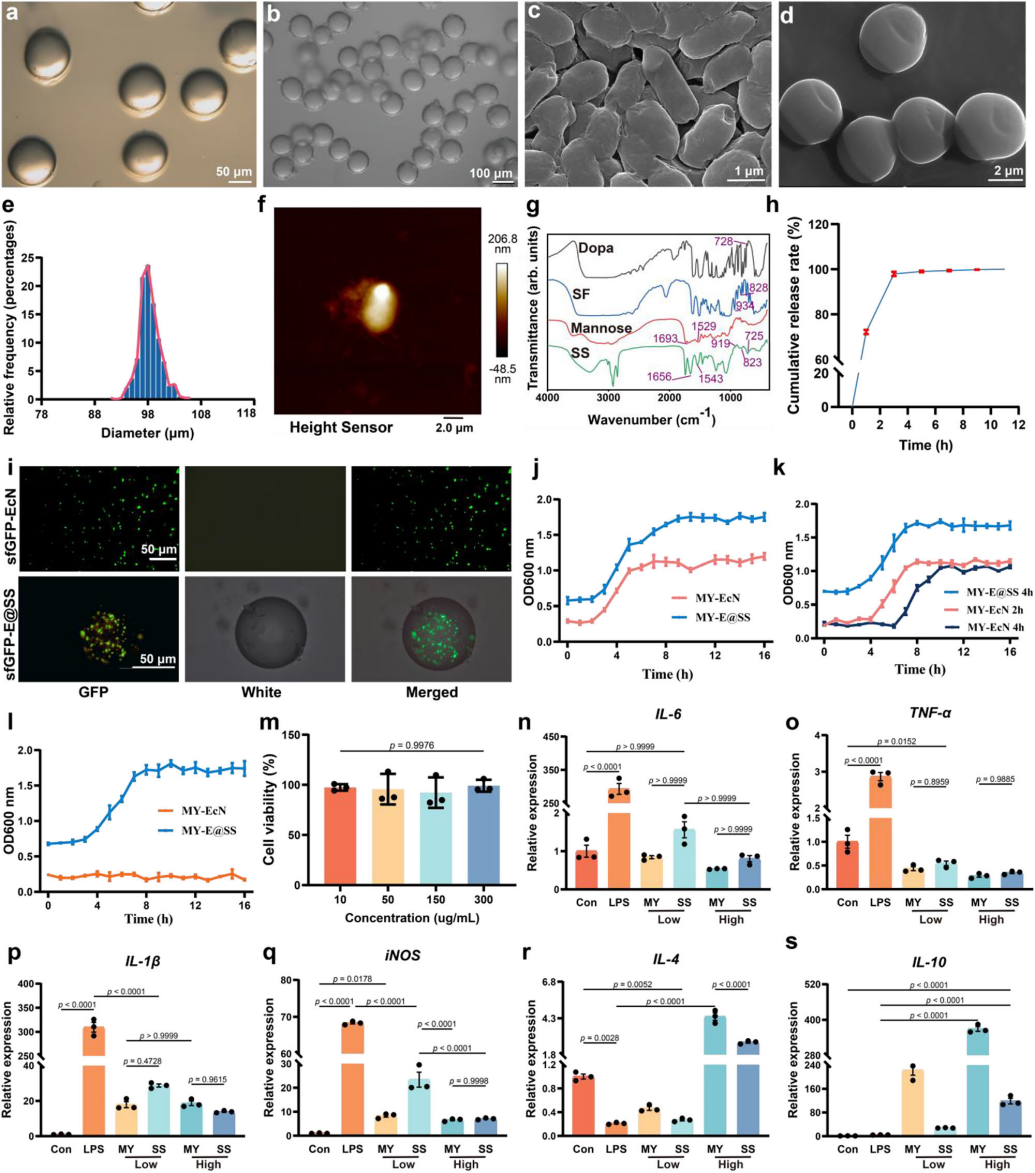
Characterization and evaluation of MY-E@SS. **a**, **b** Optical microscopy images of MY-E@SS before lyophilization. **c** SEM image of MY-EcN. (**d**) SEM image of lyophilized MY-E@SS. **e** Particle size distribution of MY-E@SS (before lyophilization). **f** Atomic force microscopy (AFM) topography of MY-E@SS. **g** FTIR spectra of SS and MY-E@SS. Characteristic peaks were observed at: 919 cm⁻¹ (β-pyranose ring of mannose), 823 cm⁻¹ (α-pyranose ring of mannose), and 725 cm⁻¹ (C-H bending of the dopamine benzene ring). The shifts of the amide I (1693 → 1656 cm⁻¹) and amide II (1529 → 1543 cm⁻¹) bands indicate the formation of amide bonds among SF, mannose, and dopamine. **h** Cumulative release of vitamin-B12@SS. **i** Confocal laser scanning microscopy (CLSM) images of sfGFP-EcN and sfGFP-E@SS. **j** Bacterial growth curves under standard culture conditions. **k** Growth profiles after 1.5 h of simulated gastric digestion. **l** Growth profiles after 2 h of simulated intestinal digestion. **m** Cell viability after treatment with varying doses. Data are normalized to untreated controls (set as 100% viability). **n**–**s** qRT-PCR analysis of inflammatory cytokine gene expression (*IL-6*, *TNF-α*, *IL-1β*, *iNOS*, *IL-4*, *IL-10*) in RAW264.7. Cells were treated with MY‑EH (4 and 8 ng/mL) and SS (150 and 300 μg/mL). Low denotes 4 ng/mL MY‑EH and 150 μg/mL SS; High denotes 8 ng/mL MY‑EH and 300 μg/mL SS. **a**–**d**, **f**, **g** and **i** show representative images from *n* = 3 independent experiments. All quantitative data (panels j-s) are presented as the mean ± SEM from *n* = 3 biologically independent samples. Statistical analysis for panels **h**, **j**–**l**, **m**, and **n**–**s** was performed using one-way ANOVA with Tukey’s test. Source data are provided as a Source Data file.

To systematically evaluate the physicochemical properties of the MY-E@SS microcapsule reactor, comprehensive characterization was performed, focusing on its microscopic morphology, structure, and release behavior. Uniform spherical microcapsules with well-defined morphology were prepared using droplet microfluidics (Fig. 4a, b). Both SF and SS formed stable hydrogels (Fig. S9a, b). After freeze-drying, MY-E@SS obtained an off-white powdered form (Fig. S9c). Scanning electron microscopy (SEM) images revealed the distinct surface morphology and microstructure of lyophilized MY-EcN and MY-E@SS (Fig. 4c, d). The hydrated microcapsules exhibited an average diameter of ~98 μm (Fig. 4e), while the dried particles measured about 4 μm (Fig. 4d, f). Such a particle size distribution reduces the gritty sensation, enhancing patient compliance during oral intake. Fourier transform infrared (FTIR) spectroscopy confirmed successful chemical bonding among SF, dopamine, and mannose (Fig. 4g). Given the high encapsulation efficiency of SS (Fig. S10), the sustained-release behavior of the SS shell was assessed using vitamin B12 as a model molecule for bacterial metabolites, based on a previous method^29^. As shown in Fig. 4h, approximately 80% of the encapsulated vitamin B12 was released within 1 h, with complete release achieved within 3 h. Such rapid release kinetics facilitates the prompt attainment of effective therapeutic concentrations at the target site.

Achieving sufficient bacterial density is paramount for developing effective oral probiotic formulations. To evaluate the bacterial immobilization capacity of SS, we encapsulated fluorescently labeled bacteria (sfGFP-EcN, Fig. S11) into SS-based microcapsules (named sfGFP-E@SS). SS traps bacteria in confined compartments, leading to an increase in local bacterial density (Fig. 4i). After 12 h in tissue-mimicking fluid at 37 °C, the SS shell protected the encapsulated engineered bacteria and their progeny by preventing bacterial leakage (Fig. S12). Moreover, the microcapsule reactor could be lyophilized and stored at room temperature for at least two months without losing bacterial viability. The recovered sfGFP-EcN resumed full functionality after resuscitation in LB medium (Fig. S13), demonstrating its potential for long-term storage.

Further evaluation was performed to assess the effect of SS encapsulation on the biological function of the probiotic. While both strains grew similarly under standard conditions (Fig. 4j), MY-E@SS proved vastly superior in simulated gastrointestinal environments. Exposure to simulated gastric fluid (SGF) almost entirely inactivated the unencapsulated MY-EcN (Fig. 4k), and simulated intestinal fluid (SIF) markedly inhibited its growth (Fig. 4l). In contrast, MY-E@SS retained full growth capacity after both treatments. Collectively, the SS coating effectively shields the probiotic from harsh gastrointestinal conditions, ensuring significantly improved survival and proliferative potential.

To evaluate the biocompatibility and antioxidant activity of MY-E@SS, we conducted in vitro assessments of cytotoxicity and ROS scavenging capacity. Co-culture with RAW 264.7 cells showed no significant toxicity (Fig. 4m). Furthermore, in an LPS-induced macrophage inflammatory model, both MY and SS components scavenged ROS (Fig. S14). At the mRNA level, they downregulated pro-inflammatory cytokines (*IL-6*, *TNF-α*, *IL-1β*, and *iNOS*; Fig. 4n-q) and upregulated anti-inflammatory factors (*IL-10* and *IL-4*; Fig. 4r-s). The above results confirm that MY-E@SS is biocompatible, antioxidative, and anti-inflammatory.

### Therapeutic efficacy of MY-E@SS for IBD

**Fig. 5:**
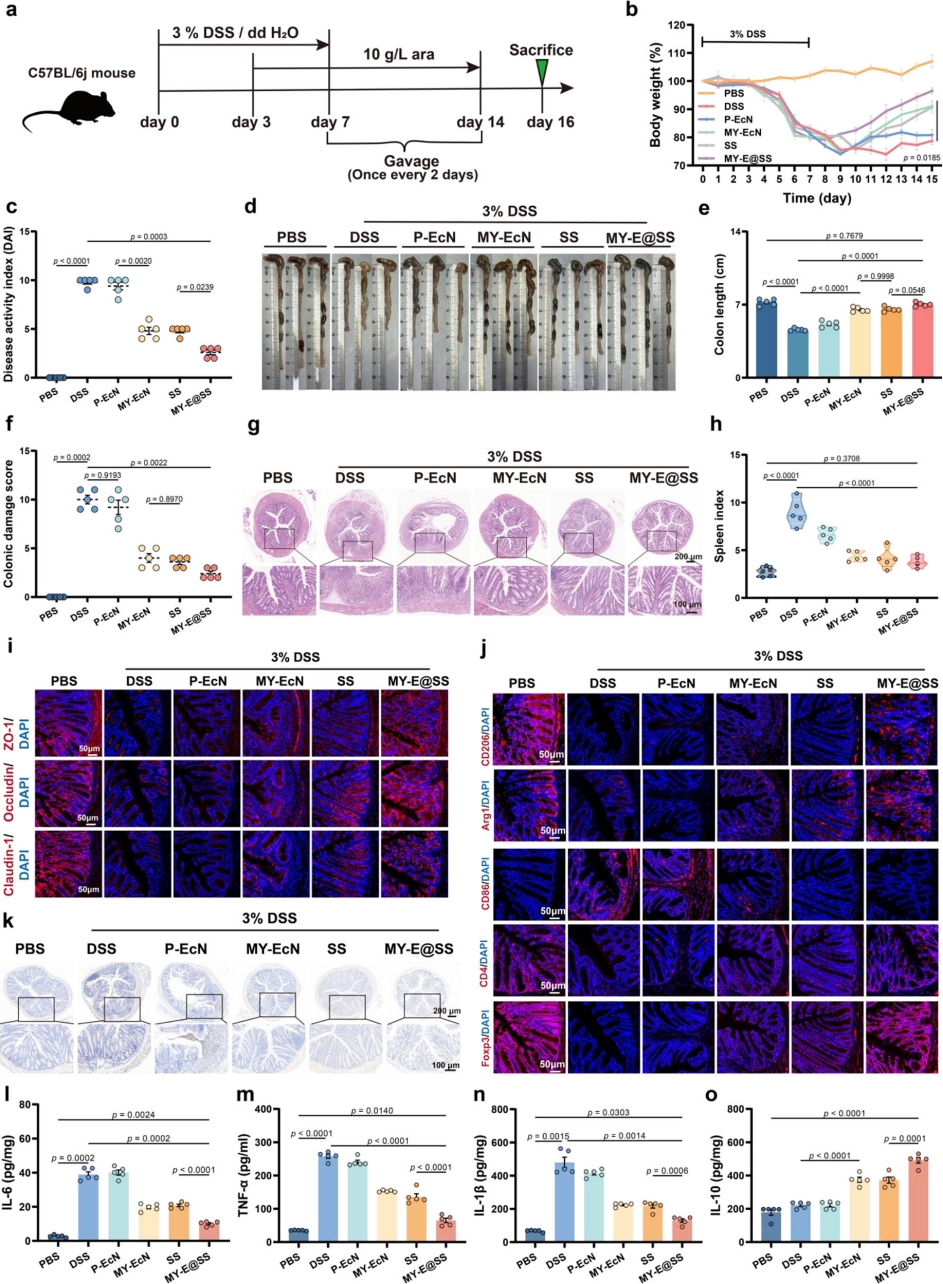
Therapeutic efficacy of MY-E@SS in a DSS-induced murine IBD model. **a** Experimental timeline. C57BL/6 mice were administered 3% DSS in drinking water from day 0 to day 7. From day 7 to day 14, mice received daily oral gavage of PBS, P-ECN, MY-ECN, SS, or MY-E@SS (1 × 10⁸ CFU). **b** Body weight changes and (**c**) disease activity index (DAI) throughout the experimental period. **d** Representative colon images. **e** Colon lengths. **f** Colonic histopathological scores. **g** Representative hematoxylin and eosin (H&E)-stained sections of colon tissues. **h** Spleen index (spleen weight/body weight). **i** Immunofluorescence staining of key tight junction proteins in transverse colon sections. **j** Immunofluorescence analysis reveals MY-E@SS modulates the local colonic immune microenvironment. **k** Myeloperoxidase (MPO) staining in colon sections. **l**–**o** Cytokine levels (IL-6, TNF-α, IL-1β, IL-10) in colon tissues measured by ELISA. All samples were collected on day 16. Data are presented as the mean ± SEM. Statistical analysis was performed using an unpaired two‑tailed Student’s t‑test for (**b**); one-way ANOVA with Tukey’s test (**c**, **e**, **f**, **h**, **l**–**o**). For all panels, *n* = 5 biologically independent animals per group. Representative images from *n* = 5 biologically independent experiments. Source data are provided as a Source Data file. DSS Dextran sulfate sodium.

Given its favorable gastrointestinal viability, RONS-scavenging capacity, and in vitro anti-inflammatory activity, the therapeutic effect of MY-E@SS was further evaluated in a dextran sulfate sodium (DSS)-induced IBD mouse model. The experimental scheme is depicted in Fig. 5a. Mice with IBD exhibited progressive weight loss, which was significantly ameliorated by oral gavage of MY-E@SS (Fig. 5b). Concurrently, fecal occult blood tests showed substantial improvement in the MY-E@SS-treated group approximately by day 10 (Fig. S15). Consistent with expectations, the MY-E@SS group exhibited a markedly reduced disease activity index (DAI) on day 15 relative to the DSS control and other treatments (Fig. 5c, and Table S5). To facilitate evaluation, the colon and internal organs of the mice were harvested on day 16 of the experiment. In comparison to the other treatment groups, the colon length in the MY-E@SS group was significantly extended (Fig. 5d, e). The spleen, as a primary immune response organ^29^, exhibited significant recovery in size and weight in the MY-EcN, SS, and MY-E@SS groups compared to the DSS group (Fig. 5f, and S16). Most notably, colon tissue morphology closely resembled the normal state, with well-preserved architecture, intact crypts, and markedly reduced inflammation (Fig. 5g, h, and Table S6), demonstrating effective repair of DSS-induced damage. Immunofluorescence analysis confirmed that MY-E@SS significantly restored the expression and distribution of key tight junction proteins (ZO-1, Occludin, and Claudin-1) in the colonic epithelium compared to the disease model (Fig. 5i, and S17), supporting enhanced mucosal barrier integrity. While MY-EcN or SS alone showed limited efficacy in intestinal repair, MY-E@SS promoted macrophage polarization from a pro-inflammatory (CD86^+^) to an anti-inflammatory/reparative (CD206^+^/Arg1^+^) phenotype and increased Foxp3^+^CD4^+^ regulatory T cell infiltration, collectively rebalancing the local immune environment (Fig. 5j, S18 and S19). Furthermore, all groups showed no significant differences in systemic organ indices or hematological parameters compared to the PBS control, indicating no obvious systemic toxicity and confirming favorable biosafety (Fig. S20 and S21). Immunofluorescence staining for NE further demonstrated a significant increase in neutrophil infiltration at DSS-induced IBD (Fig. S22), which is consistent with the observed reduction in myeloperoxidase (MPO) activity (Fig. 5k) and corroborates the in vivo activation conditions for the engineered bacteria to sense and respond to this microenvironment. Excessive inflammation in IBD is primarily driven by increased pro-inflammatory cytokines in intestinal cells^30^. Accordingly, we quantified key pro-inflammatory cytokines (IL-1β, TNF-α, IL-6) in colonic tissues via ELISA. As shown in Fig. 5l–n, MY-E@SS treatment significantly suppressed the expression of these cytokines. Conversely, the level of the anti-inflammatory cytokine IL-10, which plays a critical role in mitigating inflammation and promoting mucosal healing, was markedly elevated in the MY-E@SS group (Fig. 5o). Furthermore, MY-E@SS treatment systematically alleviated systemic oxidative stress in IBD mice, as indicated by increased serum levels of the antioxidant markers superoxide dismutase (SOD) and glutathione (GSH), and a decreased level of the lipid peroxidation product malondialdehyde (MDA) (Fig. S23). Importantly, a consistently potent therapeutic efficacy of MY-E@SS was also observed in a complementary (2,4,6-Trinitrobenzenesulfonic acid) TNBS-induced colitis model (Fig. S24), further substantiating its robust and reliable anti-inflammatory and mucosal healing properties across diverse experimental IBD settings. Together, these observations demonstrate the broad protective effect of MY-E@SS against IBD, evidenced by marked amelioration of its key pathological, histological, and immunological hallmarks.

### SS Shell Prolongs the Colonization of Probiotics in the Inflamed colon

**Fig. 6:**
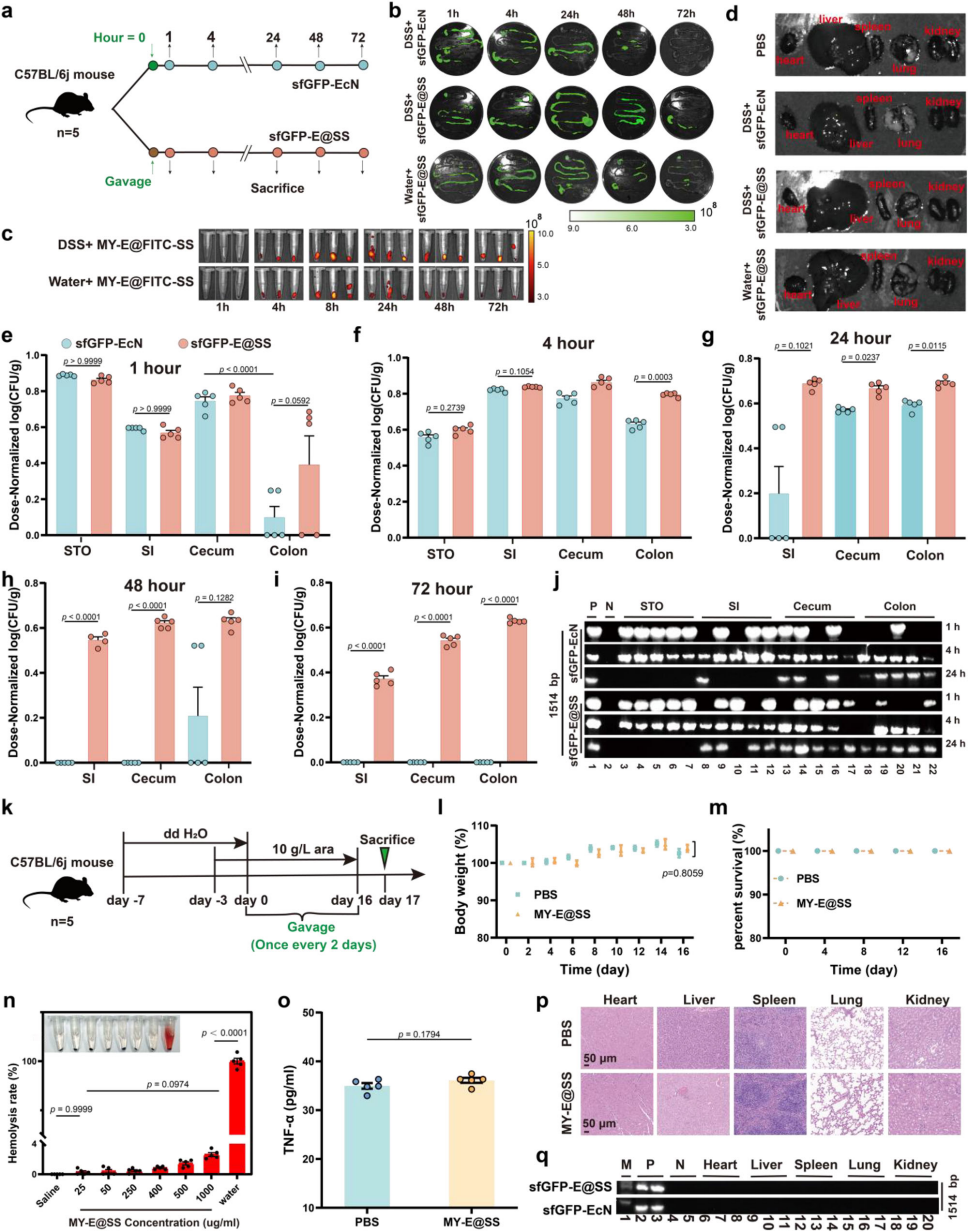
MY-E@SS prolongs the colonization of probiotics and has good biocompatibility. **a** Scheme of the experimental procedure for evaluating probiotic colonization. DSS-induced IBD mice were orally gavaged with 1 × 10⁸ CFU of sfGFP-EcN or sfGFP-E@SS. Intestinal colonization was assessed at the indicated time points (1, 4, 24, 48, and 72 h) by measuring sfGFP-derived bioluminescence in the colon using an in vivo imaging system. **b**, **c** In vivo fluorescence tracking of probiotics: **b** Representative fluorescence images. **c** Assessment of gastrointestinal transit time by monitoring fecal FITC-SS excretion levels. **d** Distribution of sfGFP-EcN in major organs at 4 h after oral gavage. **e**–**i** Kinetic profiles of colonizing EcN in different gastrointestinal segments (stomach, small intestine, cecum, colon) at 1, 4, 24, 48, and 72 h post-administration. **j** Strain‑specific PCR analysis of tissue homogenates showing the temporal and spatial distribution of sfGFP-EcN/sfGFP-E@SS. P positive control; N negative control; STO stomach; SI: small intestine. The specific amplicon is 1514 bp. All samples were derived from the same experiment and the gels/blots were processed in parallel. **k** Schematic of the experimental design for biocompatibility evaluation. **l** Body weight changes of mice with or without oral administration of MY-E@SS. **m** Survival rates of the two groups during the observation period. Both groups exhibited 100% survival. **n** Concentration-dependent hemolytic activity of MY-E@SS on erythrocytes in vitro. **o** Quantitative analysis of TNF-α levels in colon tissues at day 17 post-treatment. **p** H&E staining of major organs at day 17. **q** PCR‑based detection of bacterial translocation in visceral organs. The specific amplicon is 1514 bp. Lanes: M DNA marker; N negative control; P positive control. All samples were derived from the same experiment and the gels/blots were processed in parallel. Data are presented as mean ± SEM. For (**e**–**i**, **l**, **n**, **o**), *n* = 5 biologically independent animals per group. For (**d**, **j**, **p**), representative results from n = 5 biologically independent experiments are shown. For (**b**, **c**, **q**), representative results from *n* = 3 biologically independent experiments are shown. Statistical analysis was performed using one‑way ANOVA with Tukey’s post hoc test for (**e**–**i**); an unpaired two‑tailed Student’s t‑test for (**l**) and (**o**). Source data are provided as a Source Data file. DSS dextran sulfate sodium; sfGFP superfolder green fluorescent protein; EcN *Escherichia coli* Nissle 1917; FITC fluorescein isothiocyanate.

Encouraged by these positive results, we investigated whether the bionic shell could enhance the intestinal persistence of probiotics in a mouse model of IBD. Mice were administered either free or encapsulated probiotics, and colonization in the colon was monitored over 72 h using bioluminescence imaging (Fig. 6a). Encapsulation with the SS shell led to a significantly higher viable bacterial count compared to the non-encapsulated sfGFP-EcN group (Fig. 6b, and S25). Notably, at the 72h time point, IBD mice showed a significantly stronger colonic signal than healthy controls after receiving sfGFP-E@SS. Similarly, compared with healthy control mice, IBD mice treated with FITC-labeled SS shell microcapsules (MY-E@FITC-SS) displayed enhanced intestinal fluorescence (Fig. 6c), collectively indicating the specific accumulation and prolonged colonization of the SS encapsulated probiotics at inflammatory sites. Accordingly, we infer that the efficacy of the SS shell critically depends on pathophysiological factors unique to the inflammatory intestinal microenvironment. These factors are absent in healthy controls, leading to reduced colonization under normal conditions. Furthermore, the absence of significant bacterial byproduct leakage in major organs (Fig. 6d) confirms the ability of SS to enhance colonization without systemic spread. To precisely delineate the in vivo behavior of the microcapsules, we performed dual-fluorescence labeling of the SS shell (Cy5) and the engineered bacteria (sfGFP) for spatiotemporal tracking. Imaging over 72 hours revealed high colocalization of both signals in the colon during the first 8 hours, visually confirming effective protection and co-transport by the shell through the upper GI tract (Fig. S26). The shell signal peaked at 8-12 hours and subsequently decayed, indicating its gradual degradation. Concurrently, bacterial fluorescence in the colon increased markedly and persisted beyond 48 h, quantitatively confirming effective capsule disintegration followed by bacterial release, proliferation, and sustained colonization in situ. We further employed the plate counting method to quantitatively assess the colonization dynamics of the engineered bacteria in various segments of the gastrointestinal tract (Fig. 6a). Tissue samples from the stomach, small intestine, cecum, and colon were collected at designated intervals following the administration of sfGFP-EcN or sfGFP-E@SS, and bacterial loads were quantified as CFUs (Fig. 6e-i). Consistent with our above results, the bionic shell significantly delayed the clearance process of the bacteria. To confirm the identity of the colonized strain and avoid interference from environmental microorganisms, we performed PCR on tissue and fecal samples collected at various time points (Fig. 6j and S27). The stronger and more persistent signals in the sfGFP-E@SS group further molecularly confirmed the efficacy of the SS coating in promoting sustained probiotic colonization.

Collectively, the combination of viable bacterial counting, molecular detection, and fluorescence tracing across various time points confirms that the SS shell significantly enhances the gastrointestinal stability of the probiotics, facilitating their prolonged retention and enrichment at inflammatory sites.

### Biocompatibility evaluation of MY-E@SS

Given the promising therapeutic efficacy of MY-E@SS in a murine IBD model, its systemic biosafety was rigorously assessed (Fig. 6k). Over the treatment period, all mice survived, and their body weights remained stable (Fig. 6l, m). Blood analysis showed that all hematological parameters remained within normal physiological ranges (Fig. S28). The lack of a significant increase in hemolysis with MY-E@SS compared to the PBS group in vitro further indicates its hemocompatibility (Fig. 6n). Moreover, quantification of inflammatory cytokines (TNF-α, IL-6, IL-1β) and the anti-inflammatory cytokine IL-10 in colon tissue revealed no significant differences between the MY-E@SS and PBS groups (Fig. 6o, S29), indicating an absence of local immune activation or tissue toxicity under healthy conditions. Histological assessment of major organs from MY-E@SS treated mice showed normal morphology and no pathological changes on H&E staining (Fig. 6p), indicating no adverse organ effects. Additionally, sfGFP signal was absent from visceral organs (Fig. 6d) and homogenates (Fig. 6q, S30) following gavage with either sfGFP-EcN or sfGFP-E@SS, demonstrating that the bacteria did not translocate to peripheral sites.

Encouraged by these findings, we proceeded to conduct a 29-day in vivo biocompatibility assessment. Throughout the study, no abnormalities were observed in body weight, survival rate, or inflammatory cytokine levels in the mice (Fig. S31). Serum analysis after the experiment revealed no significant increase in IgE antibodies in the MY‑E@SS group, and the level of the allergy‑related chemokine MCP‑1 remained comparable to that of the PBS control group. Moreover, no allergy‑associated behaviors such as scratching or dyspnea were observed during the entire experimental period (Fig. S32). Additionally, the splenic index showed no significant difference compared with that of normal mice. Collectively, these results indicate a low risk of immediate‑type hypersensitivity reaction induced by MY‑E@SS and demonstrate an absence of apparent immunotoxicity or systemic adverse effects.

Different from the safety evaluation based on a single indicator, we adopted a multi-dimensional strategy to systematically assess various parameters-including body weight, survival rate, blood indices, hemolytic activity, inflammatory factors, and histopathology-thereby ensuring a comprehensive and reliable safety profile. Moreover, MY-E@SS exhibited a pronounced ability to avoid systemic dissemination, underscoring its potential as delivery platform. The collective evidence from these observations offers not only confirmation of the high biocompatibility of MY-E@SS but also a safety basis for advancing bionic systems as a therapeutic strategy for IBD.

### MY-E@SS ameliorates respiratory metabolism in mice with IBD

**Fig. 7:**
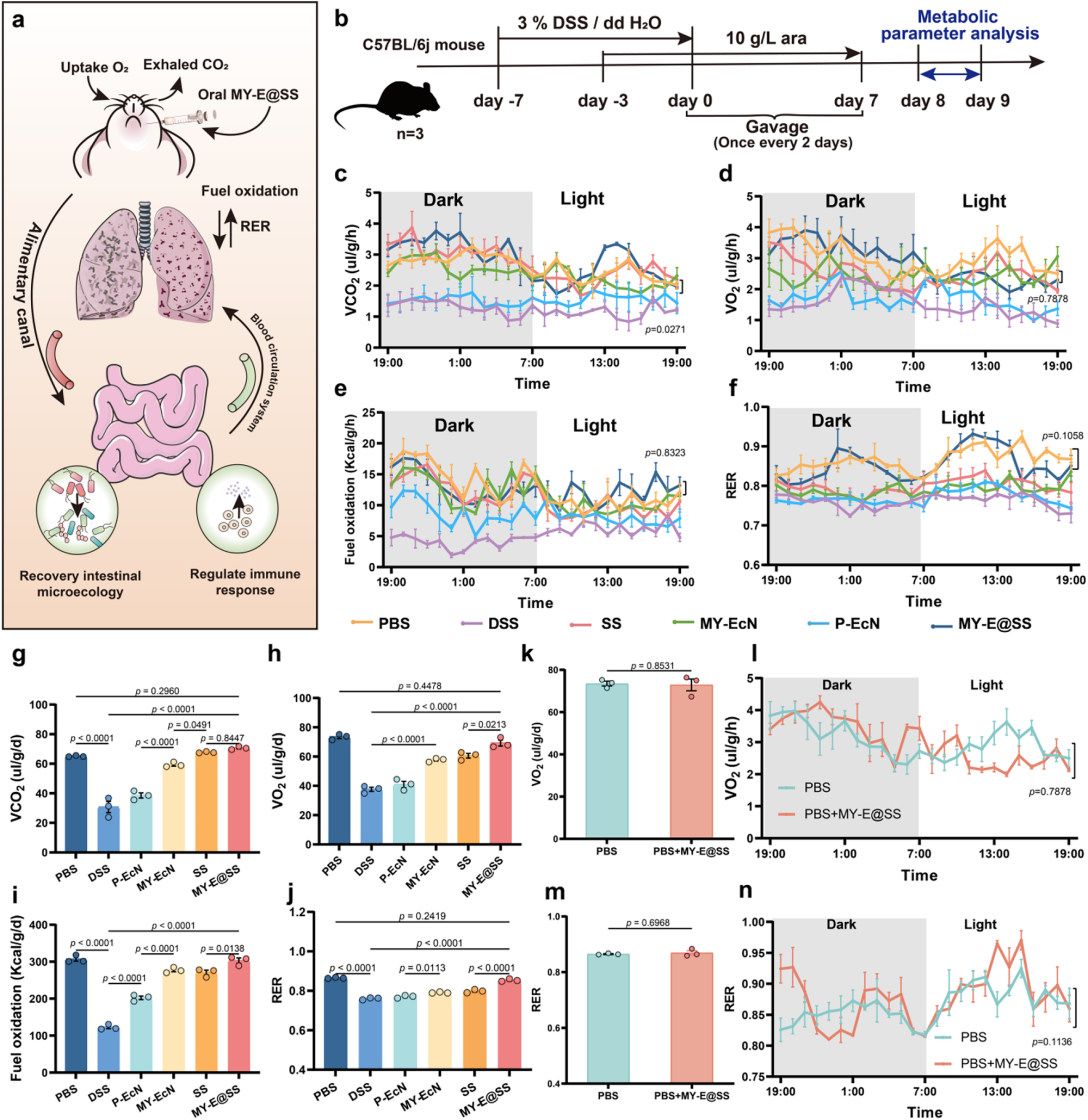
MY-E@SS restores respiratory metabolism in a murine model of IBD. **a** Schematic illustration of altered respiratory metabolism in mice with acute inflammation. **b** Experimental design for the analysis of metabolic parameters. **c**–**f** Dynamic changes in VCO₂, VO₂, fuel oxidation rate, and the RER in each group on day 7 post-treatment. **g**–**j** Total 24-hour cumulative VCO₂, VO₂, fuel oxidation, and RER in each group on day 7 post-treatment. **k**, **l** Total 24-h oxygen consumption (**k**) and dynamic VO₂ curves (**l**) in healthy control mice following 16 days of MY-E@SS gavage. **m**, **n** Total 24-h cumulative RER (**m**) and dynamic RER curves (**n**) in healthy control mice after 16 days of gavage. Data are presented as the mean ± SEM (**c**–**n**). For (**g**–**j**), statistical analysis was performed using one‑way ANOVA with Tukey’s post hoc test. For (**c**–**f**, **k**, **l**, **m**, **n**), comparisons between groups were performed using unpaired Student’s t-test. All data (**c**–**n**) represent *n* = 3 biologically independent experiments. Source data are provided in the Source Data file. RER respiratory exchange ratio; DSS Dextran sulfate sodium.

Respiratory metabolism provides a critical indicator of systemic energy homeostasis and overall physiological status (Fig. 7a). However, the alterations in respiratory metabolism during IBD and post-therapy recovery remain incompletely understood^31^. Using metabolic cage monitoring (Fig. 7b), we observed that DSS-induced IBD caused significant respiratory metabolic dysfunction in mice, marked by pronounced reductions in oxygen consumption (VO₂), carbon dioxide production (VCO₂), the respiratory exchange ratio (RER), and fuel oxidation (Fig. 7c-f). These changes reflect IBD severity from an energy metabolism perspective, consistent with recent study^31^. After intervention, the MY-EcN, SS, and MY-E@SS groups exhibited substantial recovery, characterized by restored respiratory metabolic parameters, stabilized circadian rhythms, and enhanced fuel utilization (Fig. 7g-j and S33). Notably, the MY-E@SS group showed superior recovery across multiple metrics, outperforming each component alone, indicating a synergistic effect between MY-EcN and SS in promoting energy metabolism recovery.

Although some recent studies have begun to explore metabolic reprogramming in the context of IBD treatment, respiratory metabolism–a non-invasive and holistic indicator, remains underrepresented in therapeutic evaluation frameworks^32,33^. Therefore, we not only validated the sensitivity of respiratory metabolic parameters as markers of treatment efficacy but also systematically assessed the biosafety profile of MY-E@SS. In healthy mice, MY-E@SS had no significant effect on RER, energy expenditure, VO₂, or VCO₂ (Fig. 7k–n and S34), suggesting that respiratory function and basal metabolic homeostasis remained uncompromised. Together with its efficacy in alleviating colonic inflammation, this favorable biosafety profile underscores its promising potential for clinical translation.

### MY-E@SS regulated colonic microbiota in IBD mice

**Fig. 8:**
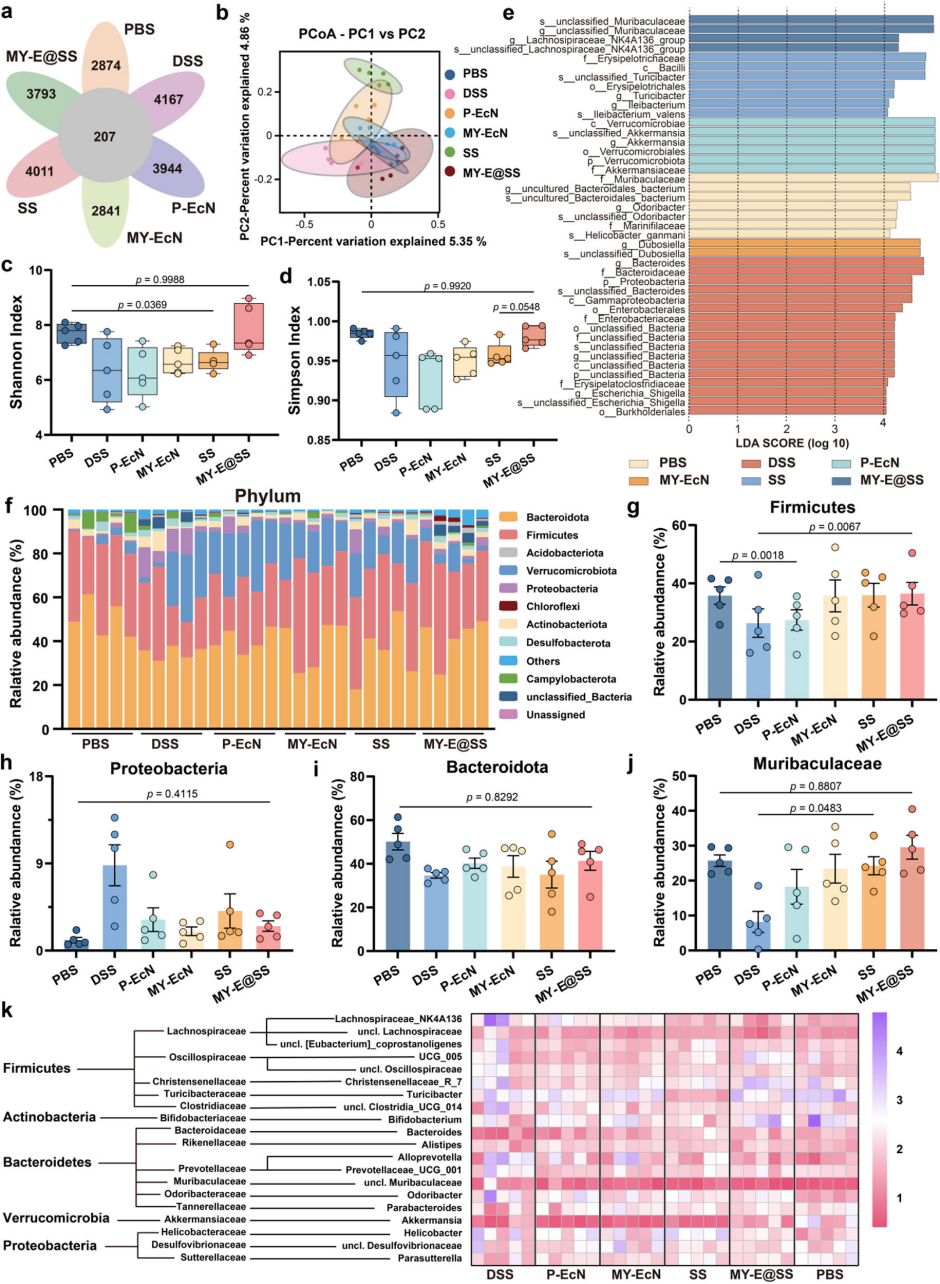
MY-E@SS alleviates colonic microbiota dysbiosis in IBD mice. **a** Venn diagram illustrating the overlap of operational taxonomic units (OTUs) across different treatment groups. **b** β-diversity analysis visualized using principal coordinate analysis (PCoA). **c**, **d** α-diversity indices assessed by the Shannon (**c**) and Simpson (**d**) indices. In the box plots, the center line denotes the median; the bounds of the box represent the 25th and 75th percentiles; the whiskers extend to the minimum and maximum values. **e** Distribution of linear discriminant analysis (LDA) scores. **f** Stacked bar plot showing the relative abundance of the top 12 bacterial taxa at the phylum level. **g**–**j** Relative abundances of key microbial taxa: Firmicutes (**g**), Proteobacteria (**h**), Bacteroidota (**i**), and Muribaculaceae (**j**). **k** Heatmap displaying differentially abundant taxa at the family level. Data are presented as the mean ± SEM. Statistical analysis were performed using one‑way ANOVA with Tukey’s test (**g**–**j**). (**a**–**k**) *n*  =  5 biologically independent experiments. Source data are provided as a Source Data file.

In order to investigate the regulatory role of MY-E@SS in the colonic microbiota of IBD mice, we characterized the microbial communities in mouse colonic contents using 16S rRNA gene sequencing. From 30 mice distributed across six groups, 2,400,056 high-quality 16S rRNA sequences were obtained. Sequences were resampled to an even depth and clustered into operational taxonomic units (OTUs) at 97% similarity. Comparative analysis revealed a significant microbial dysbiosis in the DSS-induced model relative to the control, characterized by 4,167 unique OTUs (Fig. 8a). In this work, we measured α-diversity using the Shannon and Simpson indices. Principal coordinate analysis (PCoA) showed that MY-E@SS administration induced a substantial shift in the colonic microbiota structure of IBD mice (Fig. 8b). While individual treatments of MY-EcN and SS enhanced microbial richness, the MY-E@SS group showed significantly higher Shannon and Simpson indices than the DSS group and all other treatment groups (Fig. 8c, d). Conversely, the P-EcN group exhibited values comparable to the DSS group, indicating a minimal effect on restoring microbial diversity in the compromised gut.

To identify differentially abundant taxa across groups, a Linear discriminant analysis effect size (LEfSe) analysis was performed. This analysis revealed key discriminant microbial taxa from the phylum to genus level (Fig. 8e). Based on LDA scores, biomarker taxa from the phylum to genus level were identified (Fig. 8e). It is noteworthy that four dominant bacterial groups in the MY-E@SS group were characterized as probiotics. Among these, Lachnospiraceae was particularly highlighted for its potential to maintain intestinal health via short-chain fatty acid production and immune response modulation^34^. Microbial composition at the phylum and genus/species levels was further visualized using bar plots and heatmaps, respectively (Fig. 8f and S35). Across all samples, 43 phyla were identified. At the phylum level, the MY-EcN, SS, and MY-E@SS treatment groups exhibited microbial composition patterns comparable to the PBS group (Fig. 8f). In the DSS group, the abundance of Firmicutes was significantly reduced, while that of Proteobacteria was markedly increased (Fig. 8g-i). In contrast, MY-EcN, SS, and MY-E@SS treatments enhanced the relative abundance of Firmicutes and effectively suppressed the abnormal proliferation of Proteobacteria. A bacterial family known for its probiotic properties, Muribaculaceae, showed higher abundance in the MY-E@SS group (Fig. 8j, k). Its beneficial effects may arise from competitive inhibition of pathogenic microorganisms and regulation of the intestinal immune microenvironment^35^. Moreover, MY-E@SS specifically modulated several bacterial families, including Akkermansiaceae, Bacteroidaceae, Bifidobacteriaceae, Oscillospiraceae, and Prevotellaceae (Fig. 8k). To further elucidate the dynamics of the microbial community, samples collected at multiple time points during the intervention were analyzed. Analyses of β-diversity (Fig. S36a) and α-diversity (Fig. S36b, c), along with the compositional structure of the microbiota at the phylum, genus, and species levels (Fig. S37), revealed that the community structure of the MY-E@SS treatment group gradually shifted toward that of the PBS group over time, visually demonstrating the restoration of the microbiota toward homeostasis. Concurrently, the abundance of several typical probiotic bacteria in the MY-E@SS group showed an increasing trend post-intervention (Fig. S38a, b) and approached the level of the control group by the end of the experiment, whereas typical harmful bacteria exhibited an opposite trend (Fig. S38c). Thus, MY-E@SS positively contributes to disease alleviation by restoring colonic microbial homeostasis, which is characterized by the reversal of diversity loss and structural disorder.

### MY-E@SS recovered gene expression pattern from inflammation to normalcy

**Fig. 9:**
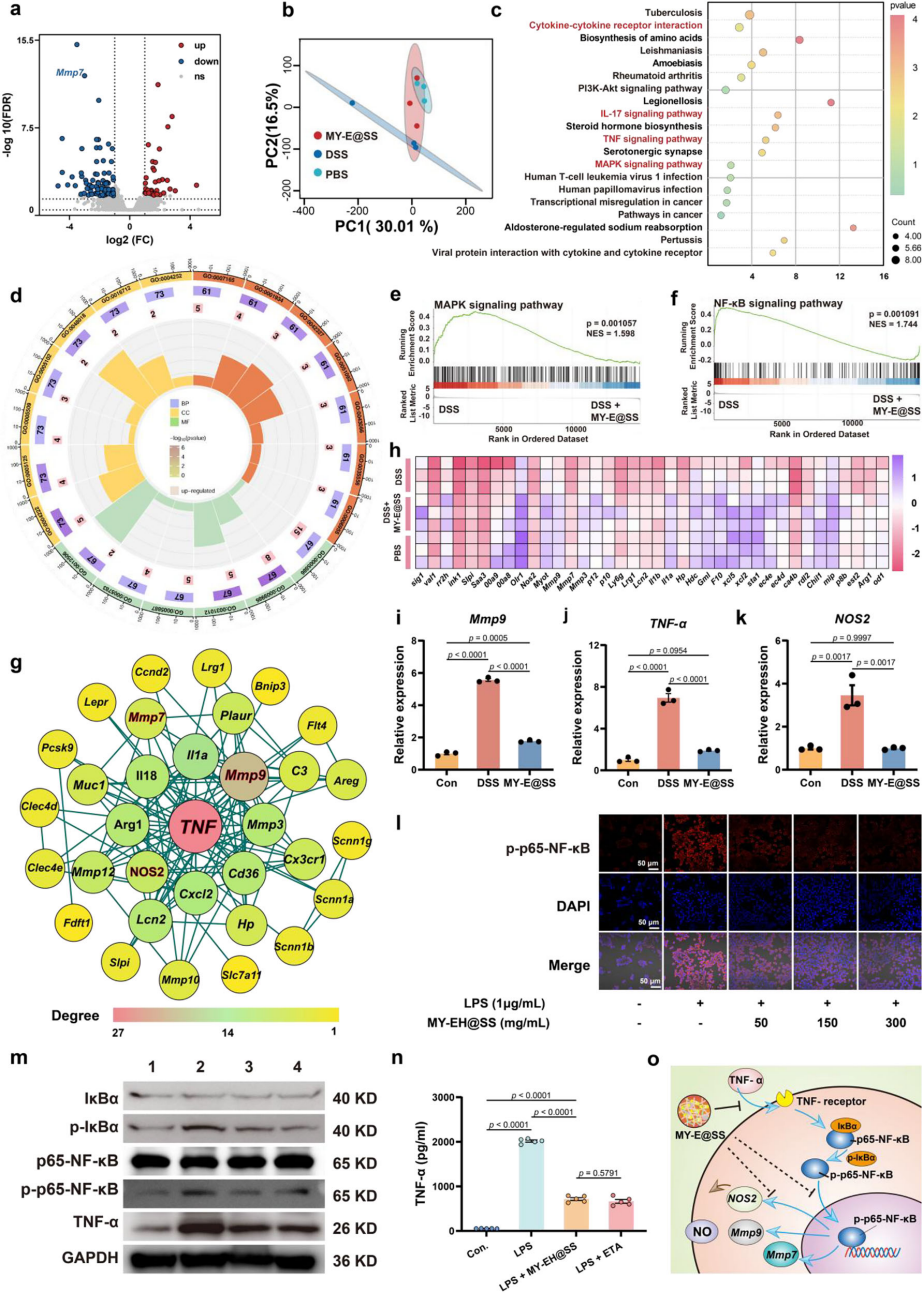
MY-E@SS reprograms intestinal gene expression in IBD mice. **a** Volcano plot of differentially expressed genes (DEGs) between DSS and MY-E@SS groups. Two-sided Wald test from DESeq2 with Benjamini–Hochberg (BH)-adjusted *p* values with false discovery rate (FDR). Red: significantly upregulated; blue: significantly downregulated. **b** Principal component analysis (PCA) of gene expression profiles. **c** KEGG pathway enrichment analysis of terms downregulated by MY-E@SS treatment. **d** GO analysis across BP, CC, and MF categories. **e**, **f** GSEA shows MY-E@SS-mediated suppression of MAPK (**e**) and NF-κB (**f**) signaling pathways. *p* values adjusted with BH (two-sided permutation test). **g** Protein-protein interaction (PPI) network of DEGs. **h** Heatmap of DEGs (Z-score normalized). **i-k** qRT-PCR validation of gene expression for matrix metallopeptidase 9 (*Mmp9*) (**i**), *TNF-α* (**j**), and nitric oxide synthase 2 (*NOS2*) (**k**). Data were normalized to GAPDH and expressed as mean ± SEM (*n* = 3). **l** Representative confocal microscopy images showing the inhibition of LPS-induced NF-κB p65 nuclear translocation in RAW264.7 macrophages by MY-EH@SS. Nuclei were stained with DAPI (blue), and p65 was immunofluorescently labeled (red). Representative of *n* = 3 biologically independent experiments. **m** Western blot analysis of key proteins in the TNF-α/NF-κB pathway in RAW264.7 macrophages. MY-EH@SS (150 μg/mL) inhibited LPS-induced IκBα phosphorylation (p-IκBα) and degradation, as well as p65-NF-κB phosphorylation (p-p65-NF-κB). Etanercept (ETA) was used as a positive control. Cells were treated as follows: Lane 1, control; Lane 2, LPS; Lane 3, LPS + MY-EH@SS (150 μg/mL); Lane 4, LPS + etanercept (ETA, positive control). All samples were derived from the same experiment and the gels/blots were processed in parallel. Representative of *n* = 3 biologically independent experiments. **n** ELISA quantification of TNF-α secretion in the cell culture supernatant of RAW264.7 macrophages. **o** Mechanism of action for MY-E@SS. Statistical analysis was performed using one-way ANOVA with Tukey’s test for panels (**i**, **j**, **k**, and **n**). Data are presented as the mean ± SEM. For panels **a**–**k**, *n* = 3 biologically independent experiments; for panel **n**, *n* = 5 biologically independent experiments. Source data are provided as a Source Data file. LPS Lipopolysaccharide; IκBα Inhibitor of kappa B alpha; p-IκBα Phosphorylated Inhibitor of kappa B alpha; p65-NF-κB p65 subunit of Nuclear Factor kappa B; p-p65-NF-κB Phosphorylated p65 subunit of Nuclear Factor kappa B; *NOS2* nitric oxide synthase 2.

Since the restoration of intestinal gene expression marks inflammation resolution, we hypothesized that MY-E@SS mediates this process. In order to explore the anti-inflammatory mechanism of MY-E@SS, we collected colon tissues for transcriptome analysis during the recovery period. Compared to the DSS group, MY-E@SS treatment resulted in the upregulation of 38 genes and downregulation of 84 genes (Fig. 9a), which contributed to distinct transcriptomic differences between the groups (Fig. 9b and S39) and ultimately restored the DSS-altered colonic gene expression to a pattern resembling healthy tissue (Fig. S40). KEGG analysis showed that MY-E@SS significantly suppressed key inflammatory pathways (Fig. 9c and S41–S43) such as cytokine-cytokine receptor interaction, PI3K-Akt, TNF, NF-κB, IL-17, and MAPK signaling, whereas DSS had the opposite effect (Fig. S44). Gene ontology (GO) analysis revealed that MY-E@SS treatment enriched differentially expressed genes (DEGs) in processes including complement activation, inflammatory response, and cytokine expression (Fig. 9d). This enrichment corresponded with the downregulation of inflammatory signaling to near-normal levels. In contrast, DSS-induced inflammatory responses, TNF signaling, and leukocyte migration were markedly upregulated, and this trend was significantly reversed by MY-E@SS intervention (Fig. S45 and S46). Gene Set Enrichment Analysis (GSEA) further confirmed that MY-E@SS treatment suppressed key processes like MAPK, immune response, and NF-κB signaling (Fig. 9e, f and S47). Notably, MY-E@SS effectively inhibited TNF signaling in the colon, resulting in an expression profile resembling the healthy state. Protein-protein interaction network analysis suggested that TNF, MMP9, and NOS may be key targets of MY-E@SS therapy (Fig. 9g). Interestingly, consistent with the above findings, a comprehensive analysis of all DEGs showed that MY-E@SS downregulated key genes in both the IL-17 (e.g., *Mmp9*, *Mmp7*) and TNF signaling pathways (Fig. 9h). TNF-α, a critical activator of the NF-κB pathway, can directly promote *Mmp9* transcription via downstream NF-κB activation^5,36^. MMP9, a zinc-dependent endopeptidase that degrades collagen and elastin in the extracellular matrix, is strongly associated with IBD progression^37^. Additionally, excessive nitric oxide production has been linked to various inflammatory, immune-mediated, and neurodegenerative diseases. To further verify these findings at the transcriptional level, we performed qRT-PCR to assess the expression of genes encoding *TNF-α*, *Mmp9*, *Mmp7*, and *NOS2*. As predicted, MY-E@SS treatment significantly downregulated the expression of these key genes (Fig. 9i-k, S48). To directly validate this mechanism, in vitro studies were conducted in LPS-stimulated RAW264.7 macrophages, where MY-E@SS treatment was observed to concentration-dependently inhibit LPS-induced p65 nuclear translocation. (Fig. 9l, S49). Further investigation revealed that at 150 μg/mL, it significantly suppressed TNF-α secretion to a level comparable to the inhibitor etanercept and concurrently reduced IκBα phosphorylation and degradation along with p65 phosphorylation (Fig. 9m, n, S50 and S51). These results demonstrate that MY-E@SS counteracts inflammation through directly inhibiting the TNF-α/NF-κB signaling pathway in immune cells, thereby suppressing downstream cytokine production and inflammatory activation (Fig. 9o).

### MY-E@SS regulated gut metabolic profile in IBD mice

**Fig. 10:**
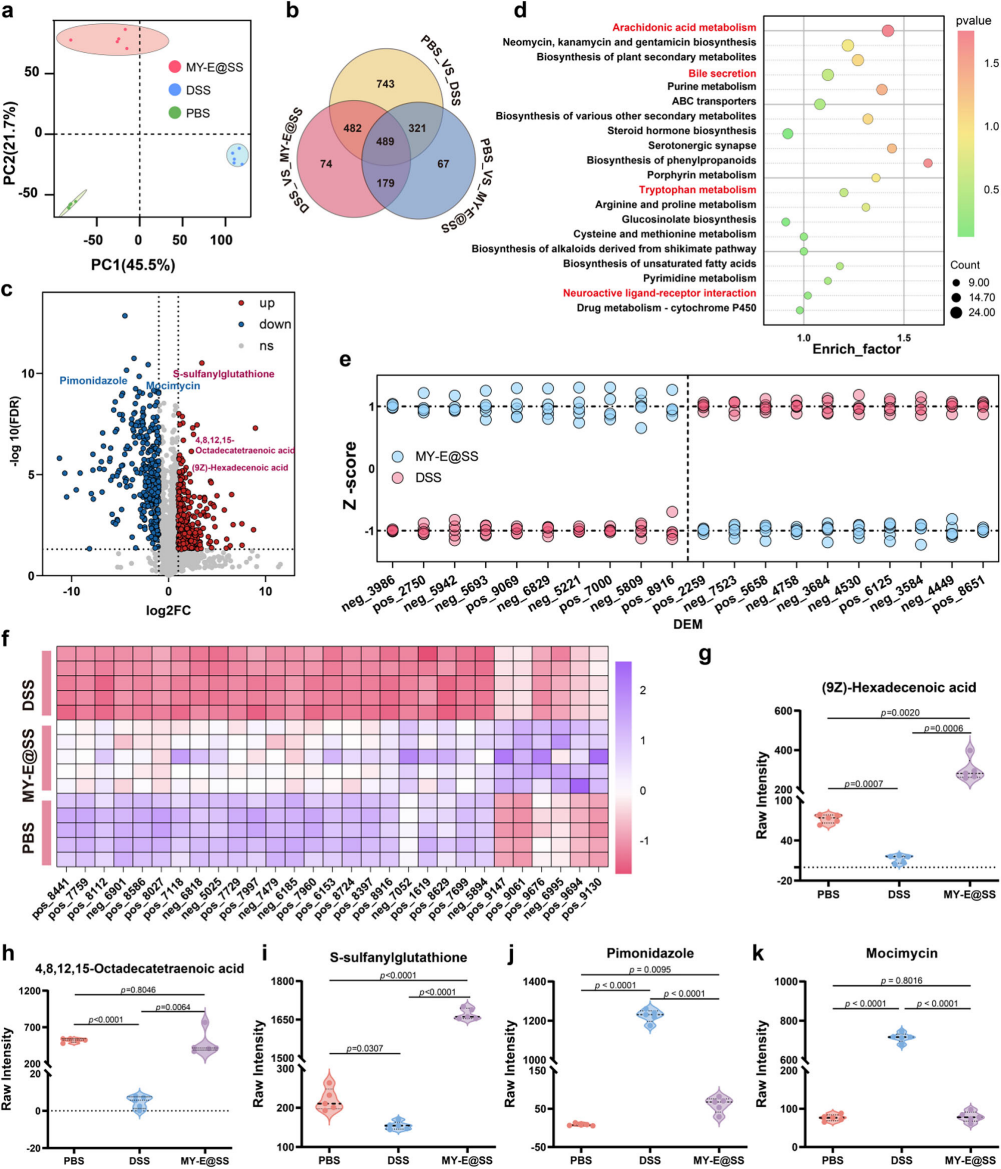
MY-E@SS regulates intestinal metabolism in a murine model of IBD. **a** PCA score plot of fecal metabolite profiles. **b** Venn diagram illustrating differentially accumulated metabolites (DAMs). **c** Volcano plot displaying DAMs between the DSS group and the MY-E@SS treatment group. Statistical significance was evaluated using a two-tailed Student’s t-test, with metabolites meeting the criteria of |log2(fold change)| > 1 and FDR < 0.05 were identified as DAMs. **d** KEGG pathway enrichment analysis of metabolites downregulated in the MY-E@SS group compared to the DSS group. **e** Z-score heatmap of the top 20 DAMs ranked by p-value (DSS vs. MY-E@SS). **f** Clustered heatmap of all identified DAMs. **g–k** Relative abundances of key metabolites: (9Z)-Hexadecenoic acid (**g**), 4,8,12,15-Octadecatetraenoic acid (**h**), S-sulfanylglutathione (**i**), Pimonidazole (**j**), Mocimycin (**k**). Data are presented as the mean ± SEM. Statistical analysis was performed using one way ANOVA with Tukey’s test (**g**–**k**). **a**–**k** *n*  =  5 biologically independent experiments. Source data are provided as a Source Data file. DSS Dextran sulfate sodium.

Beyond transcriptional alterations, we investigated the metabolic landscape using non-targeted metabolomics on colonic tissue. Multivariate analyses (PCA and OPLS-DA) revealed clear group separations, demonstrating that both DSS and MY-E@SS substantially reshaped the colonic metabolome (Fig. 10a, S52 and S53). The OPLS-DA model was validated as robust and not overfitted (Fig. S52), and it exhibited strong predictive power (Q² > 0.9) for subsequent analysis (Fig. S53). Several metabolite classes were identified, primarily consisting of fatty acyls, glycerophospholipids, and sterol lipids (Fig. S54). Relative to the PBS group, the DSS group displayed significant metabolite alterations (Fig. 10b), including 483 upregulated and 524 downregulated metabolites (Fig. S55a). KEGG pathway analysis revealed disruptions in key metabolites in the IBD model, such as bile acids (sterol lipids) and arachidonic acid (fatty acyls) (Fig. S56). Importantly, MY-E@SS treatment largely reversed these alterations, with 331 metabolites upregulated and 323 downregulated compared to the DSS group (Fig. 10c), thereby normalizing the overall metabolite profile (Fig. S55b). Differentially abundant metabolites were significantly enriched in metabolic pathways including arachidonic acid metabolism, bile secretion, GSH metabolism, and flavonoid biosynthesis (Fig. 10d). Heatmap analysis confirmed that MY-E@SS intervention markedly reversed DSS-induced alterations in specific metabolites across several classes: arachidonic acid derivatives (neg_5025, pos_6153, neg_5894, pos_9061), phospholipids (pos_8441, pos_7759, pos_8112, pos_8586, neg_6901, pos_7729), carotenoids (pos_8916), and bile acids along with their derivatives (neg_6818, neg_6185, pos_8629) (Fig. 10f). Further analysis showed that the MY-E@SS group exhibited significantly elevated levels of anti-inflammatory metabolites, such as GSH (pos_2750), neoxanthin (pos_8916), and retinol (pos_9069). Conversely, the levels of pro-inflammatory metabolites, including pimonidazole (pos_2259), PIP (22:3(10Z,13Z,16Z)/PGE2) (neg_3584), 11-dehydro-thromboxane B2 (neg_4449), (Z)-8-tetradecenal (pos_8651), and mocimycin (neg_4758), were substantially decreased (Fig. 10e, f). This transition holds significant functional relevance, given that GSH serves as a critical agent in free radical scavenging and redox homeostasis, while neoxanthin and retinol are known for their capacities to mitigate oxidative damage and suppress inflammation^38,39^. Notably, MY-E@SS also substantially increased key metabolites associated with arachidonic acid metabolism, such as 4,8,12,15-octadecatetraenoic acid (neg_5652) and (9Z)-hexadecenoic acid (neg_6772), along with the antioxidant S-sulfanylglutathione (neg_3986), bringing their concentrations close to normal levels (Fig. 10c, g–i). Concurrently, MY-E@SS significantly lowered the levels of pimonidazole (a hypoxia marker) and mocimycin (a protein synthesis inhibitor) (Fig. 10j, k). Collectively, these findings indicate that MY-E@SS alleviates DSS-induced metabolic disruption in the IBD model, largely via regulation of central pathways including arachidonic acid metabolism.

## Discussion

This work introduces a biomimetic encapsulation strategy to enhance the environmental tolerance of engineered bacteria and improve their diversity and safety. An inducible priming subsystem is used to increase the production of therapeutic proteins and enable precise controlled release in engineered strains. Concurrently, the release of anti-inflammatory peptides is regulated by the inflammatory response mechanism, thereby reducing the safety risks caused by continuous activation. This bionic encapsulation strategy further strengthens environmental isolation and broadens the operational safety boundary of engineered bacteria. Nevertheless, the off-target effect and horizontal gene transfer risk of engineered bacteria cannot be completely eliminated. In order to address these challenges and promote personalized treatment, future promoter designs can be tailored to the individual characteristics of patients. For example, inflammation-responsive promoters or self-inducible promoters can be utilized. Inflammation-responsive promoters can be specifically activated by overexpressed inflammatory factors (such as TNF-α, IL-6, NO, etc.) in the local microenvironment; the self-inducible promoter realizes the spatiotemporal regulation of gene expression through the bacterial quorum sensing system, ensuring that the therapeutic protein is only released in the target microenvironment, thereby avoiding systemic risks caused by continuous activation. In summary, by integrating biomimetic and synthetic biology strategies, our constructed MY-E@SS successfully achieves intelligent sensing and precise repair of the inflammatory microenvironment. As such, this work provides a translatable feasible strategy for the collaborative management of IBD and other inflammatory diseases.

## Methods

Materials. The EcN strain (maintained in our laboratory) was used. The expression vector pBAD33 (Addgene, HG-VYA1388) and the fluorescent-labeled vector pTD103luxI-sfGFP (Addgene, HG-VYA1090) were purchased from HonorGene (Changsha, China). Sodium carbonate (Na₂CO₃, ≥99 %, Cat# S924146) was obtained from Macklin. Dopamine hydrochloride (Sigma, Cat# H8502-5G) and D-mannose (Macklin, Cat# D813082) were purchased from Sigma and Macklin, respectively. Cell culture reagents included DMEM high-glucose medium (Gibco, Cat# 11965092). Dextran sulfate sodium (DSS, MW 36–50 kDa, Cat# D0361) was obtained from Shenzhen Lijing Biochemical. All chemicals were used as received without further purification.

Engineering of MY-EcN. Following the determination of the MY amino acid sequence through bioinformatic analysis, the corresponding gene sequence was optimized according to the codon usage bias of EcN. The MY-pBAD33 recombinant vector was constructed using seamless cloning technology (pClone007 Simple Vector Kit, Tsingke Biotech, Beijing) and sequenced by Tsingke Biotech (Changsha). Plasmid validation and application were achieved through transformation into EcN.

Identification of MY-EcN. Single colonies grown on LB agar plates with chloramphenicol (25 μg/mL) were picked for colony PCR using pBAD33-specific primers (Table S7). Purified amplicons were sequenced by Tsingke Biotech (Beijing, China), and alignment with the target sequence via SnapGene confirmed genetic accuracy. Uncropped and unprocessed scans of the gels are provided in the Source Data file.

Preparation of MY-E@SS. Silk fibroin (SF) was extracted by boiling cocoons in Na₂CO₃, drying, and dissolving in LiBr solution (40.39 g/50 mL), followed by dialysis to obtain 6% SF solution. The SF (5 mL) was activated with EDC/NHS for 30 min, then reacted with dopamine and mannose for 1 h to form microcapsule material. Bacterial concentrate (OD₆₀₀ = 10) was mixed with the material (1:10 ratio) as the dispersed phase (DP), which was combined with oil phase (CP, 0.5% soy phosphatidylcholine) via droplet microfluidics (DP:1 μL/min, CP:14 μL/min). Microcapsules were purified by centrifugation, gradient freezing (4°C → −20°C → −80°C), and lyophilization.

Characterization of MY-E@SS. The morphology and particle size of the samples were examined using scanning electron microscopy (SEM; Hitachi SU8000) and transmission electron microscopy (TEM; JEOL JEM-1400). FTIR spectroscopy (JASCO FT/IR-4700) was employed to identify characteristic chemical functional groups. Elemental composition and chemical states were analyzed via X-ray photoelectron spectroscopy (XPS; Thermo Scientific K-Alpha). Crystallinity was assessed by X-ray diffraction (XRD; Shimadzu XRD-7000). Hydrodynamic diameter, zeta potential, and dispersion stability were measured by dynamic light scattering (DLS; Malvern Zetasizer Nano ZS). Thermal stability was evaluated through thermogravimetric analysis (TGA; TA Instruments Q50).

Based on an LC-MS/MS analytical method for biomolecules/peptides. Sample preparation was conducted according to enzymatic digestion requirements. For enzymatic digestion, 5 mg of protease was added to 1 mL of sample supernatant and incubated at 25°C for 4 hours, after which the reaction was terminated by heating at 85 °C. Non-digested samples proceeded directly to subsequent steps. Both sample types were then subjected to ultrafiltration (10 kDa molecular weight cutoff, 4 °C, 12,000 rcf) to isolate low-molecular-weight fractions. A consistent reduction and alkylation protocol was applied: first, reduction with 10 mM DTT at 56 °C for 1 h, followed by alkylation using 20 mM IAM under dark conditions at room temperature for 40 min, and finally quenching with additional DTT. Following this, all samples were desalted using C18 Stage-Tip columns and dried under vacuum at 45 °C. For instrumental analysis, nanoflow liquid chromatography was performed on a C18 column with a mobile phase composed of 0.1% formic acid in water (solvent A) and 0.1% formic acid in 80% acetonitrile (solvent B), at a flow rate of 600 nL/min over a 35-minute gradient. This was coupled to a mass spectrometer operated in data-dependent acquisition mode, with a full-scan range of m/z 100–1500 at a resolution of 60,000.

Sustained-release profiling of microcapsules. Vitamin B12-loaded microcapsules coated with SS were dispersed in saline (0.9% NaCl). At predetermined intervals, 500 µL aliquots were collected, and the absorbance at 550 nm was measured using a spectrophotometer to quantify release kinetics.

Growth curve analysis. Bacterial growth was systematically evaluated under three conditions: (1) Normal growth in LB medium at 37 °C for 16 h (control); (2) Gastric tolerance by pre-treatment in SGF (0.32% pepsin, pH 1.2, 37 °C) for 1.5 h followed by LB medium recovery for 16 h; and (3) Intestinal resistance through incubation in simulated intestinal fluid (0.16% pancreatin, pH 7.0, 37 °C) for 4 h prior to 16h LB medium cultivation. Growth profiles were monitored by hourly OD₆₀₀ measurements.

Assessment of bacterial/byproduct leakage from microcapsules. Microcapsules (1 g) were incubated in 5 mL of sterile tissue-mimetic fluid (3 mM glucose + 0.9% NaCl, pH 7.35) at 37  °C. At predetermined intervals, 300 μL aliquots of the supernatant were withdrawn, plated in triplicate onto LB agar, and subjected to viable colony enumeration. The cumulative bacterial leakage into the 5 mL volume was extrapolated from these measurements, with a free bacterial suspension serving as the control (0 h).

Assessment of strain preservation stability. The viability of both fluorescent bacteria and sfGFP‑E@SS lyophilized powders during room‑temperature storage was assessed through periodic sampling. Retrieved samples were reactivated in LB medium for 1 h at 37  °C, followed by serial dilution and plating for viable colony enumeration to determine preservation stability.

RONS scavenging abilities. The radical scavenging capacities of the materials were systematically evaluated against key RONS using a panel of standardized assays. (1) Scavenging activities against nitric oxide (·NO, using ·PTIO), ABTS radical cation (·ABTS⁺), and DPPH radical (·DPPH) were determined by monitoring absorbance at their characteristic wavelengths (557, 734, and 420–620 nm, respectively), with half-maximal inhibitory concentration (IC_50_) values derived from corresponding dose–response curves. (2) Hydroxyl radical (·OH) elimination was assessed via TMB colorimetric (450 nm) and salicylate (510 nm) methods within Fenton reaction systems. (3) Inhibition of superoxide anion (·O₂⁻) was quantified by measuring pyrogallol autoxidation at 299 nm in a Tris-HCl buffer system. (4) Anti-lipid peroxidation potential was examined with the thiobarbituric acid reactive substances (TBARS) assay (535 nm) following Fe^3+^-induced lecithin oxidation. All measurements were performed in triplicate, with scavenging rates calculated as [1 − (A/A₀)] × 100%, where A and A₀ denote the absorbance values of the test sample and the blank control, respectively. This integrated analytical strategy enables a comprehensive assessment of the antioxidant efficacy against major RONS.

Fecal occult blood test. Fecal samples were homogenized with saline (0.9% NaCl) and centrifuged (3000 × g, 5 min). The supernatant was mixed with 1% o-tolidine reagent (in glacial acetic acid-ethanol solution). Color development (blue indicates positive occult blood) was assessed semi-quantitatively based on reaction kinetics and intensity (graded from - to ++++).

Animals and Housing. Male C57BL/6 J mice (6–8 weeks old, 21–23 g, stock number: N000013) used in colitis models were bred and housed under specific pathogen-free (SPF) conditions. The mice were maintained in microisolator cages on individually ventilated racks, with aspen chip bedding, in a controlled environment (temperature: 20–22 °C, humidity: 50%–60%, 12-h light/dark cycle). Autoclaved rodent chow and water were provided ad libitum. Mice were acclimatized for one week prior to experiments. Both experimental and control groups were housed separately in different cages. All experiments were performed using male C57BL/6 J mice. All efforts were made to minimize animal suffering during the experimental procedures.

Ethics Statement. The animal study was approved by the Ethics Committee of Hunan Agricultural University, and the animal certification number is Ethics Approval 2023 No. 146. The animal experiment guidance from the Ethics Committee of Hunan Agricultural University and the Guide for the Care and Use of Laboratory Animals from the NIH were followed throughout the entire experiment.

Establishment of DSS-induced IBD model and drug administration regimen. Male C57BL/6 mice (n = 5 per group) were administered 3% DSS for 7 days to establish the experimental IBD model. Concurrently, after grouping, the mice were orally gavaged with 100 μL of PBS, P-EcN, MY-EcN, SS, or MY-E@SS, at a bacterial dose of 1 × 10⁸ CFU per administration, every other day. Throughout the treatment period, all mice were provided with drinking water containing L-arabinose (10 g/L) to induce the sustained expression of MY. During the experiment, changes in body weight and fecal occult blood in mice from each group were closely monitored.

Establishment of TNBS-induced IBD model and drug administration regimen. Male C57BL/6 mice were used to establish a colitis model via rectal administration of TNBS. The animals were then divided into six groups (*n* = 5 per group) and orally gavaged with 100 μL of PBS, P-EcN, MY-EcN, SS, or MY-E@SS, at a bacterial dose of 1 × 10⁸ CFU once daily for four consecutive days starting from day 1.

Long-term biocompatibility assessment. Male C57BL/6 mice (n = 5 per group) were administered 100 μL of either ovalbumin (OVA, 20 mg/kg), MY-E@SS, or PBS via oral gavage every two days. On day 29, mice were challenged with an equal volume but fourfold concentration of the corresponding reagent (OVA 60 mg/kg, MY-E@SS or PBS at an equivalently elevated concentration) 15 minutes prior to euthanasia. The allergic response was scored according to the following criteria: 0 (no symptoms), 1 (scratching around the mouth and nose), 2 (swelling around the eyes and mouth, reduced activity, increased respiratory rate), 3 (labored breathing, cyanosis of the lips and tail), 4 (excitation, tremors, and muscle contractions), and 5 (shock or death).

Metabolic parameter analysis. Prior to experimentation, all animals were acclimated to facility conditions for at least one week. Male mice were maintained under a 12h light/12h dark cycle at a controlled ambient temperature of 20–22 °C. Following the induction of the IBD model and subsequent interventions, metabolic parameters were continuously monitored over a 24h period using an International FOXBOX™ field metabolic analysis system. Data acquired during this continuous recording period were normalized to body weight to calculate rates of fuel oxidation and the RER.

In vitro hemolysis assay. Mouse anticoagulated whole blood was centrifuged (1500 × g, 10 min) to prepare 2% (v/v) erythrocyte suspension in normal saline. The suspension was mixed with MY-E@SS samples (25-1000 μg/mL) at 1:10 volume ratio and incubated at 37 °C for 3 h, with normal saline and ddH_2_O serving as negative and positive controls, respectively. After centrifugation (1500 × *g*, 10 min), supernatant absorbance at 540 nm was measured to calculate hemolysis percentage.

Cell viability assay (CCK-8 protocol). Cells were seeded in 96-well plates at 1 × 10⁴ cells/well and cultured for 24 h. After treatment with varying doses of MY-SS, 10 μL CCK-8 solution was added to each well followed by 2 h incubation (37 °C, light-protected). Absorbance at 450 nm was measured, and viability was calculated as: Viability (%) = [(A_treated_ - A_blank_)/(A_control_ - A_blank_)] × 100%.

Establishment of an inflammatory model in RAW264.7 cells. RAW264.7 cells were cultured in 6‑well plates at a working volume of 2 mL per well. After reaching an appropriate growth state, the cells were divided into the following groups: a blank control group, an LPS‑model group (stimulated with 0.1 μg/mL LPS), and LPS combined with different volumes of the test material.

Assessment of microencapsulation efficiency (EE). To quantify encapsulation efficiency, sfGFP‑E@SS microcapsules were dissolved in disintegration buffer (pH 7.5; 50 mM sodium citrate, 20 mM NaHCO₃, 10 mM Tris‑HCl) at 37  °C for 30 min. Both pre‑ and post‑encapsulation bacterial suspensions were subjected to serial ten‑fold dilution and plated on LB agar (37 °C, 24 h). EE was calculated according to the following formula: EE(%) = (N_released_ /N_initial_) × 100%.

Assessment of Intestinal Colonization Kinetics. Mice were assigned to three groups (PBS, sfGFP‑E@SS, and sfGFP‑EcN) and administered the respective suspensions via oral gavage. At 1, 4, 24, and 72 h following administration, animals were euthanized, and tissues (small intestine, cecum, and colon) were collected. The harvested tissues were weighed, homogenized in sterile PBS, and subjected to serial ten‑fold dilution. The resulting dilutions were then plated onto kanamycin‑containing LB agar for enumeration of fluorescent colonies. Colonization levels, expressed as CFU per administered dose (CFU/dose), were determined to evaluate bacterial persistence.

Multi-omics profiling. Following a 7-day acclimation period, IBD was induced in C57BL/6 mice by administering 3% DSS in their drinking water for 7 days (days 0–7). Subsequently, the mice received a 7-day therapeutic intervention (days 7–14). All mice were euthanized on day 16. Colon tissues and luminal contents were collected, immediately snap-frozen in liquid nitrogen, and stored for subsequent analyses: transcriptomic and metabolomic profiling of the tissues, and 16S rRNA sequencing of the gut microbiota. All analyses were conducted using the BMK Cloud platform.

Cellular mechanism studies. The anti-inflammatory mechanism of MY-EH@SS was assessed in RAW264.7 macrophages stimulated with LPS (0.5 µg/mL). To evaluate NF-κB inhibition, cells were treated with LPS and MY-EH@SS (0–300 µg/mL) for 6 h, and p65 nuclear translocation was visualized by confocal microscopy and quantified. For pathway analysis, cells were pretreated with 150 µg/mL MY-EH@SS or etanercept (ETA, 1 µg/mL, positive control) before LPS stimulation. TNF-α in supernatant was measured by ELISA. Cell lysates were analyzed by western blot for p-IκBα, IκBα, p-p65-NF-κB, p65-NF-κB, and TNF-α, with GAPDH as loading control. Uncropped and unprocessed scans of the key western blots presented in this study are provided in the Source Data file accompanying this paper.

Statistical analysis and reproducibility. Representative images are shown from one of three independent experiments. All data are presented as mean ± standard error of the mean (SEM) from at least three independent replicates. Statistical analyses were conducted using GraphPad Prism 9.5.1 software. One-way analysis of variance (ANOVA) was used for multiple group comparisons, and two-tailed Student’s t-test was applied for pairwise comparisons. Details of the statistical methods used for each dataset are provided in the figure legends. A p-value  <  0.05 was considered statistically significant.

### Reporting summary

Further information on research design is available in the Nature Portfolio Reporting Summary linked to this article.

## Supplementary information

Supplementary information

Description Of Additional Supplementary File

Supplementary Movie 1.

Reporting summary

Transparent Peer Review file

## Source data

Source data

## Data Availability

The DNA sequence generated in this study has been deposited in the National Center for Biotechnology Information (NCBI) GenBank database under accession number PP894312. The raw 16S rRNA gene sequences data generated in this study have been deposited in the NCBI SRA database under BioProject accession number PRJNA1439786. The 16S rRNAgene sequencing data generated in this study have been deposited in the NCBI SRA database under BioProject accession number PRJNA1440972. The mass spectrometry metabolomics data generated in this study have been deposited in the China National Center for Bioinformation (NGDC) under accession number PRJCA060448. All data from this study are fully available within the article, Supplementary Information or Source Data file. Any additional requests for information can be directed to, and will be fulfilled by, the corresponding authors. Source data are provided with this paper.
