# Supplementary material for: Bioinspired microcapsule reactor with engineered probiotics for IBD therapy
Source: Nat Commun. 2026 Jul 13;17:6095. doi: 10.1038/s41467-026-72027-1 (PMC13358097; doi:10.1038/s41467-026-72027-1)
Supplement: Supplementary file 1 — Supplementary information [file 41467_2026_72027_MOESM1_ESM.pdf]

Supporting Information

Bioinspired Microcapsule Reactor with Engineered Probiotics for IBD Therapy

Minhui Xu[1], Yuanchun Du[2,*], Guangfu Feng[1,*]

[1]College of Bioscience and Biotechnology, Hunan Agricultural University, Changsha 410128, Hunan, China

[2]College of Chemistry and Materials Science, Hunan Agricultural University, Changsha 410128, Hunan, China

*Corresponding authors: Yuanchun Du, Email: Dyc2025@hunau.edu.cn; Guangfu Feng, Email: GuangfuFeng@hunau.edu.cn

Contents

Supplementary Figures **4**
Figure S1. Construction of recombinant vector MY-pBAD33. 4
Figure S2. Western blot analysis of MY protein expression in LB medium. 5
Figure S3. LC-MS/MS analysis of proteins MY and P before enzymatic digestion. 6
Figure S4. Temporal and distribution analysis of active peptide KPV released by MY-E@SS in a DSS-induced IBD mouse model. 7
Figure S5. Quantification of MY protein expression levels in colonic contents of mice from each group by ELISA. 8
Figure S6. Extraction procedure of silk fibroin protein. 9
Figure S7. Antioxidant capacity assays of four engineered bacterial microcapsules. 10
Figure S8. Mathematical modeling for optimal anti-inflammatory engineered bacterial microcapsule selection using the entropy weight-TOPSIS method. 11
Figure S9. Macroscopic characterization of SF, SS, and MY-E@SS. 12
Figure S10. CFU of bacteria before and after encapsulation. 13
Figure S11. Schematic of pTD103luxI_sfGFP vector. 14
Figure S12. Culture of sfGFP-E@SS and naked sfGFP-EcN in tissue-mimicking fluid. 15
Figure S13. Room temperature storage stability of sfGFP-E@SS versus naked sfGFP-EcN. 16
Figure S14. MY-EH and SS ameliorated LPS-induced morphological damage in Raw264.7. 17
Figure S15. Fecal occult blood test results from different experimental groups. 18
Figure S16. Representative images showing spleen size and morphology across experimental groups. 19
Figure S17. Assessment of tight junction protein expression based on immunofluorescence staining and quantitative analysis. 20
Figure S18. Immunophenotypic analysis of key immune cell subsets in colonic tissues following MY-E@SS treatment. 21
Figure S19. Dynamics of regulatory T cells (Tregs) in the colonic mucosa. 22
Figure S20. Hematoxylin and eosin (H&E)-stained sections of major organs demonstrating systemic biocompatibility. 23
Figure S21. Hematological parameters in mice from different treatment groups. 24
Figure S22. Quantitative analysis of neutrophil elastase (NE) expression and neutrophil infiltration at the inflammatory site 25
Figure S23. Changes in serum oxidative stress markers in IBD mice after different interventions. 26
Figure S24. Therapeutic efficacy of MY-E@SS in a TNBS - induced IBD model 27
Figure S25. Quantitative analysis of intestinal GFP fluorescence intensity and in vivo tracking of probiotics. 29
Figure S26. Spatiotemporal fluorescence imaging of sfGFP-E@Cy5-SS microcapsules in IBD model mice. 30
Figure S27 Temporal and spatial distribution of MY-EcN/MY-E@SS by PCR analysis. 31
Figure S28. Hematological profiling of normal mice with/without MY-E@SS gavage. 32
Figure S29. Quantitative analysis of pro- and anti-inflammatory cytokine levels in colon tissues at day 17 post-treatment. 33
Figure S30. Assessment of bacterial translocation via sfGFP-tagged strain detection. 34

Figure S31. Extended (29-day) *in vivo* biocompatibility and immunogenicity assessment of MY-E@SS in healthy mice. 35
Figure S32. Assessment of immunogenicity and hypersensitivity potential after extended administration of MY-E@SS. 36
Figure S33. Quantitative analysis of the restorative effects of various treatment groups on key circadian parameters of respiratory metabolism. 37
Figure S34. Evaluation of MY-E@SS biocompatibility in healthy mice based on respiratory metabolic parameters. 38
Figure S35. Compositional analysis of gut microbiota across treatment groups. 39
Figure S36. Compositional analysis of gut microbiota across treatment groups 40
Figure S37. Microbial community composition at the phylum, genus, and species levels. 41
Figure S38. Temporal dynamics of typical probiotic and harmful bacterial abundances. 42
Figure S39. Venn diagram analysis of differentially expressed genes. 43
Figure S40. Volcano plots of differentially expressed genes. 44
Figure S41. KEGG pathway map of the TNF signaling pathway in DSS and MY-E@SS groups. 45
Figure S42. KEGG pathway map of the MAPK signaling pathway in DSS and MY-E@SS groups. 46
Figure S43. KEGG pathway map of the IL-17 signaling pathway in DSS and MY-E@SS groups. 47
Figure S44. KEGG pathway enrichment of upregulated terms in DSS group (vs PBS). 48
Figure S45. GO enrichment analysis of downregulated terms in MY-E@SS group (vs DSS). 49
Figure S46. GO pathway enrichment of upregulated terms in DSS group (vs PBS). 50
Figure S47. GSEA showing MY-E@SS-mediated regulate of immune response. 51
Figure S48. qRT-PCR analysis of *Mmp7* mRNA expression in mouse colon tissues from different groups. 52
Figure S49. Quantification of p65 nuclear translocation from confocal images. 53
Figure S50. Inhibition of the TNF-α/NF-κB pathway by MY-EH@SS in RAW*264.7* macrophages. 54
Figure S51. Quantitative analysis of TNF-α/NF-κB pathway inhibition by MY-E@SS in RAW*264.7* macrophages. 55
Figure S52. Permutation test of OPLS-DA model. 56
Figure S53. OPLS-DA score plots of untargeted metabolomics data. 57
Figure S54. Top 20 lipid categories classified by LIPID MAPS database. 58
Figure S55. Volcano plots of DAMs. 59
Figure S56. KEGG pathway enrichment analysis of upregulated metabolites in DSS group (vs PBS). 60

Supplementary Tables........ 61
Table S1 The rusult of LC-MS/MS (MY-EH) 61
Table S2. Positive-negative ideal solution 62
Table S3. Summary of weight calculation results based on entropy weight TOPSIS method 63
Table S4. TOPSIS evaluation results 64
Table S5. Disease activity index (DAI) parameters and their associated scoring 65
Table S6. Histological grading scheme for DSS colitis 66
Table S7. PCR primer sequences in this study 67

Reference........ 68

Supplementary Figures

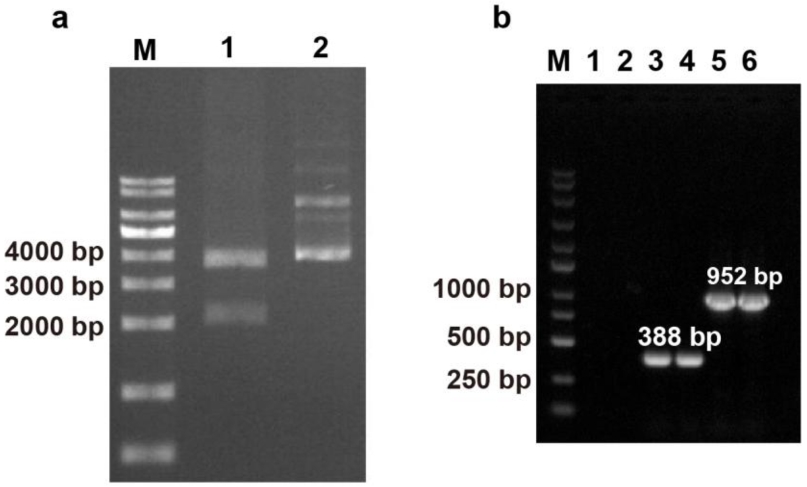

Figure S1. Construction of recombinant vector MY-pBAD33. (a) Electrophoresis of MY-*pBAD33* after double digestion. Lane 1: HindIII + NcoI double digestion identification; Lane 2: Original plasmid electrophoresis (integrity verification). (b) Colony PCR verification of successful MY-EcN construction. Lanes 1-2: Original EcN strain; Lanes 3-4: P-EcN strain containing empty vector *pBAD33*; Lanes 5-6: Engineered strain MY-EcN. M: DNA marker. Representative images are shown from one of three independent experiments. Source data are provided as a Source Data file.

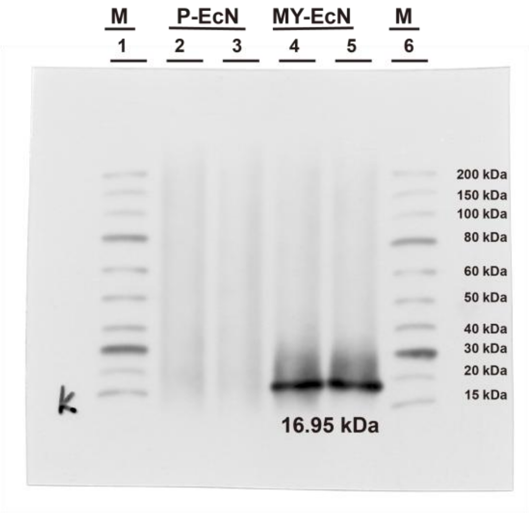

Figure S2. Western blot analysis of MY protein expression in LB medium. Lane 1-2: Protein expression from P-EcN (empty vector control); Lane 3-4: Protein expression from MY-EcN (recombinant strain). Representative images are shown from one of three independent experiments. Source data are provided as a Source Data file.

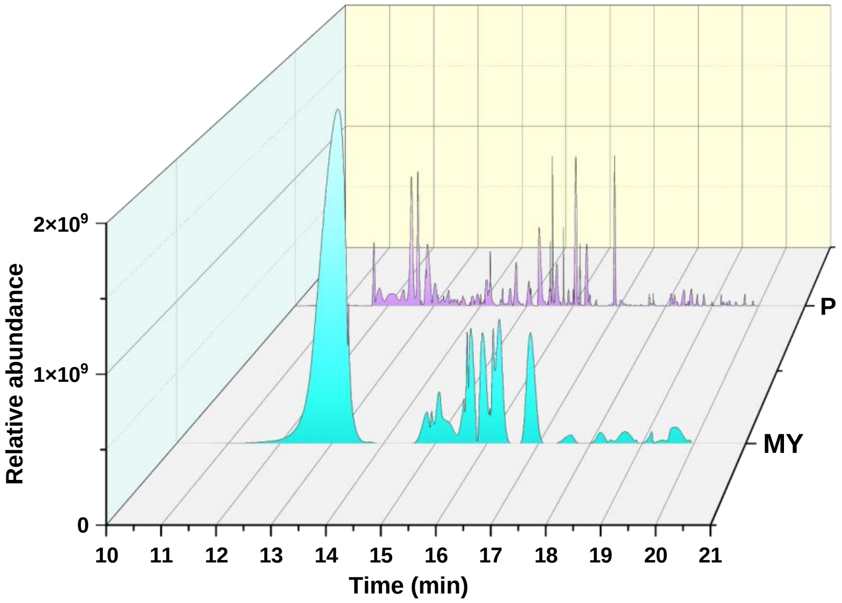

Figure S3. LC-MS/MS analysis of proteins MY and P before enzymatic digestion.

Note: MY / P are the secreted protein of MY-EcN or P-EcN, respectively, before neutrophil elastase digestion. Source data are provided as a Source Data file.

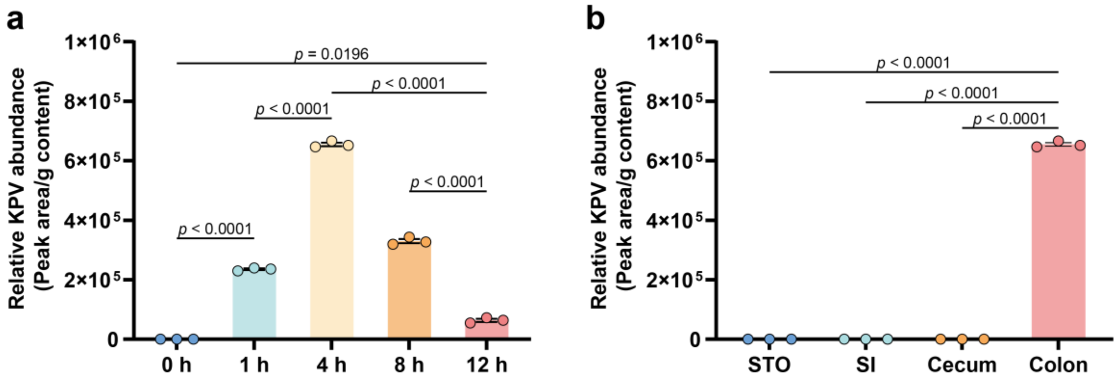

Figure S4. Temporal and distribution analysis of active peptide KPV released by MY-E@SS in a DSS-induced IBD mouse model. (a). Time-course changes in KPV concentration in colonic contents. (b). Distribution of KPV in the contents of different gastrointestinal segments at 4 h post-gavage. Data are presented as the mean ± SEM (n=3). Statistical analysis was performed with one way ANOVA with Tukey's test. Source data are provided as a Source Data file.

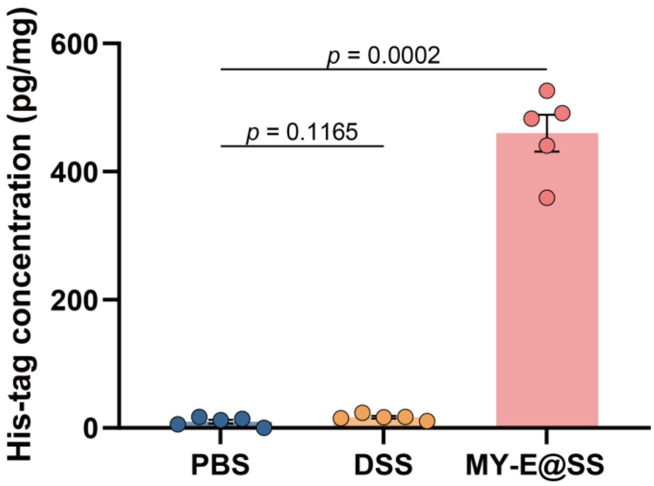

Figure S5. Quantification of MY protein expression levels in colonic contents of mice from each group by ELISA. Data are presented as the mean ± SEM (n=5). Statistical analysis was performed with one way ANOVA with Tukey's test. Source data are provided as a Source Data file.

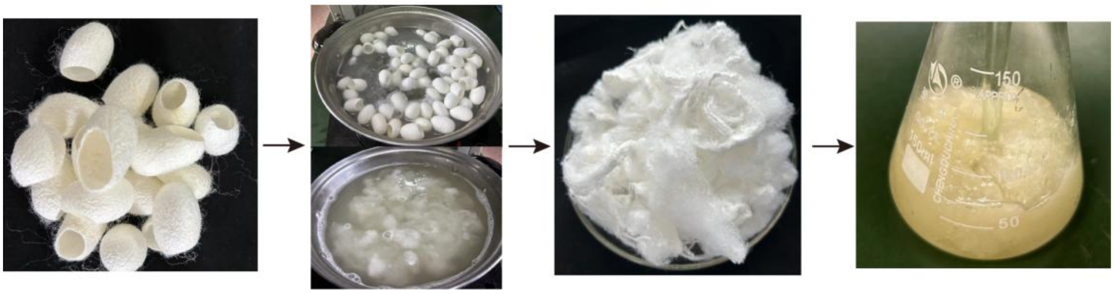

Figure S6. Extraction procedure of silk fibroin protein. Representative images are shown from one of three independent experiments.

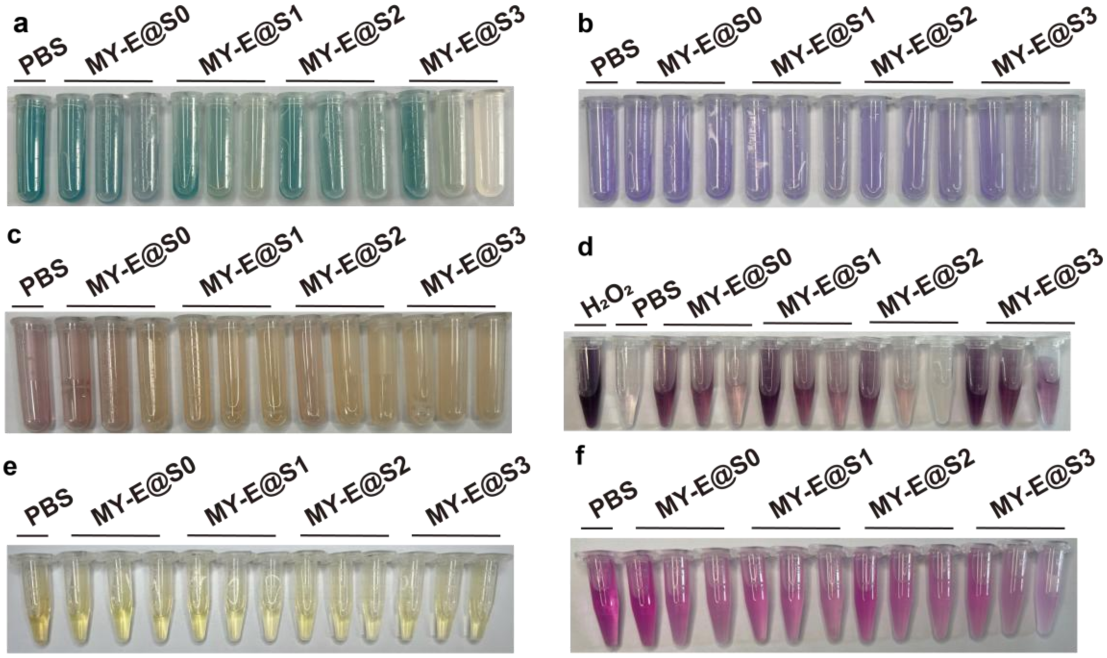

Figure S7. Antioxidant capacity assays of four engineered bacterial microcapsules. (a) $ABTS^{+}\cdot$ radical scavenging assay. (b) PTIO· radical scavenging assay. (c) DPPH· radical scavenging assay. (d) Hydroxyl radical (·OH) scavenging assay. (e) Superoxide anion radical ($O_2^{-}\cdot$) scavenging assay. (f) SOD-like enzyme activity measurement of microcapsules at different concentrations. Representative images are shown from one of three independent experiments.

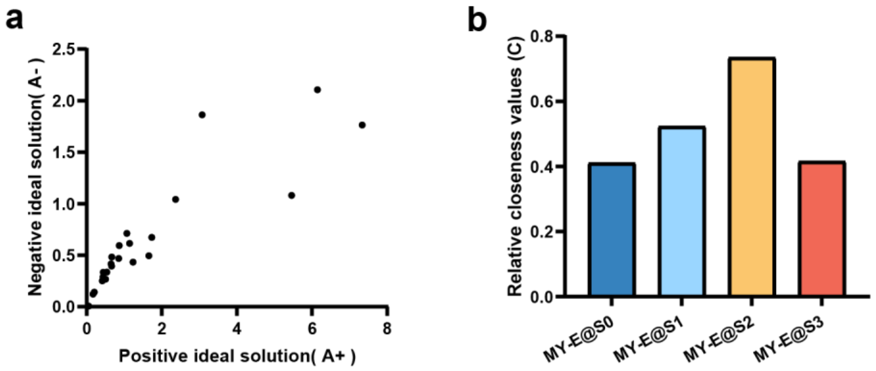

Figure S8. Mathematical modeling for optimal anti-inflammatory engineered bacterial microcapsule selection using the entropy weight-TOPSIS method. (a) Positive and negative ideal solutions (A^+ and A^-) identified for each evaluation parameter. (b) Comparison of relative closeness values (C) among microcapsule variants, with a higher C value indicating greater proximity to the ideal solution. Source data are provided as a Source Data file.

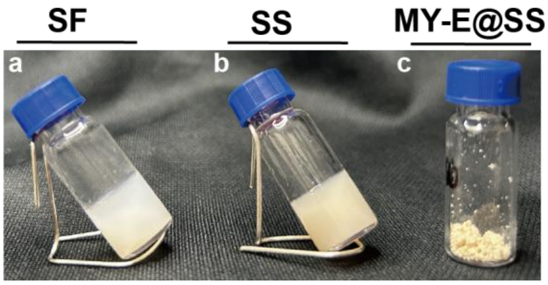

Figure S9. Macroscopic characterization of SF, SS, and MY-E@SS. (a, b) Photographic images showing the hydrogel formation of both SF and SS, with SS exhibiting a darker coloration. (c) MY-E@SS appears as an off-white powder demonstrating wall-adhesion properties in glass vials. All samples were prepared under identical conditions at room temperature (25±1°C). Representative images are shown from one of three independent experiments.

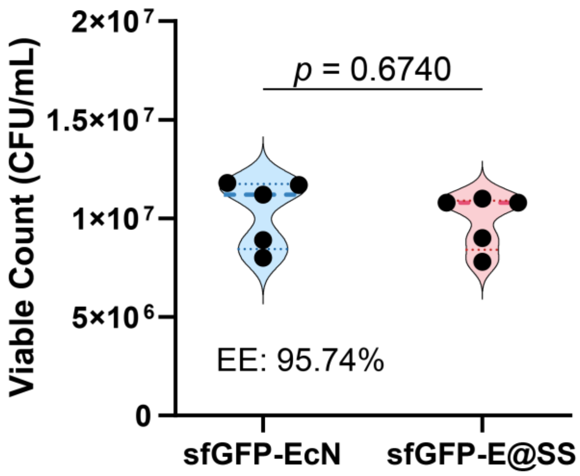

Figure S10. CFU of bacteria before and after encapsulation. Data are presented as the mean ± SEM (n=5 biologically independent experiments). Source data are provided as a Source Data file. EE, Encapsulation efficiency.

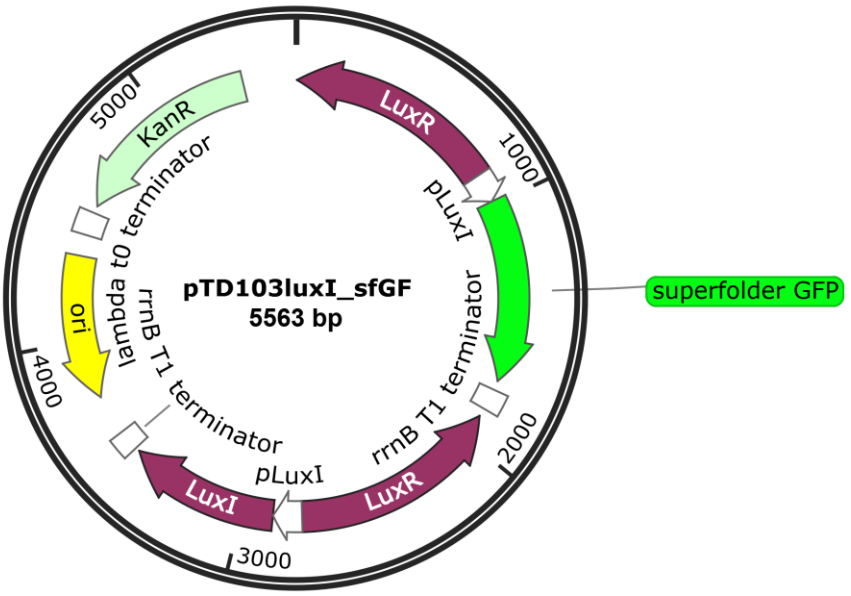

Figure S11. Schematic of pTD103luxI_sfGFP vector. Diagram shows the luxI promoter-driven sfGFP expression construct with essential regulatory elements and restriction sites.

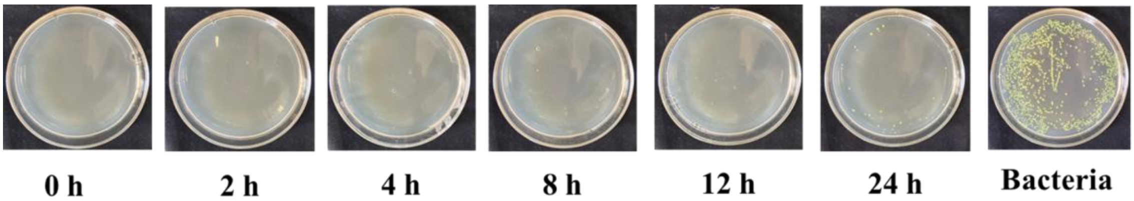

Figure S12. Culture of sfGFP-E@SS and naked sfGFP-EcN in tissue-mimicking fluid.

Note: although trace leakage (~40 CFU) was observed at 24 hours, this value was several orders of magnitude lower than the total encapsulated bacterial load (~1×10^8 CFU), rendering it negligible in practical scenarios. Representative images are shown from one of three independent experiments.

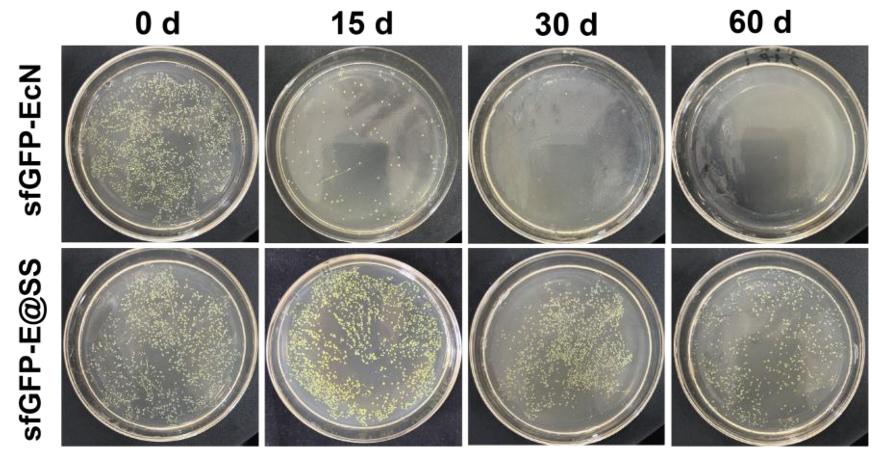

Figure S13. Room temperature storage stability of sfGFP-E@SS versus naked sfGFP-EcN. Representative images are shown from one of three independent experiments.

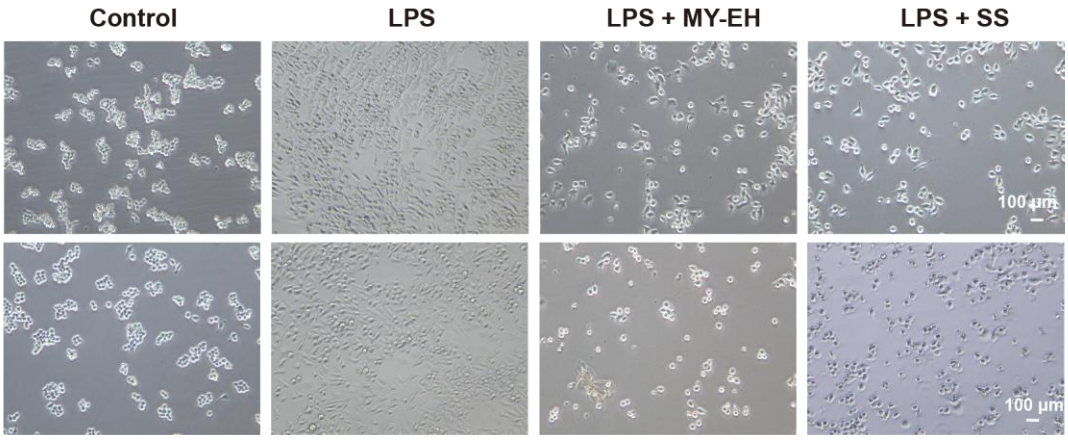

Figure S14. MY-EH and SS ameliorated LPS-induced morphological damage in Raw264.7. Upper row (Low concentration): MY-EH 4 ng/mL, SS 150 μg/mL. Lower row (High concentration): MY-EH 8 ng/mL, SS 300 μg/mL. Representative images are shown from one of three independent experiments.

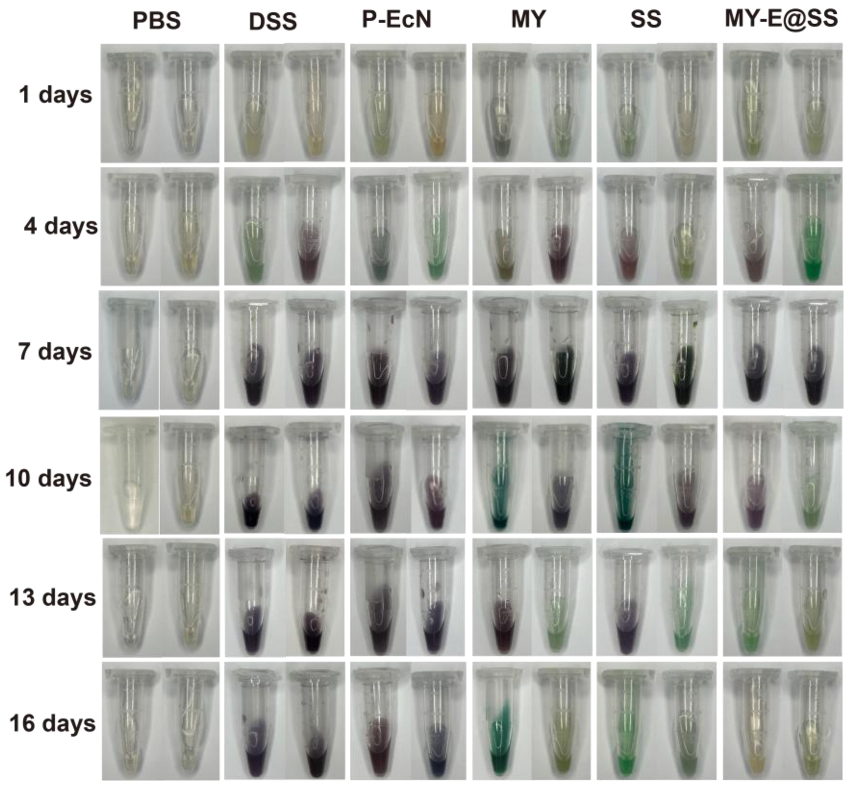

Figure S15. Fecal occult blood test results from different experimental groups. Representative images showing colorimetric changes in fecal samples, darker colors (black/brown) indicate more severe occult bleeding, while lighter colors (green/yellow) represent milder cases.

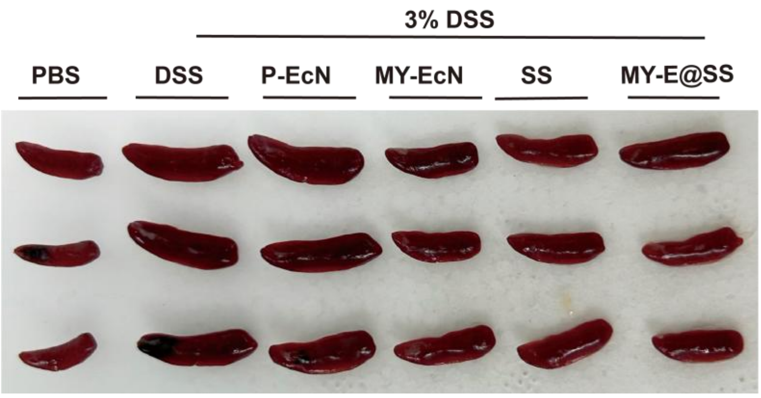

Figure S16. Representative images showing spleen size and morphology across experimental groups.

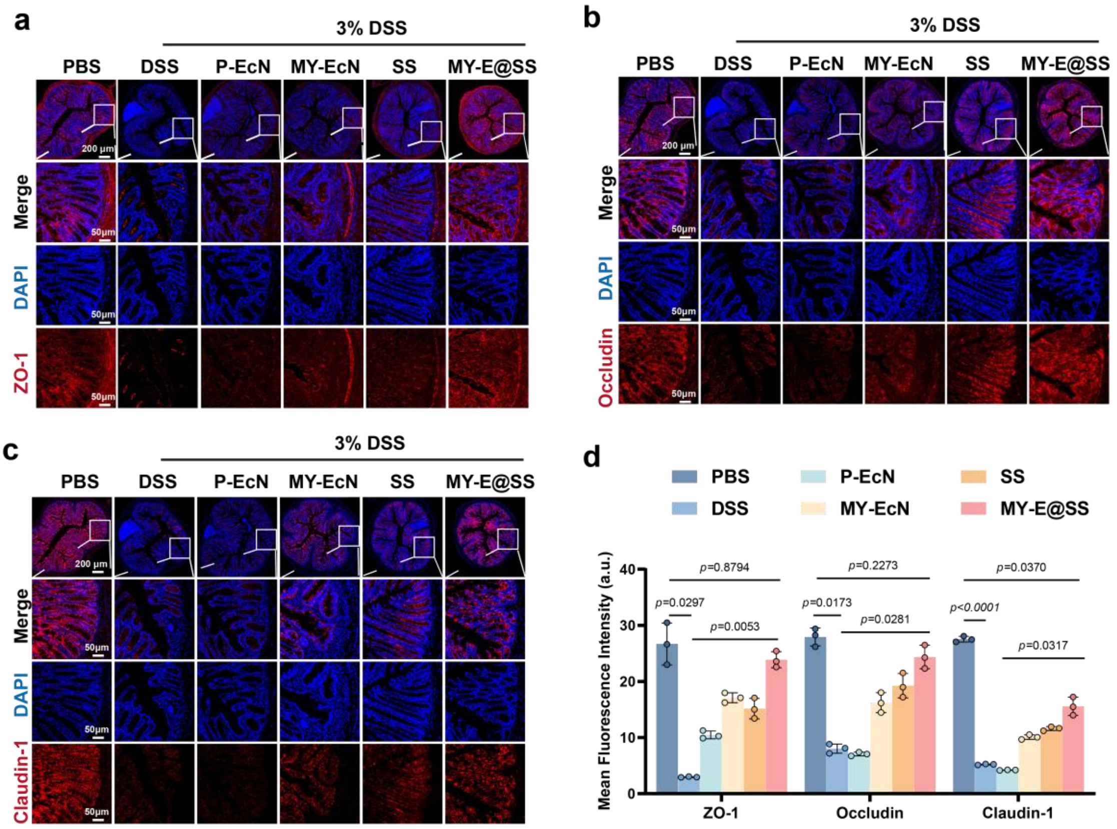

Figure S17. Assessment of tight junction protein expression based on immunofluorescence staining and quantitative analysis. (a-c) Representative immunofluorescence staining images of tight junction proteins ZO-1 (a), Occludin (b), and Claudin-1 (c) in colon tissues from each group of mice, respectively. (d) Quantitative analysis bar graph showing the relative expression levels of the three tight junction proteins. Data are presented as the mean ± SEM (n=3). Statistical analysis was performed with one way ANOVA with Tukey's test. Representative images are shown from one of three independent experiments. Source data are provided as a Source Data file.

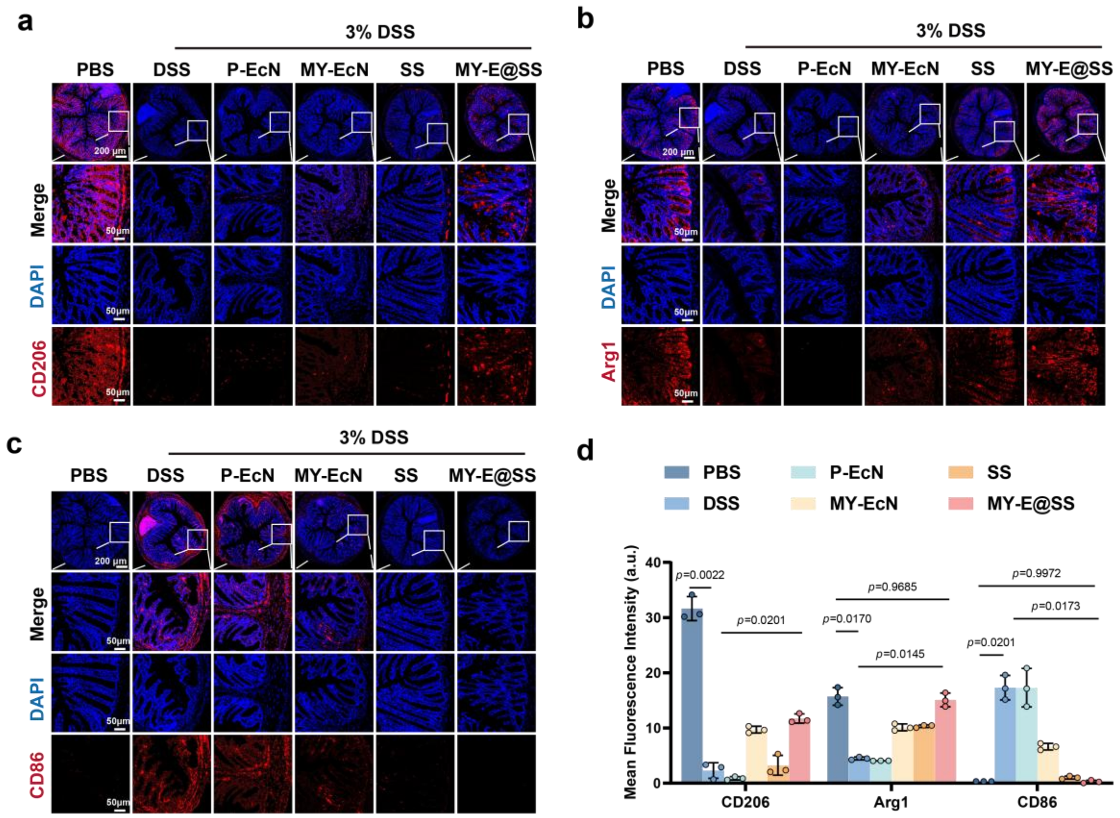

Figure S18. Immunophenotypic analysis of key immune cell subsets in colonic tissues following MY-E@SS treatment. (a–c) Representative immunofluorescence staining images of the M2 macrophage markers CD206 (a) and Arg1 (b), and the M1 macrophage marker CD86 (c) in colon tissues. (d) Quantitative analysis of the relative abundance of M1 ($CD86^{+}$) and M2 ($CD206^{+}/Arg1^{+}$) macrophage populations. Data are presented as the mean ± SEM (n=3). Statistical analysis was performed with one way ANOVA with Tukey's test. Representative images are shown from one of three independent experiments. Source data are provided as a Source Data file.

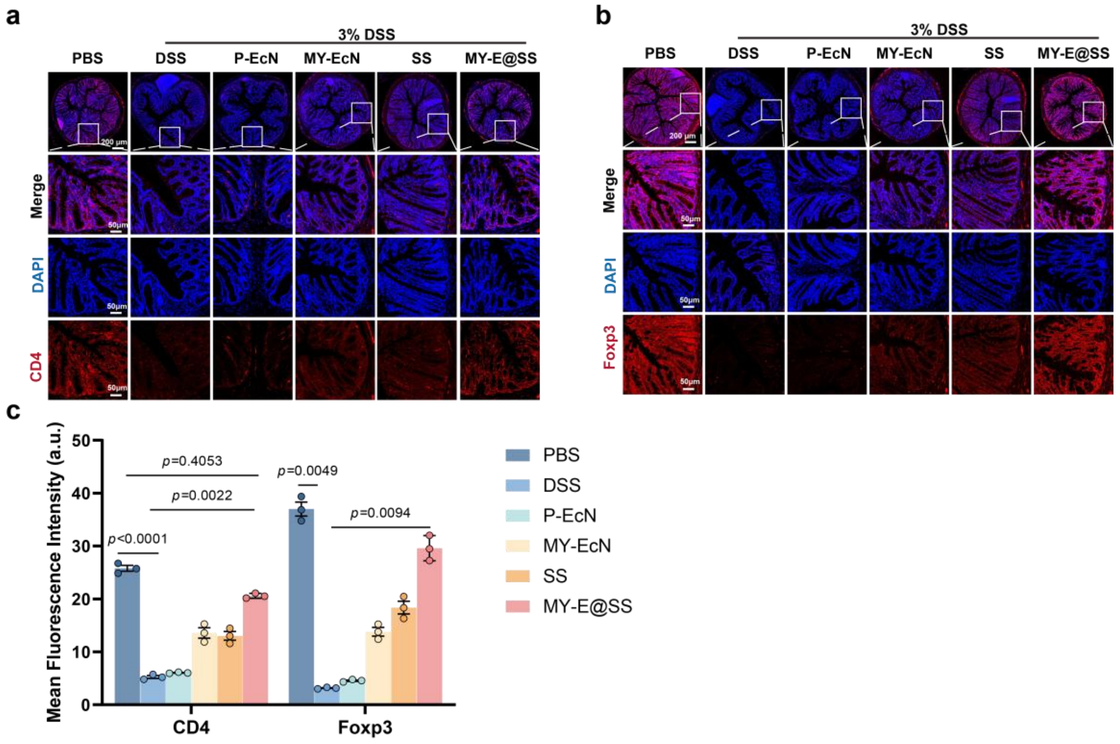

Figure S19. Dynamics of regulatory T cells (Tregs) in the colonic mucosa. (a) Representative immunofluorescence staining of CD4 in colon tissues. (b) Representative immunofluorescence staining of Foxp3 in colon tissues. (c) Quantitative analysis of the relative abundance and distribution of $Foxp3^{+}CD4^{+}$ regulatory T cells. Data are presented as the mean ± SEM (n=3). Statistical analysis was performed with one way ANOVA with Tukey's test. Representative images are shown from one of three independent experiments. Source data are provided as a Source Data file.

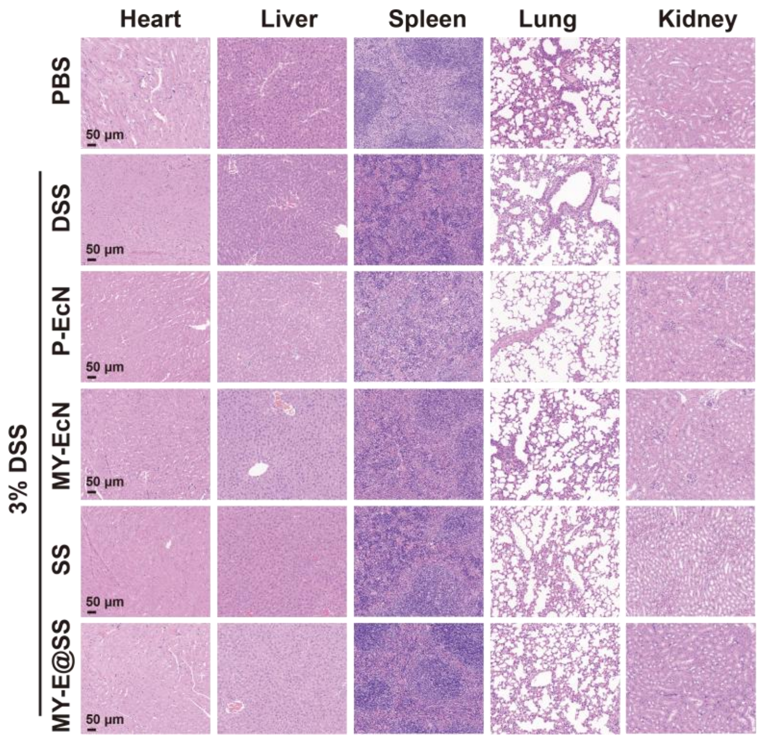

Figure S20. Hematoxylin and eosin (H&E)-stained sections of major organs demonstrating systemic biocompatibility. Representative images are shown from one of three independent experiments.

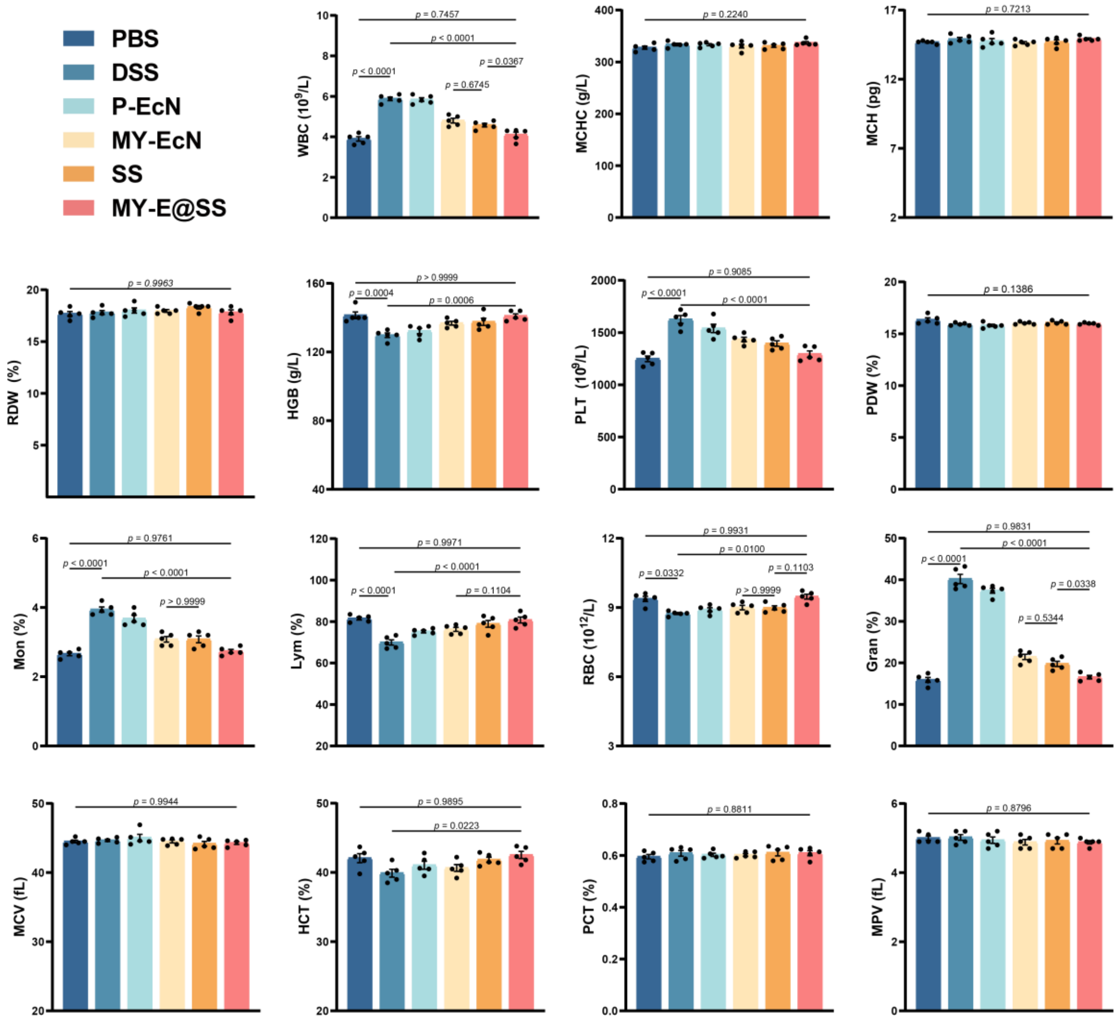

Figure S21. Hematological parameters in mice from different treatment groups. Data are presented as the mean ± SEM (n = 5 biologically independent experiments). Statistical analysis was performed with one way ANOVA with Tukey's test. Source data are provided as a Source Data file.

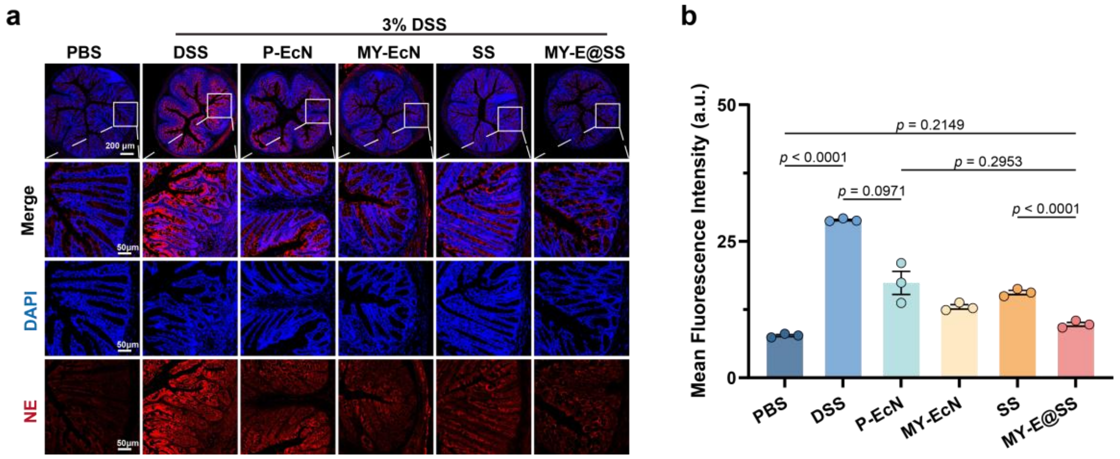

Figure S22. Quantitative analysis of neutrophil elastase (NE) expression and neutrophil infiltration at the inflammatory site. (a) Representative immunofluorescence staining images of NE in colon tissues from each group of mice. (b) Bar graph showing the relative expression levels of NE. Data are presented as the mean ± SEM (n=3). Statistical analysis was performed with one way ANOVA with Tukey's test. Representative images are shown from one of three independent experiments. Source data are provided as a Source Data file.

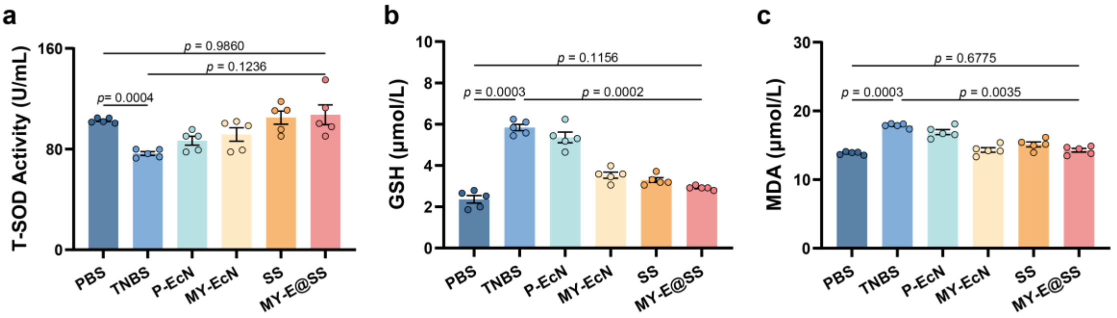

Figure S23. Changes in serum oxidative stress markers in IBD mice after different interventions. (a) Superoxide dismutase (SOD) activity, representing antioxidative enzyme capacity. (b) Glutathione (GSH) level, a major component of the non-enzymatic antioxidant system. (c) Malondialdehyde (MDA) content, reflecting the degree of lipid peroxidation. Data are presented as the mean ± SEM (n = 5 biologically independent experiments). Statistical analysis was performed with one way ANOVA with Tukey's test. Source data are provided as a Source Data file.

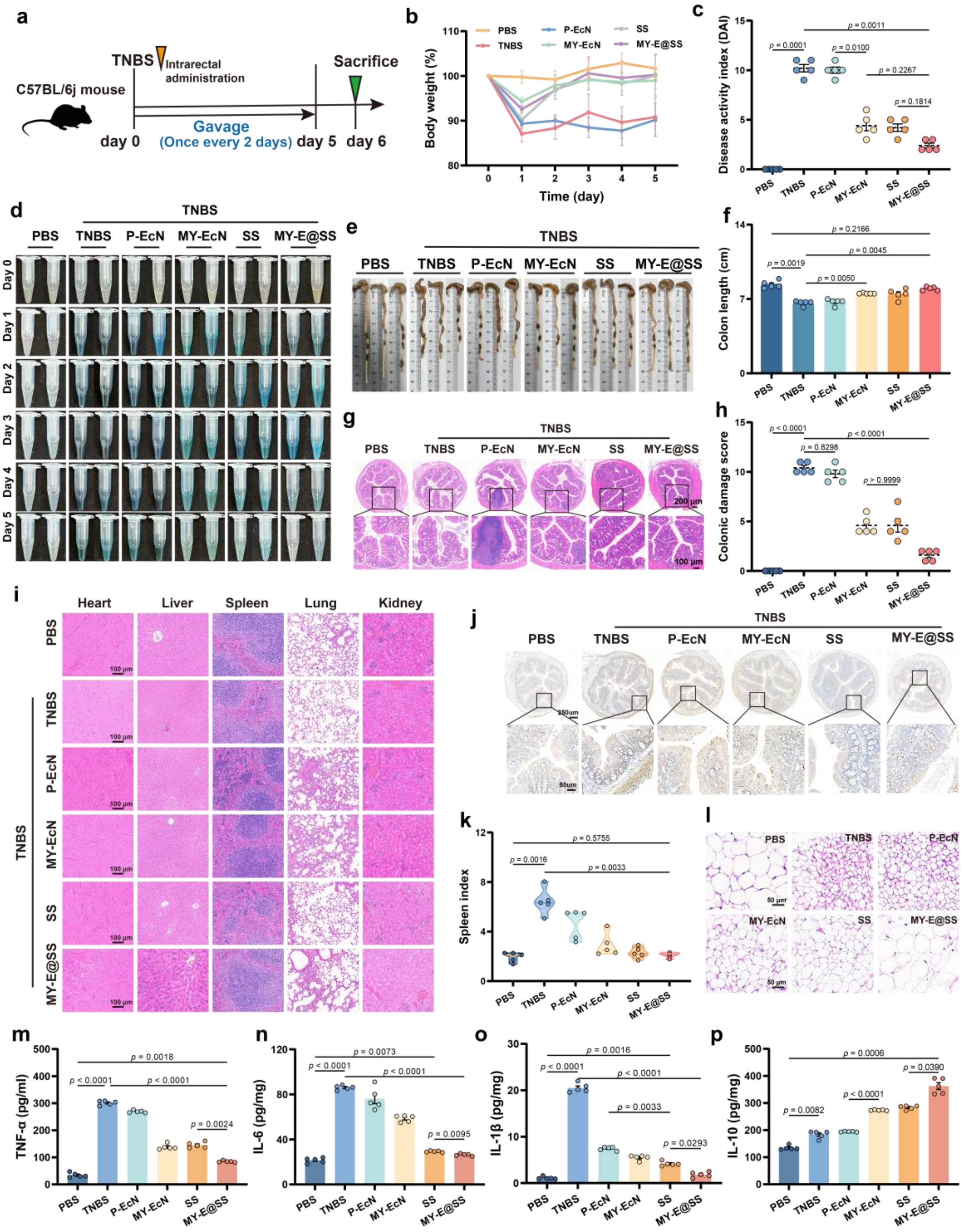

Figure S24. Therapeutic efficacy of MY-E@SS in a TNBS - induced IBD model. (a) Experimental timeline. IBD was induced in C57BL/6 mice by a single intrarectal administration of TNBS on day 0. From day 1 to day 5, mice received oral gavage of PBS, P-EcN, MY-EcN, SS, or MY-E@SS every other day. (b) Body weight changes and (c) disease activity index (DAI) throughout the experimental period. (d) Temporal changes in fecal occult blood (photographically documented). (e) Representative colon images, (f) colon lengths, and (g) representative H&E-

stained sections of colon tissues. (h) Colonic histopathological scores. (i) H&E-stained sections of major organs demonstrating systemic biocompatibility. (j) Myeloperoxidase (MPO) staining in colon sections. (k) Spleen index (spleen weight/body weight). (l) H&E-stained sections of adipose tissue. (m–p) Cytokine levels (TNF-α, IL-6, IL-1β, IL-10) in colon tissues measured by ELISA. All samples were collected on day 6. Data are presented as the mean ± SEM (n = 5 biologically independent experiments). Statistical analysis was performed with one way ANOVA with Tukey's test. Representative images are shown from one of three independent experiments. Source data are provided as a Source Data file.

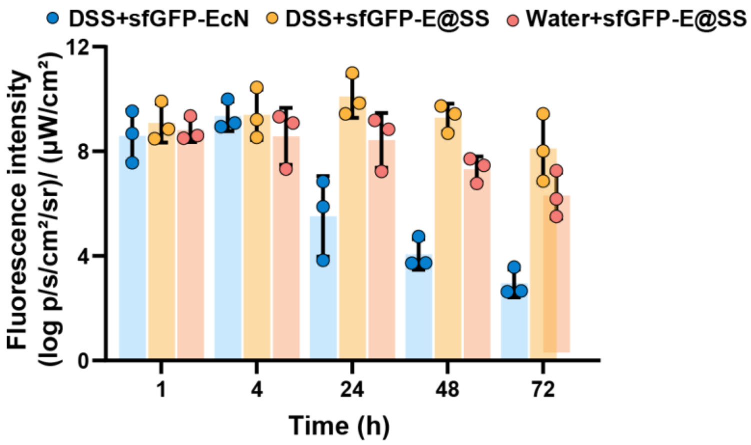

Figure S25. Quantitative analysis of intestinal GFP fluorescence intensity and in vivo tracking of probiotics. Data are presented as the mean ± SEM (n = 3 biologically independent experiments). Statistical analysis was performed with one way ANOVA with Tukey's test. Source data are provided as a Source Data file.

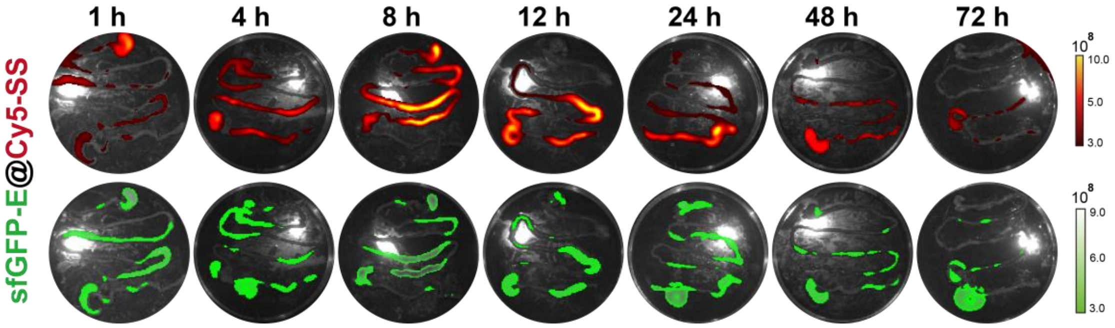

Figure S26. Spatiotemporal fluorescence imaging of sfGFP-E@Cy5-SS microcapsules in IBD model mice. SS shell: Cy5-labeled, red; Engineered bacteria: sfGFP-labeled, green. Representative images are shown from one of three independent experiments.

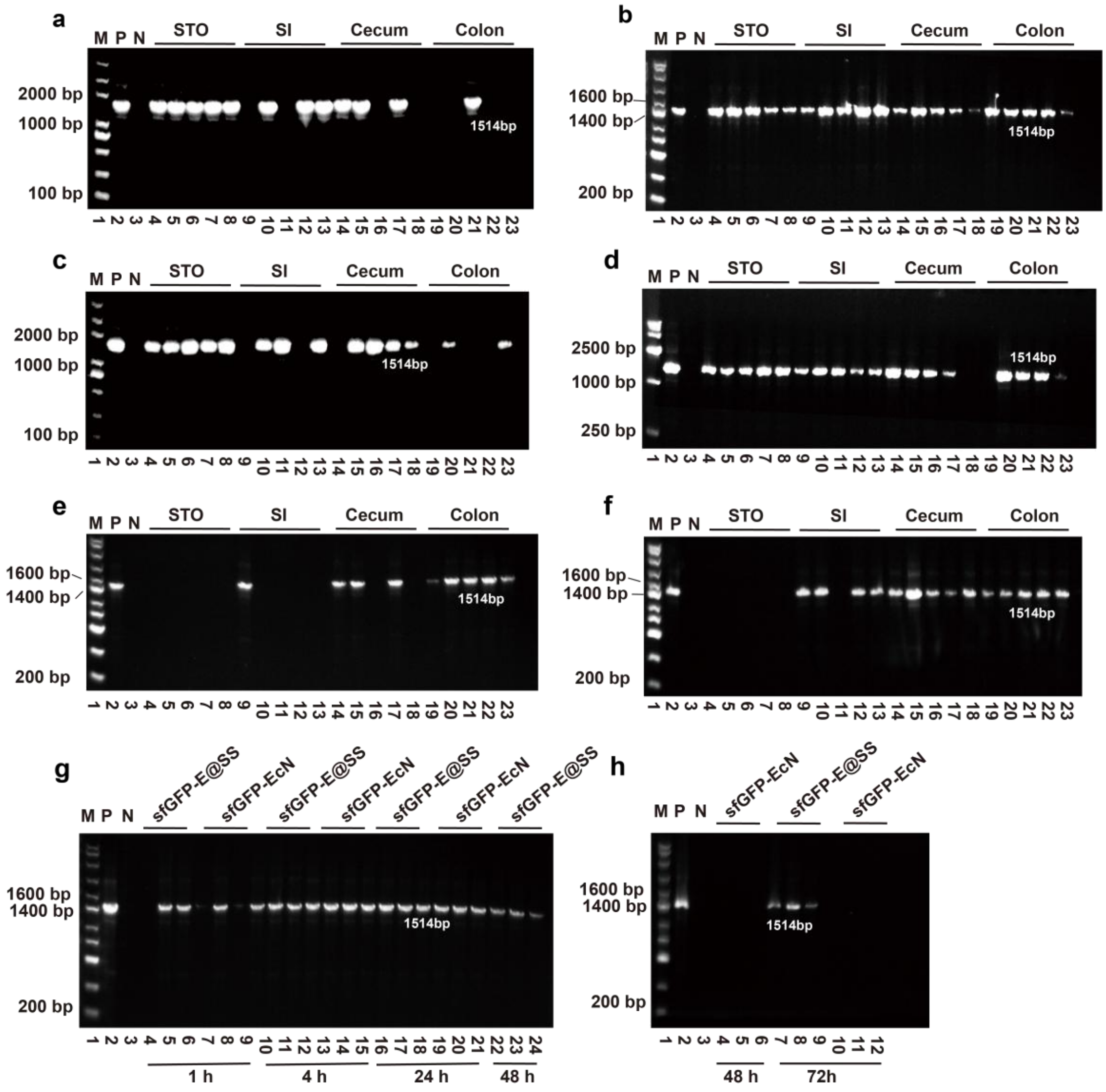

Figure S27. Temporal and spatial distribution of MY-EcN/MY-E@SS by PCR analysis. (a-f) Tissue homogenate samples (n = 5):(a) sfGFP-EcN at 1 h. (b) sfGFP-EcN at 4 h. (c) sfGFP-E@SS at 1 h. (d) sfGFP-E@SS at 4 h. (e) sfGFP-EcN at 24 h. (f) sfGFP-E@SS at 24 h. (g,h) Fecal samples: 1,4,24,48,72 h sfGFP-EcN / sfGFP-E@SS group PCR analysis of fecal homogenates. Data are presented as biologically independent experiments (n = 5 for a-f, n = 3 for g, h). Representative images are shown from one of three independent experiments.

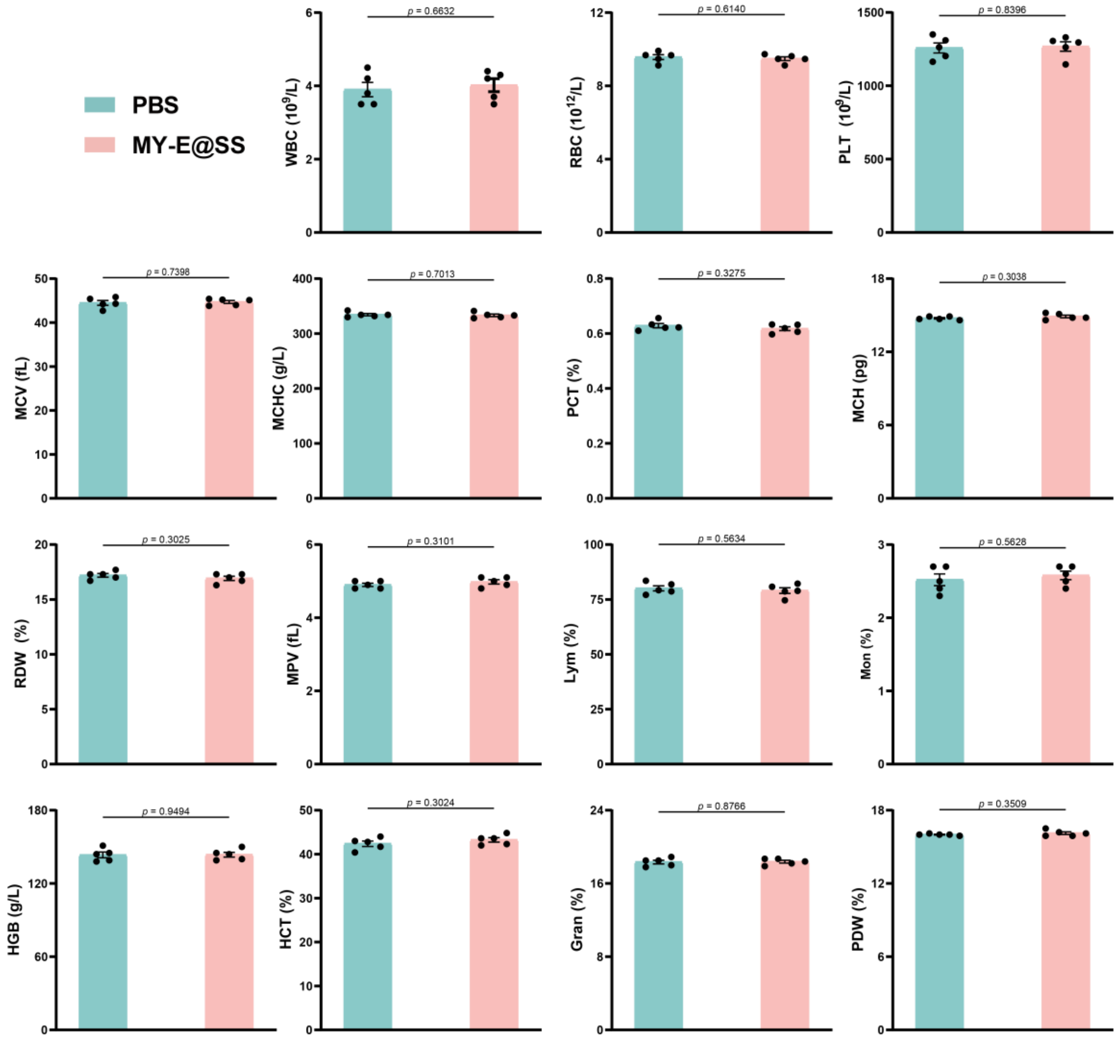

Figure S28. Hematological profiling of normal mice with/without MY-E@SS gavage. Data are presented as the mean ± SEM (n = 5 biologically independent experiments). Statistical analysis was performed with one way ANOVA with Tukey's test. Source data are provided as a Source Data file.

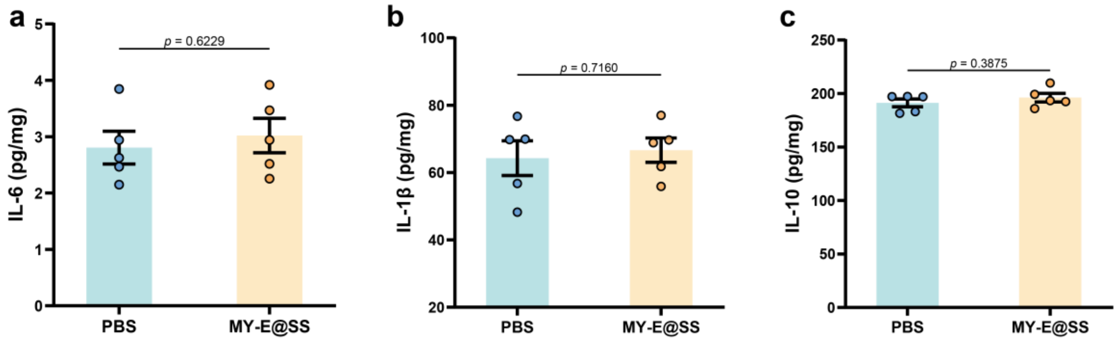

Figure S29. Quantitative analysis of pro- and anti-inflammatory cytokine levels in colon tissues at day 17 post-treatment. (a) IL-6, (b) IL-1β, (c) IL-10. Data are presented as the mean ± SEM (n = 5 biologically independent experiments). Statistical analysis was performed with one way ANOVA with Tukey's test. Source data are provided as a Source Data file.

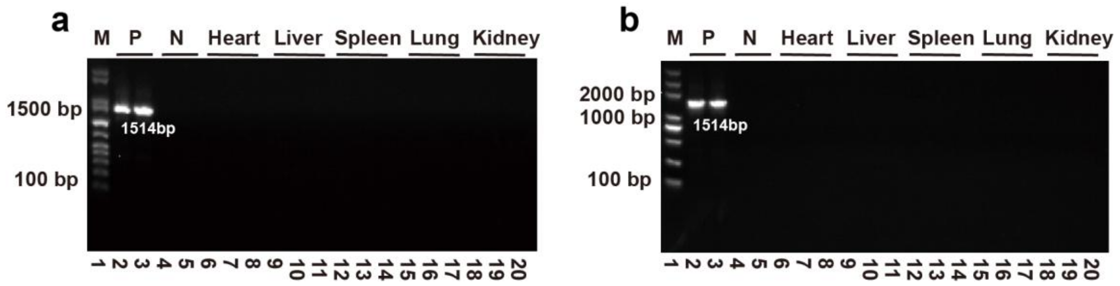

Figure S30. Assessment of bacterial translocation via sfGFP-tagged strain detection. (a) PCR amplification of sfGFP-E@SS in visceral organs. (b) PCR amplification of sfGFP-EcN in visceral organs. M: DNA marker; N: negative control; P: positive control. Representative images are shown from one of three independent experiments.

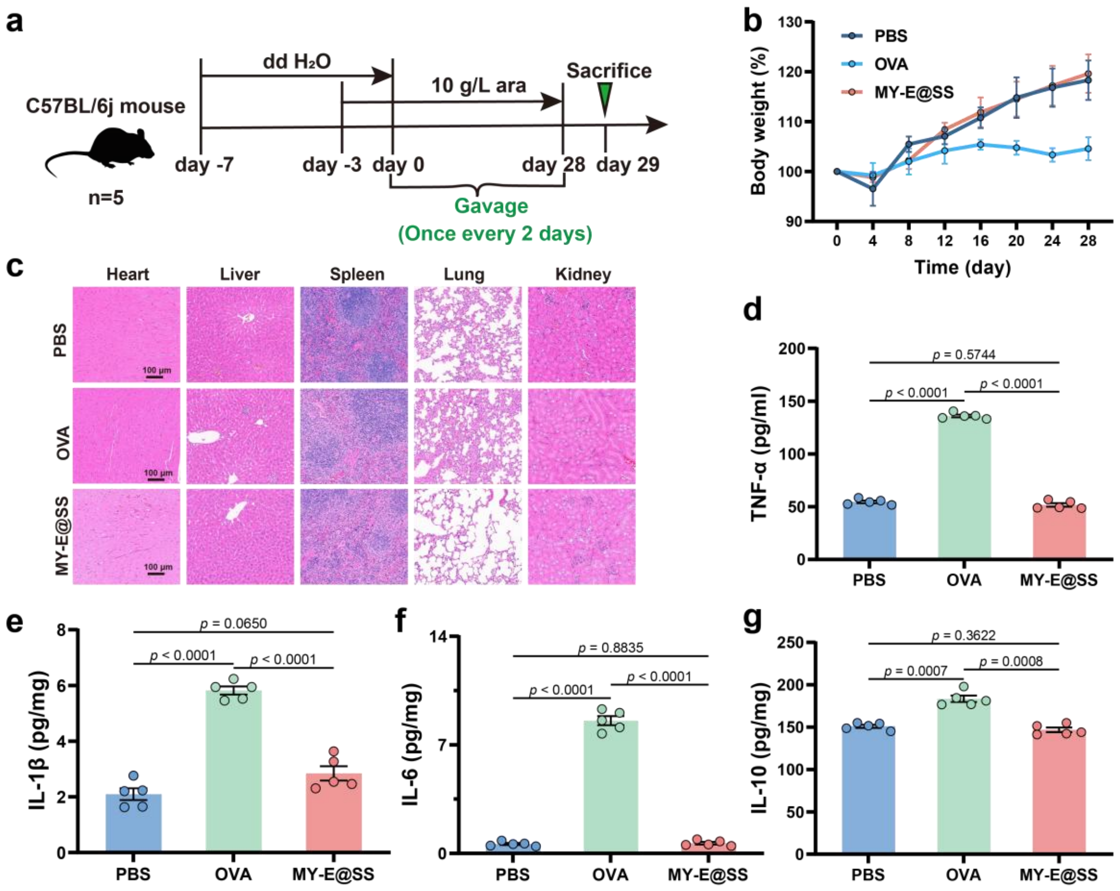

Figure S31. Extended (29-day) *in vivo* biocompatibility and immunogenicity assessment of MY-E@SS in healthy mice. (a) Schematic of experimental design. (b) Body weight changes over the 29-day period. (c) Representative H&E-stained histological images of major organs (heart, liver, spleen, lung, and kidney). (d–g) Systemic cytokine analysis: serum levels of (d) TNF-α, (e) IL-1β, (f) IL-6, and (g) IL-10. Data are presented as the mean ± SEM (n = 5 biologically independent experiments). Statistical analysis was performed with one way ANOVA with Tukey's test. Representative images are shown from one of three independent experiments. Source data are provided as a Source Data file.

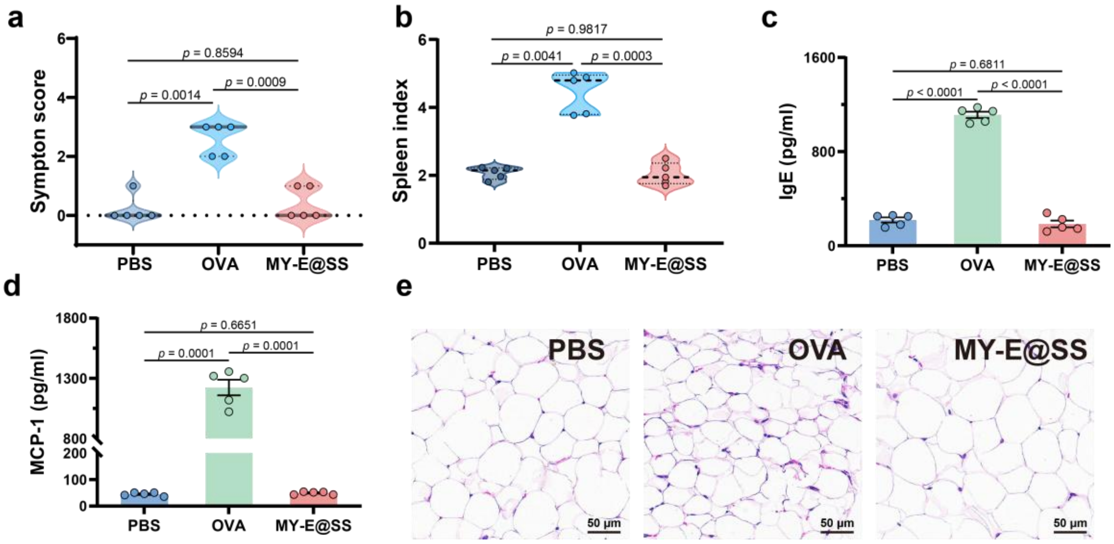

Figure S32. Assessment of immunogenicity and hypersensitivity potential after extended administration of MY-E@SS. (a) Behavioral monitoring record. (b) Spleen index. (c) Serum total IgE antibody levels measured by ELISA on day 29. (d) Serum levels of the allergy-related chemokine MCP-1. (e) H&E staining of adipose tissue. Data are presented as the mean ± SEM (n = 5 biologically independent experiments). Statistical analysis was performed with one way ANOVA with Tukey's test. Representative images are shown from one of three independent experiments. Source data are provided as a Source Data file.

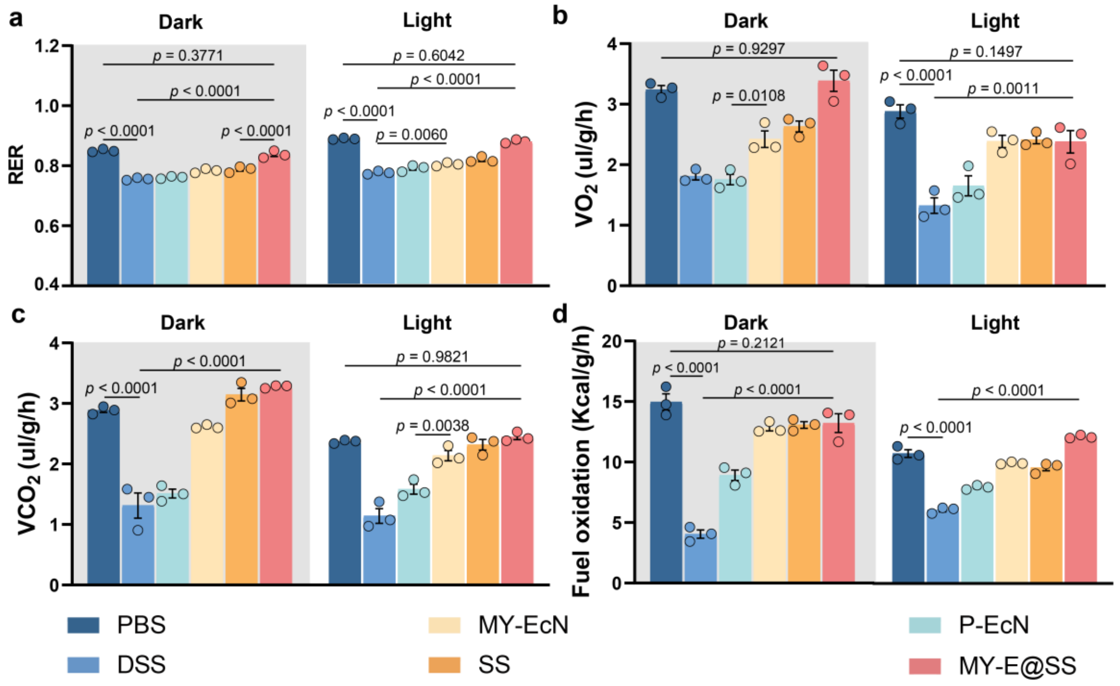

Figure S33. Quantitative analysis of the restorative effects of various treatment groups on key circadian parameters of respiratory metabolism. (a) Respiratory exchange ratio (RER). (b) Oxygen consumption (VO_2). (c) Carbon dioxide production (VCO_2). (d) Fuel oxidation rate. Data are presented as the mean ± SEM (n = 5 biologically independent experiments). Statistical analysis was performed with one way ANOVA with Tukey's test. Source data are provided as a Source Data file.

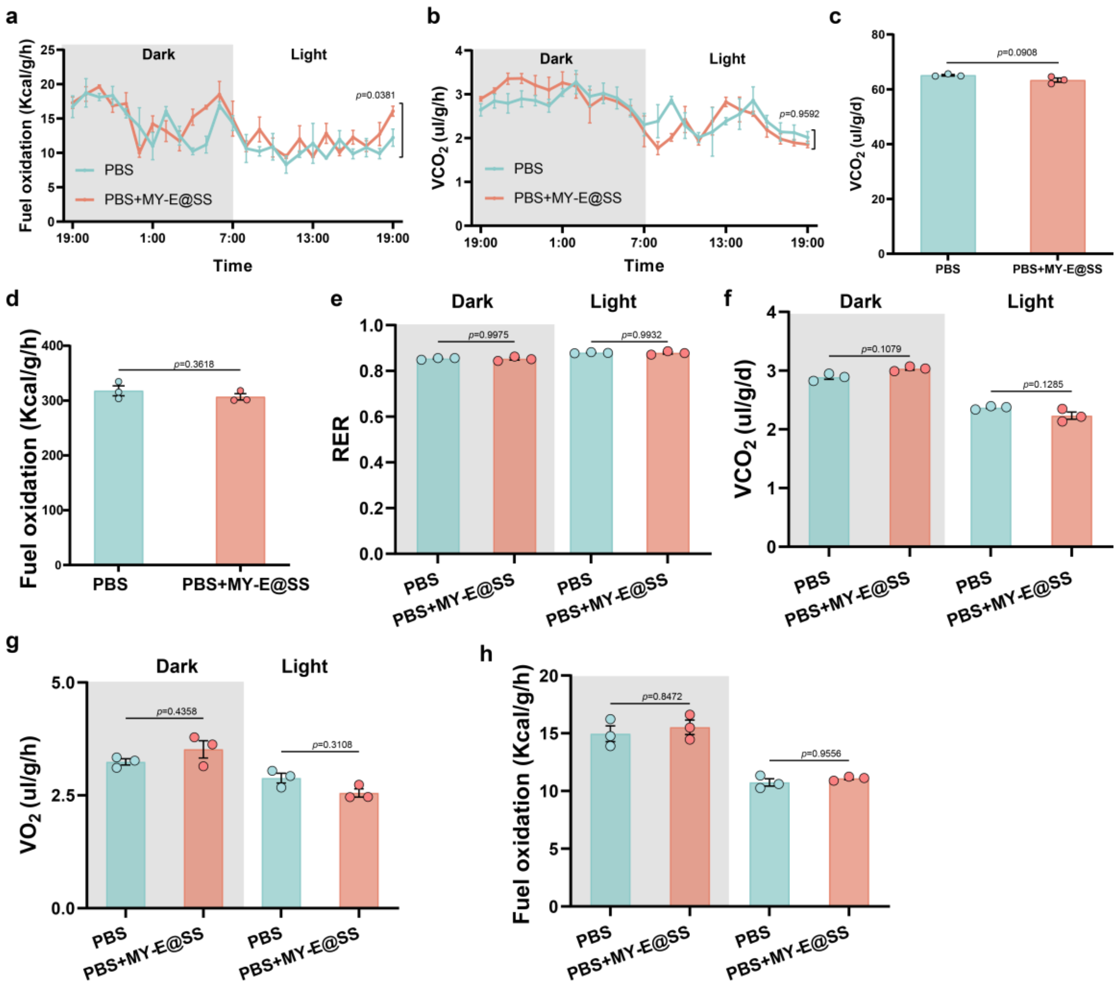

Figure S34. Evaluation of MY-E@SS biocompatibility in healthy mice based on respiratory metabolic parameters. (a,b) Dynamic changes in 24-hour fuel oxidation (a) and carbon dioxide production (VCO_2) (b) in healthy control mice after 16 days of MY-E@SS gavage. (c,d) Total 24-hour cumulative carbon dioxide production (c) and fuel oxidation (d) in healthy control mice after 16 days of gavage. (e-h) Total daytime and nighttime respiratory exchange ratio (RER), carbon dioxide production (VCO_2), oxygen consumption (VO_2), and fuel oxidation in healthy control mice after 16 days of gavage. Data are presented as the mean ± SEM (n = 3 biologically independent experiments). Statistical analysis were performed with one way ANOVA with Tukey's test (c-h), whereas an unpaired Student's t-test was used for panel a and b. Source data are provided in the Source Data file.

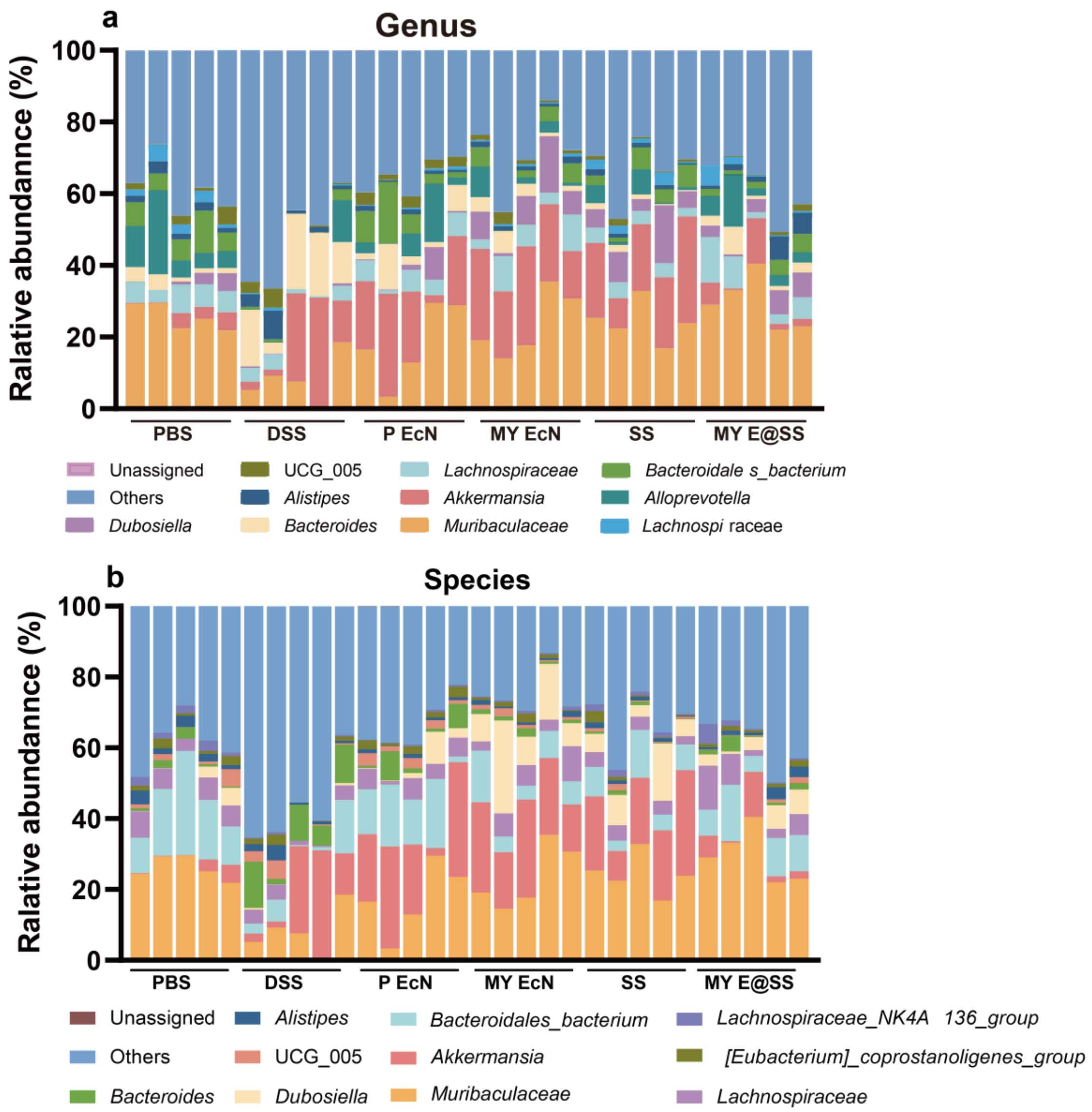

Figure S35. Compositional analysis of gut microbiota across treatment groups. (a) Genus-level stacked bar plot showing top 12 taxa by relative abundance. (b) Species-level stacked bar plot showing top 12 taxa by relative abundance. Data represent means from n=5 biologically independent samples. Source data are provided as a Source Data file.

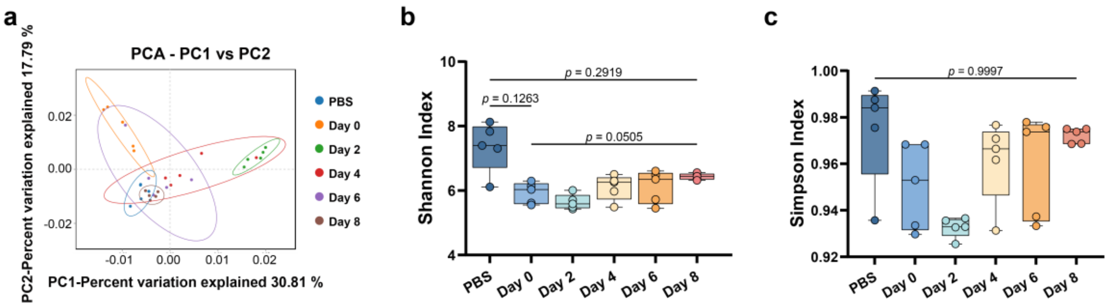

Figure S36. Compositional analysis of gut microbiota across treatment groups (a) β-diversity analysis (Principal component analysis (PCA)) illustrating the temporal shifts in community structure among different treatment groups. (b, c) α-diversity analysis using the Shannon index (b) and Simpson index (c), showing temporal trends in microbial richness and evenness within each treatment group. Data are presented as the mean ± SEM (b,c). Statistical analysis was performed with one way ANOVA with Tukey's test. (a-c) n =5 biologically independent experiments. Source data are provided as a Source Data file.

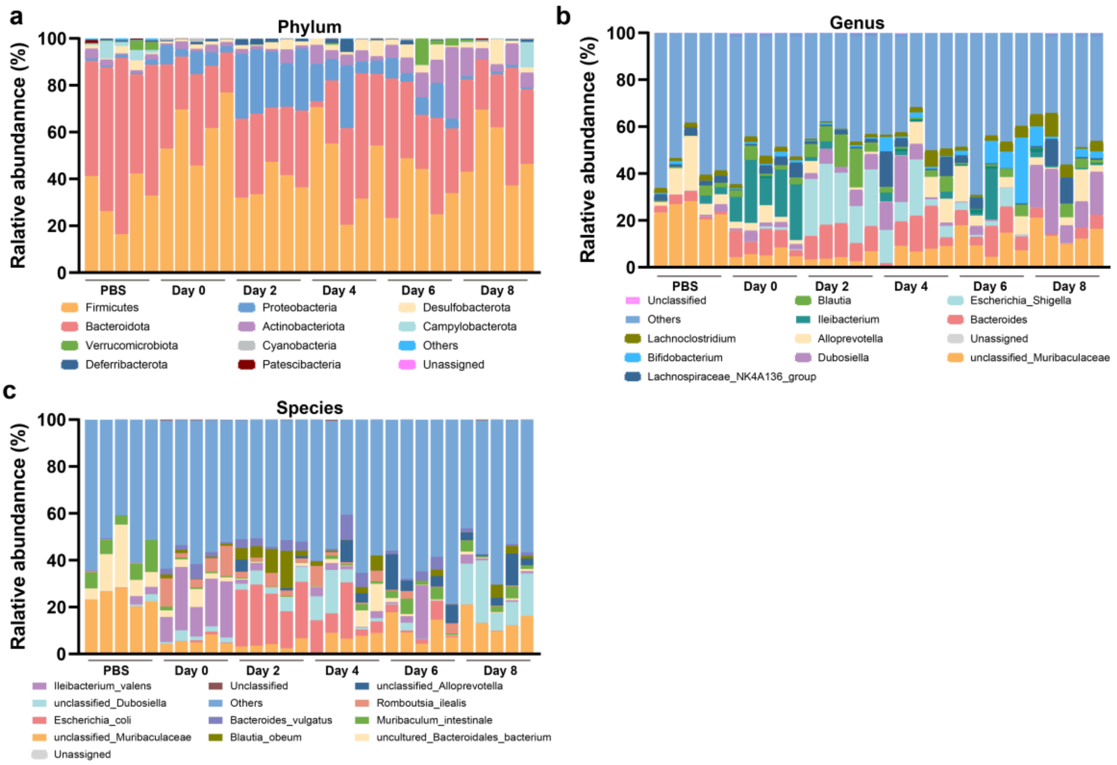

Figure S37. Microbial community composition at the phylum, genus, and species levels. Shown is the relative abundance of microbial communities across different treatment groups at various time points during the intervention, analyzed at the phylum (a), genus (b), and species (c) taxonomic ranks. n =5 biologically independent experiments. Source data are provided as a Source Data file.

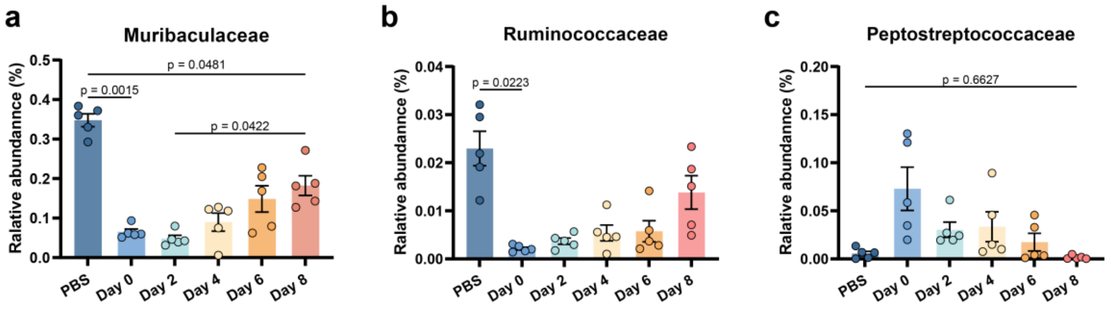

Figure S38. Temporal dynamics of typical probiotic and harmful bacterial abundances. (a-b) Post-intervention changes in the relative abundance of representative probiotic bacteria (Muribaculaceae (a), Ruminococcaceae (b)) in the MY-E@SS-treated group, showing a gradual increase over time toward the level observed in the control group. (c) Temporal trend in the abundance of a representative harmful bacterium (Peptostreptococcaceae) in the MY-E@SS-treated group, exhibiting a pattern opposite to that of the probiotics. Data are presented as the mean ± SEM (n = 5 biologically independent experiments). Statistical analysis was performed with one way ANOVA with Tukey's test. Source data are provided as a Source Data file.

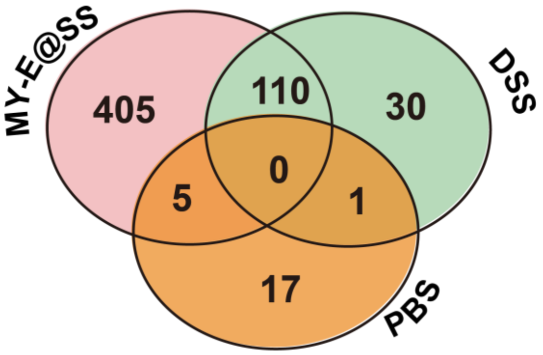

Figure S39. Venn diagram analysis of differentially expressed genes. n = 5 biologically independent experiments. Source data are provided as a Source Data file.

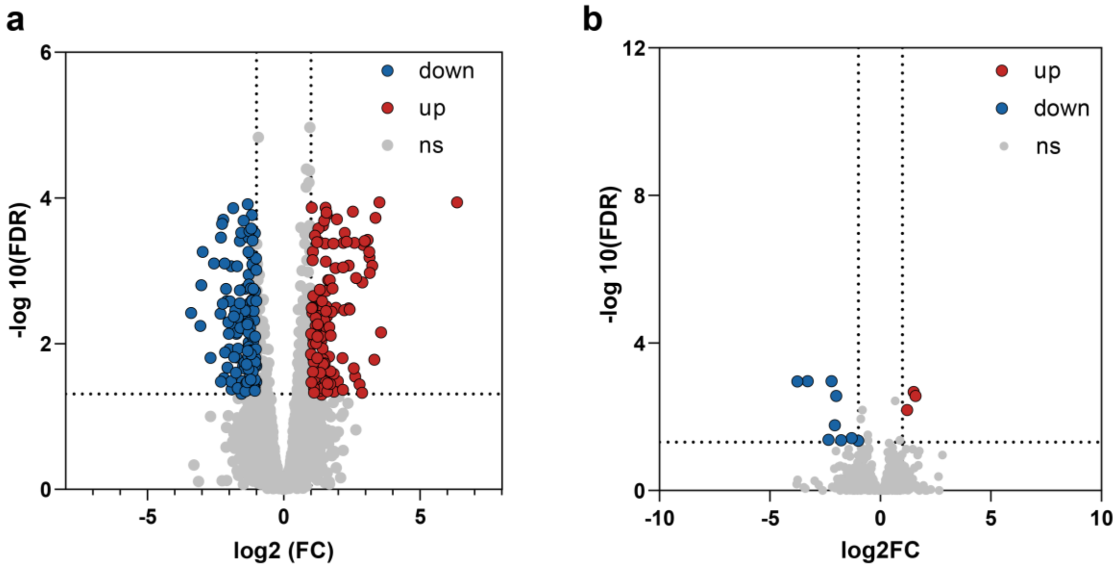

Figure S40. Volcano plots of differentially expressed genes. (a) PBS vs DSS group. (b) PBS vs MY-E@SS group. Red: significantly upregulated; blue: significantly downregulated. n = 3 biologically independent experiments. Source data are provided as a Source Data file.

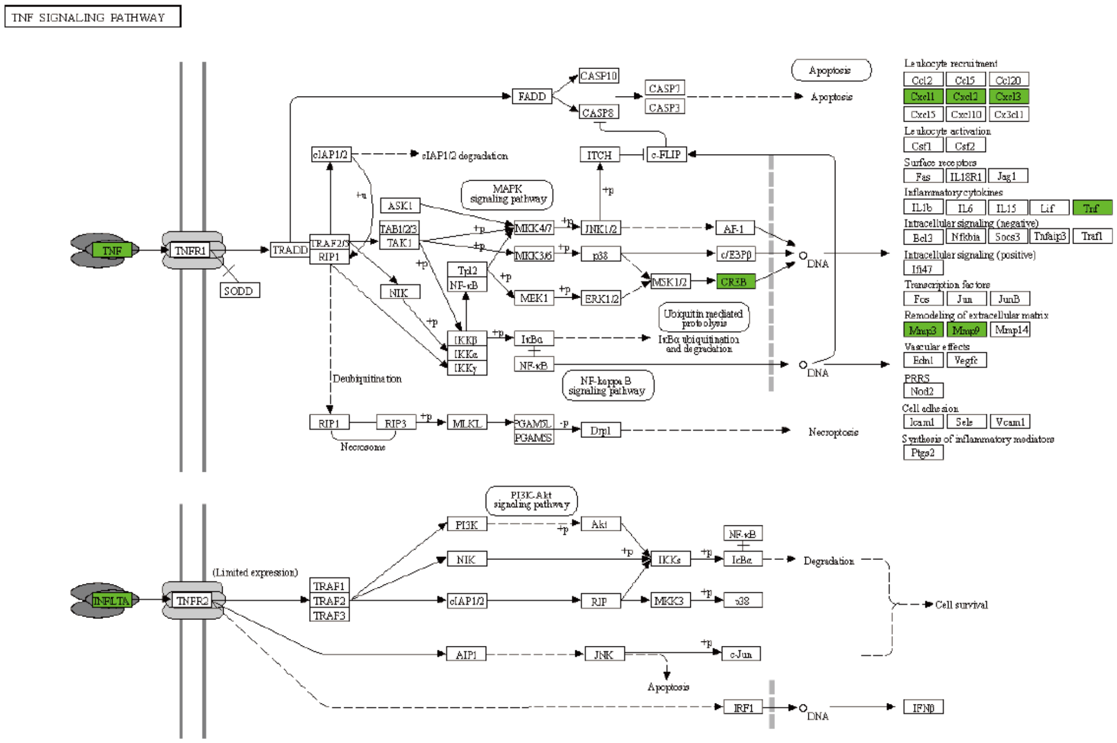

Figure S41. KEGG pathway map of the TNF signaling pathway in DSS and MY-E@SS groups. Green: Down-regulated genes; Red: Up-regulated genes. n = 3 biologically independent experiments. Data analysis was performed on BMKCloud.

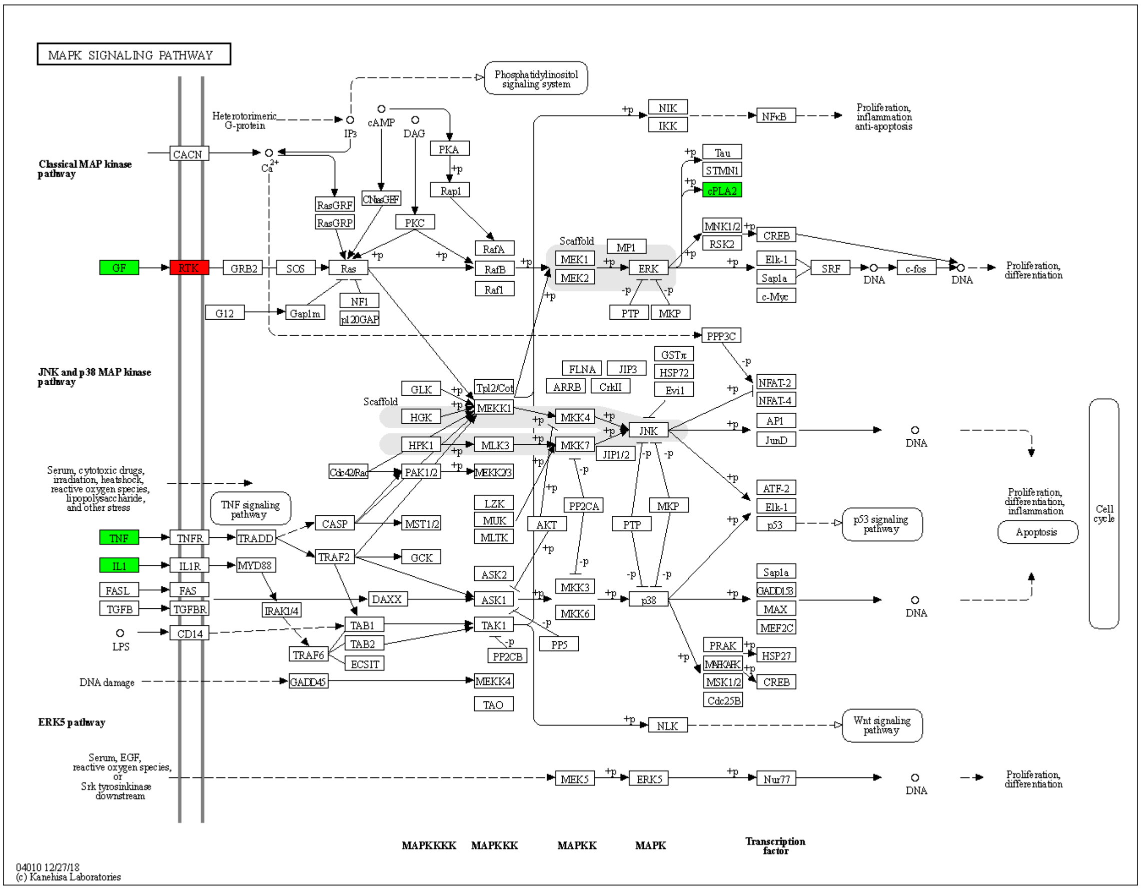

Figure S42. KEGG pathway map of the MAPK signaling pathway in DSS and MY-E@SS groups. Green: Down-regulated genes; Red: Up-regulated genes. n = 3 biologically independent experiments. Data analysis was performed on BMKCloud.

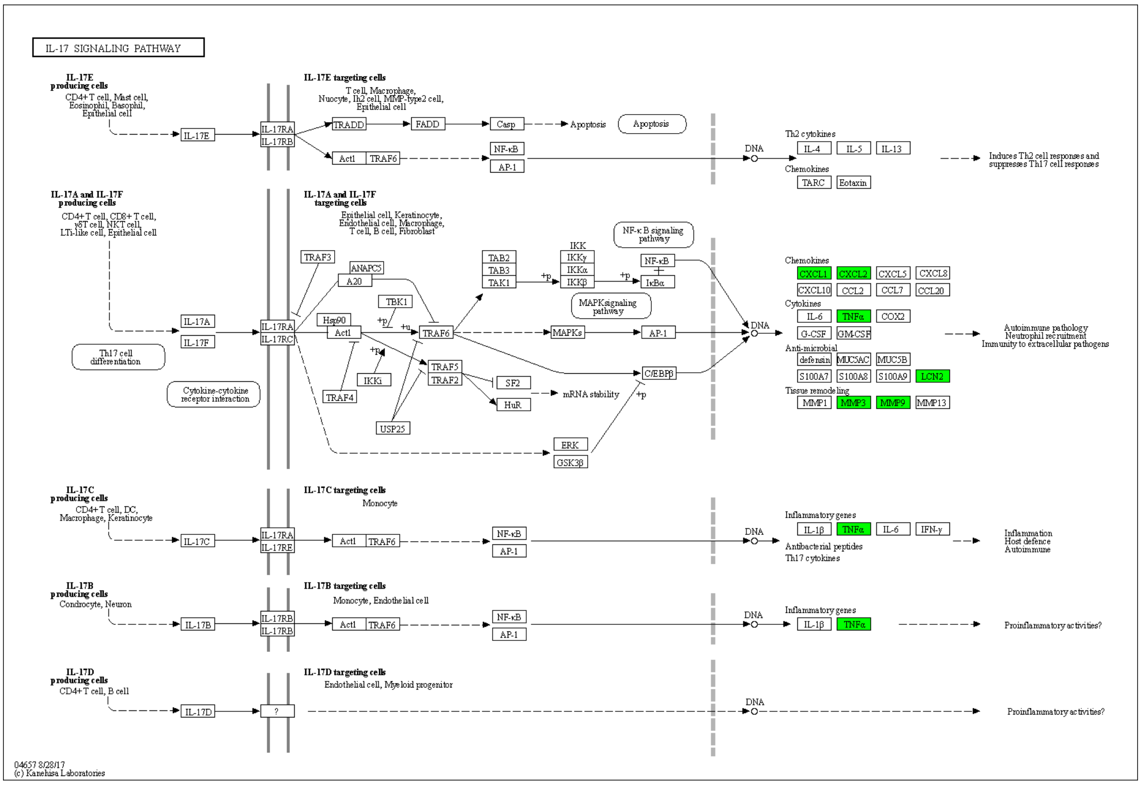

Figure S43. KEGG pathway map of the IL-17 signaling pathway in DSS and MY-E@SS groups. Green: Down-regulated genes; Red: Up-regulated genes. n = 3 biologically independent experiments. Data analysis was performed on BMKCloud.

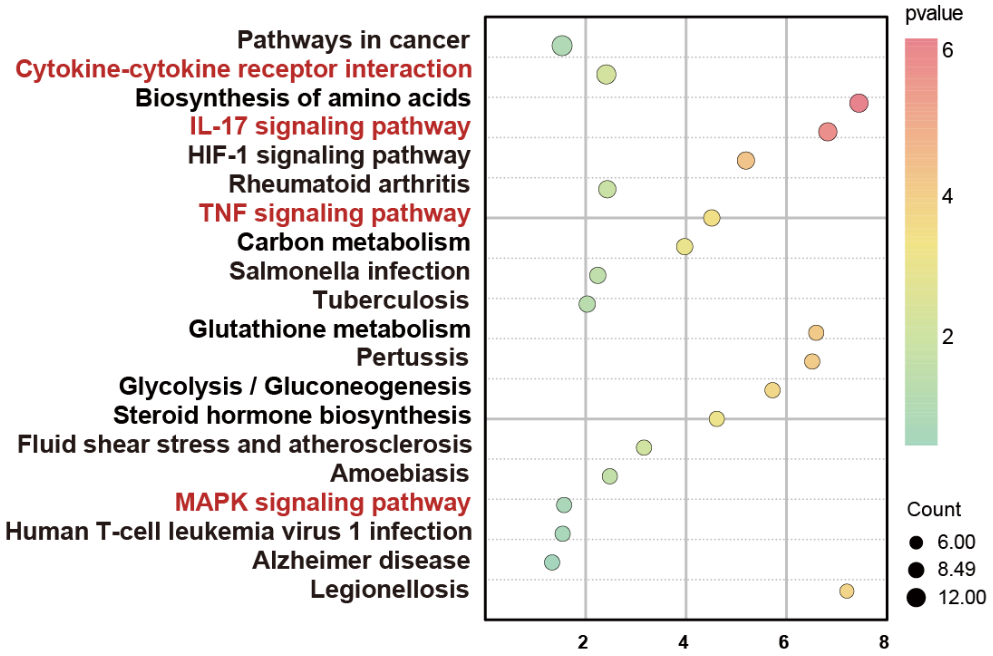

Figure S44. KEGG pathway enrichment of upregulated terms in DSS group (vs PBS). n = 3 biologically independent experiments. Source data are provided as a Source Data file.

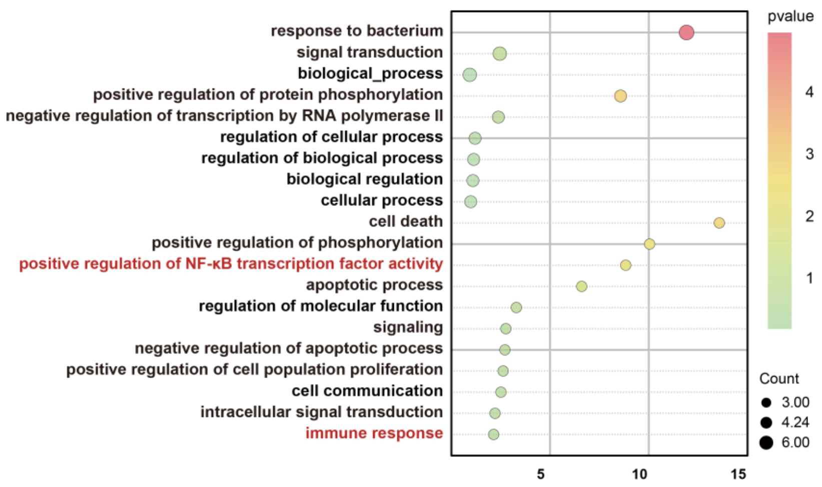

Figure S45. GO enrichment analysis of downregulated terms in MY-E@SS group (vs DSS). n = 3 biologically independent experiments. Source data are provided as a Source Data file.

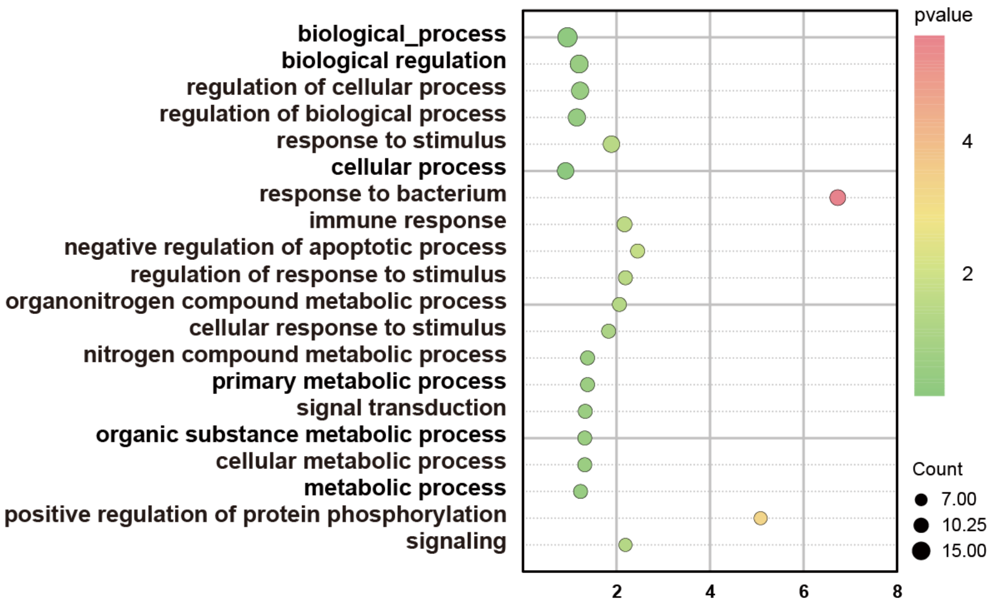

Figure S46. GO pathway enrichment of upregulated terms in DSS group (vs PBS). n = 3 biologically independent experiments. Source data are provided as a Source Data file.

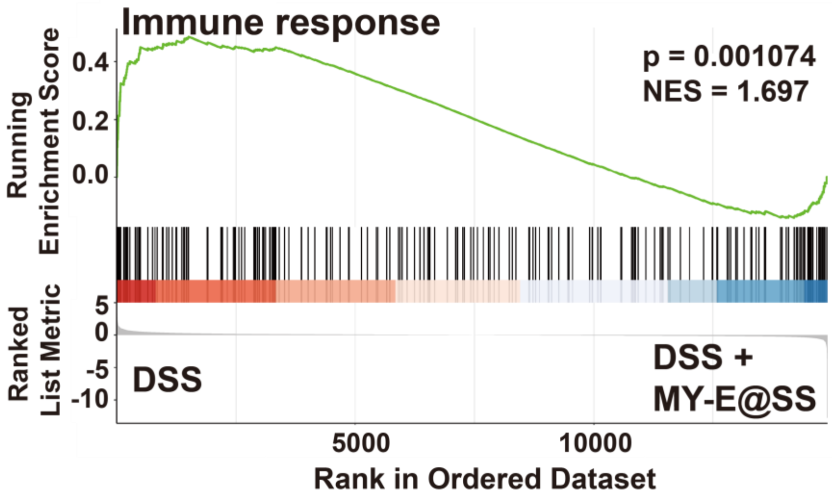

Figure S47. GSEA showing MY-E@SS-mediated regulate of immune response. n = 3 biologically independent experiments.

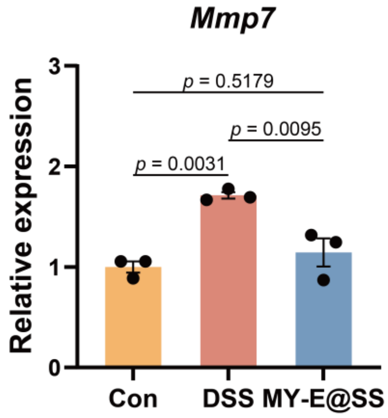

Figure S48. qRT-PCR analysis of *Mmp7* mRNA expression in mouse colon tissues from different groups. Data were normalized to GAPDH and expressed as mean ± SEM (n = 3 biologically independent experiments). Statistical analysis was performed with one way ANOVA with Tukey's test. Source data are provided as a Source Data file.

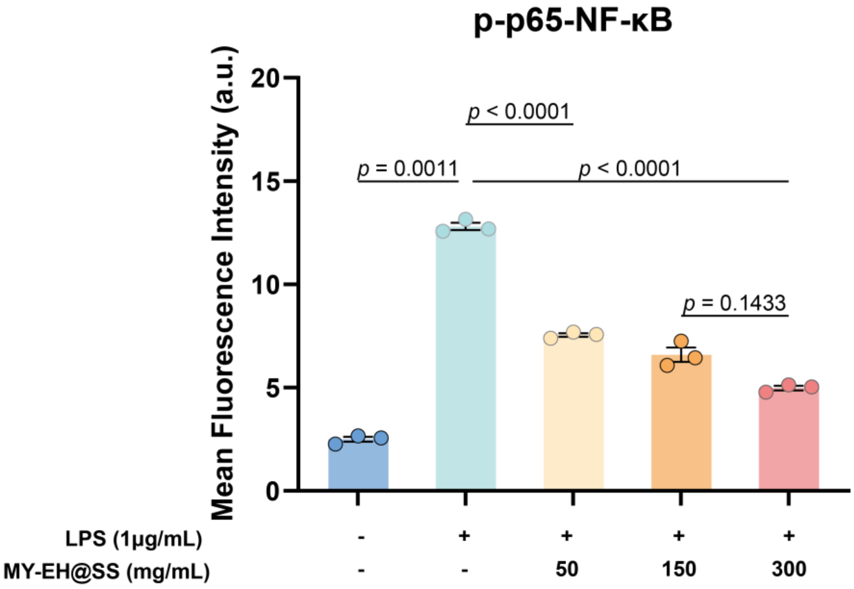

Figure S49. Quantification of p65 nuclear translocation from confocal images. Data were expressed as the mean ±SEM (n = 3 biologically independent experiments). Statistical analysis was performed with one way ANOVA with Tukey's test. Source data are provided as a Source Data file.

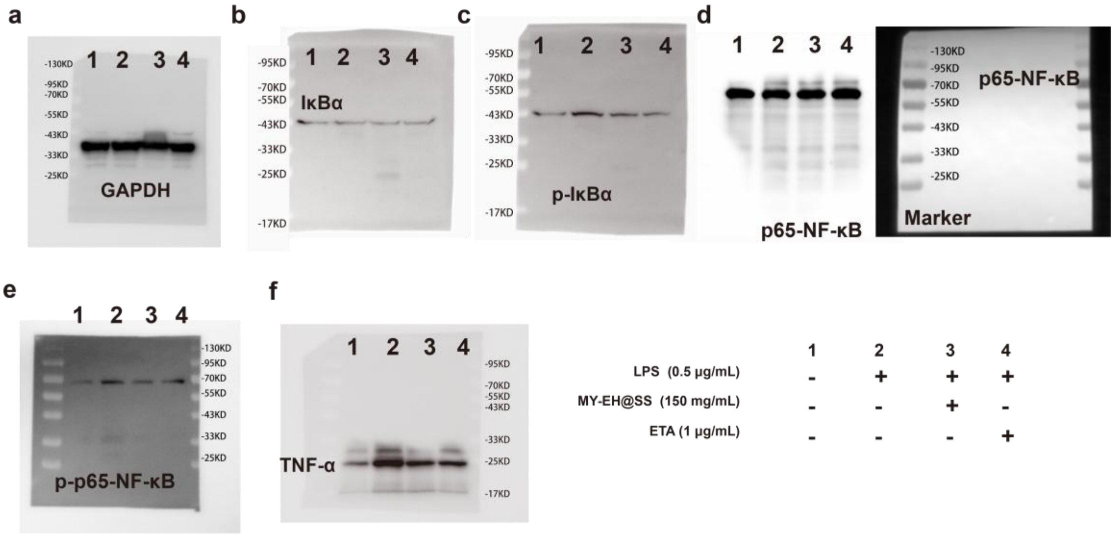

Figure S50. Inhibition of the TNF-α/NF-κB pathway by MY-EH@SS in RAW*264.7* macrophages. (a) GAPDH (loading control). (b) IκBα. (c) Phospho-IκBα (p-IκBα). (d) p65-NF-κB. To achieve optimal exposure for both the chemiluminescent signal and the visible molecular weight marker, the target protein signal and the marker were imaged separately from the same membrane. The two images are aligned according to the corresponding lanes. (e) Phospho-NF-κB p65 (p-p65-NF-κB). (f) TNF-α. Cells were treated as follows: Lane 1, control (Con); Lane 2, LPS; Lane 3, LPS + MY-EH@SS (150 μg/mL); Lane 4, LPS + Etanercept (ETA, positive control). Representative images are shown from one of three independent experiments. Source data are provided as a Source Data file.

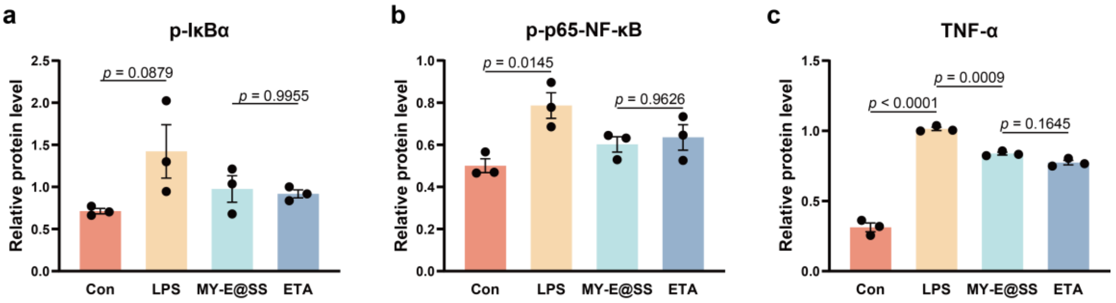

Figure S51. Quantitative analysis of TNF-α/NF-κB pathway inhibition by MY-E@SS in RAW*264.7* macrophages. (a) Quantitative analysis of p-IκBα protein levels normalized to GAPDH. (b) Quantitative analysis of p-p65-NF-κB protein levels normalized to GAPDH. (c) Quantitative analysis of TNF-α protein levels normalized to GAPDH. All samples were derived from the same experiment and the gels/blots were processed in parallel. Data are presented as the mean ± SEM (n = 3 biologically independent experiments). Statistical analysis was performed with one way ANOVA with Tukey's test. Source data are provided as a Source Data file.

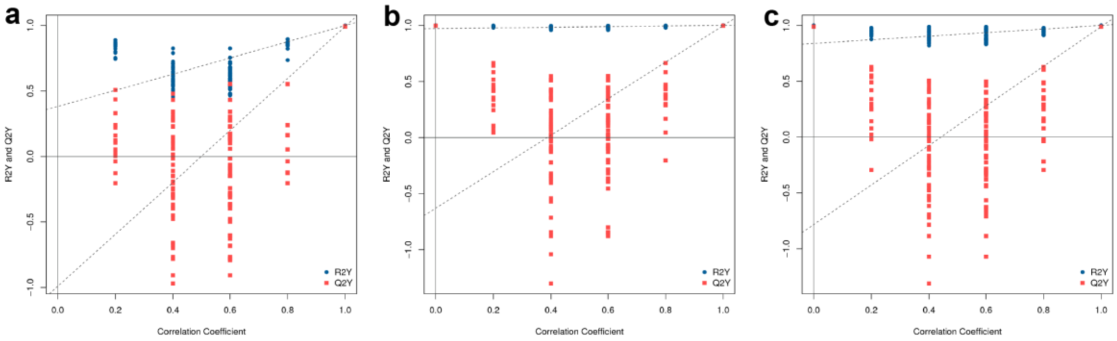

Figure S52. Permutation test of OPLS-DA model. (a) PBS group vs DSS group. (b) DSS group vs MY-E@SS group. (c) PBS group vs MY-E@SS group. Note: The x-axis shows correlation between permuted and original groupings. y-axis displays R^2Y (blue) and Q^2Y (red) values (x=1 represents original model). Dashed lines indicate regression fits. A positive Q^2Y slope indicates model validity. Decreasing R^2/Q^2 with permutation demonstrates model stability. n = 3 biologically independent experiments. Data analysis was performed on BMKCloud.

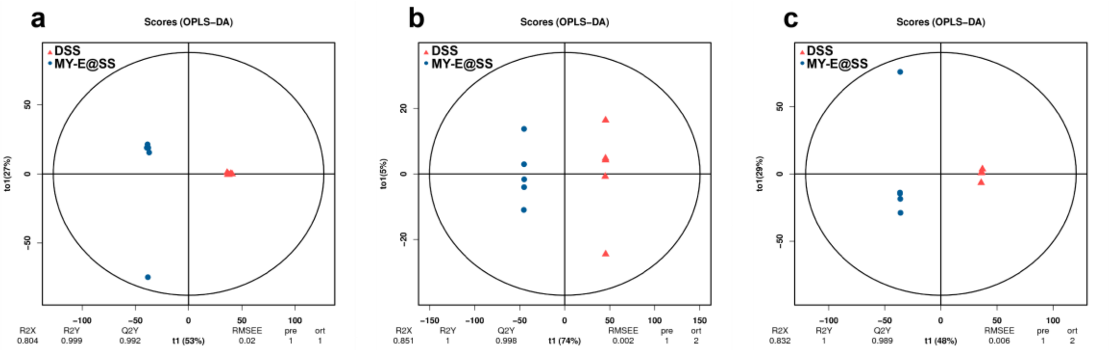

Figure S53. OPLS-DA score plots of untargeted metabolomics data. (a) PBS group vs DSS group. (b) DSS group vs MY-E@SS group. (c) PBS group vs MY-E@SS group. $Q^2Y>0.5$ indicates model validity. n = 3 biologically independent experiments. Data analysis was performed on BMKCloud.

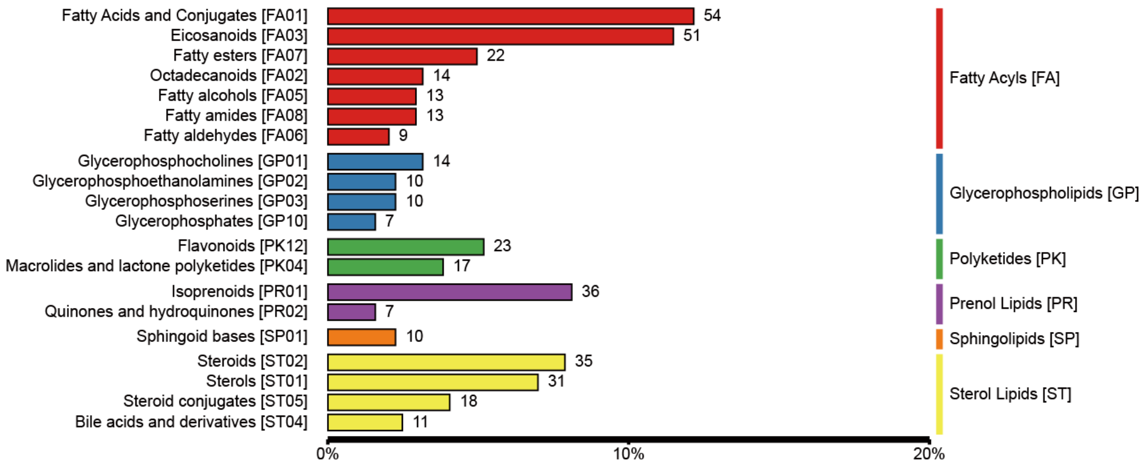

Figure S54. Top 20 lipid categories classified by LIPID MAPS database. Note: Lipid categories were ranked by annotation frequency in untargeted metabolomics data. Only the most abundantly annotated classes are shown. n = 3 biologically independent experiments. Data analysis was performed on BMKCloud. Source data are provided as a Source Data file.

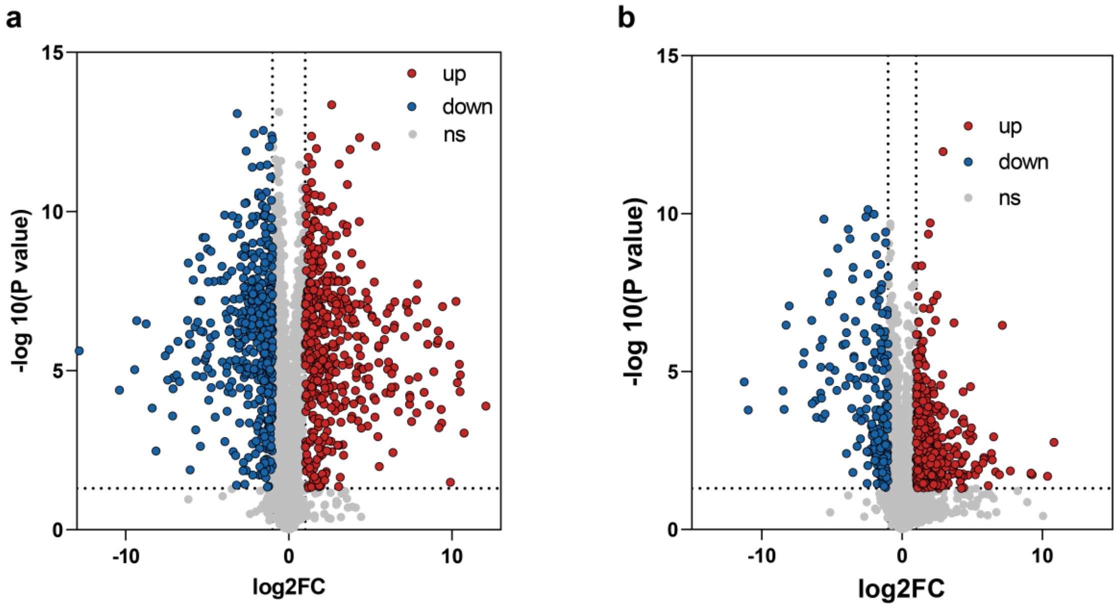

Figure S55. Volcano plots of DAMs. Volcano plots of DAMs between (a) PBS and DSS groups; (b) PBS and MY-E@SS. Two-sided Wald test from DESeq2 with Benjamini–Hochberg (BH)-adjusted p values false discovery rate (FDR). Red: significantly upregulated; blue: significantly downregulated. n = 3 biologically independent experiments. Source data are provided as a Source Data file.

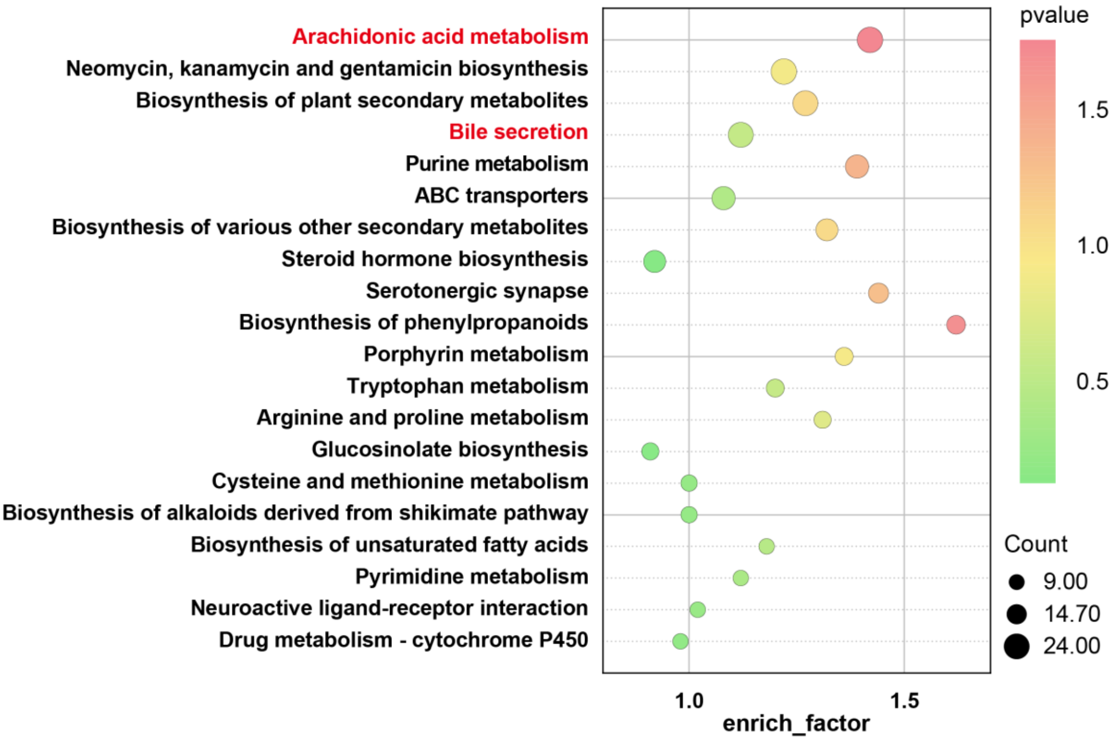

Figure S56. KEGG pathway enrichment analysis of upregulated metabolites in DSS group (vs PBS). Data are presented as the mean ± SEM. n = 3 biologically independent experiments. Source data are provided as a Source Data file.

Supplementary Tables

Table S1 The rusult of LC-MS/MS (MY-EH)

Peptide	Observed (m/z)	Calc. mass (M+H)	Scan Time	Intensity
D.[+42.011].RGDRGDAAPV.K	528.264	1055.523	11.7875	31232000
D.RGDRGDAAPV.K	507.253	1013.512	11.7924	637100000
D.RGDRGDAAPV.K	507.254	1013.512	11.8264	66073000
V.AAPVHHHHHH.-	590.287	1179.567	11.8824	877100000
V.AAPVHHHHHH.-	590.292	1179.567	11.9867	131950000
D.RGDRGDAAPV.K	1013.498	1013.512	12.2418	12412000
V.[+42.011].AAPVHHHHH.H	542.772	1084.518	12.2557	138280000
G.[+42.011].GSRGDRGDRGDAAPV.K	764.355	1527.726	12.6227	1546000000
V.[+42.011].KPV.A	385.244	385.245	12.9992	43999000
V.KPVAAPV.K	341.217	681.429	13.0114	26972000
D.[+42.011].RGDRGDAAPV.K	528.271	1055.523	14.3211	59243000
D.[+42.011].RGDRGDAAPV.K	528.271	1055.523	14.3726	59243000
V.AAPVHHHHH.H	521.764	1042.508	16.7199	645440000

Table S2. Positive-negative ideal solution

	Positive ideal solution (A+)	Negative ideal solution (A-)
H_2O_2 scavenging rate (0.2 mg/mL)	1.235	0.433
H_2O_2 scavenging rate (0.5 mg/mL)	0.207	0.142
H_2O_2 scavenging rate (1 mg/mL)	0.421	0.252
OH- scavenging rate (0.5 mg/mL)	0.174	0.123
OH- scavenging rate (1 mg/mL)	7.333	1.763
OH- scavenging rate (1.5 mg/mL)	6.146	2.106
O2•− scavenging rate (0.5 mg/mL)	0.507	0.267
O2•− scavenging rate (1 mg/mL)	1.733	0.673
O2•− scavenging rate (1.5 mg/mL)	0.439	0.288
ABTS scavenging rate (0.5 mg/mL)	5.459	1.081
ABTS scavenging rate (1 mg/mL)	0.443	0.334
ABTS scavenging rate (1.5 mg/mL)	0.668	0.482
DPPH scavenging rate (0.5 mg/mL)	0.541	0.336
DPPH scavenging rate (1 mg/mL)	0.87	0.594
DPPH scavenging rate (1.5 mg/mL)	1.071	0.712
PTIO scavenging rate (0.5 mg/mL)	0.854	0.468
PTIO scavenging rate (1 mg/mL)	0.664	0.394
PTIO scavenging rate (1.5 mg/mL)	2.368	1.041
Lipid peroxidation inhibition rate (0.5 mg/mL)	0.652	0.416
Lipid peroxidation inhibition rate (1 mg/mL)	0.538	0.336
Lipid peroxidation inhibition rate (1.5 mg/mL)	1.145	0.614
CAT activity (U/mg)	1.656	0.495
POD activity (U/mg)	0.054	0.007
SOD activity (U/mg)	3.075	1.863

Table S3. Summary of weight calculation results based on entropy weight TOPSIS method

	Information Entropye (e)	Information Utility (d)	Weight Coefficients (w)
H_2O_2 scavenging rate (0.2 mg/mL)	0.9492	0.0508	6.62%
H_2O_2 scavenging rate (0.5 mg/mL)	0.9925	0.0075	0.98%
H_2O_2 scavenging rate (1 mg/mL)	0.9867	0.0133	1.74%
OH- scavenging rate (0.5 mg/mL)	0.9933	0.0067	0.87%
OH- scavenging rate (1 mg/mL)	0.9063	0.0937	12.19%
OH- scavenging rate (1.5 mg/mL)	0.9523	0.0477	6.21%
O2•− scavenging rate (0.5 mg/mL)	0.9813	0.0187	2.43%
O2•− scavenging rate (1 mg/mL)	0.9577	0.0423	5.50%
O2•− scavenging rate (1.5 mg/mL)	0.9917	0.0083	1.09%
ABTS scavenging rate (0.5 mg/mL)	0.9113	0.0887	11.55%
ABTS scavenging rate (1 mg/mL)	0.995	0.005	0.65%
ABTS scavenging rate (1.5 mg/mL)	0.9936	0.0064	0.84%
DPPH scavenging rate (0.5 mg/mL)	0.988	0.012	1.56%
DPPH scavenging rate (1 mg/mL)	0.9909	0.0091	1.18%
DPPH scavenging rate (1.5 mg/mL)	0.9911	0.0089	1.16%
PTIO scavenging rate (0.5 mg/mL)	0.9817	0.0183	2.39%
PTIO scavenging rate (1 mg/mL)	0.9882	0.0118	1.54%
PTIO scavenging rate (1.5 mg/mL)	0.971	0.029	3.77%
Lipid peroxidation inhibition rate (0.5 mg/mL)	0.9874	0.0126	1.64%
Lipid peroxidation inhibition rate (1 mg/mL)	0.9899	0.0101	1.32%
Lipid peroxidation inhibition rate (1.5 mg/mL)	0.9828	0.0172	2.23%
CAT activity (U/mg)	0.946	0.054	7.03%
POD activity (U/mg)	0.816	0.184	23.94%
SOD activity (U/mg)	0.988	0.012	1.57%

Table S4. TOPSIS evaluation results

	Positive ideal solution distance (D+)	Negative ideal solution distance (D-)	Relative closeness values (C)	Ranking results
**MY-E@S0**	6.259	4.375	0.411	4
**MY-E@S1**	4.685	5.147	0.523	2
**MY-E@S2**	2.671	7.408	0.735	1
**MY-E@S3**	6.956	4.964	0.416	3

Table S5. Disease activity index (DAI) parameters and their associated scoring

Score	Weight loss (%)	Stool consistency	Blood in stool
0	None	Normal	Normal
1	1-5	Slight loose stool	Small presence of blood
2	5-10	loose stool	Significant presence of blood
3	10-15	Diarrhea	Gross blood
4	>15		

Note: This table is reprinted from [1].

Table S6. Histological grading scheme for DSS colitis

**Colonic epithelial damage**		**Inflammatory cell infiltration**	
Score	Description	**Mucosa**	
0	Normal	Score	Description
1	Hyperproliferation, irregular crypts, and goblet cell loss	0	Normal
2	Mild to moderate crypt loss (10-50%)	1	Mild
3	Severe crypt loss (50-90%)	2	Modest
4	Complete crypt loss, surfave epitheliun intact	3	Severe
5	Small-to mediun-sized ulcers (<10 crypt widths)	**Submucosa**	
6	Large ulcers (≥ crypt widths)	Score	Description
		0	Normal
		1	Mild to modest
		2	Severe
		**Muscle/serosa**	
		Score	Description
		0	Normal
		1	Moderate to severe

Note: This table is reprinted from [2].

Table S7. PCR primer sequences in this study

Organism	Target genes	Sequences (5' to 3')
Escherichia coli	*pBAD33*	Forward: TGCGTCTTTTACTGGCTCTTCTCGC<br>Reverse: GACCGCTTCTGCGTTCTGATTT
Escherichia coli	*sfGFP-Ptd103luxI*	Forward: GGTCAACAGAGTGCTCCCA<br>Reverse: CGAATGGGCTGACCGCTTCC
Mus musculus	*β-actin*	Forward: TGTCCACCTTCCAGCAGATGT<br>Reverse: AGCTCAGTAACAGTCCGCCTAGA
Mus musculus	*iNOS*	Forward: GAGCAACTACTGCTGGTGGT<br>Reverse: CGATGTCATGAGCAAAGGCG
Mus musculus	*TNF-α*	Forward: GATCGGTCCCAAAGGGATG<br>Reverse: CCACTTGGTGGTTTGTGAGTG
Mus musculus	*IL-1β*	Forward: ATGAAAGACGGCACACCCAC<br>Reverse: GCTTGTGCTCTGCTTGTGAG
Mus musculus	*IL-6*	Forward: ACAAAGCCAGAGTCCTTCAGAG<br>Reverse: GCCACTCCTTCTGTGACTCC
Mus musculus	*IL-10*	Forward: CAGAGAAGCATGGCCCAGAA<br>Reverse: GCTCCACTGCCTTGCTCTTA
Mus musculus	*GAPDH*	Forward: AGGTCGGTGTGAACGGATTTG<br>Reverse: GGGGTCGTTGATGGCAACA
Mus musculus	*Mmp7*	Forward: GTGAGGACGCAGGAGTGAAC<br>Reverse: AATGCCTGCAATGTCGTCCT
Mus musculus	*Mmp9*	Forward: CGACTTTTGTGGTCTTCCCCA<br>Reverse: GCGGTACAAGTATGCCTCTGC
Mus musculus	*p-p65-NF-κB*	Forward: ACCCTGACCATGGACGATCT<br>Reverse: TTGCTTCGGCTGTTCGATGA
Mus musculus	*NOS2*	Forward: CTGGGAGCGCTCTAGTGAAG<br>Reverse: CTCTCCACTGCCCCAGTTTT

References

[1] M. Li, N. Liu, J. Zhu, Y. Wu, L. Niu, Y. Liu, L. Chen, B. Bai, Y. Miao, Y. Yang and Q. Chen, Engineered probiotics with sustained release of interleukin-2 for the treatment of inflammatory bowel disease after oral delivery, Biomaterials, 309 (2024) https://doi.10.1016/j.biomaterials.2024.122584

[2] T. Powles, T. Csoszi, M. Ozguroglu, N. Matsubara, L. Geczi, S. Y. Cheng, Y. Fradet, S. Oudard, C. Vulsteke, R. Morales-Barrera, A. Flechon, S. Gunduz, Y. Loriot, A. Rodriguez-Vida, R. Mamtani, E. Y. Yu, K. Nam, K. Imai, B. H. Moreno and A. S. Alva, 1L pembrolizumab (pembro) versus chemotherapy (chemo) for choice-of-carboplatin patients with advanced urothelial carcinoma (UC) in KEYNOTE-361, Journal of Clinical Oncology, 39 (2021) https://doi.10.1200/JCO.2021.39.6_suppl.450
