# Supplementary material for: Bioinspired microcapsule reactor with engineered probiotics for IBD therapy
Source: Nat Commun. 2026 Jul 13;17:6095. doi: 10.1038/s41467-026-72027-1 (PMC13358097; doi:10.1038/s41467-026-72027-1)
Supplement: Supplementary file 2 — Description Of Additional Supplementary File [file 41467_2026_72027_MOESM2_ESM.pdf]

Description of Additional supplementary files

Supplementary Movie 1: Optical microscopy images of MY-E@SS microcapsules prepared via droplet microfluidics
