# Supplementary material for: Bioinspired microcapsule reactor with engineered probiotics for IBD therapy
Source: Nat Commun. 2026 Jul 13;17:6095. doi: 10.1038/s41467-026-72027-1 (PMC13358097; doi:10.1038/s41467-026-72027-1)
Supplement: Supplementary file 4 — Reporting summary [file 41467_2026_72027_MOESM4_ESM.pdf]

nature portfolio

Corresponding author(s): Guangfu Feng, Yuanchun Du

Last updated by author(s): 2026-3-23

Reporting Summary

Nature Portfolio wishes to improve the reproducibility of the work that we publish. This form provides structure for consistency and transparency in reporting. For further information on Nature Portfolio policies, see our Editorial Policies and the Editorial Policy Checklist.

Statistics

For all statistical analyses, confirm that the following items are present in the figure legend, table legend, main text, or Methods section.

n/a	Confirmed	
☐	☒	The exact sample size (*n*) for each experimental group/condition, given as a discrete number and unit of measurement
☐	☒	A statement on whether measurements were taken from distinct samples or whether the same sample was measured repeatedly
☐	☒	The statistical test(s) used AND whether they are one- or two-sided<br>*Only common tests should be described solely by name; describe more complex techniques in the Methods section.*
☐	☒	A description of all covariates tested
☐	☒	A description of any assumptions or corrections, such as tests of normality and adjustment for multiple comparisons
☐	☒	A full description of the statistical parameters including central tendency (e.g. means) or other basic estimates (e.g. regression coefficient) AND variation (e.g. standard deviation) or associated estimates of uncertainty (e.g. confidence intervals)
☐	☒	For null hypothesis testing, the test statistic (e.g. *F*, *t*, *r*) with confidence intervals, effect sizes, degrees of freedom and *P* value noted<br>*Give P values as exact values whenever suitable.*
☒	☐	For Bayesian analysis, information on the choice of priors and Markov chain Monte Carlo settings
☒	☐	For hierarchical and complex designs, identification of the appropriate level for tests and full reporting of outcomes
☒	☐	Estimates of effect sizes (e.g. Cohen's *d*, Pearson's *r*), indicating how they were calculated

Our web collection on statistics for biologists contains articles on many of the points above.

Software and code

Policy information about availability of computer code

Data collection	Bio-Rad microplate reader,Zeiss LSM880 laser scanning confocal microscope, Biosan RTS-1 Growth curve measuring instrument, Shanghai INESA Analysis 722N Visible spectrophotometer, Beijing Saizhi ChampGel 5000 Plus Gel imaging system, Shanghai Jingxin JXFSTPRP-48 tissue homogenizer, Shanghai Qinxiao ChemiScope 6100 Chemiluminescence imaging system, Hitachi, Japan CT15RE Desktop high-speed refrigerated centrifuge, Thermo Fisher Scientific Vanquish Neo/Orbitrap Exploris 480 Liquid chromatography-mass spectrometer ,LABCONCO 268410 Vacuum centrifuge concentrator
Data analysis	Statistical calculations were performed using GraphPad Prism 9.5.1 and Origin Pro 2021. Fluorescence images were analyzed by ImageJ 1.8.1. DNA sequence data were analyzed via SnapGene 6.2.2. 16S rRNA sequence data, transcriptomic data, and metabolomic data were analyzed via BMKcloud (https://international.biocloud.net/zh/user/login?redirect=%2Fzh%2Fdashboard).

For manuscripts utilizing custom algorithms or software that are central to the research but not yet described in published literature, software must be made available to editors and reviewers. We strongly encourage code deposition in a community repository (e.g. GitHub). See the Nature Portfolio guidelines for submitting code & software for further information.

Data

Policy information about availability of data

All manuscripts must include a data availability statement. This statement should provide the following information, where applicable:

- Accession codes, unique identifiers, or web links for publicly available datasets
- A description of any restrictions on data availability
- For clinical datasets or third party data, please ensure that the statement adheres to our policy

The DNA sequence generated in this study has been deposited in the National Center for Biotechnology Information (NCBI) GenBank database under accession number PP894312 (https://www.ncbi.nlm.nih.gov/nuccore/PP894312). The raw 16S rRNA gene sequences data generated in this study have been deposited in the NCBI SRA database under BioProject accession code PRJNA1439786 (https://www.ncbi.nlm.nih.gov/bioproject/PRJNA1439786). The 16S rDNA bacterial gene sequencing data generated in this study have been deposited in the NCBI SRA database under BioProject accession code PRJNA1440972 (https://www.ncbi.nlm.nih.gov/bioproject/PRJNA1440972). The mass spectrometry metabolomics data generated in this study have been deposited in the China National Center for Bioinformation (NGDC) under accession number PRJCA060448 (https://ngdc.cncb.ac.cn/bioproject/browse/PRJCA060448). All data from this study are fully available within the article, Supplementary Information or Source Data file. Any additional requests for information can be directed to, and will be fulfilled by, the corresponding authors. Source data are provided with this paper.

Research involving human participants, their data, or biological material

Policy information about studies with human participants or human data. See also policy information about sex, gender (identity/presentation), and sexual orientation and race, ethnicity and racism.

Reporting on sex and gender	N/A
Reporting on race, ethnicity, or other socially relevant groupings	N/A
Population characteristics	N/A
Recruitment	N/A
Ethics oversight	N/A

Note that full information on the approval of the study protocol must also be provided in the manuscript.

Field-specific reporting

Please select the one below that is the best fit for your research. If you are not sure, read the appropriate sections before making your selection.

☒ Life sciences ☐ Behavioural & social sciences ☐ Ecological, evolutionary & environmental sciences

For a reference copy of the document with all sections, see nature.com/documents/nr-reporting-summary-flat.pdf

Life sciences study design

All studies must disclose on these points even when the disclosure is negative.

Sample size	For all animal experiments, sample sizes were chosen based on prior experience with the murine colitis model and in accordance with the 3Rs (Replacement, Reduction, Refinement) principles of animal ethics regulation. No statistical method was used to predetermine sample size. Instead, sample sizes (n = 5 per group for in vivo experiments) were selected by referring to established protocols and previously published studies in related colitis models (J Neuroinflammation. 2021,18(1):153; Adv Sci. 2019, 6(18):1900610), which demonstrated that these sample sizes were sufficient to detect statistically significant differences between treatment groups with acceptable biological variability. For cellular experiments, three independent samples were performed per condition, which is a standard practice in the field and has been shown to provide adequate statistical power to detect consistent biological effects. All experiments contained at least three independent replicates, and the reproducibility of results across replicates confirms that the chosen sample sizes were sufficient to draw reliable conclusion
Data exclusions	No data was excluded in this study.
Replication	All attempts at replication were successful, and the results were consistent across all independent replicates.
Randomization	For the animal experiments, mice were randomly allocated into experimental groups using a random number generator before treatment initiation. This ensured that potential confounding factors such as body weight or age were evenly distributed across groups. For the in vitro cellular experiments, all cells used in each independent replicate were derived from the same batch and cultured under identical conditions. Therefore, randomization was not applicable, as all experimental units were homogeneous at the time of allocation. Covariates such as passage number and cell density were kept consistent across groups to control for variability
Blinding	Blinding was carried out during data collection and/or analysis for the animal and cell experiments. Specifically, group allocation was

Blinding	concealed by using coded samples during outcome assessment, and the investigator performing quantitative and statistical analyses was blinded to experimental group identity. However, a formal doubleblinding protocol (i.e., blinding during both the intervention phase and outcome assessment) was not implemented for the animal experiments because a single investigator was responsible for both administering treatments and collecting data, making complete blinding during the intervention phase unfeasible. Similarly, in the cellular experiments, blinding was applied during data acquisition and analysis, but the investigator conducting the cell culture and treatments was aware of group allocation due to practical constraints. All attempts at replication were successful, and the consistency of results across independent replicates supports the reliability of the findings.

Reporting for specific materials, systems and methods

We require information from authors about some types of materials, experimental systems and methods used in many studies. Here, indicate whether each material, system or method listed is relevant to your study. If you are not sure if a list item applies to your research, read the appropriate section before selecting a response.

Materials & experimental systems

n/a	Involved in the study
☐	☒ Antibodies
☒	☐ Eukaryotic cell lines
☒	☐ Palaeontology and archaeology
☐	☒ Animals and other organisms
☒	☐ Clinical data
☒	☐ Dual use research of concern
☒	☐ Plants

Methods

n/a	Involved in the study
☒	☐ ChIP-seq
☒	☐ Flow cytometry
☒	☐ MRI-based neuroimaging

Antibodies

Antibodies used

IHC and IF stainings:
1. Rabbit monoclonal anti- Arg1,servicebio,cat#GB115724,dil: 1:2000
2. Rabbit monoclonal anti-CD44, servicebio, cat# GB112054,dil: 1:150
3. Rabbit monoclonal anti-CD86, servicebio, clone: SB350, cat# GB150054,dil: 1:2000
4. Rabbit monoclonal anti-CD206, servicebio , cat# GB113497,dil: 1:3000
5. Rabbit monoclonal anti-Claudin 1, servicebio,clone: SB151, cat# GB152543,dil: 1:5000
6. Rabbit monoclonal anti-FOXP3, servicebio,clone: SB369, cat# GB152325,dil: 1:500
7. Rabbit monoclonal anti-NE, ZENBIO,clone: R01-6K1, cat# R381631,dil: 1:100
8. Rabbit monoclonal anti-Occludin, servicebio, cat# GB111401,dil: 1:5000
9. Rabbit monoclonal anti-ZO-1, servicebio,clone: SB192, cat# GB151981,dil: 1:4000
10. Rabbit monoclonal anti-MPO, servicebio,clone: SB135, cat# GB150006,dil: 1:500
10. S-vision Immunohistochemical polyclonal antibody (Goat against Rabbit), Servicebio,cat# G1302, dil: Ready to use
11. Cy3-labeled goat anti-rabbit IgG, Servicebio, cat#GB21303, dil: 1:300

Western blot:
1. Mouse monoclonal anti-GAPDHAffinity Biosciencesclone2F40CAT#T0004dil:12000
2. Rabbit monoclonal anti-TNF-aImmunowayclonePT0472RCAT#YM8306,dil1:1000
3. Rabbit monoclonal anti-IKBaImmunowayclonePT0702RCAT#YM8511dil1:1000
4. Rabbit monoclonal anti-p-IKBa ImmunowayPhospho Ser32/36CAT#YP0151dil1:1000
5. Mouse monoclonal anti-NF-KB P65Affinity BiosciencesCAT#BF8005dil1:1000
6. Rabbit monoclonal anti-p-NFKB-kappaB p65Cell Signaling Technology,Phospho Ser 536clone93H1CAT# 3033dil1:1000
7. HRP-Conjugated Goat anti-Mouse IgGH+L), Thermo Fisher Scientific, CAT#31430, dil: 1:10000
8. HRP-Conjugated Goat Anti-Rabbit IgG(H+L) , NCM Biotech, Cat#P8002, dil: 1: 10000
9. 6x-HIS Tag Monoclonal Anti- Mouse / IgG2b, Invitrogen, 6x-HIS Tag Monoclonal Antibody, Invitrogen,clone HIS.H8, dil: 1:1,000

Validation

All primary antibodies were commercially available and were validated by the supplier. All antibodies were used in the study according to theprofile of manufacturers. All validation statements are available on the antibody websites, respectively.

IHC and IF stainings:
1. Rabbit monoclonal anti- Arg1,servicebio,cat#GB115724,dil: 1:2000
2. Rabbit monoclonal anti-CD44, servicebio, cat# GB112054,dil: 1:150
3. Rabbit monoclonal anti-CD86, servicebio, clone: SB350, cat# GB150054,dil: 1:2000
4. Rabbit monoclonal anti-CD206, servicebio , cat# GB113497,dil: 1:3000
5. Rabbit monoclonal anti-Claudin 1, servicebio,clone: SB151, cat# GB152543,dil: 1:5000
6. Rabbit monoclonal anti-FOXP3, servicebio,clone: SB369, cat# GB152325,dil: 1:500
7. Rabbit monoclonal anti-NE, ZENBIO,clone: R01-6K1, cat# R381631,dil: 1:100
8. Rabbit monoclonal anti-Occludin, servicebio, cat# GB111401,dil: 1:5000
9. Rabbit monoclonal anti-ZO-1, servicebio,clone: SB192, cat# GB151981,dil: 1:4000
10. Rabbit monoclonal anti-MPO, servicebio,clone: SB135, cat# GB150006,dil: 1:500
10. S-vision Immunohistochemical polyclonal antibody (Goat against Rabbit), Servicebio,cat# G1302, dil: Ready to use
11. Cy3-labeled goat anti-rabbit IgG, Servicebio, cat#GB21303, dil: 1:300

Western blot:
1. Mouse monoclonal anti-GAPDHAffinity Biosciencesclone2F40CAT#T0004dil:12000

2. Rabbit monoclonal anti-TNF-aImmunowayclonePT0472RCAT#YM8306,dil1:1000
3. Rabbit monoclonal anti-IKBaImmunowayclonePT0702RCAT#YM8511dil1:1000
4. Rabbit monoclonal anti-p-IKBa ImmunowayPhospho Ser32/36CAT#YP0151dil1:1000
5. Mouse monoclonal anti-NF-KB P65Affinity BiosciencesCAT#BF8005dil1:1000
6. Rabbit monoclonal anti-p-NFKB-kappaB p65Cell Signaling Technology,Phospho Ser 536clone93H1CAT# 3033dil1:1000
7. HRP-Conjugated Goat anti-Mouse IgGH+L), Thermo Fisher Scientific, CAT#31430, dil: 1:10000
8. HRP-Conjugated Goat Anti-Rabbit IgG(H+L) , NCM Biotech, Cat#P8002, dil: 1: 10000
9. 6x-HIS Tag Monoclonal Anti- Mouse / IgG2b, Invitrogen, 6x-HIS Tag Monoclonal Antibody, Invitrogen,clone HIS.H8, dil: 1:1,000

Animals and other research organisms

Policy information about studies involving animals; ARRIVE guidelines recommended for reporting animal research, and Sex and Gender in Research

Laboratory animals	Male C57BL/6J mice (6–8 weeks old, 21–23 g) used in colitis models were bred and housed under specific pathogen-free (SPF) conditions. The mice were maintained in microisolator cages on individually ventilated racks, with aspen chip bedding, in a controlled environment (temperature: 20?–22?, humidity: 50%–60%, 12-h light/dark cycle). Autoclaved rodent chow and water were provided ad libitum. Mice were acclimatized for one week prior to experiments. Both experimental and control groups were housed separately in different cages. All experiments were performed using male C57BL/6J mice.
Wild animals	No wild animals were used in this study
Reporting on sex	We did not consider the influence of sex in the study design. Male mice were used for all the animal assay as reported in the literature studies
Field-collected samples	No field-collected samples were used in this study.
Ethics oversight	The animal study was approved by the Ethics Committee of Hunan Agricultural University, and the animal certification number is Ethics Approval 2023 No. 146. The animal experiment guidance from the Ethics Committee of Hunan Agricultural University and the Guide for the Care and Use of Laboratory Animals from the NIH were followed throughout the entire experiment.

Note that full information on the approval of the study protocol must also be provided in the manuscript.

Plants

Seed stocks	N/A
Novel plant genotypes	N/A
Authentication	N/A
