# Supplementary material for: Bioinspired microcapsule reactor with engineered probiotics for IBD therapy
Source: Nat Commun. 2026 Jul 13;17:6095. doi: 10.1038/s41467-026-72027-1 (PMC13358097; doi:10.1038/s41467-026-72027-1)
Supplement: Supplementary file 5 — Transparent Peer Review file [file 41467_2026_72027_MOESM5_ESM.pdf]

nature portfolio

Peer Review File

Bioinspired Microcapsule Reactor with Engineered Probiotics for Smart IBD Therapy

Corresponding Author: Dr Guangfu Feng

This file contains all reviewer reports in order by version, followed by all author rebuttals in order by version.

Version 0:

Reviewer comments:

Reviewer #1

(Remarks to the Author)
This study proposes and validates a “coral-inspired” oral microcapsule reactor (MY-E@SS), in which an engineered E. coli Nissle 1917 strain (MY-EcN) that secretes the anti-inflammatory peptide KPV under inflammation-specific enzymatic cleavage is encapsulated within a multifunctional shell composed of silk fibroin, dopamine, and mannose (SS). The microcapsules are fabricated through droplet microfluidics to achieve gastrointestinal tolerance, inflammation-responsive release, prolonged intestinal colonization, and synergistic scavenging of reactive oxygen and nitrogen species (RONS), thereby alleviating colitis in DSS-induced mouse models. In addition, MY-E@SS restores respiratory metabolism in mice with IBD. Overall, the study presents an innovative concept with a highly integrated design, extensive experimental validation, and multi-omics support, demonstrating strong conceptual novelty and translational potential. However, several critical issues and evidential gaps remain to be addressed.
1）The in vitro LC-MS analysis and HNE digestion experiments demonstrated that the MY protein can be cleaved by HNE to release KPV; however, direct in vivo evidence is lacking. Consequently, it remains unclear whether an effective concentration of KPV is achieved within the colon and where it exerts its biological function. Quantitative determination of KPV levels in colonic tissues should be provided.
2）HNE is a human neutrophil elastase, while the animal model used in this study is the C57BL/6J mouse rather than a transgenic strain expressing human HNE. It should be clarified whether murine neutrophil elastase can similarly trigger the cleavage of the MY protein in vivo.
3）Transcriptomic, qPCR, and metabolomic analyses suggest the involvement of the NF-κB, TNF, MMP9, and arachidonic acid pathways; however, direct mechanistic evidence is still insufficient. It remains to be determined whether KPV directly acts on epithelial or immune cells to inhibit NF-κB activation, or whether the observed effects are indirectly mediated through alterations in the gut microbiota.
4）Although the manuscript emphasizes a coral-inspired design, evidence supporting the role of MY-E@SS in promoting tissue repair during IBD treatment is lacking. Additional data should be provided to demonstrate the recovery of tight junctions and intestinal mucosal integrity after MY-E@SS treatment. Furthermore, quantitative analysis of neutrophil infiltration at inflamed colonic sites should be included to reflect the activation threshold of the elastase-responsive mechanism of MY-E@SS.
5）IBD encompasses both ulcerative colitis and Crohn’s disease, yet the current study only provides therapeutic evidence in an acute DSS-induced colitis model. Further validation in a TNBS-induced colitis model is necessary to substantiate the therapeutic effects of MY-E@SS.
6）The study attributes the improvement in respiratory metabolism of IBD mice treated with MY-E@SS to microbiota restoration and immune modulation; however, only RNA sequencing data are presented. Detailed immunological analyses are required to elucidate which specific immune cell populations are modulated during treatment. Flow cytometry should be employed to verify the immune cell subsets responsible for the observed therapeutic improvements.

Reviewer #2

(Remarks to the Author)
The manuscript entitled “From Coral to Cure: Bioinspired Microcapsule Reactor with Engineered Probiotics for Smart IBD Therapy” presents a highly creative and interdisciplinary study integrating synthetic biology and biomimetic design for inflammatory bowel disease (IBD) therapy. The concept inspired by the coral “defense–danger sensing–tissue repair”

cascade is novel, and the experimental results are systematically organized across molecular, cellular, and in vivo levels. The study is scientifically sound and has promising translational potential. However, before publication, several critical issues need to be addressed to strengthen the mechanistic depth, data quantification, and overall presentation quality.
1. Although the transcriptomic and metabolomic analyses indicate modulation of inflammation-related signaling pathways, the evidence presented remains largely correlative. The authors are encouraged to perform additional mechanistic experiments to directly confirm whether the therapeutic effects of MY-E@SS are indeed mediated through NF-κB or TNF pathway inhibition and KPV peptide release.
2. The current assessment of SS-shell degradation and bacterial/peptide release is primarily qualitative. To better understand the delivery kinetics of the bioinspired microcapsule reactor, the authors should provide quantitative data such as in vivo degradation rates, diffusion or release modeling, or spatiotemporal tracking using fluorescence or radiolabel imaging.
3. The manuscript reports changes in cytokine levels (IL-6, IL-1β, TNF-α, IL-10), but does not examine how MY-E@SS affects specific immune cell populations. It would be valuable to include flow-cytometry or immunofluorescence analyses of macrophage polarization (M1/M2) and regulatory T-cell (Treg) dynamics.
4. The expression of the MY protein relies on an L-arabinose–inducible pBAD system, which may not maintain stable induction within the gastrointestinal tract. The authors should quantify MY protein levels in intestinal tissues (e.g., via ELISA or Western blot) to confirm in vivo expression, and discuss alternative promoter strategies—such as inflammation-responsive or auto-inducible systems—to enhance stability and predictability under physiological conditions.
5. The SS shell incorporates silk fibroin, dopamine, and mannose conjugates, while the MY protein contains highly repetitive RGD/AAPV-KPV tandem sequences (22 repeats). No assessment is provided regarding possible immunogenicity, antibody formation, hypersensitivity, or long-term toxicity associated with these components.

Reviewer #3

(Remarks to the Author)
The manuscript describes the development of a core–shell bionic microcapsule system (MY-E@SS) that integrates engineered bacteria with a multifunctional protective shell to treat inflammatory bowel disease (IBD). The concept is innovative, and the preliminary results are promising. However, several major concerns regarding experimental design, mechanistic depth, reproducibility, and clarity of data interpretation must be addressed before the manuscript can be considered for publication.

Major comment
1. The study presents an innovative bionic microcapsule system inspired by coral community defense and repair mechanisms, but the rationale for selecting coral as the biological model should be more rigorously justified. It would strengthen the manuscript to compare this model with alternative biological systems that also exhibit multi-level cooperative responses.

2. The design principles of the MY-E@SS platform are conceptually compelling, yet the engineering logic behind each component of the core–shell structure could be described more systematically. In particular, the manuscript should clearly articulate how each structural layer contributes to gastric protection, targeted release, and synergistic therapeutic performance.

3. The characterization of bacterial survival, colonization efficiency, and functional peptide release is insufficiently quantified. Additional data on stability under gastrointestinal conditions, long-term viability, and in vivo colonization dynamics would support the claimed advantages.

4. While the study demonstrates therapeutic effects in an IBD model, the experimental design would benefit from the inclusion of more rigorous controls, especially comparisons with non-bionic engineered bacteria and traditional probiotic formulations. This would help clarify whether the therapeutic benefits arise primarily from the engineered strain, the microcapsule design, or their synergy.

5. The mechanistic analysis—particularly regarding gene expression changes and modulation of arachidonic acid metabolism—should be expanded. The current evidence supports association but not causality. Additional validation experiments, such as inhibition or rescue assays, would enhance mechanistic clarity.

6. The manuscript claims systemic physiological improvement and microbiota homeostasis restoration, but the supporting data are briefly described. A more detailed presentation of microbiome profiling, diversity metrics, and longitudinal dynamics would reinforce these conclusions.

7. Biocompatibility and biosafety assessments are important strengths of the work; however, long-term safety, potential off-target effects, and horizontal gene transfer risks of engineered bacteria should be discussed more comprehensively.

Minor comment:
1. The current title, “From Coral to Cure: Bioinspired Microcapsule Reactor with Engineered Probiotics for Smart IBD Therapy,” reads more like a feature article or a perspective piece rather than a primary research paper. The use of a slogan-like phrase (“From Coral to Cure”) adds a strong narrative or promotional tone, which may be less appropriate for a scientific research article.
2. Terminology inconsistencies, for example: "Intelligently detect inflammation" too vague; "Synergistic therapy" needs

definition; "Physiological functions improved" specify which functions (e.g., weight, cytokines)

Version 1:

Reviewer comments:

Reviewer #1

(Remarks to the Author)
All issues have been well addressed and so I recommend its publication in the current form.

Reviewer #2

(Remarks to the Author)
The revised manuscript is ready for publication.

Reviewer #3

(Remarks to the Author)
I appreciate the substantial effort the authors have made to address the previous concerns. Overall, the revised manuscript is significantly strengthened, particularly in terms of mechanistic clarification, quantitative validation, and control design. Below is a structured re-evaluation of each major point, with specific comments on adequacy and remaining gaps.
1. Rationale for Coral-Inspired Design
The authors have substantially improved the conceptual justification for selecting coral as the biomimetic model. The comparison with alternative biological systems (e.g., plant closure, immune cascades) is now more explicit, and the mapping of “exoskeleton–mucus–symbiont” to “shell–adhesion–engineered bacteria” is logically articulated.
2. Engineering Logic of Core–Shell Structure
The engineering rationale is now systematically described, and the structure–function relationship of each component (RGD, AAPV, KPV, dopamine, mannose, SF) is clarified.
3. Quantification of Survival, Colonization, and Peptide Release
This section is significantly strengthened.
4. Control Groups and Synergy
The inclusion of P-EcN, MY-EcN, and SS controls across multiple endpoints is appropriate and rigorous.
5. Mechanistic Depth (NF-κB, Arachidonic Acid Pathway)
The addition of NF-κB pathway validation (p65 translocation, IκBα phosphorylation, TNF-α secretion) significantly strengthens the mechanistic claims.
The reviewer’s original concern also mentioned arachidonic acid metabolism. The current response focuses almost exclusively on NF-κB inhibition. If the manuscript claims modulation of arachidonic acid metabolism, causal validation is still lacking.
6. Microbiome and Systemic Physiology
This section is significantly improved.
7. Biosafety and Horizontal Gene Transfer
Assessment:
The discussion has been expanded and now acknowledges HGT and off-target risks.

Open Access This Peer Review File is licensed under a Creative Commons Attribution 4.0 International License, which permits use, sharing, adaptation, distribution and reproduction in any medium or format, as long as you give appropriate credit to the original author(s) and the source, provide a link to the Creative Commons license, and indicate if changes were made.
In cases where reviewers are anonymous, credit should be given to 'Anonymous Referee' and the source.
The images or other third party material in this Peer Review File are included in the article's Creative Commons license, unless indicated otherwise in a credit line to the material. If material is not included in the article's Creative Commons license and your intended use is not permitted by statutory regulation or exceeds the permitted use, you will need to obtain permission directly from the copyright holder.
To view a copy of this license, visit https://creativecommons.org/licenses/by/4.0/

Responses to the Editor and Referees' Comments

Dear Editors and Reviewers,

Thank you for your letter and the reviewers' comments concerning our manuscript entitled "From Coral to Cure: Bioinspired Microcapsule Reactor with Engineered Probiotics for Smart IBD Therapy" (Manuscript ID: NCOMMS-25-82459). Those comments are all valuable and very helpful for revising and improving our paper, as well as the important guiding significance to our researches. Based on these comments and suggestions, we have rewritten the manuscript with substantial modification, incorporating the reviewers' points, and the revised portions are highlighted in red. Please also see our point-by-point responses to reviewer's comments below.

Reviewer #1:

This study proposes and validates a "coral-inspired" oral microcapsule reactor (MY-E@SS), in which an engineered *E. coli* Nissle 1917 strain (MY-EcN) that secretes the anti-inflammatory peptide KPV under inflammation-specific enzymatic cleavage is encapsulated within a multifunctional shell composed of silk fibroin, dopamine, and mannose (SS). The microcapsules are fabricated through droplet microfluidics to achieve gastrointestinal tolerance, inflammation-responsive release, prolonged intestinal colonization, and synergistic scavenging of reactive oxygen and nitrogen species (RONS), thereby alleviating colitis in DSS-induced mouse models. In addition, MY-E@SS restores respiratory metabolism in mice with IBD. Overall, the study presents an innovative concept with a highly integrated design, extensive experimental validation, and multi-omics support, demonstrating strong conceptual novelty and translational potential. However, several critical issues and evidential gaps remain to be addressed.

Response: Thanks very much for your thorough review and high recognition of our research. Following your advice, we have conducted the following supplementary experiments and analyses to enhance the rigor and persuasiveness of the study. Point-by-point responses to your comments are showed as follows.

1) The *in vitro* LC-MS analysis and HNE digestion experiments demonstrated that the MY protein can be cleaved by HNE to release KPV; however, direct *in vivo* evidence is lacking. Consequently, it remains unclear whether an effective concentration of KPV is achieved within the colon and where it exerts its biological function. Quantitative determination of KPV levels in colonic tissues should be provided.

Response: Thank you for raising this critical question. In response to your suggestions, two additional sets of experiments were conducted to directly investigate the *in vivo* release and distribution of KPV:

(1) Time-course release of KPV in the colon: Colon contents were collected at 0, 1,

4, 8, and 12 h after intragastric gavage of MY-E@SS, and KPV concentrations were quantified by LC-MS/MS (Fig. S4a). The results showed that KPV concentration peaked at 4 h and declined gradually thereafter, consistent with the colonization kinetics of the engineered bacteria in the colon. These data provide direct evidence that neutrophil elastase (NE) cleaves the MY protein *in vivo*, leading to KPV release.

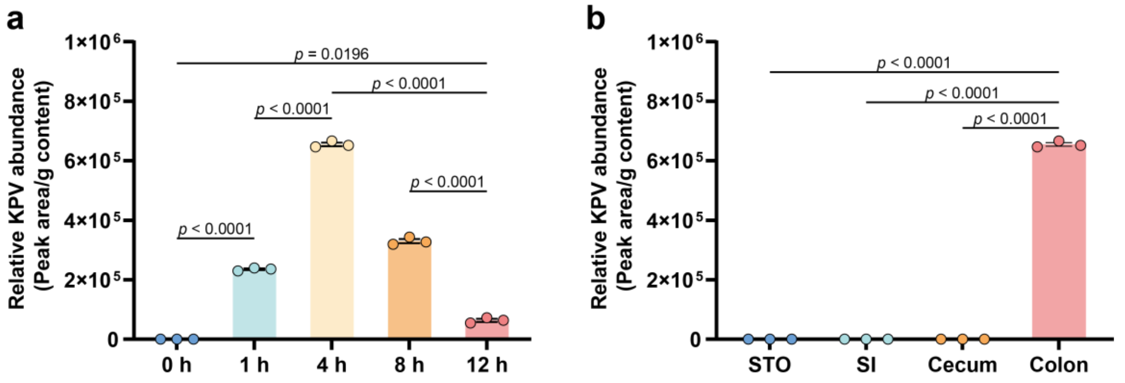

Figure S4. Temporal and distribution analysis of active peptide KPV released by MY-E@SS in a DSS-induced IBD mouse model. (a). Time-course changes in KPV concentration in colonic contents. (b). Distribution of KPV in the contents of different gastrointestinal segments at 4 h post-gavage.

(2) Gastrointestinal distribution of KPV: At 4 h after oral administration, contents from the stomach, small intestine, cecum, and colon were collected for quantitative analysis by LC-MS/MS (Fig. S4b). KPV was found to be primarily enriched in the colon, while virtually no KPV was detected in the cecum, stomach, or small intestine. These results indicate that the release of KPV is colon-targeted, which aligns with the expected site of action.

(3) Additionally, **the expression of the MY protein in colonic contents** 4 h after oral administration was measured by ELISA (Fig. S5). MY protein levels were significantly higher in the MY-E@SS group than in the control group, further supporting the conclusions stated above.

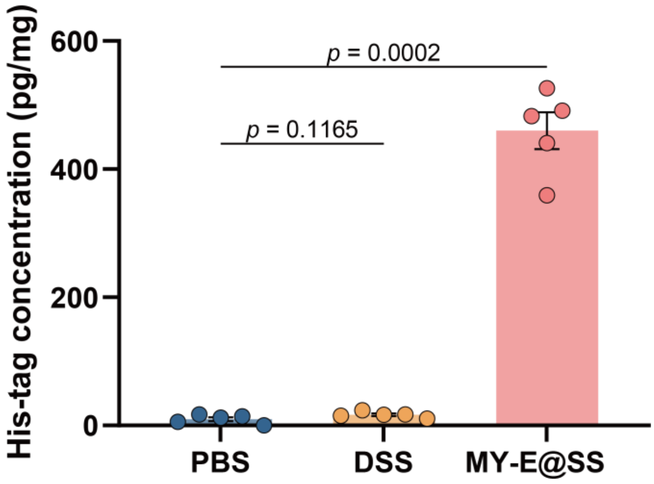

Figure S5. Quantification of MY protein expression levels in colonic contents of mice from each group by ELISA.

We have revised our manuscript as follows:

Line 154-158: To further confirm the *in vivo* release and localization of KPV, we monitored its concentration in colonic contents over time. The levels peaked at 4 h post-administration (Fig. S4a), and specific accumulation in the colon was verified (Fig. S4b). Moreover, elevated MY protein levels in colonic contents of MY-E@SS treated mice (Fig. S5) corroborated effective *in vivo* expression and processing.

2）HNE is a human neutrophil elastase, while the animal model used in this study is the C57BL/6J mouse rather than a transgenic strain expressing human HNE. It should be clarified whether murine neutrophil elastase can similarly trigger the cleavage of the MY protein *in vivo*.

Response: Thanks for your insightful comments. Based on the existing literature and our additional experimental data, we have conducted argumentation and clarification from the following perspectives:

(1) Evidence of functional conservation from literature: First, neutrophil elastase exhibits a high degree of evolutionary conservation among mammals. Sequence alignment analyses indicate that the amino acid sequence homology of elastase between mice and humans reaches approximately 74%. More importantly, functional precedents exist in previous studies. For instance, one study successfully induced typical pulmonary inflammatory responses in mice by delivering porcine pancreatic elastase into the airways [1], which directly demonstrates the functional similarity in substrate recognition and cleavage between elastases across species (porcine and murine). Another study [2] found that a neutrophil elastase inhibitor isolated from centipede effectively inhibited the activities of both porcine pancreatic elastase and human HNE, further supporting the broad conservation of substrate specificity among this class of proteases.

(2) Conservation of cleavage sites: The cleavage site we designed (AAPV) is based on the canonical recognition sequence of HNE. It has been clearly indicated in a study [3] that neutrophil elastase tends to cleave after small hydrophobic amino acids (such as alanine A, valine V) or threonine (T). The AAPV sequence we designed fully conforms to this characteristic, and this sequence is commonly found in known substrates of both human and mouse elastases, suggesting a high likelihood of recognition by murine neutrophil elastase (mNE).

(3) Direct *in vivo* experimental evidence: As stated in our response to your first comment, in a DSS-induced colitis mouse model, the concentration of KPV in the colonic contents after oral administration of MY-E@SS was significantly higher than that in the control group (Fig. S4). This result directly confirms that murine neutrophil elastase (mNE) accumulated at the inflammatory site can effectively cleave the MY protein and release KPV, thereby validating the responsive release mechanism and efficacy of this delivery system in the mouse model. To accurately indicate the species origin, we have uniformly revised “HNE” to “neutrophil elastase (NE)” throughout the main text.

References:

[1] X. Liu, B. Ucakar, K. Vanvarenberg, E. Marbaix and R. Vanbever, Impact of administration route and PEGylation on alpha-1 antitrypsin augmentation therapy, Journal of Controlled Release, 382 (2025) https://doi.10.1016/j.jconrel.2025.113643

[2] N. Luan, Q. Zhao, Z. Duan, M. Ji, M. Xing, T. Zhu, J. Mwangi, M. Rong, J. Liu and R. Lai, Identification and Characterization of ShSPI, a Kazal-Type Elastase Inhibitor from the Venom of Scolopendra Hainanum, Toxins, 11 (2019) https://doi.10.3390/toxins11120708

[3] R. Kettritz, Neutral serine proteases of neutrophils, Immunological Reviews, 273 (2016) 232–248. https://doi.10.1111/imr.12441

3）Transcriptomic, qPCR, and metabolomic analyses suggest the involvement of the NF-κB, TNF, MMP9, and arachidonic acid pathways; however, direct mechanistic evidence is still insufficient. It remains to be determined whether KPV directly acts on epithelial or immune cells to inhibit NF-κB activation, or whether the observed effects are indirectly mediated through alterations in the gut microbiota.

Response: Thanks for your comments. To provide direct evidence at the cellular signaling pathway level, two *in vitro* experiments were supplemented.

(1) Inhibition of NF-κB p65 nuclear translocation in RAW*264.7* macrophages: using an LPS-induced inflammatory model in RAW*264.7* macrophages, the nuclear translocation of the p65-NF-κB subunit was examined. Compared with the LPS-treated group, co-treatment with MY-EH@SS concentration-dependently suppressed the nuclear migration of p65 (Fig. 9l, S49), directly demonstrating its ability to block NF-κB transcriptional activation.

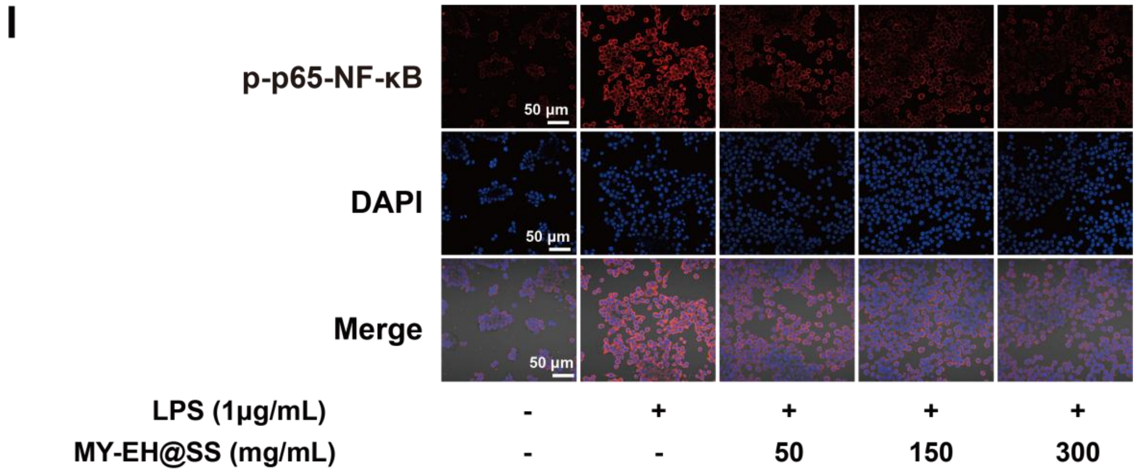

Figure 9 (l) Representative confocal microscopy images showing the inhibition of LPS-induced NF-κB p65 nuclear translocation in RAW*264.7* macrophages by MY-EH@SS. Nuclei were stained with DAPI (blue), and p65 was immunofluorescently labeled (red).

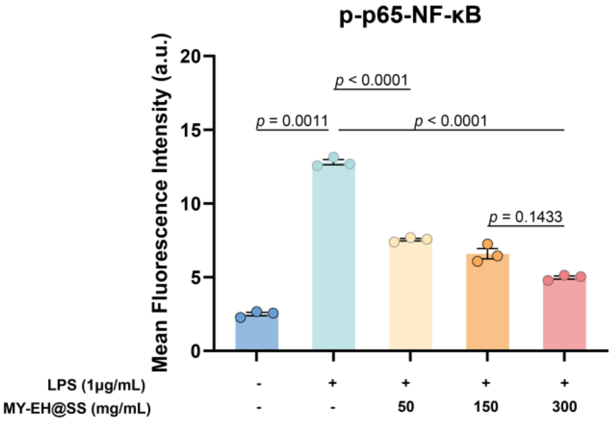

Figure S49. Quantification of p65 nuclear translocation from confocal images.

(2) To further validate the effect, 150 μg/mL of MY-EH@SS was used. This treatment significantly suppressed IκBα phosphorylation and degradation, and reduced p65 phosphorylation (Fig. 9m, S51). It also effectively inhibited LPS-induced TNF-α secretion to an extent comparable to the TNF-α inhibitor etanercept (Fig. 9n). Together, these results demonstrate that MY-EH@SS blocks the canonical NF-κB pathway by inhibiting upstream IκBα phosphorylation, thereby attenuating inflammatory cytokine production.

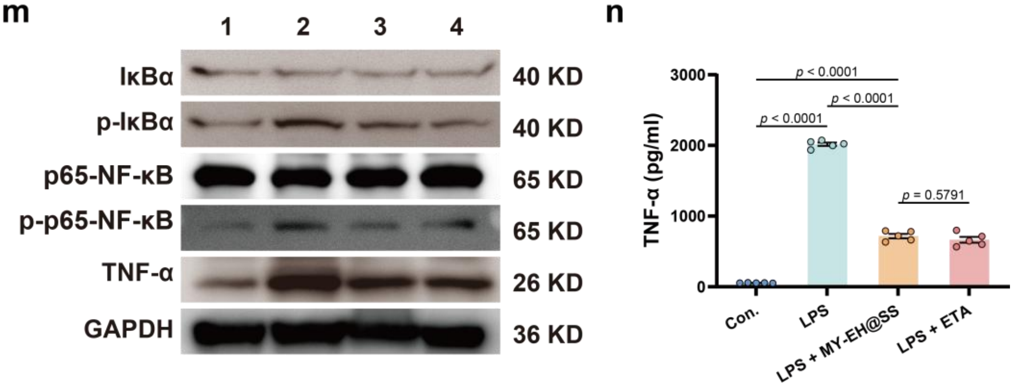

Figure 9. (m) Western blot analysis of key proteins in the TNF-α/NF-κB pathway in RAW*264.7* macrophages. MY-EH@SS (150 μg/mL) inhibited LPS-induced IκBα phosphorylation (p-IκBα) and degradation, as well as NF-κB p65 phosphorylation (p-p65). Etanercept (ETA) was used as a positive control. Cells were treated as follows: Lane 1, control (Con); Lane 2, LPS; Lane 3, LPS + MY-EH@SS (150 μg/mL); Lane 4, LPS + Etanercept (ETA, positive control). (n) ELISA quantification of TNF-α secretion in the cell culture supernatant of RAW*264.7* macrophages.

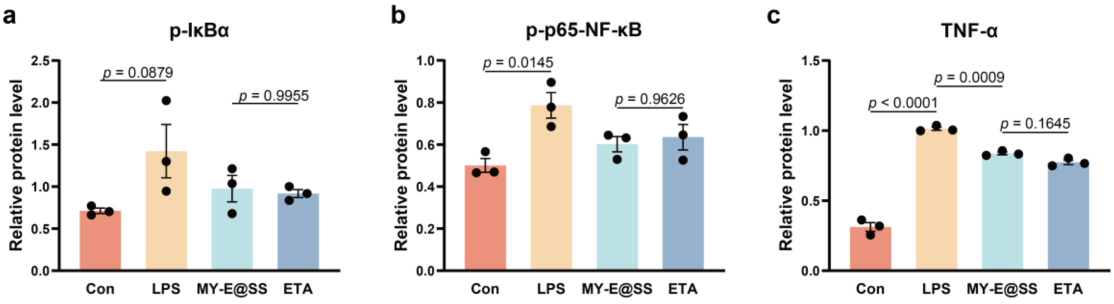

Figure S51. Quantitative analysis of TNF-α/NF-κB pathway inhibition by MY-E@SS in RAW264.7 macrophages. (a) Quantitative analysis of p-IκBα protein levels normalized to GAPDH. (b) Quantitative analysis of p-p65-NF-κB protein levels normalized to GAPDH. (c) Quantitative analysis of TNF-α protein levels normalized to GAPDH.

These results demonstrate that bioinspired microcapsule reactor acts directly on macrophages and exerts anti-inflammatory effects by inhibiting the TNF-α/NF-κB pathway, providing direct experimental support for the proposed mechanism of action of the KPV peptide.

We have added that information in our revised manuscript as follows:

Line 507-516: To directly validate this mechanism, *in vitro* studies were conducted in LPS-stimulated RAW264.7 macrophages, where MY-E@SS treatment was observed to concentration-dependently inhibit LPS-induced p65 nuclear translocation. (Fig. 9l, S49). Further investigation revealed that at 150 μg/mL, it significantly suppressed TNF-α secretion to a level comparable to the inhibitor etanercept and concurrently reduced IκBα phosphorylation and degradation along with p65 phosphorylation (Fig. 9m, n, S50 and S51). These results establish that MY-EH@SS exerts its anti-inflammatory effect through direct inhibition of the TNF-α/NF-κB signaling pathway. These results demonstrate that MY-E@SS counteracts inflammation through directly inhibiting the TNF-α/NF-κB signaling pathway in immune cells, thereby suppressing downstream cytokine production and inflammatory activation (Fig. 9o).

4） Although the manuscript emphasizes a coral-inspired design, evidence supporting the role of MY-E@SS in promoting tissue repair during IBD treatment is lacking. Additional data should be provided to demonstrate the recovery of tight junctions and intestinal mucosal integrity after MY-E@SS treatment. Furthermore, quantitative analysis of neutrophil infiltration at inflamed colonic sites should be included to reflect the activation threshold of the elastase-responsive mechanism of MY-E@SS.

Response: Thanks for your thoughtful question and for highlighting this important point. In response to your suggestions, two critical sets of experiments were added to directly evaluate the effect of MY-E@SS on intestinal barrier repair and to quantitatively verify the basis for the activation of its responsive mechanism:

(1) Evidence of intestinal barrier integrity and tight-junction recovery: colon tissue sections from mice in each group were subjected to immunofluorescence staining for the tight-junction proteins ZO-1 (Fig. S17a), Occludin (Fig. S17b), and Claudin-1 (Fig. S17c). Quantitative analysis (Fig. S17d) showed that, compared with the IBD model group, the MY-E@SS treatment group exhibited a significant restoration in both expression levels and continuous distribution of these three key tight-junction proteins in the colonic epithelium. These findings directly demonstrate that MY-E@SS effectively promotes structural repair of the intestinal mucosal barrier, providing solid morphological and molecular evidence for the "promoting tissue repair" effect described in the manuscript.

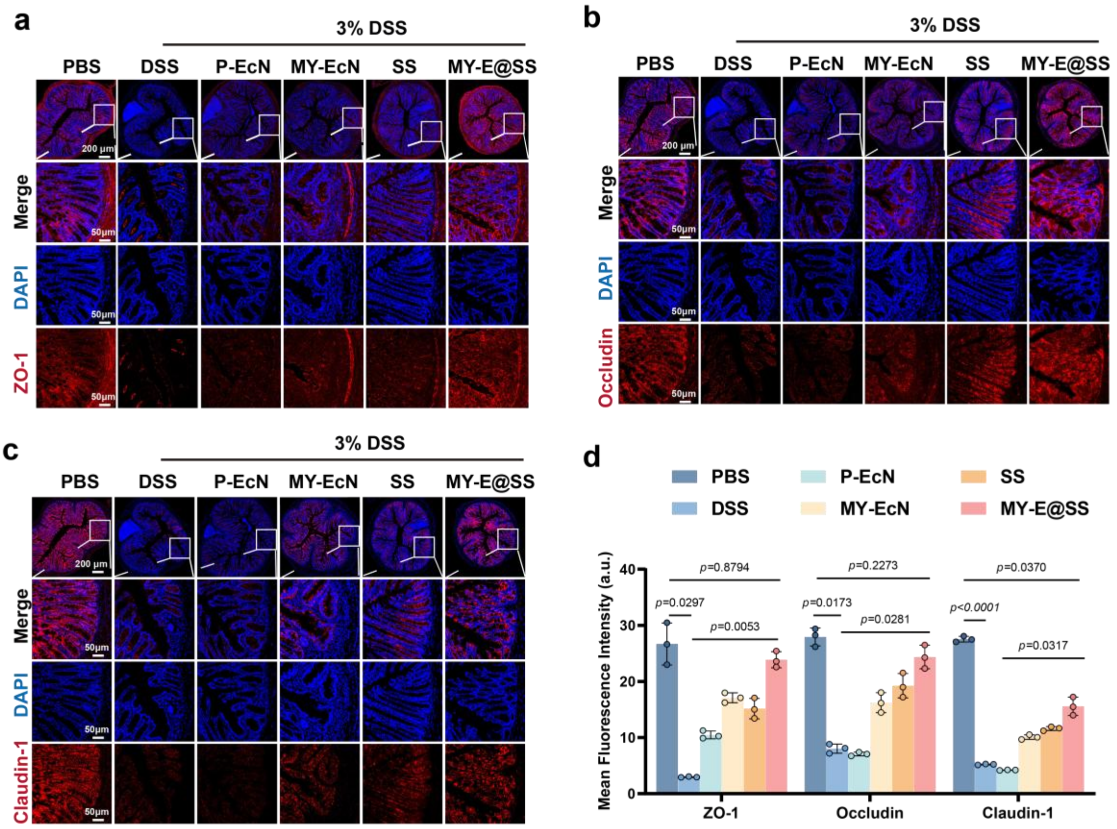

Figure S17. Assessment of tight junction protein expression based on immunofluorescence staining and quantitative analysis. (a-c) Representative immunofluorescence staining images of tight junction proteins ZO-1 (a), Occludin (b), and Claudin-1 (c) in colon tissues from each group of mice, respectively. (d) Quantitative analysis bar graph showing the relative expression levels of the three tight junction proteins.

(2) Quantitative analysis of neutrophil infiltration and enzymatic activity at inflammatory sites: To directly verify the microenvironment required for the responsive-release mechanism of our delivery system, immunofluorescence staining (Fig. S22a) and relative quantitative analysis (Fig. S22b) for neutrophil elastase (NE) were performed on colon tissue sections. The results showed a significant increase in neutrophil infiltration at the DSS-induced colitis sites, which provides direct *in vivo* evidence for the activation conditions under which the engineered bacteria sense and respond to this microenvironment to release KPV.

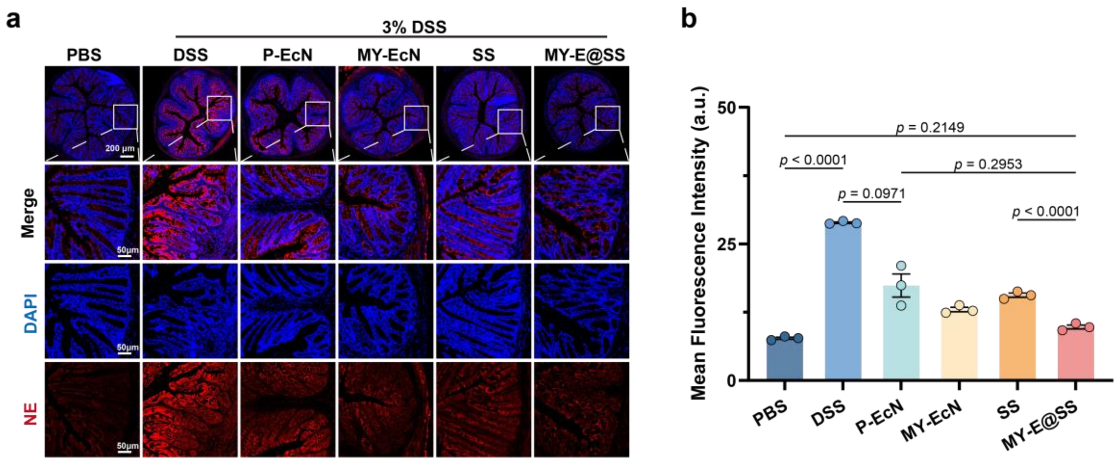

Figure S22. Quantitative Analysis of Neutrophil Elastase (NE) Expression and Neutrophil Infiltration at the Inflammatory Site. (a) Representative immunofluorescence staining images of NE in colon tissues from each group of mice. (b) Bar graph showing the relative expression levels of NE.

We have revised our manuscript as follows:

Line 283-285: Immunofluorescence analysis confirmed that MY-E@SS significantly restored the expression and distribution of key tight junction proteins (ZO-1, Occludin, and Claudin-1) in the colonic epithelium compared to the disease model (Fig. 5i, S17), supporting enhanced mucosal barrier integrity.

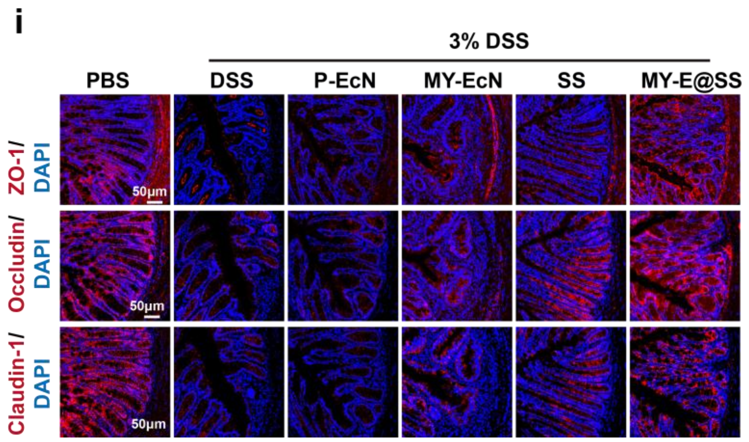

Figure 5 (i) Immunofluorescence staining of key tight junction proteins in transverse colon sections.

Line 292-295: Immunofluorescence staining for NE further demonstrated a significant increase in neutrophil infiltration at DSS-induced IBD (Fig. S22), which is consistent with the observed reduction in myeloperoxidase (MPO) activity (Fig. 5k) and corroborates the *in vivo* activation conditions for the engineered bacteria to sense and respond to this microenvironment.

5）IBD encompasses both ulcerative colitis and Crohn's disease, yet the current study only provides therapeutic evidence in an acute DSS-induced colitis model. Further validation in a TNBS-induced colitis model is necessary to substantiate the therapeutic effects of MY-E@SS.

Response: Thanks for your insightful comment. We have supplemented the

comprehensive efficacy validation experiments in the TNBS-induced colitis model (Fig. S24). The additional experimental design and key results are as follows: groups included a PBS control group, a TNBS model group, an engineered bacterial empty vector group (P-EcN), an MY-EcN treatment group, an SS treatment group, and an MY-E@SS treatment group. The following indicators were systematically evaluated:

(1) Disease activity: Changes in body weight and the Disease Activity Index (DAI) were monitored. The results showed that, compared with the TNBS model group and other control groups, MY-E@SS treatment most effectively alleviated weight loss and significantly reduced DAI scores.

(2) Systemic inflammation: Serum levels of inflammatory cytokines (such as TNF-α, IL-1β, IL-6, and IL-10) were measured, and the spleen index was assessed. The results indicated that MY-E@SS treatment significantly reduced systemic levels of pro-inflammatory cytokines and suppressed splenomegaly, demonstrating its efficacy in mitigating systemic inflammation.

(3) Local tissue damage and inflammatory infiltration: Pathological damage was assessed via H&E staining of colon tissues, and neutrophil infiltration was quantified through myeloperoxidase (MPO) staining. The results confirmed that MY-E@SS treatment markedly improved colonic mucosal structure, reduced ulceration, and significantly decreased MPO-positive cell counts, indicating effective suppression of local inflammatory responses.

We have revised our manuscript as follows:

Line 303-306: Importantly, a consistently potent therapeutic efficacy of MY-E@SS was also observed in a complementary (2,4,6-Trinitrobenzenesulfonic acid) TNBS-induced colitis model (Fig. S24), further substantiating its robust and reliable anti-inflammatory and mucosal healing properties across diverse experimental IBD settings.

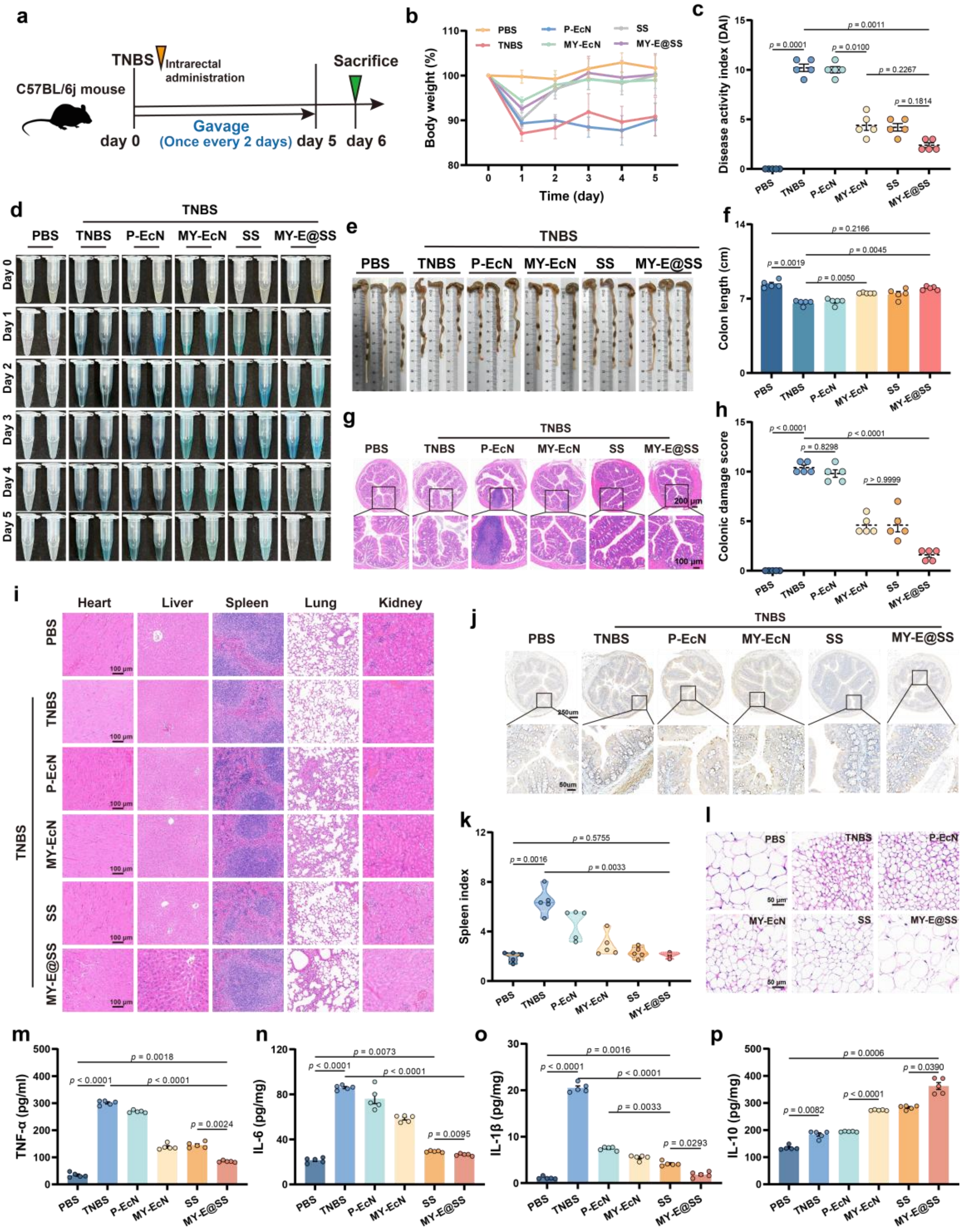

Figure S24. Therapeutic efficacy of MY-E@SS in a TNBS - induced IBD model. (a) Experimental timeline. Colitis was induced in C57BL/6 mice by a single intrarectal administration of TNBS on day 0. From day 1 to day 5, mice received oral gavage of PBS, P-EcN, MY-EcN, SS, or MY-E@SS every other day. (b) Body weight changes and (c) disease activity index (DAI) throughout the experimental period. (d) Temporal changes in fecal occult blood (photographically documented). (e) Representative colon images, (f) colon lengths, and (g) representative hematoxylin and eosin (H&E)-stained sections of colon tissues. (h) Colonic histopathological scores. (i) H&E-stained sections of major organs demonstrating systemic biocompatibility. (j) Myeloperoxidase (MPO) staining in colon sections. (k) Spleen index (spleen weight/body weight). (l) H&E-stained sections

of adipose tissue. (m–p) Cytokine levels (IL-6, TNF-α, IL-1β, IL-10) in colon tissues measured by ELISA. All samples were collected on day 6.

6) The study attributes the improvement in respiratory metabolism of IBD mice treated with MY-E@SS to microbiota restoration and immune modulation; however, only RNA sequencing data are presented. Detailed immunological analyses are required to elucidate which specific immune cell populations are modulated during treatment. Flow cytometry should be employed to verify the immune cell subsets responsible for the observed therapeutic improvements.

Response: Thanks for your kind comment. To further elucidate the specific immune cell subsets regulated by MY-E@SS during treatment, key immunophenotyping analyses were performed. Immunofluorescence staining of colon tissues was conducted to quantitatively evaluate changes in the number and phenotype of key immune cell populations in the mucosal lamina propria.

(1). We examined markers for pro-inflammatory M1 macrophages (CD86, Fig. S18c) and anti-inflammatory/reparative M2 macrophages (CD206, Fig. S18a; Arg1, Fig. S18b). Relative quantitative analysis (Fig. S18d) revealed that, compared with the IBD model group, MY-E@SS treatment significantly reduced infiltration of M1 macrophages ($CD86^{+}$) while markedly increasing the proportion of M2 macrophages ($CD206^{+}$, $Arg1^{+}$) in colonic tissue.

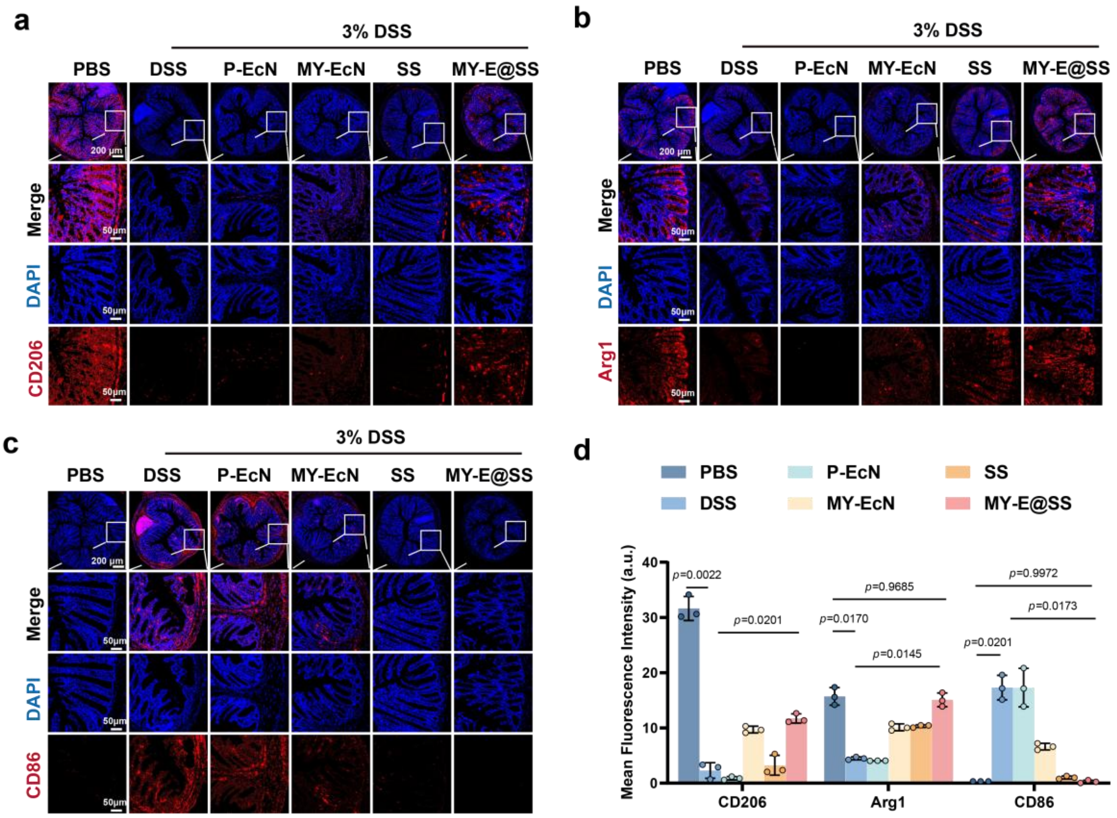

Figure S18. Immunophenotypic analysis of key immune cell subsets in colonic tissues following MY-E@SS treatment. (a–c) Representative immunofluorescence staining images of the M2 macrophage markers CD206 (a) and Arg1 (b), and the M1 macrophage marker CD86 (c) in colon

tissues. (d) Quantitative analysis of the relative abundance of M1 (CD86^{+}) and M2 (CD206^{+}/Arg1^{+}) macrophage populations.

(2) Regulatory T cell (Treg) dynamics: The dynamics of immunosuppressive regulatory T cells (Tregs) were assessed by quantifying Foxp3^{+}CD4^{+} T cells in the colonic mucosa (Fig. S19). Foxp3 is a Treg-specific transcription factor, and CD4 is a helper T cell marker. In MY-E@SS-treated mice, the number of these Tregs was significantly increased at colonic inflammatory sites.

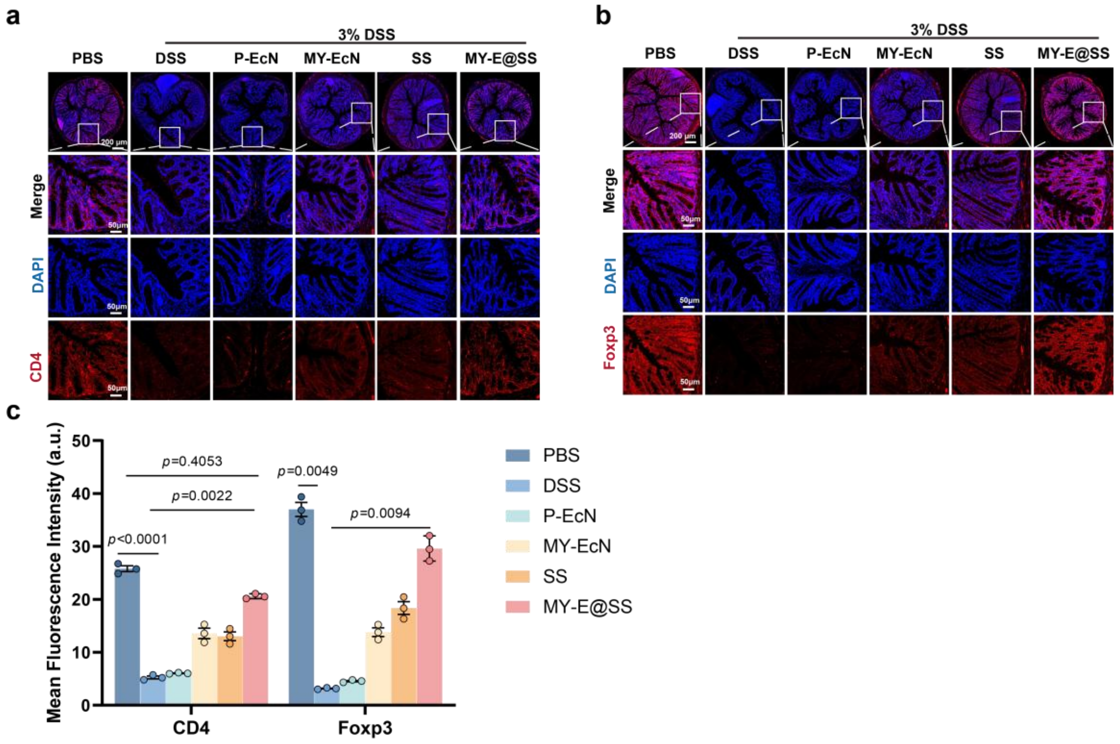

Figure S19. Dynamics of regulatory T cells (Tregs) in the colonic mucosa. (a) Representative immunofluorescence staining of CD4 (a marker for helper T cells) in colon tissues. (b) Representative immunofluorescence staining of Foxp3 (a Treg-specific transcription factor) in colon tissues. (c) Quantitative analysis of the relative abundance and distribution of Foxp3^{+}CD4^{+} regulatory T cells.

These results directly demonstrate that the therapeutic effect of MY-E@SS is closely associated with the polarization of the local immune microenvironment from a pro-inflammatory state toward an anti-inflammatory and reparative phenotype. We have added that information in our revised manuscript as follows:

Line 285-289: While MY-EcN or SS alone showed limited efficacy in intestinal repair, MY-E@SS promoted macrophage polarization from a pro-inflammatory (CD86+) to an anti-inflammatory/reparative (CD206^{+}/Arg1^{+}) phenotype and increased Foxp3^{+} CD4^{+} regulatory T cell infiltration, collectively rebalancing the local immune environment (Fig. 5j).

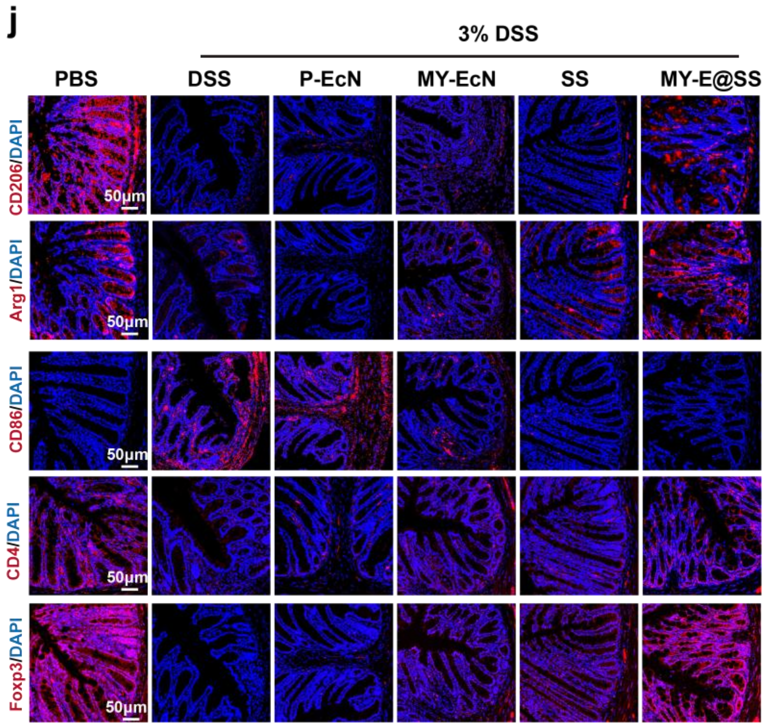

Figure 5 (j) Immunofluorescence analysis reveals MY-E@SS modulates the local colonic immune microenvironment.

Reviewer #2 (Remarks to the Author):

The manuscript entitled "From Coral to Cure: Bioinspired Microcapsule Reactor with Engineered Probiotics for Smart IBD Therapy" presents a highly creative and interdisciplinary study integrating synthetic biology and biomimetic design for inflammatory bowel disease (IBD) therapy. The concept inspired by the coral "defense–danger sensing–tissue repair" cascade is novel, and the experimental results are systematically organized across molecular, cellular, and *in vivo* levels. The study is scientifically sound and has promising translational potential. However, before publication, several critical issues need to be addressed to strengthen the mechanistic depth, data quantification, and overall presentation quality.

Response: We sincerely appreciate your positive assessment of the novelty, interdisciplinary nature, and scientific rigor of this work, as well as your recognition of its translational potential. We fully agree with your valuable suggestions regarding the mechanistic depth, data quantification, and overall presentation quality, which are critical to improving the manuscript. In response to your specific comments, we have carefully addressed each point in the revised version. In particular, we have further elaborated on the mechanistic interpretation, supplemented quantitative analyses, and refined the figures and textual descriptions to enhance the clarity of the research logic

and strengthen the supporting evidence. We hope that the revised manuscript now better addresses your concerns and thank you once again for your constructive feedback.

1. Although the transcriptomic and metabolomic analyses indicate modulation of inflammation-related signaling pathways, the evidence presented remains largely correlative. The authors are encouraged to perform additional mechanistic experiments to directly confirm whether the therapeutic effects of MY-E@SS are indeed mediated through NF-κB or TNF pathway inhibition and KPV peptide release.

Response: Thanks for your valuable comment. To provide direct evidence at the cellular signaling pathway level, two *in vitro* experiments were supplemented.

(1) Inhibition of NF-κB p65 nuclear translocation in RAW*264.7* macrophages: using an LPS-induced inflammatory model in RAW*264.7* macrophages, the nuclear translocation of the p65-NF-κB subunit was examined. Compared with the LPS-treated group, co-treatment with MY-EH@SS concentration-dependently suppressed the nuclear migration of p65 (Fig. 9l, S49), directly demonstrating its ability to block NF-κB transcriptional activation.

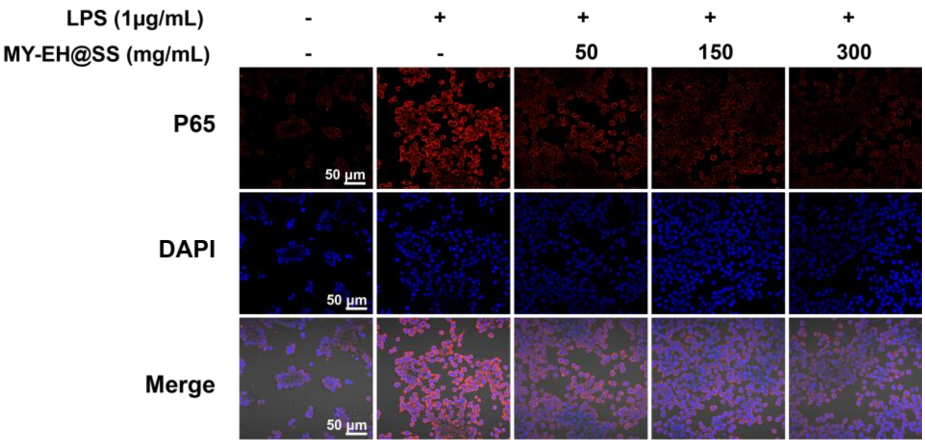

Figure 9 (l) Representative confocal microscopy images showing the inhibition of LPS-induced NF-κB p65 nuclear translocation in RAW*264.7* macrophages by MY-EH@SS. Nuclei were stained with DAPI (blue), and p65 was immunofluorescently labeled (red).

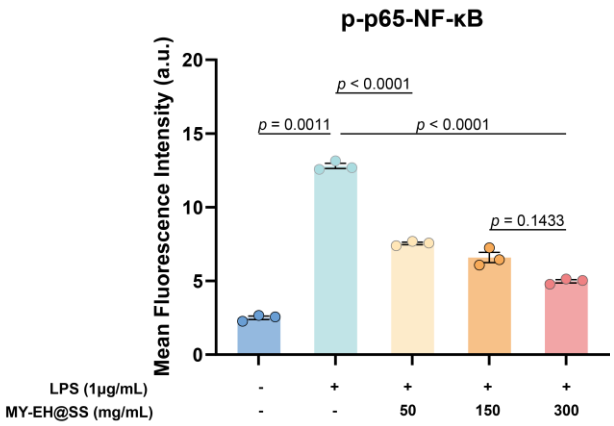

Figure S49. Quantification of p65 nuclear translocation from confocal images.

(2) To further validate the effect, 150 µg/mL of MY-EH@SS was used. This treatment significantly suppressed IκBα phosphorylation and degradation, and reduced p65 phosphorylation (Fig. 9m, S51). It also effectively inhibited LPS-induced TNF-α secretion to an extent comparable to the TNF-α inhibitor etanercept (Fig. 9n). Together, these results demonstrate that MY-EH@SS blocks the canonical NF-κB pathway by inhibiting upstream IκBα phosphorylation, thereby attenuating inflammatory cytokine production.

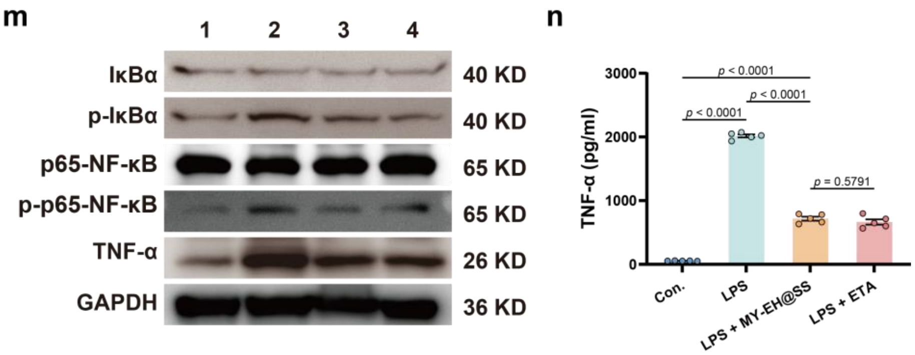

Figure 9. (m) Western blot analysis of key proteins in the TNF-α/NF-κB pathway in RAW*264.7* macrophages. MY-EH@SS (150 µg/mL) inhibited LPS-induced IκBα phosphorylation (p-IκBα) and degradation, as well as NF-κB p65 phosphorylation (p-p65). Etanercept (ETA) was used as a positive control. Cells were treated as follows: Lane 1, control (Con); Lane 2, LPS; Lane 3, LPS + MY-EH@SS (150 µg/mL); Lane 4, LPS + Etanercept (ETA, positive control). (n) ELISA quantification of TNF-α secretion in the cell culture supernatant of RAW*264.7* macrophages.

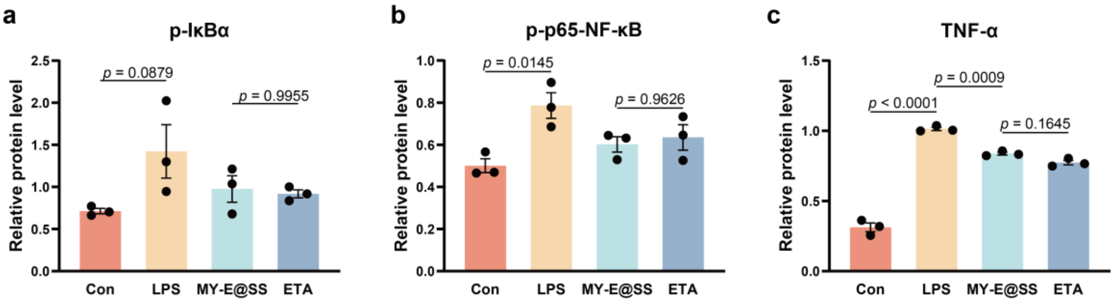

Figure S51. Quantitative analysis of TNF-α/NF-κB pathway inhibition by MY-E@SS in RAW*264.7* macrophages. (a) Quantitative analysis of p-IκBα protein levels normalized to GAPDH. (b) Quantitative analysis of p-p65-NF-κB protein levels normalized to GAPDH. (c) Quantitative analysis of TNF-α protein levels normalized to GAPDH.

These results demonstrate that MY-EH@SS acts directly on macrophages and exerts anti-inflammatory effects by inhibiting the TNF-α/NF-κB pathway, providing direct experimental support for the proposed mechanism of action of the KPV peptide.

We have added that information in our revised manuscript as follows:

Line 507-516: To directly validate this mechanism, *in vitro* studies were conducted in LPS-stimulated RAW*264.7* macrophages, where MY-E@SS treatment was observed to concentration-dependently inhibit LPS-induced p65 nuclear translocation. (Fig. 9l, S49).

Further investigation revealed that at 150 μg/mL, it significantly suppressed TNF-α secretion to a level comparable to the inhibitor etanercept and concurrently reduced IκBα phosphorylation and degradation along with p65 phosphorylation (Fig. 9m, n, S50 and S51). These results establish that MY-EH@SS exerts its anti-inflammatory effect through direct inhibition of the TNF-α/NF-κB signaling pathway. These results demonstrate that MY-E@SS counteracts inflammation through directly inhibiting the TNF-α/NF-κB signaling pathway in immune cells, thereby suppressing downstream cytokine production and inflammatory activation (Fig. 9o).

2. The current assessment of SS-shell degradation and bacterial/peptide release is primarily qualitative. To better understand the delivery kinetics of the bioinspired microcapsule reactor, the authors should provide quantitative data such as *in vivo* degradation rates, diffusion or release modeling, or spatiotemporal tracking using fluorescence or radiolabel imaging.

Response: Thanks for your valuable comment. To precisely resolve the *in vivo* behavior of the MY-E@SS microcapsules, the SS shell and the engineered bacteria were separately labeled with distinct fluorophores (Cy5 for the shell and sfGFP for the bacteria). Following oral administration to IBD model mice, spatiotemporal tracking and quantitative analysis of the fluorescence signals were performed via fluorescence imaging over a period of 72 hours.

(1) Co-localization and Co-delivery of the Shell and Bacteria: Imaging analysis revealed that during the first 8 h after administration, the fluorescence signals of the shell (Cy5) and the bacteria (sfGFP) were highly co-localized in the colonic region (Fig. S17). This sustained co-localization provides direct visual evidence that the SS shell effectively protects and co-transports the engineered bacteria through the upper gastrointestinal tract.

Line 323-331: To precisely delineate the *in vivo* behavior of the microcapsules, we performed dual-fluorescence labeling of the SS shell (Cy5) and the engineered bacteria (sfGFP) for spatiotemporal tracking. Imaging over 72 hours revealed high colocalization of both signals in the colon during the first 8 hours, visually confirming effective protection and co-transport by the shell through the upper GI tract (Fig. S26). The shell signal peaked at 8-12 hours and subsequently decayed, indicating its gradual degradation. Concurrently, bacterial fluorescence in the colon increased markedly and persisted beyond 48 hours, quantitatively confirming effective capsule disintegration followed by bacterial release, proliferation, and sustained colonization *in situ*.

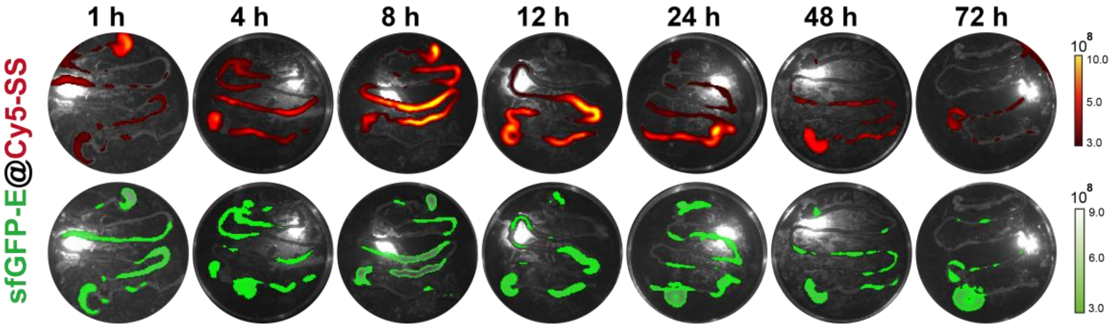

Figure S26. Spatiotemporal fluorescence imaging of sfGFP-E@Cy5-SS microcapsules in IBD model mice. SS shell: Cy5-labeled, red; Engineered bacteria: sfGFP-labeled, green.

(2) Spatiotemporal kinetics of shell degradation: The fluorescence signal from the SS shell peaked within 8–12 h post-administration, followed by a gradual decline (figure above).

(3) Quantitative tracking of bacterial release and colonization: Concurrent with the attenuation of the shell signal, the fluorescence signal of the engineered bacteria (sfGFP) increased markedly in the colonic region and remained elevated for over 48 h. Quantitative analysis of fluorescence intensity in the colon at different time points (presented earlier as a bar chart in the main text) confirmed effective disintegration of the microcapsules in the colon and subsequent in-situ proliferation and sustained retention of the engineered bacteria.

Line 331-335: We further employed the plate counting method to quantitatively assess the colonization dynamics of the engineered bacteria in various segments of the gastrointestinal tract (Fig.6a). Tissue samples from the stomach, small intestine, cecum, and colon were collected at designated intervals following the administration of sfGFP-EcN or sfGFP-E@SS, and bacterial loads were quantified as CFUs (Fig. 6e-i).

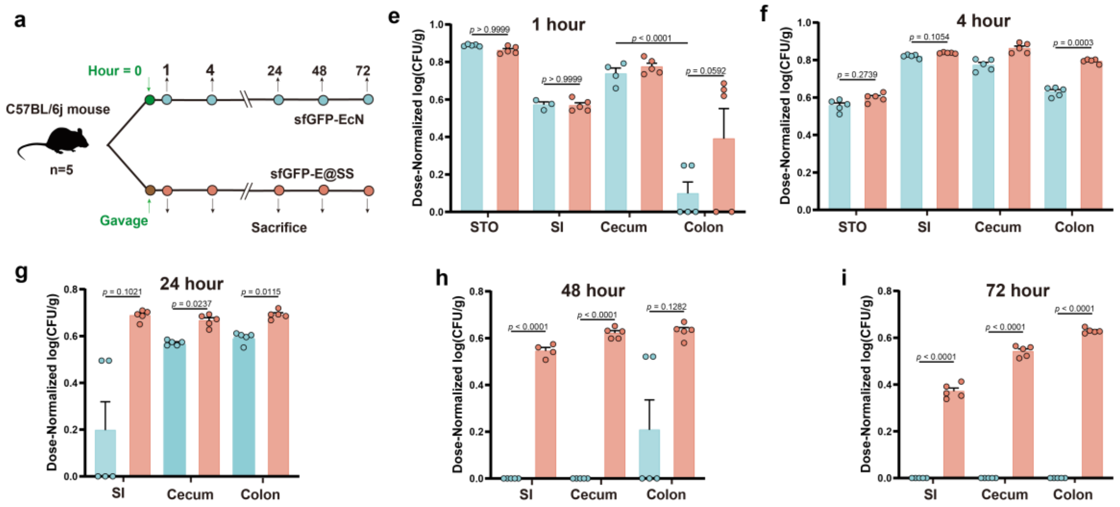

Figure 6. MY-E@SS prolongs the colonization of probiotics and has good biocompatibility. (a) Scheme of the experimental procedure for evaluating probiotic colonization. (e-i) Kinetic profiles of colonizing EcN in different gastrointestinal segments (stomach, small intestine, cecum, colon) at 1, 4-, 24-, 48-, and 72-hours post-administration.

3. The manuscript reports changes in cytokine levels (IL-6, IL-1β, TNF-α, IL-10), but does not examine how MY-E@SS affects specific immune cell populations. It would be valuable to include flow-cytometry or immunofluorescence analyses of macrophage polarization (M1/M2) and regulatory T-cell (Treg) dynamics.

Response: Thanks for your valuable comment. We have supplemented the study with detailed immunophenotypic analyses. Multiplex immunofluorescence staining was performed on colon tissue sections to quantify the two key regulatory immune cell populations highlighted in your comments:

(1) Macrophage polarization status: CD86 (a marker for pro-inflammatory M1 macrophages), along with CD206 and Arg1 (markers for anti-inflammatory/pro-repair M2 macrophages), were co-stained to systematically evaluate the inflammatory/repair balance in the local immune microenvironment (Fig. S18).

(2) Regulatory T cell (Treg) dynamics: Foxp3 (the Treg-specific transcription factor) and CD4 (a helper T cell marker) were labeled to perform relative quantification of Foxp3^{+} CD4^{+} Treg cells, which possess immunosuppressive functions, within the colonic mucosa (Fig. S19).

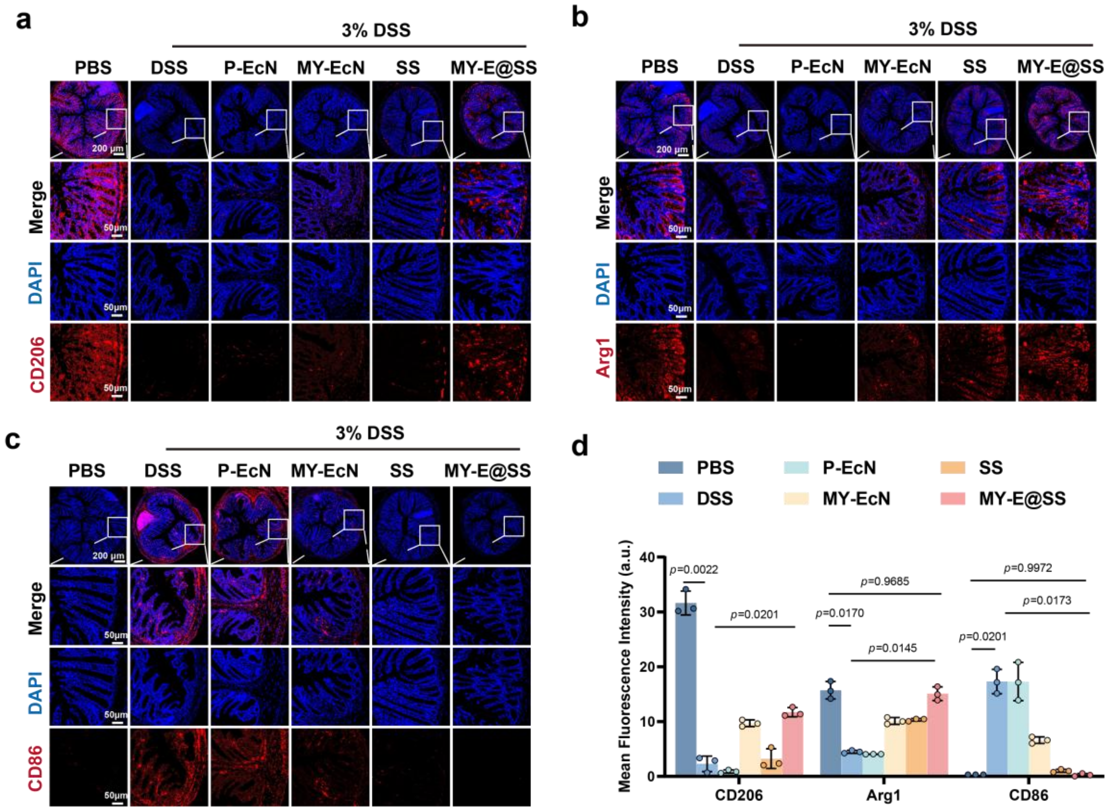

Figure S18. Immunophenotypic analysis of key immune cell subsets in colonic tissues following MY-E@SS treatment. (a–c) Representative immunofluorescence staining images of the M2 macrophage markers CD206 (a) and Arg1 (b), and the M1 macrophage marker CD86 (c) in colon tissues. (d) Quantitative analysis of the relative abundance of M1 (CD86^{+}) and M2 (CD206^{+}/Arg1^{+}) macrophage populations.

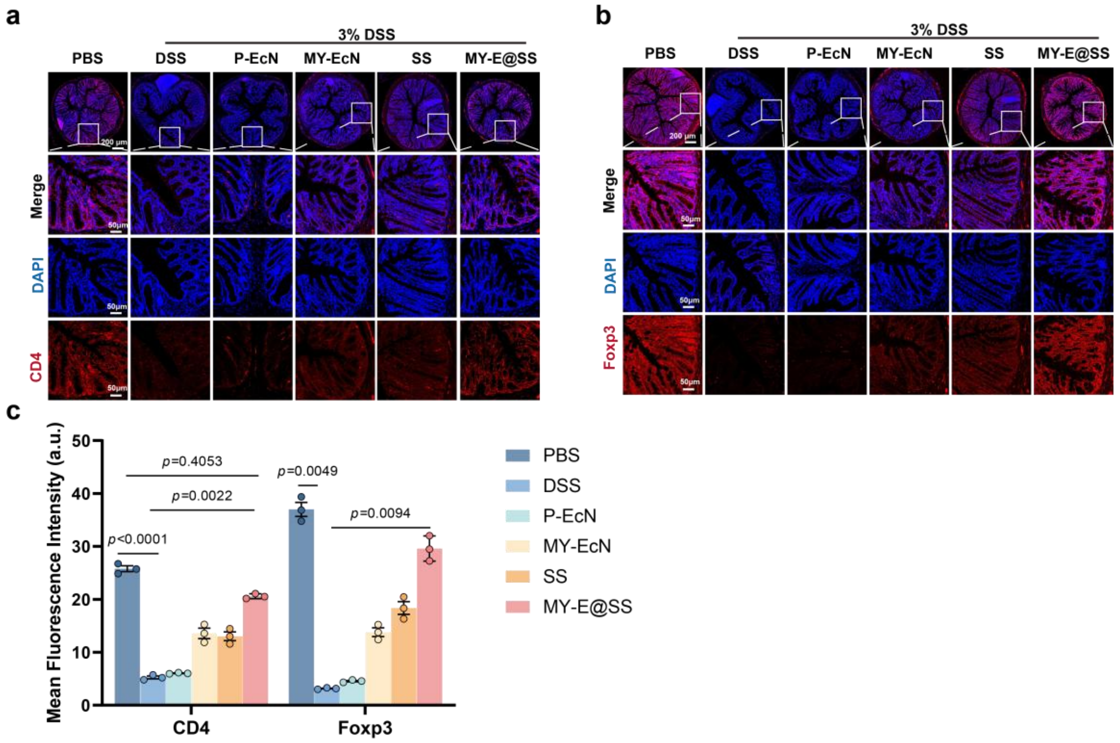

Figure S19. Dynamics of regulatory T cells (Tregs) in the colonic mucosa. (a) Representative immunofluorescence staining of CD4 (a marker for helper T cells) in colon tissues. (b) Representative immunofluorescence staining of Foxp3 (a Treg-specific transcription factor) in colon tissues. (c) Quantitative analysis of the relative abundance and distribution of $Foxp3^{+}CD4^{+}$ regulatory T cells.

The analysis revealed that, compared to the IBD model group, mice treated with MY-E@SS exhibited a significant reduction in the infiltration of $CD86^{+}$ M1 macrophages and a marked increase in the proportion of $CD206^{+}$ and $Arg1^{+}$ M2 macrophages within colon tissue. These results indicate a successful shift in the local macrophage phenotype from a pro-inflammatory state toward an anti-inflammatory and reparative state following treatment. Furthermore, the number of $Foxp3^{+}$ $CD4^{+}$ Treg cells was significantly elevated at colonic inflammatory sites in the MY-E@SS-treated group. This finding is consistent with the previously observed increase in the anti-inflammatory cytokine IL-10, thereby providing key cellular evidence for the immunosuppressive and anti-inflammatory effects mediated by MY-E@SS.

We have added that information in our revised manuscript as follows:

Line 286-289: While MY-EcN or SS alone showed limited efficacy in intestinal repair, MY-E@SS promoted macrophage polarization from a pro-inflammatory ($CD86^{+}$) to an anti-inflammatory/reparative ($CD206^{+}/Arg1^{+}$) phenotype and increased $Foxp3^{+}CD4^{+}$ regulatory T cell infiltration, collectively rebalancing the local immune environment (Fig. 5j).

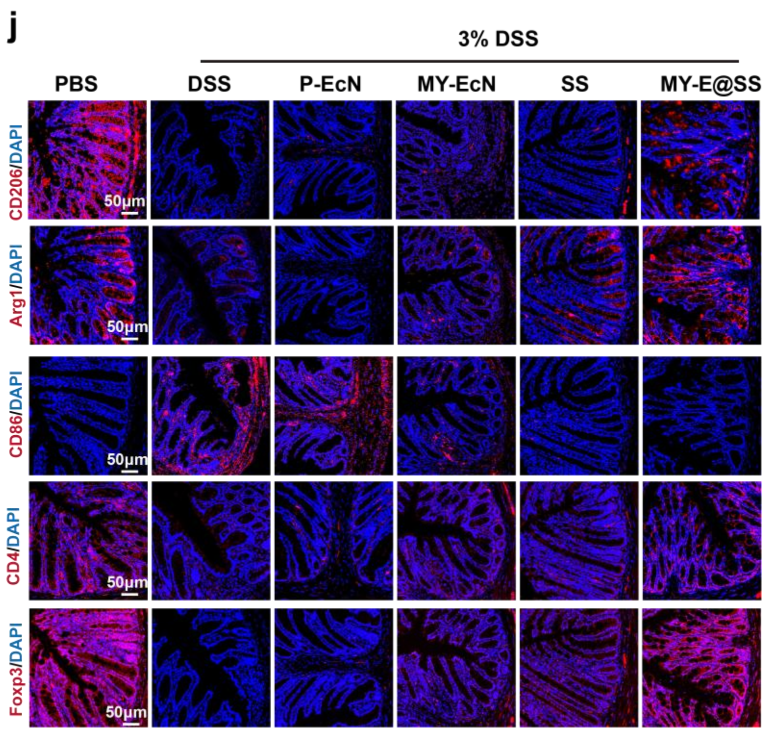

Figure 5 (j) Immunofluorescence analysis reveals MY-E@SS modulates the local colonic immune microenvironment.

4. The expression of the MY protein relies on an L-arabinose–inducible pBAD system, which may not maintain stable induction within the gastrointestinal tract. The authors should quantify MY protein levels in intestinal tissues (e.g., via ELISA or Western blot) to confirm *in vivo* expression, and discuss alternative promoter strategies—such as inflammation-responsive or auto-inducible systems—to enhance stability and predictability under physiological conditions.

Response: Thank you for this insightful and forward-looking suggestion. In light of your comment, we have supplemented the study with the following critical validation experiments and have incorporated a corresponding discussion of the results.

(1) The expression of the MY protein *in vivo* was quantitatively measured. Specifically, colonic contents and colon tissues were collected from mice with DSS-induced colitis 4 hours after oral gavage with the MY-E@SS formulation — a time point selected based on our data on bacterial colonization and drug-release kinetics. MY protein levels were specifically quantified using enzyme-linked immunosorbent assay (ELISA). The results showed that mice in the MY-E@SS treatment group exhibited significantly higher levels of MY protein in colonic contents compared to mice in both the healthy control group and the DSS model group.

Line 156-158: Moreover, elevated MY protein levels in colonic contents of MY-E@SS treated mice (Fig. S5) corroborated effective *in vivo* expression and processing.

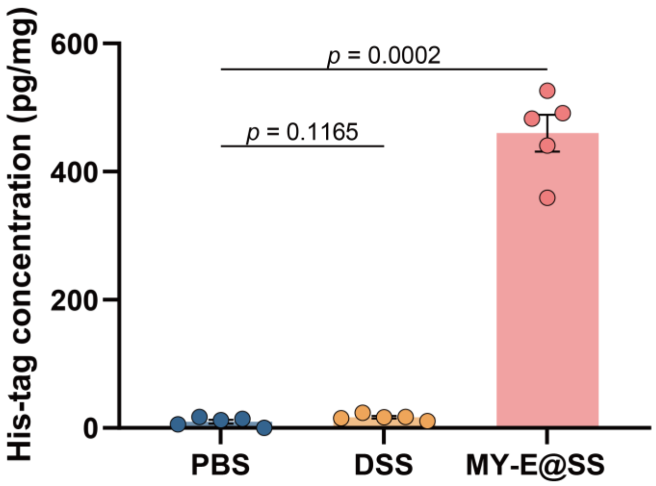

Figure S5. Quantification of MY protein expression levels in colonic contents of mice from each group by ELISA.

(2) Additionally, the discussion section of the revised manuscript has been expanded to specifically address the advantages of alternative promoter strategies.

Line 599-617: This work introduces a biomimetic encapsulation strategy to enhance the environmental tolerance of engineered bacteria and improve their diversity and safety. An inducible priming subsystem is used to increase the production of therapeutic proteins and enable precise controlled release in engineered strains. Concurrently, the release of anti-inflammatory peptides is regulated by the inflammatory response mechanism, thereby reducing the safety risks caused by continuous activation. This bionic encapsulation strategy further strengthens environmental isolation and broadens the operational safety boundary of engineering bacteria. Nevertheless, the off-target effect and horizontal gene transfer risk of engineered bacteria cannot be completely eliminated. In order to address these challenges and promote personalized treatment, future promoter designs can be tailored to the individual characteristics of patients. For example, inflammation-responsive promoters or self-inducible promoters can be utilized. Inflammation-responsive promoters can be specifically activated by overexpressed inflammatory factors (such as TNF-α, IL-6, NO, etc.) in the local microenvironment; the self-inducible promoter realizes the spatiotemporal regulation of gene expression through the bacterial quorum sensing system, ensuring that the therapeutic protein is only released in the target microenvironment, thereby avoiding systemic risks caused by continuous activation. In summary, by integrating biomimetic and synthetic biology strategies, our constructed MY-E@SS successfully achieves intelligent sensing and precise repair of the inflammatory microenvironment. As such, this work provides a novel and translatable feasible strategy for the collaborative management of IBD and other inflammatory diseases.

5. The SS shell incorporates silk fibroin, dopamine, and mannose conjugates, while the MY protein contains highly repetitive RGD/AAPV-KPV tandem sequences (22 repeats). No assessment is provided regarding possible immunogenicity, antibody formation, hypersensitivity, or long-term toxicity associated with these components.

Response: Thank you for your valuable comment. In response, we have systematically

supplemented comprehensive safety evaluation experiments, as detailed below.

Long-term Toxicity Assessment: Beyond the previously conducted 17-day biocompatibility experiment (Fig. 6k–q), to fully investigate potential immune responses, the administration period was extended to 29 days with a high-concentration formulation challenge administered at the end of the experiment (Fig. S31a). Healthy mice were randomly assigned to a PBS control group (negative control), an OVA group (positive control), and the MY-E@SS treatment group. The following parameters were systematically monitored throughout and after the experimental period:

(1) Body Weight and General Condition Observation (Fig. S31b): Body weight in both the PBS and MY-E@SS groups showed a steady increasing trend, with no statistically significant difference between the two groups. Mice in the OVA positive control group exhibited the expected transient weight gain slowdown or fluctuation associated with an immune response, confirming the responsiveness of the experimental model. These results indicate that MY-E@SS administration did not adversely affect normal animal growth and development.

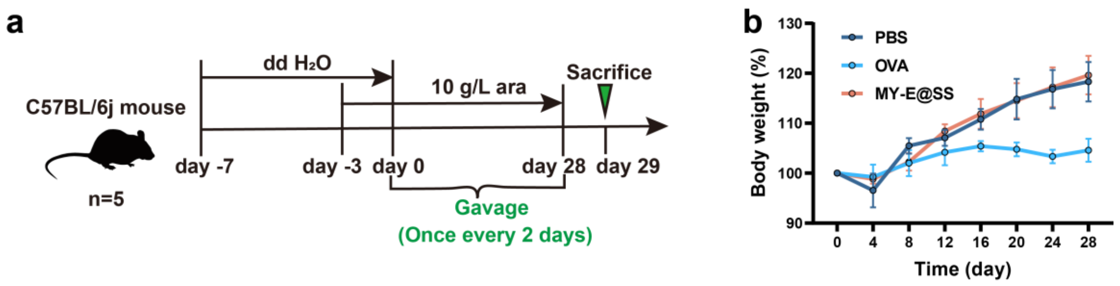

Figure S31. Extended (29-day) *in vivo* biocompatibility and immunogenicity assessment of MY-E@SS in healthy mice. (a) Schematic of experimental design. (b) Body weight changes over the 29-day period.

(2) Systemic Inflammation and Hematological Parameters (Fig. S31d–g): Serum levels of key inflammatory cytokines (TNF-α, IL-1β, IL-6, and IL-10) were measured, and a comprehensive hematological analysis was performed on days 17 and 29 post-administration. The results showed no significant differences in serum inflammatory cytokine levels or major hematological parameters between the MY-E@SS group and the PBS control group, indicating that the formulation did not induce unintended systemic inflammatory responses or hematopoietic dysfunction in healthy subjects.

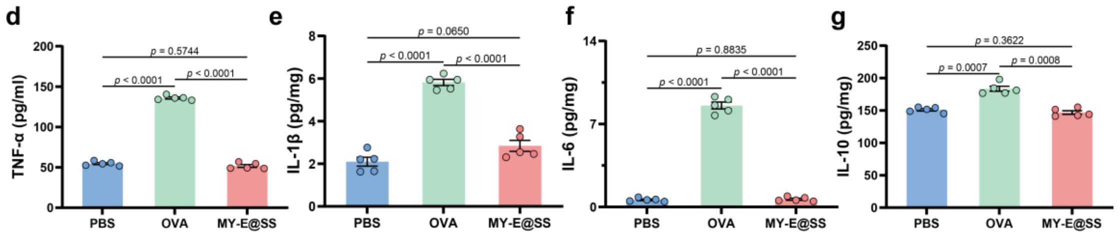

Figure S31 (d–g) Systemic cytokine analysis: serum levels of (d) TNF-α, (e) IL-1β, (f) IL-6, and (g) IL-10.

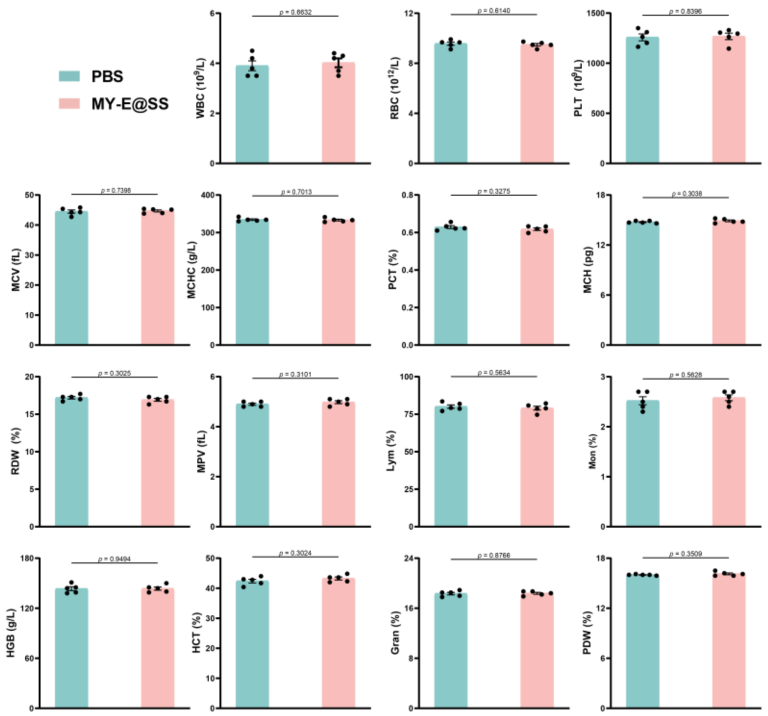

Figure S28. Hematological profiling of normal mice with/without MY-E@SS gavage.

(3) Histopathological Examination of Major Organs (Fig. S31c): H&E staining analysis of the heart, liver, spleen, lungs, and kidneys revealed no treatment-related pathological alterations or inflammatory infiltration.

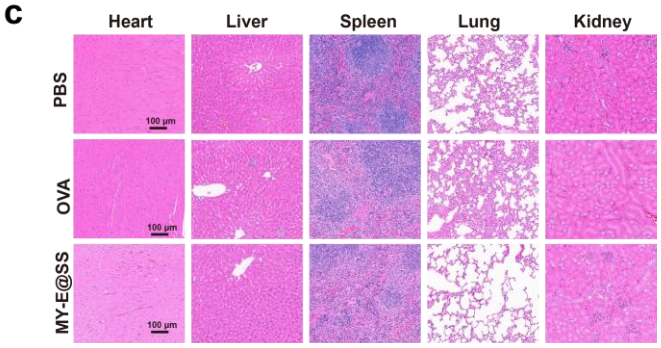

Figure S31 (c) Representative H&E-stained histological images of major organs (heart, liver, spleen, lung, and kidney).

(4) Evaluation of Immunogenicity and Hypersensitivity: Comprehensive assessment revealed the following: Throughout the 29-day experimental period, no allergy-related abnormal behaviors such as scratching or dyspnea were observed in the mice (Fig. S32a). The splenic index showed no significant difference compared with normal mice (Fig. S32b). Serological analysis indicated no significant elevation of specific IgE antibodies in the MY-E@SS group (Fig. S32c). Furthermore, the level of the allergy-associated chemokine MCP-1 in the MY-E@SS group was comparable to that in the PBS control group and significantly lower than that in the OVA-positive control group (Fig. S32d). Histopathological analysis further confirmed the absence of immune cell infiltration in the adipose tissue of the MY-E@SS group, a phenomenon

observed in the OVA-positive control group (Fig. S32e). Collectively, these results demonstrate that MY-E@SS does not induce significant immunogenicity or elicit a notable risk of immediate hypersensitivity under prolonged administration, indicating favorable biocompatibility.

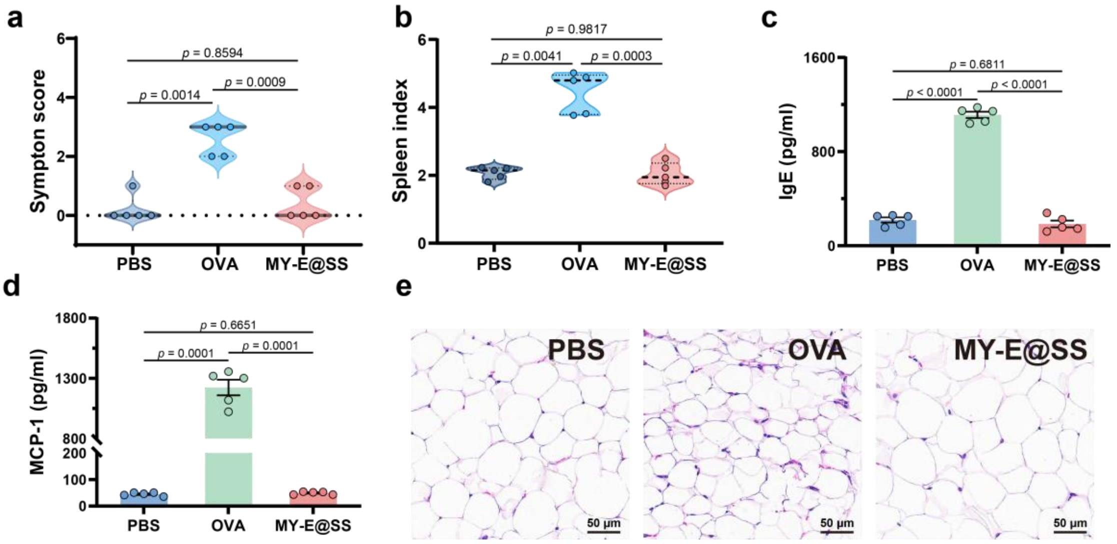

Figure S32. Assessment of immunogenicity and hypersensitivity potential after extended administration of MY-E@SS. (a) Behavioral monitoring record. (b) Spleen index. (c) Serum total IgE antibody levels measured by ELISA on day 29. (d) Serum levels of the allergy-related chemokine MCP-1.

We have added that information in our revised manuscript as follows:

Line 377-386: Encouraged by these findings, we proceeded to conduct a 29-day *in vivo* biocompatibility assessment. Throughout the study, no abnormalities were observed in body weight, survival rate, or inflammatory cytokine levels in the mice (Fig. S31). Serum analysis after the experiment revealed no significant increase in IgE antibodies in the MY-E@SS group, and the level of the allergy-related chemokine MCP-1 remained comparable to that of the PBS control group. Moreover, no allergy-associated behaviors such as scratching or dyspnea were observed during the entire experimental period (Fig. S32). Additionally, the splenic index showed no significant difference compared with that of normal mice. Collectively, these results indicate a low risk of immediate-type hypersensitivity reaction induced by MY-E@SS and demonstrate an absence of apparent immunotoxicity or systemic adverse effects.

Reviewer #3 (Remarks to the Author):

The manuscript describes the development of a core–shell bionic microcapsule system (MY-E@SS) that integrates engineered bacteria with a multifunctional protective shell to treat inflammatory bowel disease (IBD). The concept is innovative, and the preliminary results are promising. However, several major concerns regarding experimental design, mechanistic depth, reproducibility, and clarity of data interpretation must be addressed before the manuscript can be

considered for publication.

Response: We sincerely thank you for your recognition of the novelty and potential of our study, as well as for your valuable comments. These suggestions are crucial for enhancing the rigor and depth of the manuscript. Following your recommendations, we have conducted comprehensive revisions and additions to the manuscript. Below is our point-by-point response to each specific comment.

Major comment

1. The study presents an innovative bionic microcapsule system inspired by coral community defense and repair mechanisms, but the rationale for selecting coral as the biological model should be more rigorously justified. It would strengthen the manuscript to compare this model with alternative biological systems that also exhibit multi-level cooperative responses.

Response: Thank you for your insightful and constructive comments. In the revised introduction, the rationale for selecting coral as the bionic model has been further elaborated. The hierarchical "exoskeleton–mucus–symbiont" cooperative system offers a highly relevant blueprint for addressing the three key challenges in the gastrointestinal delivery of engineered bacteria: survival, colonization, and intelligent response. Unlike rapid plant closure or animal immune cascades, the multi-layered coral defense mechanism more intuitively integrates sustained protection with dynamic responsiveness. Specifically, corals sense and adapt to environmental stress by activating a cascade of defense and repair mechanisms. Their calcareous exoskeleton provides physical protection, while secreted mucus forms a biochemical barrier that resists adverse conditions, enhances adhesion, and contains immune-active components such as antimicrobial peptides to combat infection. Furthermore, coral-associated symbiotic microorganisms strengthen host immunity by scavenging reactive oxygen species and alleviating oxidative stress. This synergistic defense system, refined through long-term evolution, demonstrates remarkable environmental adaptability, thereby providing direct inspiration for the core "defense–sensing–repair" concept of this study and highlighting the innovative bionic nature of the work. In our work, a synthetic bionic shell (SS) was constructed to mimic coral exoskeleton and mucus layers, enabling resistance to gastric acid and promoting intestinal colonization and tissue repair. The bionic microcapsule encapsulates engineered bacteria (MY-EcN), which express the MY protein designed to achieve controlled release of the anti-inflammatory peptide KPV in the inflammatory gut environment. This mechanism emulates the danger-sensing–response strategy observed in corals. Thus, the coral survival system aligns closely with the bionic design principles applied here.

We have added that information in our revised manuscript as follows (marked in red):

Line 72-101: In bionic design, we selected coral as the model because its "exoskeleton–mucus–symbiont" hierarchical cooperative architecture provides a highly compatible blueprint for addressing the synergistic challenges of probiotic survival, colonization, and inflammatory

response in oral delivery. Compared to other stress-responsive systems (e.g., rapid closure in plants or the immune cascade in animals), this model more intuitively integrates the dual functions of long-term protection and intelligent response, directly inspiring the core "defense protection–danger sensing–tissue repair" structure of this work.

Inspired by the adaptive mechanisms of corals, we have engineered a bionic microcapsule reactor (MY-E@SS) through the integration of synthetic biology and biomimetic principles. It operates by implementing a biomimetic "defense protection–danger sensing–tissue repair" cascade, mimics the coral survival strategy, for the treatment of IBD (Fig. 1). MY-E@SS was fabricated via droplet microfluidics, with its core–shell structure comprising a biomimetic shell (SS) and engineered bacteria (MY-EcN) encapsulated within it. The MY protein expressed in MY-EcN was designed with a tandem peptide structure. This construct contains an Arg-Gly-Asp (RGD) sequence, which enhances cell adhesion by binding to integrin receptors on intestinal epithelial cells.[25, 26] Leveraging this property, the MY protein can be directed to target intestinal epithelial cells. Alongside the RGD motif, the protein was designed to include the inflammation-sensitive Ala-Ala-Pro-Val (AAPV) motif and the anti-inflammatory peptide Lys-Pro-Val (KPV). In the inflammatory microenvironment, the AAPV motif is specifically cleaved by the abundant neutrophil elastase (NE).[25-27] Thus, within the inflammatory colon, the AAPV-KPV tandem linkage is cleaved, thereby triggering the controlled release of KPV-a process that emulates the integrated danger-sensing and defense response of corals. To mimic the structure of the coral exoskeleton and mucus layer, the SS shell was synthesized via a one-pot chemical method based on silk fibroin (SF), dopamine, and mannose. Dopamine provides additional adhesive properties, while mannose confers targeting functionality; together, they enable the SS shell to scavenge reactive oxygen and nitrogen species (RONS). Furthermore, the SS shell enhances the aggregation density of engineered bacteria and works synergistically with MY-EcN to promote tissue repair, thereby simulating the coral-derived defense–tissue repair process. This design constitutes a synergistic therapy: the shell provides protection and targeted delivery, while the engineered bacteria enable sensing-triggered KPV release. Their combination ensures effective bacterial delivery and yields therapeutic outcomes that surpass the efficacy of either component alone.

2. The design principles of the MY-E@SS platform are conceptually compelling, yet the engineering logic behind each component of the core–shell structure could be described more systematically. In particular, the manuscript should clearly articulate how each structural layer contributes to gastric protection, targeted release, and synergistic therapeutic performance.

Response: Thank you for your valuable suggestions. As you rightly pointed out, a systematic elucidation of the engineering logic linking each component of the core-shell structure to its functional outcomes is essential to highlight the innovative design and scientific rigor of this work. Accordingly, we have supplemented and revised the manuscript to provide a clearer explanation of the design rationale and functional contribution of each structural layer within the MY-E@SS platform. Additionally, the experimental section in the main text now includes a series of validation experiments to further confirm the functional realization of each structural unit.

Line 79-101: Inspired by the adaptive mechanisms of corals, we have engineered a bionic microcapsule reactor (MY-E@SS) through the integration of synthetic biology and biomimetic principles. It operates by implementing a biomimetic "defense protection – danger sensing – tissue repair" cascade, mimics the coral survival strategy, for the treatment of IBD (Fig. 1). MY-E@SS was fabricated via droplet microfluidics, with its core – shell structure comprising a biomimetic shell (SS) and engineered bacteria (MY-EcN) encapsulated within it. The MY protein expressed in MY-EcN was designed with a tandem peptide structure. This construct contains an Arg-Gly-Asp (RGD) sequence, which enhances cell adhesion by binding to integrin receptors on intestinal epithelial cells.[25, 26] Leveraging this property, the MY protein can be directed to target intestinal epithelial cells. Alongside the RGD motif, the protein was designed to include the inflammation-sensitive Ala-Ala-Pro-Val (AAPV) motif and the anti-inflammatory peptide Lys-Pro-Val (KPV). In the inflammatory microenvironment, the AAPV motif is specifically cleaved by the abundant neutrophil elastase (NE).[25-27] Thus, within the inflammatory colon, the AAPV-KPV tandem linkage is cleaved, thereby triggering the controlled release of KPV-a process that emulates the integrated danger-sensing and defense response of corals. To mimic the structure of the coral exoskeleton and mucus layer, the SS shell was synthesized via a one-pot chemical method based on silk fibroin (SF), dopamine, and mannose. Dopamine provides additional adhesive properties, while mannose confers targeting functionality; together, they enable the SS shell to scavenge reactive oxygen and nitrogen species (RONS). Furthermore, the SS shell enhances the aggregation density of engineered bacteria and works synergistically with MY-EcN to promote tissue repair, thereby simulating the coral-derived defense – tissue repair process. This design constitutes a synergistic therapy: the shell provides protection and targeted delivery, while the engineered bacteria enable sensing-triggered KPV release. Their combination ensures effective bacterial delivery and yields therapeutic outcomes that surpass the efficacy of either component alone.

3. The characterization of bacterial survival, colonization efficiency, and functional peptide release is insufficiently quantified. Additional data on stability under gastrointestinal conditions, long-term viability, and *in vivo* colonization dynamics would support the claimed advantages.

Response: Thank you for your insightful suggestion. A series of additional experiments has been conducted, and the results are detailed below:

(1) Quantification of bacterial viability within microcapsules in simulated gastrointestinal fluids (SGF/SIF): We have performed supplementary experiments to quantify the viability of encapsulated bacteria. The results demonstrate that after 1.5 hours of exposure to simulated gastric fluid (SGF), uncoated MY-EcN was almost completely inactivated, whereas MY-E@SS maintained full proliferative capacity. Similarly, in simulated intestinal fluid (SIF), MY-E@SS exhibited significantly superior performance compared to free bacteria (Figure 4k, l). These findings indicate that the SS shell effectively protects against the harsh gastrointestinal environment, substantially enhancing the survival and proliferation potential of the probiotic bacteria.

Line 241-248: Further evaluation was performed to assess the effect of SS encapsulation on the biological function of the probiotic. While both strains grew similarly under standard

conditions (Fig. 4j), MY-E@SS proved vastly superior in simulated gastrointestinal environments. Exposure to simulated gastric fluid (SGF) almost entirely inactivated the unencapsulated MY-EcN (Fig. 4k), and simulated intestinal fluid (SIF) markedly inhibited its growth (Fig. 4l). In contrast, MY-E@SS retained full growth capacity after both treatments. Collectively, the SS coating effectively shields the probiotic from harsh gastrointestinal conditions, ensuring significantly improved survival and proliferative potential.

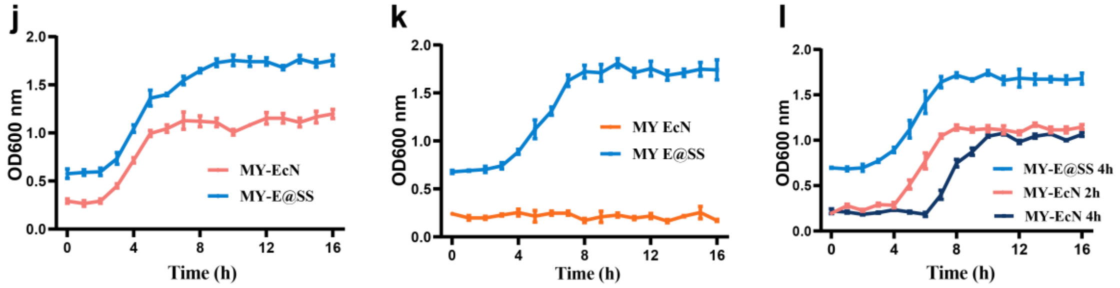

Figure 4 (j) Bacterial growth curves under standard culture conditions. (k) Growth profiles after 1.5 h of simulated gastric digestion. (l) Growth profiles after 2 h of simulated intestinal digestion.

(2) To **further elucidate the *in vivo* stability and delivery kinetics of MY-E@SS**, the SS shell and the engineered bacteria were labeled with Cy5 and sfGFP, respectively, enabling dual-fluorescence tracking. Spatiotemporal dynamic imaging was conducted over 72 hours in an IBD mouse model. The results showed a high degree of colocalization between the shell and bacterial fluorescence signals within the first 8 hours post-gavage, providing direct visual confirmation that the SS shell effectively protects and co-delivers the engineered bacteria through the upper gastrointestinal tract (Figure S26). The shell signal peaked between 8–12 hours and subsequently diminished, indicating its progressive degradation. Concurrently, the bacterial fluorescence signal in the colon increased significantly and was sustained for over 48 hours. These data quantitatively verify the dynamic process of targeted capsule disintegration, bacterial release, *in situ* proliferation, and sustained colonization at the intended site.

Line 324-331: To precisely delineate the *in vivo* behavior of the microcapsules, we performed dual-fluorescence labeling of the SS shell (Cy5) and the engineered bacteria (sfGFP) for spatiotemporal tracking. Imaging over 72 hours revealed high colocalization of both signals in the colon during the first 8 hours, visually confirming effective protection and co-transport by the shell through the upper GI tract (Fig. S26). The shell signal peaked at 8-12 hours and subsequently decayed, indicating its gradual degradation. Concurrently, bacterial fluorescence in the colon increased markedly and persisted beyond 48 hours, quantitatively confirming effective capsule disintegration followed by bacterial release, proliferation, and sustained colonization *in situ*.

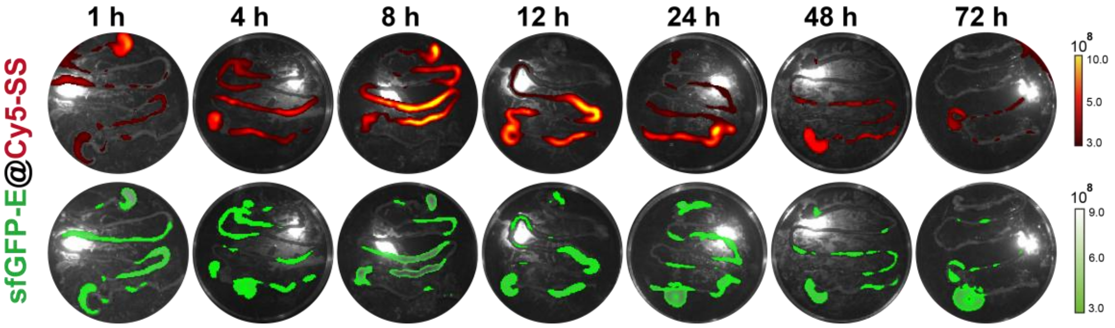

Figure S26. Spatiotemporal fluorescence imaging of sfGFP-E@Cy5-SS microcapsules in IBD model mice. SS shell: Cy5-labeled, red; Engineered bacteria: sfGFP-labeled, green.

(3) Furthermore, **the colonization dynamics of the engineered bacteria in various gastrointestinal segments (stomach, small intestine, cecum, and colon) were quantitatively assessed** using the plate colony-counting method (Fig. 6a). The results demonstrate that MY-E@SS exhibited a significantly higher colonization load in the colon compared to free bacteria, with a prolonged retention time extending to 72 hours (Fig. 6e-i). These microbiological data quantitatively confirm the enhanced colon-targeting capability and long-term colonization conferred by the SS capsule.

Line 331-335: We further employed the plate counting method to quantitatively assess the colonization dynamics of the engineered bacteria in various segments of the gastrointestinal tract (Fig.6a). Tissue samples from the stomach, small intestine, cecum, and colon were collected at designated intervals following the administration of sfGFP-EcN or sfGFP-E@SS, and bacterial loads were quantified as CFUs (Fig. 6e-i).

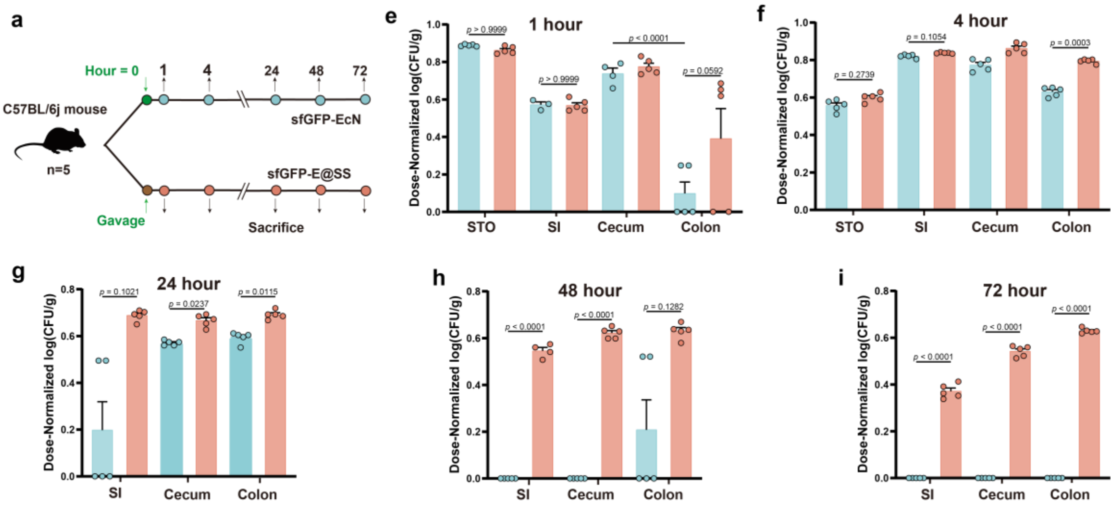

Figure 6 (a) Scheme of the experimental procedure for evaluating probiotic colonization. (e-i) Kinetic profiles of colonizing EcN in different gastrointestinal segments (stomach, small intestine, cecum, colon) at 1, 4-, 24-, 48-, and 72-hours post-administration.

(4) To **quantitatively evaluate the local release of the functional peptide,** colon contents were collected at 0, 1, 4, 8, and 12 hours after the oral gavage of MY-E@SS, and KPV concentration was quantified by liquid chromatography – tandem mass

spectrometry (LC-MS/MS) (Fig. S4a). The results indicate that the KPV concentration peaked at 4 hours and subsequently declined gradually, which aligns with the colonization cycle of the engineered bacteria in the colon. This provides direct evidence that the NE enzyme can cleave the MY protein and release KPV *in vivo*. Furthermore, LC-MS/MS quantification was performed on contents collected from the stomach, small intestine, cecum, and colon 4 hours post-gavage (Fig. S4b). The analysis revealed that KPV was predominantly enriched in the colon, while it was nearly undetectable in the cecum, stomach, and small intestine. This demonstrates that the release of KPV is inflammation-targeted, with the site of action consistent with the intended design.

Line 154-156: To further confirm the *in vivo* release and localization of KPV, we monitored its concentration in colonic contents over time. The levels peaked at 4 h post-administration (Fig. S4a), and specific accumulation in the colon was verified (Fig. S4b).

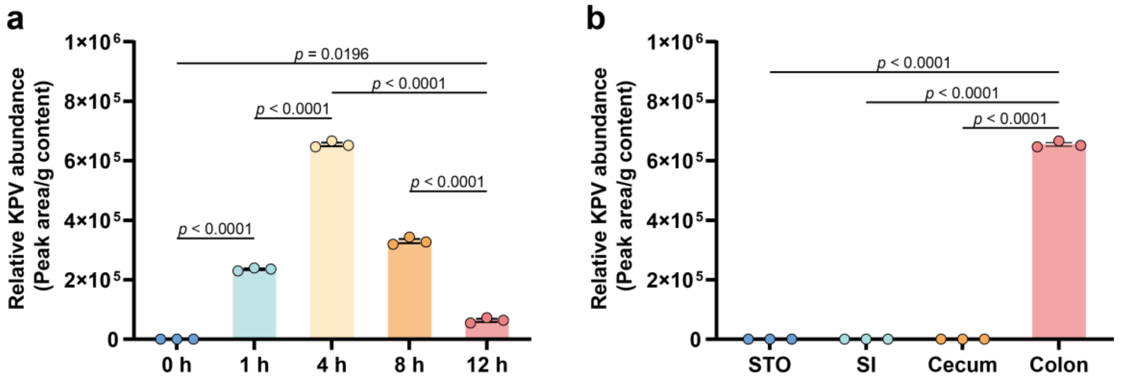

Figure S4. Temporal and distribution analysis of active peptide KPV released by MY-E@SS in a DSS-induced IBD mouse model. (a). Time-course changes in KPV concentration in colonic contents. (b). Distribution of KPV in the contents of different gastrointestinal segments at 4 h post-gavage.

4. While the study demonstrates therapeutic effects in an IBD model, the experimental design would benefit from the inclusion of more rigorous controls, especially comparisons with non-bionic engineered bacteria and traditional probiotic formulations. This would help clarify whether the therapeutic benefits arise primarily from the engineered strain, the microcapsule design, or their synergy.

Response: Thank you for your valuable comment. We fully agree that rigorous control groups are essential to delineate the specific contributions of each component to the therapeutic outcome. The experimental design of this study systematically incorporated multiple key control groups, precisely to clarify whether the observed therapeutic benefits originated from the engineered bacterial strain, the microencapsulation design, or their synergistic interaction. Specifically, the core *in vivo* pharmacodynamic experiments included the following control groups:

(1) Empty vector-engineered bacteria (P-EcN): Serving as a baseline probiotic control to assess the inherent effects of the bacterial chassis.

(2) Therapeutic protein-expressing but unencapsulated bacteria (MY-EcN): Used to isolate and evaluate the contribution of genetic engineering alone.

(3) Empty microcapsules (SS): To rule out potential biological effects arising solely from the capsule material itself.

These controls were consistently implemented across multiple efficacy evaluations, including those presented in the main figures assessing ***Therapeutic efficacy of MY-E@SS for IBD*** (Fig. 5), ***MY-E@SS Ameliorates Respiratory Metabolism in Mice with IBD*** (Fig. 7), ***MY-E@SS regulated colon microflora in IBD mice*** (Fig. 8), ***Fecal occult blood test results from different experimental groups*** (Fig.S15), ***Representative images showing spleen size and morphology across experimental groups*** (Fig.S16), ***Assessment of tight junction protein expression based on immunofluorescence staining and quantitative analysis*** (Fig.S17), ***Immunophenotypic analysis of key immune cell subsets in colonic tissues following MY-E@SS treatment*** (Fig.S18). ***Dynamics of regulatory T cells (Tregs) in the colonic mucosa*** (Fig.S19), ***Hematoxylin and eosin (H&E)-stained sections of major organs demonstrating systemic biocompatibility*** (Fig.S20), ***Hematological parameters in mice from different treatment groups*** (Fig.S21), ***Quantitative analysis of neutrophil elastase (NE) expression and neutrophil infiltration at the inflammatory site*** (Fig.S22), ***Changes in serum oxidative stress markers in IBD mice after different interventions*** (Fig.S23), ***Therapeutic efficacy of MY-E@SS in a TNBS - induced IBD model*** (Fig.S24), ***Quantitative analysis of the restorative effects of various treatment groups on key circadian parameters of respiratory metabolism*** (Fig.S33), and ***Compositional analysis of gut microbiota across treatment groups*** (Fig.S35). The integrated analysis of results from these control experiments conclusively demonstrates that the significant therapeutic efficacy is primarily dependent on the synergistic enhancement between the therapeutic function of the engineered bacteria and the protective delivery afforded by the microcapsules, rather than on any single component alone.

We sincerely appreciate your insightful suggestion. We have revised the manuscript to articulate the rationale and results of this control design more explicitly, which mainly includes, for example:

Line 286-291: While MY-EcN or SS alone showed limited efficacy in intestinal repair, MY-E@SS promoted macrophage polarization from a pro-inflammatory ($CD86^+$) to an anti-inflammatory/reparative ($CD206^+$/$Arg1^+$) phenotype and increased $Foxp3^+CD4^+$ regulatory T cell infiltration, collectively rebalancing the local immune environment (Fig. 5j, S18 and S19). Furthermore, all groups showed no significant differences in systemic organ indices or hematological parameters compared to the PBS control, indicating no obvious systemic toxicity and confirming favorable biosafety (Fig. S20 and S21).

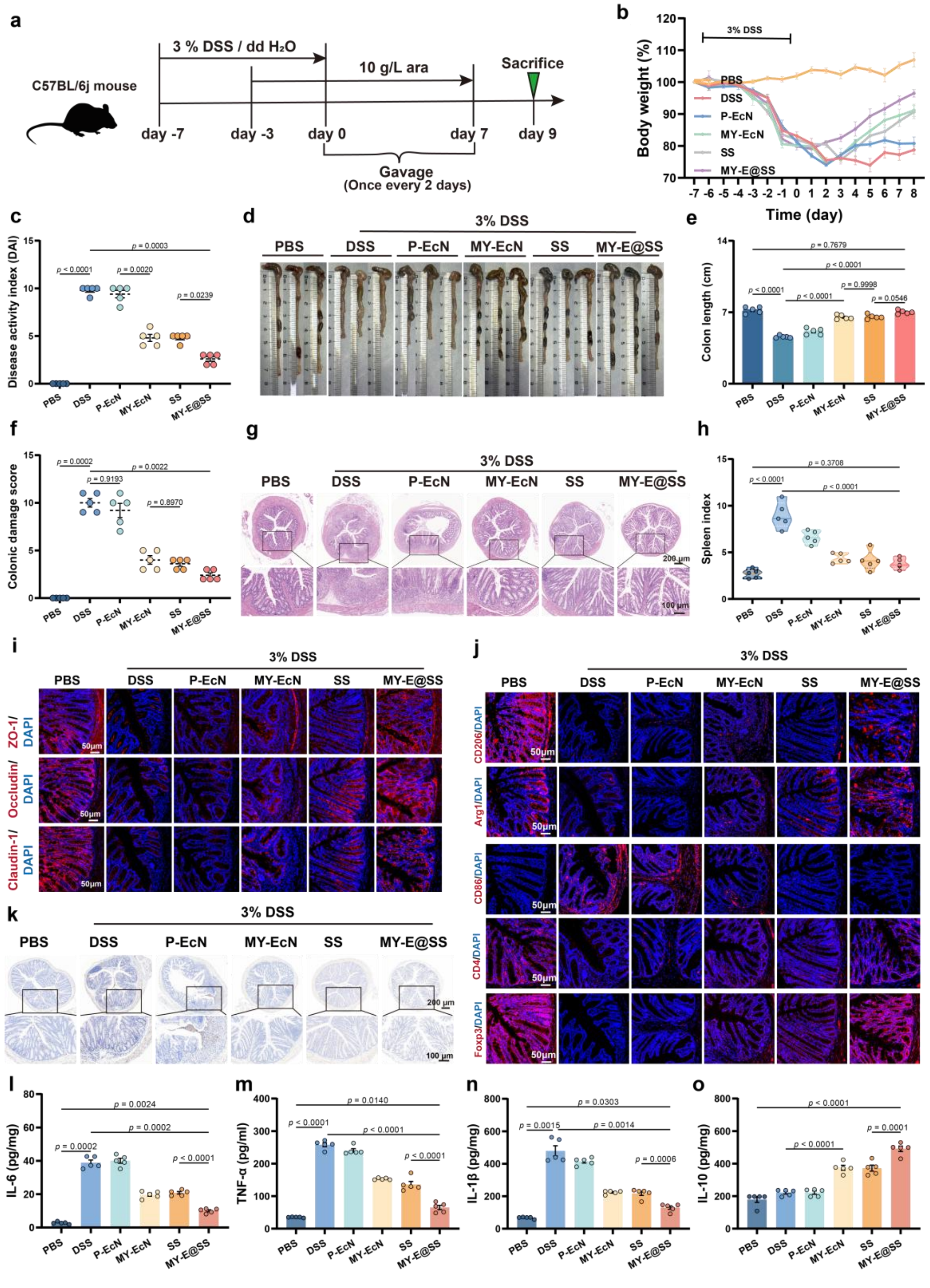

Figure 5. Therapeutic efficacy of MY-E@SS in a DSS-induced murine IBD model. (a) Experimental timeline. C57BL/6 mice were administered 3% DSS in drinking water from day -7 to day 0. From day 0 to day 7, mice received daily oral gavage of PBS, P-ECN, MY-ECN, SS, or MY-E@SS (1×10^8 CFU). (b) Body weight changes and (c) disease activity index (DAI) throughout the experimental period. (d) Representative colon images, (e) colon lengths, (f) Spleen index (spleen weight/body weight). (g) Colonic histopathological scores. (h) Representative Hematoxylin and eosin (H&E)-stained sections of colon tissues. (i) Immunofluorescence staining of key tight junction

proteins in transverse colon sections. (j) Immunofluorescence analysis reveals MY-E@SS modulates the local colonic immune microenvironment. (k) Myeloperoxidase (MPO) staining in colon sections. (l-o) Cytokine levels (IL-6, TNF-α, IL-1β, IL-10) in colon tissues measured by ELISA. All samples were collected on day 9.

Line 401-416: After intervention, the MY-EcN, SS, and MY-E@SS groups exhibited substantial recovery, characterized by restored respiratory metabolic parameters, stabilized circadian rhythms, and enhanced fuel utilization (Fig. 7g-j and S33). Notably, the MY-E@SS group showed superior recovery across multiple metrics, outperforming each component alone, indicating a synergistic effect between MY-EcN and SS in promoting energy metabolism recovery.

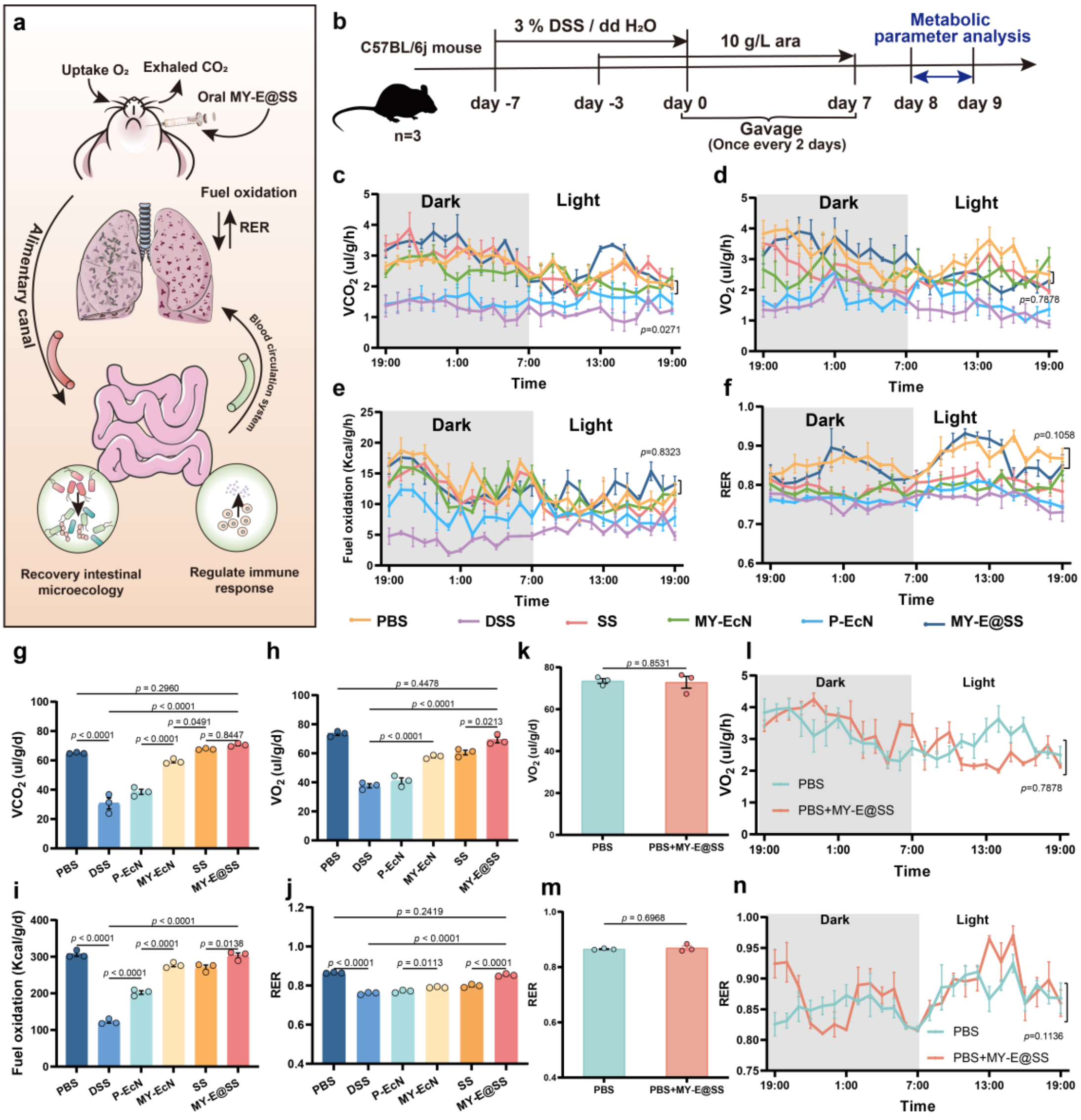

Figure 7. MY-E@SS restores respiratory metabolism in a murine model of IBD. (a) Schematic illustration of altered respiratory metabolism in mice with acute inflammation. (b) Experimental design for the analysis of metabolic parameters. (c-f) Dynamic changes in VCO_2, VO_2, fuel oxidation rate, and the RER in each group on day 7 post-treatment. (g-j) Total 24-hour cumulative VCO_2, VO_2, fuel oxidation, and RER in each group on day 7 post-treatment. (k, l) Total 24-hour

oxygen consumption (k) and dynamic VO_2 curves (l) in healthy control mice following 16 days of MY-E@SS gavage. (m, n) Total 24-hour cumulative RER (m) and dynamic RER curves (n) in healthy control mice after 16 days of gavage.

Line 445-449: While individual treatments of MY-EcN and SS enhanced microbial richness, the MY-E@SS group showed significantly higher Shannon and Simpson indices than the DSS group and all other treatment groups (Fig. 8c, d). Conversely, the P-EcN group exhibited values comparable to the DSS group, indicating a minimal effect on restoring microbial diversity in the compromised gut.

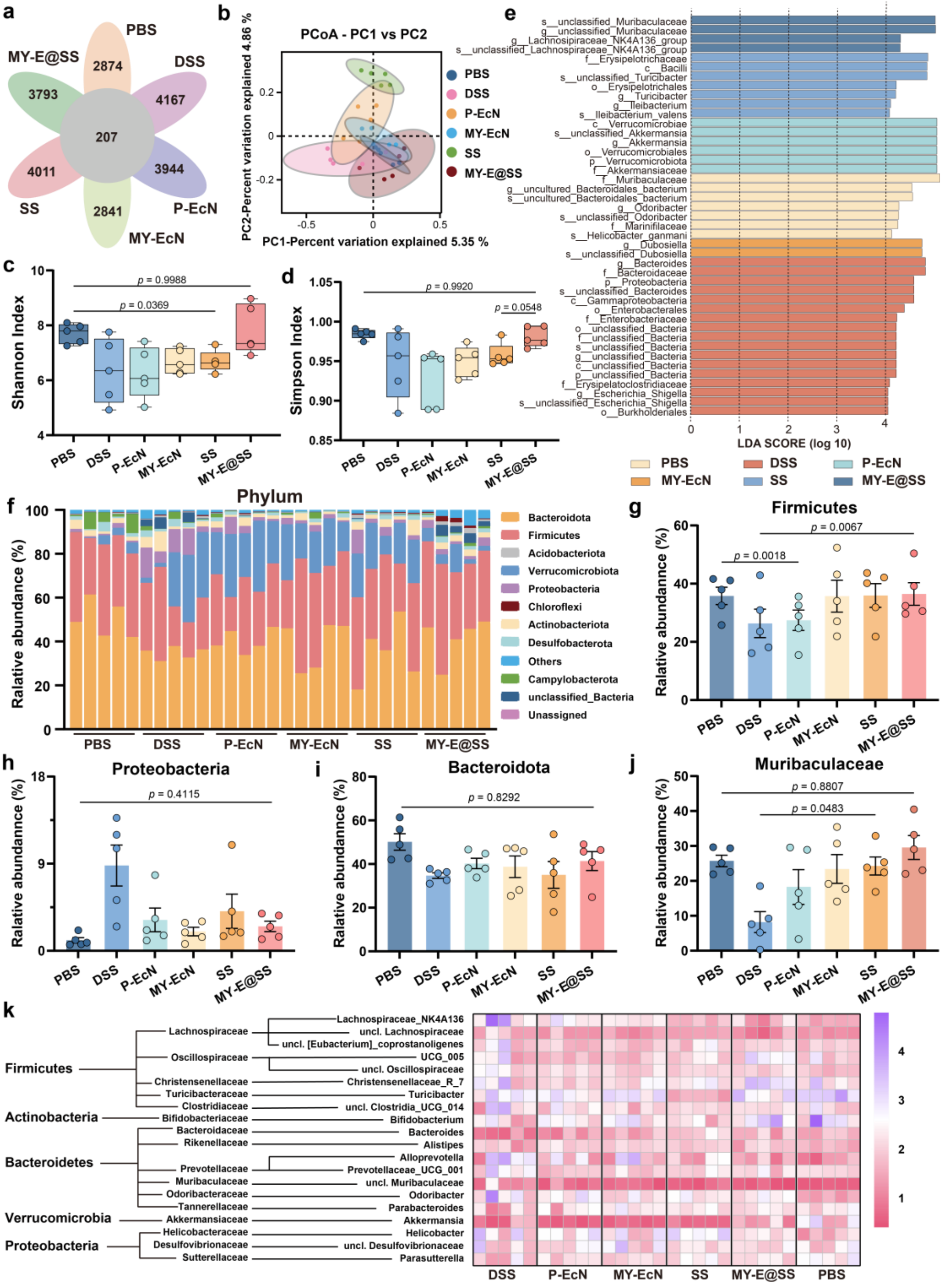

Figure 8. MY-E@SS alleviates gut microbiota dysbiosis in IBD mice. (a) Venn diagram illustrating the overlap of operational taxonomic units (OTUs) across different treatment groups. (b) Beta-diversity analysis visualized using principal coordinate analysis (PCoA). (c, d) Alpha-diversity indices assessed by the Shannon (c) and Simpson (d) indices. (e) Distribution of linear discriminant analysis (LDA) scores. (f) Stacked bar plot showing the relative abundance of the top 12 bacterial taxa at the phylum level. (g-j) Relative abundances of key microbial taxa: Firmicutes (g), Proteobacteria (h), Bacteroidota (i), and Muribaculaceae (j). (k) Heatmap displaying differentially abundant taxa at the family level.

5. The mechanistic analysis—particularly regarding gene expression changes and modulation of arachidonic acid metabolism—should be expanded. The current evidence supports association but not causality. Additional validation experiments, such as inhibition or rescue assays, would enhance mechanistic clarity.

Response: Thanks for your valuable comment. To provide direct evidence at the cellular signaling pathway level, two *in vitro* experiments were supplemented.

(1) Inhibition of NF-κB p65 nuclear translocation in RAW*264.7* macrophages: using an LPS-induced inflammatory model in RAW*264.7* macrophages, the nuclear translocation of the p65-NF-κB subunit was examined. Compared with the LPS-treated group, co-treatment with MY-EH@SS concentration-dependently suppressed the nuclear migration of p65 (Fig. 9l, S49), directly demonstrating its ability to block NF-κB transcriptional activation.

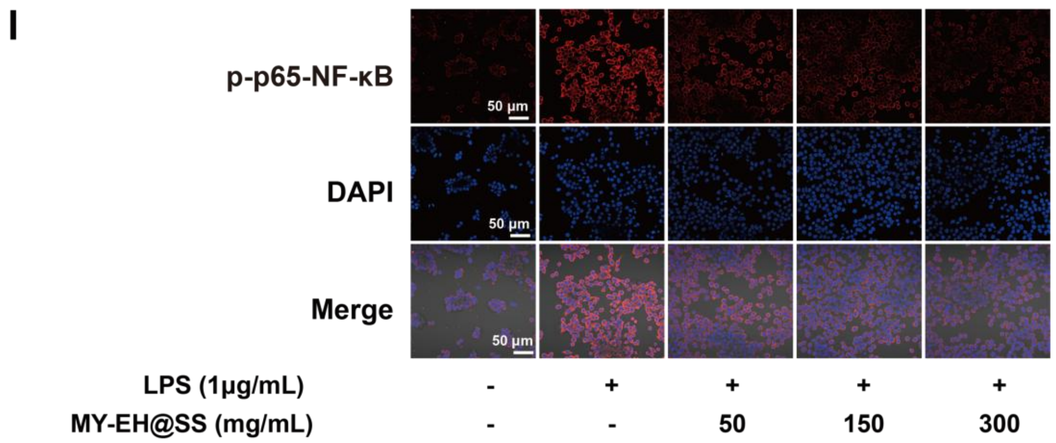

Figure 9 (l) Representative confocal microscopy images showing the inhibition of LPS-induced NF-κB p65 nuclear translocation in RAW*264.7* macrophages by MY-EH@SS. Nuclei were stained with DAPI (blue), and p65 was immunofluorescently labeled (red).

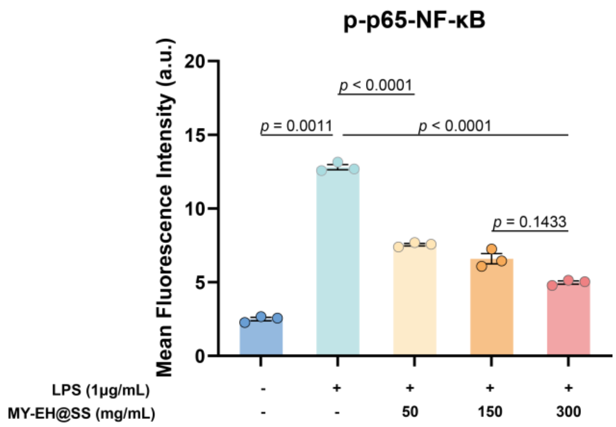

Figure S49. Quantification of p65 nuclear translocation from confocal images.

(2) To **further validate the effect**, 150 μg/mL of MY-EH@SS was used. This treatment significantly suppressed IκBα phosphorylation and degradation, and reduced p65 phosphorylation (Fig. 9m, S51). It also effectively inhibited LPS-induced TNF-α secretion to an extent comparable to the TNF-α inhibitor etanercept (Fig. 9n). Together, these results demonstrate that MY-EH@SS blocks the canonical NF-κB pathway by inhibiting upstream IκBα phosphorylation, thereby attenuating inflammatory cytokine production.

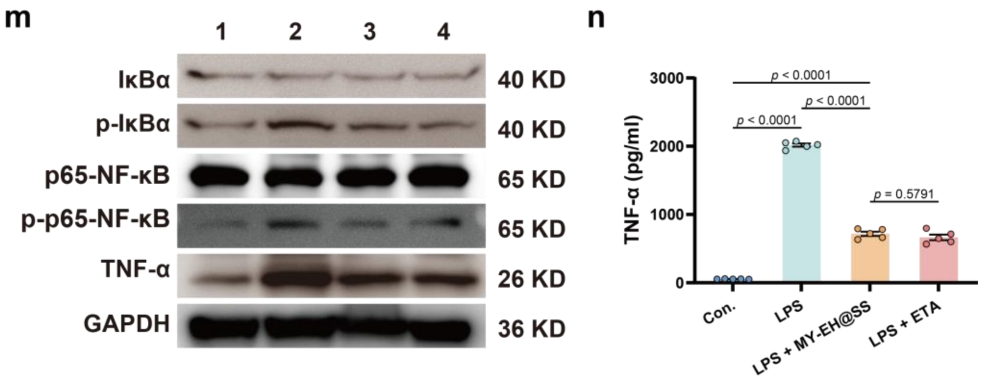

Figure 9. (m) Western blot analysis of key proteins in the TNF-α/NF-κB pathway in RAW*264.7* macrophages. MY-EH@SS (150 μg/mL) inhibited LPS-induced IκBα phosphorylation (p-IκBα) and degradation, as well as NF-κB p65 phosphorylation (p-p65). Etanercept (ETA) was used as a positive control. Cells were treated as follows: Lane 1, control (Con); Lane 2, LPS; Lane 3, LPS + MY-EH@SS (150 μg/mL); Lane 4, LPS + Etanercept (ETA, positive control). (n) ELISA quantification of TNF-α secretion in the cell culture supernatant of RAW*264.7* macrophages.

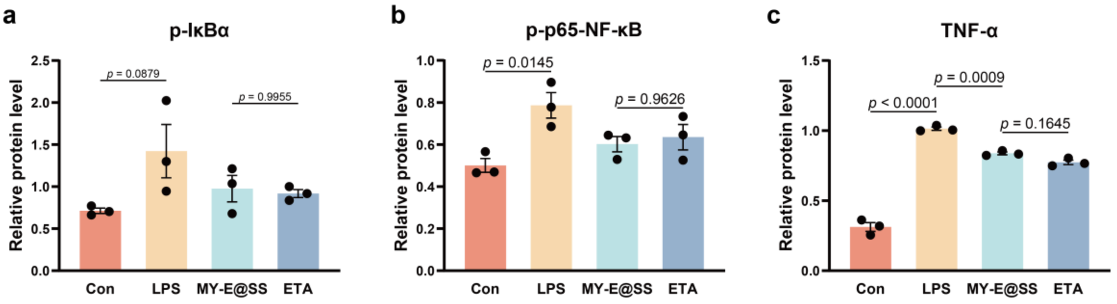

Figure S51. Quantitative analysis of TNF-α/NF-κB pathway inhibition by MY-E@SS in RAW*264.7* macrophages. (a) Quantitative analysis of p-IκBα protein levels normalized to GAPDH. (b) Quantitative analysis of p-p65-NF-κB protein levels normalized to GAPDH. (c) Quantitative analysis of TNF-α protein levels normalized to GAPDH.

These results demonstrate that the bioinspired microcapsule reactor acts directly on macrophages and exerts anti-inflammatory effects by inhibiting the TNF-α/NF-κB pathway, providing direct experimental support for the proposed mechanism of action of the KPV peptide.

We have added that information in our revised manuscript as follows:

Line 507-516: To directly validate this mechanism, *in vitro* studies were conducted in LPS-stimulated RAW*264.7* macrophages, where MY-E@SS treatment was observed to concentration-dependently inhibit LPS-induced p65 nuclear translocation. (Fig. 9l, S49). Further investigation revealed that at 150 μg/mL, it significantly suppressed TNF-α secretion to a level comparable to the inhibitor etanercept and concurrently reduced IκBα phosphorylation and degradation along with p65 phosphorylation (Fig. 9m, n, S50 and S51). These results establish that the bioinspired microcapsule reactor exerts its anti-inflammatory effect through direct inhibition of the TNF-α/NF-κB signaling pathway. These results demonstrate that MY-E@SS counteracts inflammation through directly inhibiting the TNF-α/NF-κB signaling pathway in immune cells, thereby suppressing downstream cytokine production and inflammatory activation (Fig. 9o).

6. The manuscript claims systemic physiological improvement and microbiota homeostasis restoration, but the supporting data are briefly described. A more detailed presentation of microbiome profiling, diversity metrics, and longitudinal dynamics would reinforce these conclusions.

Response: Thanks for your valuable comment. We have supplemented the manuscript with the following additional information:

(1) **Detailed data from the microbiome analysis have been provided to further validate the restoration of microbiota homeostasis.**

Line 437-477: In order to investigate the regulatory role of MY-E@SS in the gut microbiota of IBD mice, we characterized the microbial communities in mouse colonic contents using 16S rRNA gene sequencing. From 30 mice distributed across six groups, 2,400,056 high-quality 16S rDNA sequences were obtained. Sequences were resampled to an even depth and clustered into operational taxonomic units (OTUs) at 97% similarity. Comparative analysis revealed a significant microbial dysbiosis in the DSS-induced model relative to the control, characterized by 4,167 unique OTUs (Fig. 8a). In this work, we measured α-diversity using the Shannon and Simpson indices. Principal coordinate analysis (PCoA) showed that MY-E@SS administration induced a substantial shift in the gut microbiota structure of IBD mice (Fig. 8b). While individual treatments of MY-EcN and SS enhanced microbial richness, the MY-E@SS group showed significantly higher Shannon and Simpson indices than the DSS group and all other treatment groups (Fig. 8c, d). Conversely, the P-EcN group exhibited values comparable to the

DSS group, indicating a minimal effect on restoring microbial diversity in the compromised gut.

To identify differentially abundant taxa across groups, a Linear Discriminant Analysis Effect Size (LEfSe) analysis was performed. This analysis revealed key discriminant microbial taxa from the phylum to genus level (Fig. 8e). Based on LDA scores, biomarker taxa from the phylum to genus level were identified (Fig. 8e). It is noteworthy that four dominant bacterial groups in the MY-E@SS group were characterized as probiotics. Among these, Lachnospiraceae was particularly highlighted for its potential to maintain intestinal health via short-chain fatty acid production and immune response modulation.[34] Microbial composition at the phylum and genus/species levels was further visualized using bar plots and heatmaps, respectively (Fig. 8f and S35). Across all samples, 43 phyla were identified. At the phylum level, the MY-EcN, SS, and MY-E@SS treatment groups exhibited microbial composition patterns comparable to the PBS group (Fig. 8f). In the DSS group, the abundance of Firmicutes was significantly reduced, while that of Proteobacteria was markedly increased (Fig. 8g–i). In contrast, MY-EcN, SS, and MY-E@SS treatments enhanced the relative abundance of Firmicutes and effectively suppressed the abnormal proliferation of Proteobacteria. A bacterial family known for its probiotic properties, Muribaculaceae, showed higher abundance in the MY-E@SS group at the genus level (Fig. 8j and k). Its beneficial effects may arise from competitive inhibition of pathogenic microorganisms and regulation of the intestinal immune microenvironment.[35] Moreover, MY-E@SS specifically modulated several bacterial families, including Akkermansiaceae, Bacteroidaceae, Bifidobacteriaceae, Oscillospiraceae, and Prevotellaceae (Fig. 8k). To further elucidate the dynamics of the microbial community, samples collected at multiple time points during the intervention were analyzed. Analyses of β-diversity (Fig. S36a) and α-diversity (Fig. S36b, c), along with the compositional structure of the microbiota at the phylum, genus, and species levels (Fig. S37), revealed that the community structure of the MY-E@SS treatment group gradually shifted toward that of the PBS group over time, visually demonstrating the restoration of the microbiota toward homeostasis. Concurrently, the abundance of several typical probiotic bacteria in the MY-E@SS group showed an increasing trend post-intervention (Fig. S38a, b) and approached the level of the control group by the end of the experiment, whereas typical harmful bacteria exhibited an opposite trend (Fig. S38c). Thus, MY-E@SS positively contributes to disease alleviation by restoring gut microbial homeostasis, which is characterized by the reversal of diversity loss and structural disorder.

(2) Longitudinal microbiome data: Analysis of samples from additional time points was conducted to delineate the dynamic changes in the microbial community.

Line 467-475: To further elucidate the dynamics of the microbial community, samples collected at multiple time points during the intervention were analyzed. Analyses of β-diversity (Fig. S36a) and α-diversity (Fig. S36b, c), along with the compositional structure of the microbiota at the phylum, genus, and species levels (Fig. S37), revealed that the community structure of the MY-E@SS treatment group gradually shifted toward that of the PBS group over time, visually demonstrating the restoration of the microbiota toward homeostasis. Concurrently, the abundance of several typical probiotic bacteria in the MY-E@SS group showed an increasing trend post-intervention (Fig. S38a, b) and approached the level of the control group by the end

of the experiment, whereas typical harmful bacteria exhibited an opposite trend (Fig. S38c).

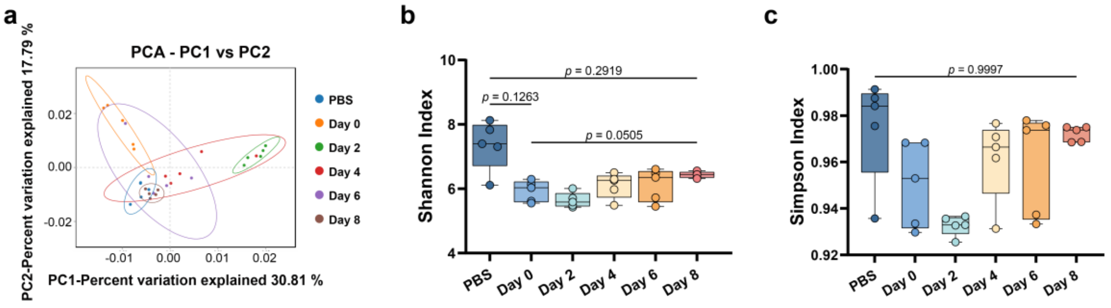

Figure S36. Analysis of microbial α- and β-diversity during MY-E@SS intervention. (a) β-diversity analysis (Principal component analysis (PCA)) illustrating the temporal shifts in community structure among different treatment groups. (b, c) α-diversity analysis using the Shannon index (b) and Simpson index (c), showing temporal trends in microbial richness and evenness within each treatment group.

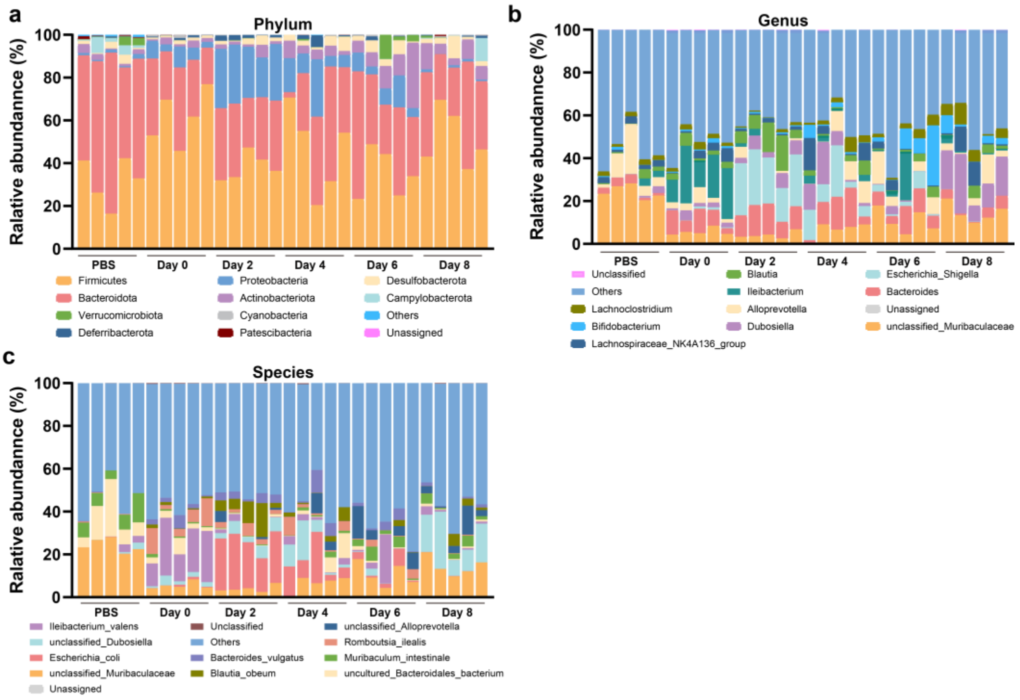

Figure S37. Microbial community composition at the phylum, genus, and species levels. Shown is the relative abundance of microbial communities across different treatment groups at various time points during the intervention, analyzed at the phylum (a), genus (b), and species (c) taxonomic ranks.

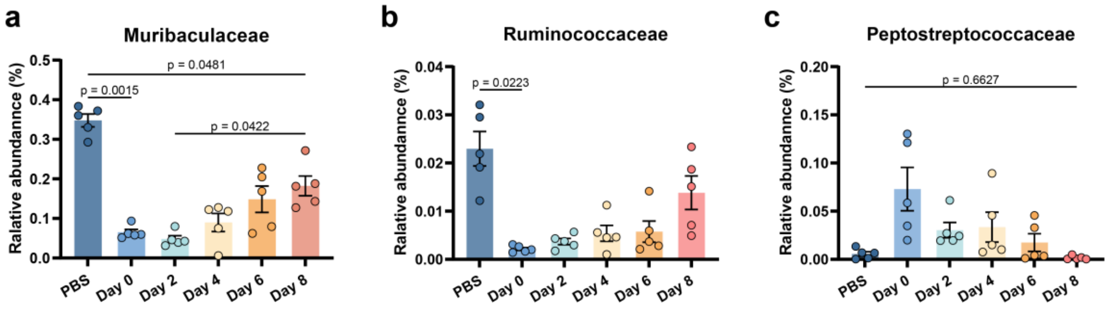

Figure S38. Temporal dynamics of typical probiotic and harmful bacterial abundances. (a-b) Post-intervention changes in the relative abundance of representative probiotic bacteria (Muribaculaceae (a), Ruminococcaceae (b)) in the MY-E@SS-treated group, showing a gradual increase over time toward the level observed in the control group. (c) Temporal trend in the abundance of a representative harmful bacterium (Peptostreptococcaceae) in the MY-E@SS-treated group, exhibiting a pattern opposite to that of the probiotics.

(3) Systemic physiological improvement data: in addition to the original spleen index, colon pathology, and respiratory and energy metabolism indicators, H&E staining of adipose tissue (indicative of metabolic improvement) was included (Fig. S24). Concurrently, serum oxidative stress markers (MDA, SOD, GSH) were measured (Fig. S23) to evaluate the recovery of systemic physiological status.

Line 301-303: Furthermore, MY-E@SS treatment systematically alleviated systemic oxidative stress in IBD mice, as indicated by increased serum levels of the antioxidant markers superoxide dismutase (SOD) and glutathione (GSH), and a decreased level of the lipid peroxidation product malondialdehyde (MDA) (Fig. S23).

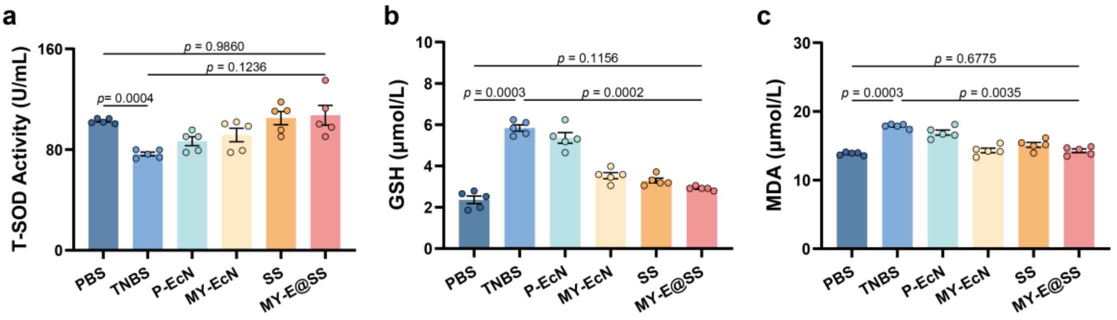

Figure S23. Changes in serum oxidative stress markers in IBD mice after different interventions. (a) Superoxide dismutase (SOD) activity, representing antioxidative enzyme capacity. (b) Glutathione (GSH) level, a major component of the non-enzymatic antioxidant system. (c) Malondialdehyde (MDA) content, reflecting the degree of lipid peroxidation.

Line 303-306: Importantly, a consistently potent therapeutic efficacy of MY-E@SS was also observed in a complementary (2,4,6-Trinitrobenzenesulfonic acid) TNBS-induced colitis model (Fig. S24), further substantiating its robust and reliable anti-inflammatory and mucosal healing properties across diverse experimental IBD settings.

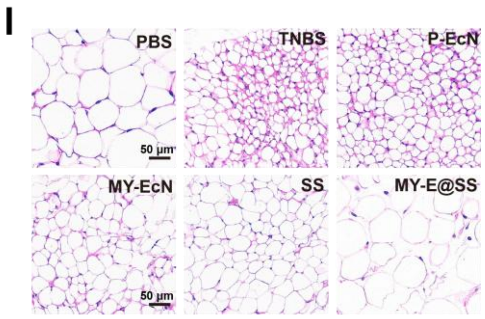

Figure S24 (l) H&E-stained sections of adipose tissue.

7. Biocompatibility and biosafety assessments are important strengths of the work;

however, long-term safety, potential off-target effects, and horizontal gene transfer risks of engineered bacteria should be discussed more comprehensively.

Response: Thank you for your positive acknowledgment of the assessment value of biocompatibility and biosafety in this work. In the revised version, we have provided a more comprehensive discussion of the long-term safety, potential off-target effects, and risks of horizontal gene transfer associated with the engineered bacteria.

Line 599-627: This work introduces a biomimetic encapsulation strategy to enhance the environmental tolerance of engineered bacteria and improve their diversity and safety. An inducible priming subsystem is used to increase the production of therapeutic proteins and enable precise controlled release in engineered strains. Concurrently, the release of anti-inflammatory peptides is regulated by the inflammatory response mechanism, thereby reducing the safety risks caused by continuous activation. This bionic encapsulation strategy further strengthens environmental isolation and broadens the operational safety boundary of engineering bacteria. Nevertheless, the off-target effect and horizontal gene transfer risk of engineered bacteria cannot be completely eliminated. In order to address these challenges and promote personalized treatment, future promoter designs can be tailored to the individual characteristics of patients. For example, inflammation-responsive promoters or self-inducible promoters can be utilized. Inflammation-responsive promoters can be specifically activated by overexpressed inflammatory factors (such as TNF-α, IL-6, NO, etc.) in the local microenvironment; the self-inducible promoter realizes the spatiotemporal regulation of gene expression through the bacterial quorum sensing system, ensuring that the therapeutic protein is only released in the target microenvironment, thereby avoiding systemic risks caused by continuous activation. In summary, by integrating biomimetic and synthetic biology strategies, our constructed MY-E@SS successfully achieves intelligent sensing and precise repair of the inflammatory microenvironment. As such, this work provides a novel and translatable feasible strategy for the collaborative management of IBD and other inflammatory diseases.

Minor comment:

1. The current title, “From Coral to Cure: Bioinspired Microcapsule Reactor with Engineered Probiotics for Smart IBD Therapy,” reads more like a feature article or a perspective piece rather than a primary research paper. The use of a slogan-like phrase (“From Coral to Cure”) adds a strong narrative or promotional tone, which may be less appropriate for a scientific research article.

Response: Thank you for your valuable feedback. In accordance with your suggestion, the manuscript title has been revised to "Bioinspired Microcapsule Reactor with Engineered Probiotics for Smart IBD Therapy" to better align with the style of an original research article.

2. Terminology inconsistencies, for example: "Intelligently detect inflammation" too vague; "Synergistic therapy" needs definition; "Physiological functions improved" specify which functions (e.g., weight, cytokines)

Response: Thanks for your kind reminder. The relevant descriptions have been

clarified and refined in the revised manuscript as follows:

(1) Regarding "Intelligently detect inflammation": In the original text, this phrase referred to the ability of the MY protein expressed by the engineered bacterial strain MY-EcN to specifically respond to neutrophil elastase (NE), which is highly expressed in the intestinal inflammatory microenvironment, leading to cleavage of the protein and release of the anti-inflammatory peptide KPV. This process mimics a biological sensing–response mechanism to pathological signals and exhibits specificity to the inflammatory microenvironment. To avoid potential ambiguity associated with the term "Intelligently detect inflammation," it has been revised to more explicitly describe the process as "intelligently sensing inflammatory signals and releasing the anti-inflammatory peptide." In the corresponding section, a brief explanation of the underlying molecular mechanism (NE-responsive cleavage) has also been added.

Line 10-12: This system integrates synthetic biology and bionic strategies, featuring engineered bacteria (MY-EcN) capable of intelligently sensing inflammatory signals and releasing the anti-inflammatory peptide KPV as the core, encapsulated within a multifunctional bionic shell.

Line 84-92: The MY protein expressed in MY-EcN was designed with a tandem peptide structure. This construct contains an Arg-Gly-Asp (RGD) sequence, which enhances cell adhesion by binding to integrin receptors on intestinal epithelial cells.[25, 26] Leveraging this property, the MY protein can be directed to target intestinal epithelial cells. Alongside the RGD motif, the protein was designed to include the inflammation-sensitive Ala-Ala-Pro-Val (AAPV) motif and the anti-inflammatory peptide Lys-Pro-Val (KPV). In the inflammatory microenvironment, the AAPV motif is specifically cleaved by the abundant neutrophil elastase (NE).[25-27] Thus, within the inflammatory colon, the AAPV-KPV tandem linkage is cleaved, thereby triggering the controlled release of KPV-a process that emulates the integrated danger-sensing and defense response of corals.

(2) Regarding "Synergistic therapy": This term here refers to the therapeutic approach in which both the biomimetic microcapsule shell and the encapsulated engineered bacteria possess distinct treatment functions, and their combined effect produces an overall therapeutic outcome superior to the sum of their individual effects. Specifically, the shell provides physical protection, targeted delivery, and localized sustained release, while the engineered bacteria are responsible for sensing inflammation and specifically releasing the anti-inflammatory peptide. Their combination not only ensures the viable delivery and colonization of the bacteria but also enhances anti-inflammatory efficacy, barrier repair, and microbiome modulation through spatiotemporal coordination. In the revised manuscript, the term has been explicitly defined, and relevant experimental evidence (e.g., comparative data from treatment groups) has been added.

Line 90-101: Thus, within the inflammatory colon, the AAPV-KPV tandem linkage is cleaved, thereby triggering the controlled release of KPV-a process that emulates the integrated danger-sensing and defense response of corals. To mimic the structure of the coral exoskeleton and mucus layer, the SS shell was synthesized via a one-pot chemical method based on silk fibroin (SF), dopamine, and mannose. Dopamine provides additional adhesive properties, while

mannose confers targeting functionality; together, they enable the SS shell to scavenge reactive oxygen and nitrogen species (RONS). Furthermore, the SS shell enhances the aggregation density of engineered bacteria and works synergistically with MY-EcN to promote tissue repair, thereby simulating the coral-derived defense – tissue repair process. This design constitutes a synergistic therapy: the shell provides protection and targeted delivery, while the engineered bacteria enable sensing-triggered KPV release. Their combination ensures effective bacterial delivery and yields therapeutic outcomes that surpass the efficacy of either component alone.

Line 401-406: After intervention, the MY-EcN, SS, and MY-E@SS groups exhibited substantial recovery, characterized by restored respiratory metabolic parameters, stabilized circadian rhythms, and enhanced fuel utilization (Fig. 7g-j and S33). Notably, the MY-E@SS group showed superior recovery across multiple metrics, outperforming each component alone, indicating a synergistic effect between MY-EcN and SS in promoting energy metabolism recovery.

Line 285-289: While MY-EcN or SS alone showed limited efficacy in intestinal repair, MY-E@SS promoted macrophage polarization from a pro-inflammatory ($CD86^{+}$) to an anti-inflammatory/reparative ($CD206^{+}/Arg1^{+}$) phenotype and increased $Foxp3^{+}CD4^{+}$ regulatory T cell infiltration, collectively rebalancing the local immune environment.

Line 582-585: The synergistic interaction between MY-EcN and SS imparts MY-E@SS with pronounced anti-inflammatory capacity, enabling RONS scavenging, prolonged intestinal colonization, resilience to gastric acidity, and markedly improved probiotic storage stability.

(3) Regarding "Physiological functions improved": This aspect has been elaborated in the Abstract, Results, and Discussion sections, with the main improvements encompassing:

Line 14-18: In IBD model, the optimized MY-E@SS demonstrated excellent biocompatibility and significant therapeutic efficacy, improving systemic physiological indicators, including enhancing intestinal barrier function, reducing systemic inflammation and oxidative stress levels, and modulating respiratory metabolism while restoring microbial homeostasis.

(4) Additionally, the terminology throughout the manuscript has been reviewed to ensure consistency and accuracy. We believe the revised version now employs more precise terminology and provides more comprehensive information. Once again, we sincerely appreciate your thorough and professional review, which has significantly enhanced the clarity and scientific rigor of this paper.

We tried our best to improve the manuscript and made some changes in the manuscript.

We appreciate for Editors/Reviewers' warm work earnestly and hope that the correction will meet with approval. Once again, thank you very much for your comments and suggestions.

Responses to the Reviewers' Comments

Dear Reviewers,

Thank you for your comments concerning our manuscript entitled "Bioinspired Microcapsule Reactor with Engineered Probiotics for Smart IBD Therapy" (Manuscript ID: NCOMMS-25-82459). We greatly appreciate your time and effort in providing constructive feedback, which has been invaluable in strengthening our work. Based on these comments, we have carefully revised the manuscript to enhance its scientific rigor and clarity. Below, please find our point-by-point responses to all reviewer comments.

Reviewer #1:

All issues have been well addressed and so I recommend its publication in the current form.

Response: We would like to thank you for taking the time to reassess our manuscript and for the positive feedback. We appreciate your recognition of our work and are grateful for the constructive comments provided during the review process, which have helped us significantly improve the quality of our paper.

Reviewer #2:

The revised manuscript is ready for publication.

Response: Thank you for your positive feedback. We are glad that the revised manuscript is now ready for publication. We appreciate your time and valuable suggestions throughout the review process.

Reviewer #3:

I appreciate the substantial effort the authors have made to address the previous concerns. Overall, the revised manuscript is significantly strengthened, particularly in terms of mechanistic clarification, quantitative validation, and control design. Below is a structured re-evaluation of each major point, with specific comments on adequacy and remaining gaps.

Response: Thank you very much for this encouraging feedback. We are delighted to hear that you appreciate the substantial effort made in the revision and that you find the manuscript significantly strengthened, especially in the areas of mechanistic clarification, quantitative validation, and control design. We have carefully considered your structured re-evaluation and the specific comments regarding any remaining gaps.

Below, we provide a point-by-point response to each of your comments, outlining how we have addressed them in the revised manuscript.

1. Rationale for Coral-Inspired Design

The authors have substantially improved the conceptual justification for selecting coral as the biomimetic model. The comparison with alternative biological systems (e.g., plant closure, immune cascades) is now more explicit, and the mapping of "exoskeleton–mucus–symbiont" to "shell–adhesion–engineered bacteria" is logically articulated.

Response: Thank you for your positive assessment. We are glad the revised coral-inspired framework is now clear and logically sound.

2. Engineering Logic of Core–Shell Structure

The engineering rationale is now systematically described, and the structure–function relationship of each component (RGD, AAPV, KPV, dopamine, mannose, SF) is clarified.

Response: Thank you for your positive assessment. We are glad that the engineering rationale and structure-function relationships are now clearly described, which has significantly improved the clarity and scientific rigor of the manuscript.

3. Quantification of Survival, Colonization, and Peptide Release

This section is significantly strengthened.

Response: Thank you for your positive feedback. We are pleased that the quantification data on survival, colonization, and peptide release are now significantly strengthened, which greatly enhances the overall quality of the manuscript.

4. Control Groups and Synergy

The inclusion of P-EcN, MY-EcN, and SS controls across multiple endpoints is appropriate and rigorous.

Response: Thank you for your positive feedback. We are glad that the inclusion of P-EcN, MY-EcN, and SS controls across multiple endpoints is considered appropriate and rigorous, which further validates the robustness of our findings.

5. Mechanistic Depth (NF-κB, Arachidonic Acid Pathway)

The addition of NF-κB pathway validation (p65 translocation, IκBα phosphorylation, TNF-α secretion) significantly strengthens the mechanistic claims.

The reviewer's original concern also mentioned arachidonic acid metabolism. The current response focuses almost exclusively on NF-κB inhibition. If the manuscript claims modulation of arachidonic acid metabolism, causal validation is still

lacking.

Response: Thank you for your insightful comment. To maintain scientific rigor, we have now removed all related claims from the manuscript and focused the mechanistic discussion solely on the NF-κB pathway, which is fully supported by our data. We believe this revision strengthens the clarity and accuracy of the study.

6. Microbiome and Systemic Physiology

This section is significantly improved.

Response: Thank you for your kind comment. We are pleased that the improved microbiome and systemic physiology data now better support our conclusions, which has substantially enhanced the overall quality of the manuscript.

7. Biosafety and Horizontal Gene Transfer

Assessment:

The discussion has been expanded and now acknowledges HGT and off-target risks.

Response: Thank you for your positive feedback. We are glad that the expanded discussion on HGT and off-target risks adequately addresses these important biosafety considerations.

We tried our best to improve the manuscript and made some changes in the manuscript.

We appreciate for the Reviewers' warm work earnestly and hope that the correction will meet with approval. Once again, thank you very much for your comments and suggestions.
